# Sex and Circadian Rhythm Dependent Behavioral Effects of Chronic Stress in Mice and Modulation of Clock Genes in the Prefrontal Cortex

**DOI:** 10.3390/ijms26136410

**Published:** 2025-07-03

**Authors:** Jessica Mingardi, Mattia Giovenzana, Noemi Nicosia, Paulina Misztak, Alessandro Ieraci, Laura Musazzi

**Affiliations:** 1 School of Medicine and Surgery, University of Milano-Bicocca, 20900 Monza, Italy; jessica.mingardi@unimib.it (J.M.); m.giovenzana6@campus.unimib.it (M.G.); n.nicosia@campus.unimib.it (N.N.); paulina.misztak@gmail.com (P.M.); 2 PhD Program in Neuroscience, School of Medicine and Surgery, University of Milano-Bicocca, 20900 Monza, Italy; 3 Department of Neuroscience, Istituto di Ricerche Farmacologiche Mario Negri IRCCS, 20156 Milan, Italy; alessandro.ieraci@uniecampus.it; 4 Department of Theoretical and Applied Sciences, eCampus University, 22060 Novedrate, Italy; 5 Fondazione IRCCS San Gerardo dei Tintori, 20900 Monza, Italy

**Keywords:** chronic stress, circadian rhythm, sex differences, depressive behavior, anxious behavior, anhedonic behavior, mice, prefrontal cortex, clock genes

## Abstract

Behavioral stress is a recognized triggering factor for systemic diseases, including psychiatric disorders. The stress response is subjected to circadian regulation and many factors shape the susceptibility to its maladaptive consequences, including the biological sex. Accordingly, circadian dysregulation of the stress response, often occurring in a sexually dimorphic manner, is typically associated with psychiatric disorders. However, the interaction between stress, sex, circadian phases, and behavior is still largely unknown. Here, we used the chronic restraint stress (CRS) model in male and female mice to assess the impact of sex and circadian phases on the behavioral consequences of chronic stress. Animals were stressed either in the light or dark phase, and anxious-/depressive-/anhedonic-like behaviors were assessed. Associated transcriptional changes in clock genes were measured in the prefrontal cortex. A significant interaction of stress, sex, and circadian phase was found in most of the parameters evaluated, with no behavioral response to stress in males stressed in the dark phase, and an exaggerated response in females stressed in the dark phase compared to the light phase. We also found some molecular changes in corticosterone serum levels and expression of clock genes in the prefrontal cortex.

## 1. Introduction

The stress response is a physiological reaction of the body towards a stressor that alters the homeostasis [1]. Nevertheless, the intensity and chronicity of the insults are decisive for challenging the individual capability to cope with stress. Accordingly, stress and especially chronic stress is recognized as a major environmental risk factor for both physical and mental health [2]. The association between chronic stress exposure and adverse mental health outcomes involves a complex interplay of biological, psychological, and behavioral mechanisms [3]. The stress response is indeed individually shaped based on the genetic background, epigenetic remodeling, personality traits, socioeconomic state, and many other factors [4]. Among these, in recent years, scientific evidence highlighted the influence of biological sex on response to different types of stress exposure [5].

In humans, the prevalence of stress-related psychiatric diseases is higher in females than in males and, despite similar rates of exposure to most types of stressors, women more often face specific interpersonal life events that increase the risk for developing psychiatric illness [6]. Moreover, ovarian hormone fluctuations were reported to modulate not only women’s susceptibility to stress, but also brain plasticity and function [5,7]. Accordingly, preclinical studies use stress protocols to induce behavioral phenotypes resembling psychiatric symptoms of depressed patients and showed specific behavioral responses, associated with different molecular signatures, between males and females. Such differences include changes in the reactivity of the hypothalamus–pituitary–adrenal (HPA) axis, epigenetic remodeling, and immune response to stress [6].

At the same time, psychiatric disorders are frequently associated with a dysregulation of circadian rhythms and sleep patterns, often occurring in a sexually dimorphic manner [8]. However, the interaction between circadian phase, stress response, sex, and depressive-like behaviors is still largely unknown.

Circadian rhythms influence a wide range of physiological and behavioral aspects and are driven by the complex interplay of a set of core clock genes. Among these, brain-and-muscle arnt-like protein 1 (*Bmal1*) codes for a transcription factor promoting the transcription of period (*Per1* and *Per2*), and cryptochrome (*Cry1* and *Cry2*) genes, which in turn activate a negative feedback suppressing *Bmal1* activity [9]. A period of approximately 24 h is achieved via a delay between peak transcription and peak translation, and via the accumulation and degradation of proteins. Interestingly, sex hormones can influence circadian phase by controlling clock genes expression [10,11].

The master clock is located in the suprachiasmatic nuclei (SCN) of the anterior hypothalamus, which receives light information by the retinohypothalamic tract and, in turn, coordinates the response both in other brain areas (including the cortex) and in peripheral organs [9]. Importantly, the prefrontal cortex (PFC), a brain area implicated in executive functions, stress response, emotion, and learning [12], as well as with depression pathogenesis [13,14], is anatomically linked to SCN and shows diurnal changes in the expression of circadian clock genes [15], with significant functional consequences. Indeed, it has been demonstrated that alterations of daily rhythms may impact fundamental properties of PFC neurons [16], as well as PFC-associated behaviors [15].

Here we used a mouse model of depression based on chronic restraint stress to study sex and circadian rhythm dependent behavioral alterations and their association with changes in the activation of the HPA and in the expression of core clock genes in the PFC. We hypothesized that chronic stress would modulate behavior and clock gene expression in a sex- and circadian phase-dependent manner.

## 2. Results

### 2.1. Sex and Circadian Rhythm Dependent Effects of Chronic Restraint Stress on Body Weight in Mice

**Figure 1:**
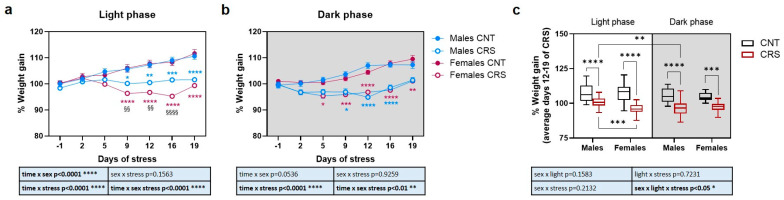
**Body weight changes in male and female mice stressed in the light or dark phase.** (**a**) Percentage of body weight gain in male and female control (CNT) and CRS mice stressed in the light phase, during the 21 days of CRS. Mixed-effects model followed by Tukey’s post hoc test: * *p* < 0.05, ** *p* < 0.01, *** *p* < 0.001, **** *p* < 0.0001 vs. CNT on the same day within sex; ^§§^ *p* < 0.01, ^§§§§^ *p* < 0.0001 vs. CRS on the same day between sexes. Data are reported as means ± SEM. (**b**) Percentage of body weight gain in male and female CNT and CRS mice stressed in the dark phase, during the 21 days of CRS. Mixed-effects model followed by Tukey’s post hoc test: * *p* < 0.05, *** *p* < 0.001, **** *p* < 0.0001 vs. CNT on the same day within sex. Data are reported as means ± SEM. (**c**) Average percentage of weight gain in male and female mice from day 12 to day 19 of CRS mice stressed in the light or dark phase. Three-way ANOVA followed by Tukey’s post hoc test: ** *p* < 0.01, *** *p* < 0.001, **** *p* < 0.0001. The statistics of the interactions between the independent variables are reported in the inserts below each graph. Full statistical details are reported in Table S1.

Male and female mice were exposed to 3 weeks of chronic restraint stress (CRS) either during their active or inactive phase (dark or light phase, respectively) and body weight was measured twice weekly (Figure 1). To neutralize obvious changes in the absolute weight (male mice weigh more than females), data are reported as a percentage compared to the mean value of the same experimental group the day before the start of CRS.

When stressed during the light phase (Figure 1a), although control animals gained weight physiologically as young adults, both male and female CRS mice showed a decreased body weight gain compared to respective controls, but with significant sex differences across the 3 weeks of stress. Indeed, while the weight of CRS male mice remained almost stable starting from 5 days of stress, female CRS mice showed a significant reduction in their weight compared to the pre-stress condition starting from the 9th day of stress, except for the recovery of part of the weight on the 19th day to values similar to the 5th day, suggesting a certain degree of adaptation. Conversely, when animals were stressed during the dark phase (Figure 1b), CRS mice showed again a reduction in weight gain but with minor sex differences and both male and female mice showed a progressive recovery of weight starting from day 16th of stress back to respective weights before the beginning of the stress protocol.

Our data suggest that, while in females the body weight is similarly affected by stress in the light or dark phase, in males a more pronounced effect is seen when the animals are stressed during the dark phase. To confirm this hypothesis, we calculated the mean weight gain from day 12 to day 19 of stress for each animal and evaluated the combined effect of sex, stress, and circadian phase on weight gain (Figure 1c). We observed that although CRS mice gained less weight than controls in all the conditions, in male mice the reduction in weight was significantly higher when the animals were stressed during the dark phase compared to when they were stressed during the light phase.

### 2.2. Sex and Circadian Rhythm Dependent Behavioral Effects of Chronic Restraint Stress in Mice

To assess sex and circadian rhythm dependent behavioral alterations induced by CRS in mice, we exposed the animals to a battery of behavioral tests aimed at assessing anxious-like (open field test, OFT), depressive-like (tail suspension test, TST and forced swim test, FST), and anhedonic-like (sucrose splash test, SST) behaviors. Behavioral evaluations were performed in the morning before CRS exposure, either in the light or dark phase depending on the specific experimental group.

**Figure 2:**
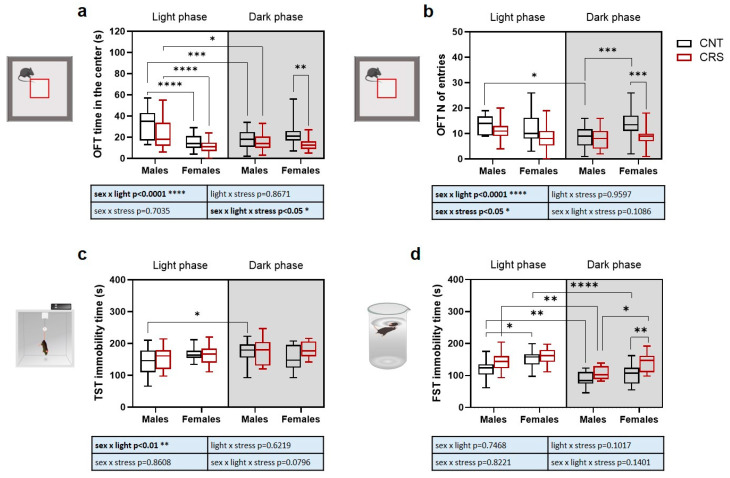
**Phenotypical characterization of anxious and depressive-like behaviors in male and female mice stressed in the light or dark phase.** (**a**) Time and (**b**) number of entries in the center of the arena during the OFT. (**c**) Immobility time in the TST and (**d**) in the FST. Three-way ANOVA followed by Tukey’s post hoc test: * *p* < 0.05, ** *p* < 0.01, *** *p* < 0.001, **** *p* < 0.0001. The statistics of the interactions between the independent variables are reported in the inserts below each graph. Full statistical details are reported in Table S2. Image provided by Servier Medical Art [17], licensed under CC BY 4.0.

As for time spent in the center of the arena in the OFT (Figure 2a), we found a significant interaction of sex, stress, and circadian phase. Major changes included significant reductions in the time spent in the center in (i) females compared to males when tested in the light phase, (ii) males when tested in the dark phase compared to light phase, and (iii) CRS females stressed during the dark phase compared to the respective control. Conversely, only simple interactions between stress and circadian phase, as well as between stress and sex, were observed for the number of entries in the central part of the arena (Figure 2b). Specifically, we observed a reduction in the number of entries in male control animals tested in the dark phase compared to light phase while, in animals tested in the dark phase, we measured an increase in the number of entries in control females compared to control males and a reduction in CRS females compared to control females.

In the TST (Figure 2c), we only found a significant effect of the interaction between stress and circadian phase, with increased immobility time in control males when tested during the dark phase compared to light phase, while more changes were observed for the time of immobility in the FST (Figure 2d). A significant effect of the three independent variables was observed with no significant interactions (Table S2). In control animals, males showed a decrease in immobility time when tested in the dark phase compared to light phase, while females showed a higher immobility than males in the light phase. In CRS mice, the immobility time was decreased in males tested in the dark phase compared to light phase while, in animals tested in the dark phase, the time of immobility was increased in females compared to males and in CRS females compared to control females.

**Figure 3:**
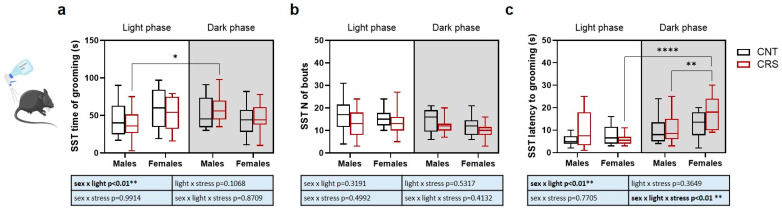
**Phenotypical characterization of anhedonic behavior in male and female mice stressed in the light or dark phase.** (**a**) Time of grooming, (**b**) number of bouts, and (**c**) latency to first event of grooming during the SST. Three-way ANOVA followed by Tukey’s post hoc test: * *p* < 0.05, ** *p* < 0.01, **** *p* < 0.0001. The statistics of the interactions between the independent variables are reported in the inserts below each graph. Full statistical details are reported in Table S3. Image provided by Servier Medical Art [17], licensed under CC BY 4.0.

In the SST, the time of grooming was increased in CRS males when tested in the dark compared to light phase (Figure 3a), no significant changes were observed in the number of bouts (Figure 3b), while the latency to grooming was higher in CRS females in the dark phase compared to both CRS females in the light phase and CRS males in the dark phase (Figure 3c).

**Figure 4:**
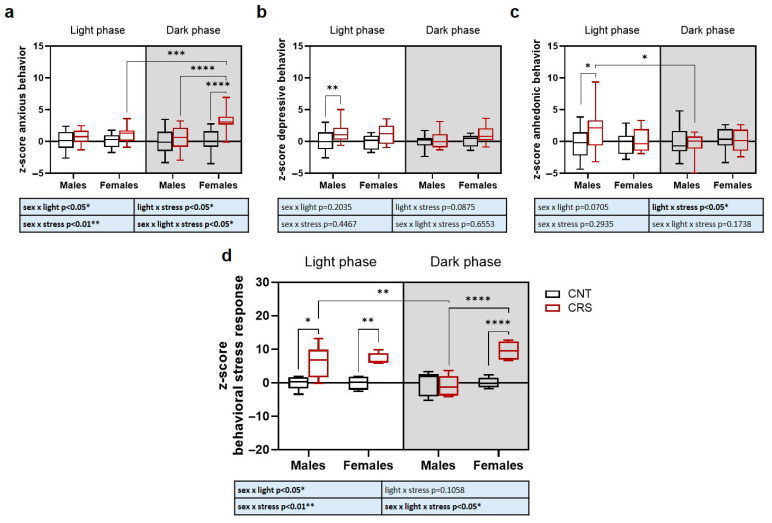
**Z-score of anxious, depressive, and anhedonic-like behaviors in male and female mice stressed in the light or dark phase.** (**a**) Z-score normalization of the parameters evaluated in the OFT (time in the center and number of entries). (**b**) Z-score normalization of the parameters evaluated in the TST and FST (immobility time in the two tests). (**c**) Z-score normalization of the parameters evaluated in the SST (time of grooming, number of bouts, and latency to grooming). (**d**) Z-score normalization of all the parameters evaluated in the behavioral tests (time in the center and number of entries in the OFT, immobility time in the TST and in the FST, time of grooming, number of bouts, and latency to grooming in the SST). Three-way ANOVA followed by Tukey’s post hoc test: * *p* < 0.05, ** *p* < 0.01, *** *p* < 0.001, **** *p* < 0.0001. The statistics of the interactions between the independent variables are reported in the inserts below each graph. Full statistical details are reported in Table S4.

Overall, our behavioral data suggest a complex interplay between sex, stress, and circadian phase depending on the specific behavioral trait analyzed. We thus applied a z-score-based approach to integrate behavioral results into three main domains: anxious-like behavior obtained by the integration of OFT results, depressive-like behavior integrating TST and FST results, and anhedonic-like behavior for SST results (Figure 4).

We found a significant interaction of sex, stress, and circadian phase for the anxious-like phenotype, with increased anxiety traits selectively in females stressed in the dark phase compared to all the other experimental groups (Figure 4a). For depressive-like behavior, we only observed a significant effect of stress, with a general increase in depressive-like traits in CRS mice which, however, reached significance only for male mice stressed in the light phase (Figure 4b). A significant stress x circadian phase interaction was revealed for anhedonic-like behavior (Figure 4c). Indeed, anhedonic-like behavior was present only in male mice stressed during the light phase and not when stressed in the dark phase.

Finally, we integrated all behavioral data as a measure of sex, stress, and circadian phase dependent behavioral stress response in mice and found a significant interaction of the three variables (Figure 4d). Interestingly, we found that in males the behavioral response was exacerbated only when the animals were stressed in the light phase but not when they were stressed in the dark phase. Conversely, in female mice, stress triggered a behavioral response both when the animals were stressed in the light and dark phases, but with a stronger effect in the dark phase.

### 2.3. Sex and Circadian Rhythm Dependent Effects of Chronic Restraint Stress on Corticosterone Serum Levels and Expression of Clock Genes in the Prefrontal Cortex of Mice

Having found that CRS exerted a differential behavioral impact on male and female mice depending on the circadian phase when stress was administered, we selected 5–7 animals per experimental group to perform molecular evaluations.

**Figure 5:**
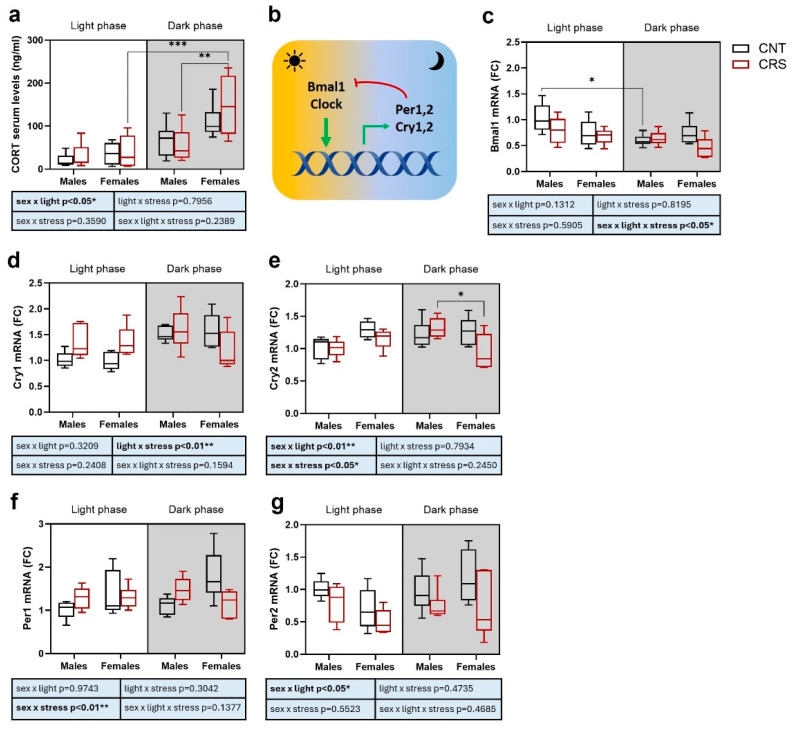
**Serum corticosterone levels and clock genes expression in the prefrontal cortex of male and female mice stressed in the light or dark phase.** (**a**) Serum corticosterone levels. (**b**) Scheme of clock genes mechanism of action in the control of circadian rhythm [9]. (**c**) *Bmal1*, (**d**) *Cry1*, (**e**) *Cry2*, (**f**) *Per1*, and (**g**) *Per2* mRNA levels in the prefrontal cortex. FC: fold change. Three-way ANOVA followed by Tukey’s post hoc test: * *p* < 0.05, ** *p* < 0.01, *** *p* < 0.001. The statistics of the interactions between the independent variables are reported in the inserts below each graph. Full statistical details are reported in Table S5.

First, we measured the serum levels of the stress hormone corticosterone as an indicator of the levels of activation of the HPA axis (Figure 5a). Interestingly, we found a significant effect of sex (higher levels in females), circadian phase (obvious higher levels in the dark phase) and interaction between sex and circadian phase, with significantly increased corticosterone serum levels in female mice stressed in the dark phase compared to males and to females stressed in the light phase.

We then asked whether behavioral and hormonal alterations were associated with changes in the expression of clock genes in the PFC and measured mRNA expression of *Bmal1*, *Cry1*, *Cry2*, *Per1*, and *Per2*.

As for *Bmal1*, as expected (Figure 5c), we found a significant effect of the circadian phase with higher levels in the light phase. We also measured a significant effect of stress and of the interaction of sex, stress, and circadian phase, with a reduction in control male mice tested in the dark phase compared to light phase (Figure 5c). The significant interaction of the three independent variables suggests that *Bmal1* levels are differentially modulated by stress in males and females depending on the circadian phase. Indeed, we can observe non-significant trends towards reduced *Bmal1* expression in male mice stressed in the light phase and in female mice stressed in the dark phase, as well as a trend towards reduction in females compared to control males in the light phase.

For *Cry1* expression, we again found a significant and expected effect of time with increased levels in the dark phase, and a significant effect of the interaction between circadian phase and stress, with trends of increased levels in animals stressed in the light phase (Figure 5d). We also observed a significant interaction between circadian phase and stress for *Cry2* (higher levels in the dark phase), and we also found a significant interaction between sex and stress, with a reduced expression exclusively in stressed females particularly in the dark phase (Figure 5e).

Differently, only a significant effect of the interaction between sex and stress was reported for *Per1* expression (Figure 5f). Indeed, CRS tend to increase *Per1* levels in males and decrease them in females independently on the circadian phase. Although *Per1* levels are expected to be higher in the dark phase than in the light phase, the significant interaction between sex and stress masks any effect of the circadian rhythm. Conversely, significant effects of stress and of the interaction between sex and circadian phase were found for *Per2* expression, whose levels were generally reduced by stress and in control male mice tested in the dark phase compared to the light phase (Figure 5g).

**Figure 6:**
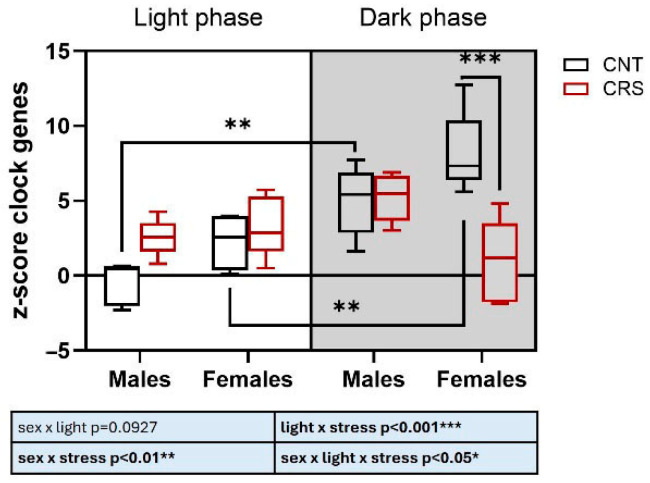
**Z-score of clock genes changes in the PFC of male and female mice stressed in the light or dark phase.** Z-score normalization of *Bmal1*, *Cry1*, *Cry2*, *Per1*, and *Per2* mRNA levels in the PFC of male and female mice stressed in the dark or light phase. Three-way ANOVA followed by Tukey’s post hoc test: ** *p* < 0.01, *** *p* < 0.001. The statistics of the interactions between the independent variables are reported in the inserts below each graph. Full statistical details are reported in Table S6.

Finally, with the aim of integrating molecular results, we applied a z-score normalization of the expression of the five analyzed clock genes (Figure 6). We found a significant effect of the interaction of the three independent variables, with a remarkable dysregulation of clock genes induced by stress in female mice stressed in the dark phase.

## 3. Discussion

To the best of our knowledge, this is the first study specifically addressing circadian rhythm dependent behavioral effects of chronic stress in male and female mice and possible molecular correlates. We found an interaction of biological sex, stress, and circadian phase in influencing body weight gain, behavioral and hormonal responses to stress, and the expression of the clock genes *Bmal1*, *Cry1*, *Cry2*, *Per1*, and *Per2* in the PFC.

Chronic restraint stress is well known to reduce body weight in both sexes in mice and rats, although with some inconsistencies [18,19,20,21,22,23]. Here we assessed, for the first time, the impact of the circadian phase on the effects of chronic restraint stress in male and female mice and found that females lose more weight than males when stressed in the light phase, while males lose more weight if stressed in the dark phase compared to the light phase.

These results suggest sex specific metabolic adaptations depending on the time of the day when the animals experience stress. Interestingly, sex differences tend to normalize towards the end of the protocol, suggesting a certain degree of adaptation, as also suggested by previous studies [21]. Previous evidence showed that the reduction in body weight induced by chronic stress is associated with a transient reduction in food consumption and with metabolic and energy homeostasis alterations [24]. We can thus speculate that, when stressed in their inactive phase (light phase), female mice may be more sensitive to metabolic alterations than males, while male mice may be more affected if stressed during their active phase (dark phase). Since we did not evaluate food intake and/or metabolic parameters in this study, this hypothesis should be verified in the future.

Interestingly, changes in body weight, appetite, and metabolic disturbances are also typical symptoms of mood disorders and often present with sexual dimorphism [25,26,27]. We thus asked whether sex and circadian rhythms could also participate in shaping the behavioral consequences of chronic stress, testing the animals in a battery of tests to evaluate changes in anxious-, depressive-, and anhedonic-like phenotypes. Of note, applying a z-score normalization, grouping behavioral readouts in the three domains, we clearly highlighted that chronic stress could exert various effects on behavior. Indeed, anxious, depressive, and anhedonic behaviors were not influenced in a concerted way but rather may follow independent trends. Although both anxious- and depressive-like behaviors were induced by stress in all the experimental conditions (significant effect of stress), the stress exposure in the dark phase clearly exacerbated anxious-like behavior selectively in females but not in males, while no sex or circadian phase dependent effects were found for depressive-like traits. Conversely, the effects of stress on anhedonic-like behavior depended only on the circadian phase and were found specifically in male mice stressed in the light phase.

Intriguingly, integrating all the behavioral results, we observed that chronic stress induced significant effects in male mice only if administered in the light phase, while female behavior was affected by stress exposure both in the light and dark phases. Accordingly, in a previous study, chronic mild stress was reported to induce depressive and anxious-like phenotypes in male Wistar rats only in the light phase, but not in the dark phase [28]. It is important to mention that in our experiments we did not consider estrous cycle, which was previously shown to significantly impact mouse behavior [29,30]. Further studies are needed to understand whether the increased behavioral sensitivity to stress in female mice depends on specific estrous phases.

The effect of circadian rhythm in the context of sexual dimorphism in behavior has been poorly explored. Animal models of mood disorders are essentially based on chronic stress exposure [31] because it is recognized to induce behavioral alterations in both males and females, although with some sex differences [23,32,33]. Indeed, preclinical and clinical evidence converge in demonstrating that sex and gender can lead to differences in stress responses that predispose males and females to different expressions of similar pathologies [6,7]. At the same time, anxiety and depressive disorders typically affect twice as many women as men, with women reporting greater severity and more atypical and somatic symptoms [34,35].

The mammalian circadian clock is based on a molecular oscillator present in virtually every cell of the body and the stress response physiologically follows the circadian rhythm as well [36]. Our data support the idea that circadian rhythm plays a crucial role in the manifestation of specific behavioral phenotypes in males and females induced by chronic stress exposure. This aspect is relevant for both preclinical research and from a translational point of view. Our data demonstrate that the laboratory environment in which animal manipulation and behavioral tests are performed can have a substantial impact on the results, thus experiments conducted in animal facilities with the daylight cycle may be incomparable to experiments conducted in a reversed light environment. This should be taken into consideration in future studies. Moreover, considering that the uncoupling of the sleep–wake cycle from natural light is a hallmark of modern society and that alterations of the circadian rhythm are not uncommon, especially in some professions, this could represent a risk factor for the development of mood disorders.

The search for possible molecular mediators involved in the behavioral changes induced by stress led us to measure corticosterone serum levels, as an evaluation of HPA axis activation [37], and the transcriptional levels of key clock genes in the PFC, a brain area involved in cognitive and emotional processes, significantly influenced by circadian rhythms [16]. In line with previous evidence [38], we found higher corticosterone serum levels in females compared to males, but we also found that chronic stress significantly increased corticosterone serum levels selectively in female mice stressed in the dark phase. If acute stress is well known to cause a rapid increase in corticosterone serum levels [39,40], chronic stress often causes adaptive mechanisms, limiting or fully preventing any changes in HPA axis activation [41,42,43]. Our data suggest that the exacerbation of the behavioral stress response in female mice stressed in the dark phase is associated with a long term hyperactivation of the HPA axis, suggesting a disruption of the physiological homeostasis [2].

Despite the small number of animals used in molecular evaluations, the study of transcriptional changes in clock genes in the PFC highlighted obvious circadian phase dependent changes, with a significant effect of the light phase for most of the analyzed genes. Moreover, we found a significant interaction of sex, stress, and circadian phase for *Bmal1* expression, a significant interaction between stress and circadian phase for *Cry1*, sex and circadian phase for *Cry2* and *Per2*, and between sex and stress for *Cry2* and *Per1.* Interestingly, we found no significant circadian changes in *Per1* levels, which were blunted by the effects of the interaction between sex and stress.

Intriguingly, integrating clock gene expression levels, we found a remarkable dysregulation in female mice stressed in the dark phase, with a complete disruption of circadian regulation. This suggests a possible functional correlation between the alteration of clock genes and the higher behavioral sensitivity to stress in the dark phase observed in females compared to males.

Although more studies involving a higher number of animals are warranted to better understand the role of these and other clock genes in the circadian effects of chronic stress in males and females, our data suggest that stress can cause a dysregulation of the circadian clock in the PFC in a sex dependent manner. Accordingly, previous studies showed that chronic stress may induce alterations in the expression of clock genes in the PFC in both mice and rats [44,45,46], although this evidence only came from male animals.

## 4. Materials and Methods

### 4.1. Animals

All the experiments were conducted in accordance with the European Community Council Directive 2010/63/UE and approved by the Italian legislation on animal experimentation (DL26/2014, authorization N 103/2022-PR). Five weeks old male and female C57BL/6 mice were purchased from Charles River (Calco, Italy) and kept in the quarantine room for one week before starting the experimental protocol. The animals were housed in same-sex groups of five and maintained under standard animal facility conditions, with temperatures at 20–22 °C and with ad libitum access to water and food. For mice subjected to CRS during the light phase, the light was set on at 7:00 a.m. and off at 7:00 p.m. while mice receiving CRS during the dark phase were exposed to light from 7:00 p.m. to 7:00 a.m. Red light was used for the shortest possible time, only to allow for handling of the animals during the experimental procedures and by placing the lamps as far away from the animals as possible to limit any stimuli during the dark phase [47].

### 4.2. Body Weight Measurements

Mice were weighed twice weekly. Data are reported as a percentage of body weight compared to the mean value of the same experimental group the day before the start of the stress protocol.

### 4.3. Chronic Restraint Stress

**Figure 7:**
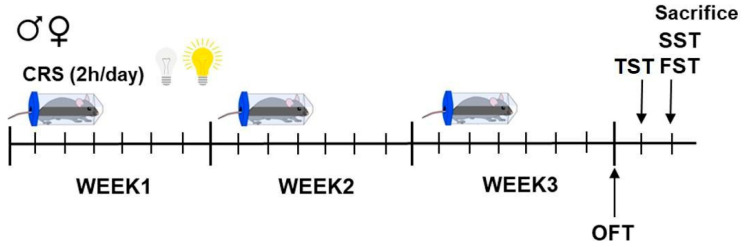
**Experimental timeline.** Male and female mice were exposed to chronic restraint stress (CRS) for 2 h every day for 21 consecutive days. Mice were weighed twice a week, and behavioral tests were performed at different timepoints. OFT: open field test; TST: tail suspension test; FST: forced swim test; SST: sucrose splash test.

The mice belonging to the CRS group were exposed for a period of 21 consecutive days to 2 h/daily of confinement within a 50 mL tube with a diameter of 5 cm, with dedicated ventilation apertures ensuring unobstructed respiration [48]. CRS was conducted randomly between 9:00 a.m. and 01:00 p.m. (ZT2–6 or ZT14–18) under standard lighting conditions for the light phase sets and red light for the dark phase sets. Control mice were left undisturbed in their cages except for behavioral tests and weight measurement. The experimental timeline is depicted in Figure 7.

### 4.4. Tail Suspension Test

The tail suspension test (TST) was conducted under dim light. Mice were hung for 6 min by the tip of the tail to a horizontal bar 1 m distant from the floor [49]. An IR camera placed in front of the mice was used for recording. The immobility time was manually recorded over the last 5 min of the video by at least two blinded experimenters.

### 4.5. Open Field Test

The open field test (OFT) was conducted in a dark squared arena (40 × 40 cm) with 20 cm high walls and a smaller illuminated square at the center. Mice were placed at the center of the arena and were allowed to move freely for 6 min [49]. An IR camera suspended above the field was used to record the test. The following parameters were manually scored during the last 5 min of the recording by two blinded experimenters: (1) time spent in the central square (sec); (2) number of times the mouse crossed the perimeter of the central square, either entering or exiting.

### 4.6. Sucrose Splash Test

For the sucrose splash test (SPT), mice were placed for 5 min in an empty cage and sprayed on the dorsal coat with a 10% (*w*/*v*) sucrose solution [50]. The test was conducted under normal lighting conditions for light phase experiments or red light for dark phase sets. The following grooming-related behaviors were measured: (1) time of grooming (sec); (2) number of bouts; (3) latency to the first grooming event (sec). After completing the test, mice were exposed to the FST.

### 4.7. Forced Swim Test

Immediately after the SST, each mouse was subjected to the forced swim test (FST) [51]. The FST lasted 5 min, during which the mouse was placed inside a cylinder of 20 cm diameter and 40 cm height, filled with 24 °C water, and allowed to move freely. After the completion of the test, the mouse was dried and returned to its cage. The test was conducted under normal lighting conditions for light phase experiments or red light for dark phase sets. The immobility time was manually recorded throughout the 5 min duration by two blinded experimenters.

### 4.8. Sacrifice

Mice were sacrificed by decapitation 1.5 h after the end of FST, in the late morning, approximately 4–6 h after light change (ZT4–ZT6 for light phase sets and ZT16–ZT18 for dark phase sets). Sacrifice was performed under normal lighting conditions for light phase experiments or red light for dark phase sets. Trunk blood was collected, centrifuged 3000 rcf for 20 min at 4 °C and stored as plasma at −80 °C. Brains were dissected on ice and the prefrontal cortex (PFC) was collected, snap-frozen in dry ice, and kept at −80 °C.

### 4.9. Corticosterone Measurement

Plasma corticosterone levels were measured as in [52].

### 4.10. RNA Isolation, Reverse Transcription, and Real-Time PCR

Total RNA was extracted from mouse PFC using Tri-Reagent (Sigma-Aldrich, Milano, Italy) and Direct-zol RNA MiniPrep (Zymo Research, Freiburg, Germany), according to the manufacturer’s instructions. Reverse transcription was carried out using the iScript cDNA Reverse Transcription kit (Bio-Rad Laboratories, Segrate, Italy). qPCR was performed using iTaq Universal SYBR Green supermix (Bio-Rad Laboratories, Segrate, Italy). Primers used for qPCR were: *Bmal1* For: CTCCAGGAGGCAAGAAGATTC, *Bmal1* Rev: ATAGTCCAGTGGAAGGAATG; *Cry1* For: AGGAGGACAGATCCCAATGGA, *Cry1* Rev: GCAACCTTCTGGATGCCTTCT; Cry2 For: GCTGGAAGCAGCCGAGGAACC, *Cry2* Rev: GGGCTTTGCTCACGGAGCGA; *Per1* For: CCAGATTGGTGGAGGTTACTGAGT, *Per1* Rev: GCGAGAGTCTTCTTGGAGCAGTAG; *Per2* For: AGAACGCGGATATGTTTGCTG, *Per2* Rev: ATCTAAGCCGCTGCACACACT; the relative expression of *Bmal1*, *Cry1*, *Cry2*, *Per1*, and *Per2*, was calculated using the comparative Ct (ΔΔCt) method and was expressed as fold change relative to control male mice tested in the light phase [53]. The mean of *Gapdh* (For: CGTGCCGCCTGGAGAAACC, Rev: TGGAAGAGTGGGAGTTGCTGTTG) and *β-actin* (For: GCCAGAGCAGTAATCTCCTTCT, Rev: AGTGTGACGTTGACATCCGTA) was used as a control reference.

### 4.11. Statistics

Statistical analysis was performed using GraphPad Prism 10 (GraphPad Software Inc., San Diego, CA, USA), applying a 3-way analysis of variance (ANOVA) followed by Tukey’s post hoc multiple comparisons test, when appropriate with time, sex, and stress as independent variables for weight gain across the 3 weeks of stress, and sex, stress, and circadian phase as independent variables for all the other evaluations.

z-scores were calculated by subtracting the mean value and then dividing the result by the standard deviation of control mice tested in the light phase for each variable. Different z-scores of the same domain were then summed. To integrate the results of RNA expression, considering that *Bmal1* expression has an opposite circadian modulation respect *Cry1*, *Cry2*, *Per1*, and *Per2*, *Bmal1* z-score was subtracted from the others.

Data are expressed as means ± standard error of the mean (SEM) for weight gain across the 3 weeks of stress or box and whiskers from minimum to maximum value for all the other evaluations. An alpha level of 0.05 was used for all statistical tests and statistical significance was assumed at *p* < 0.05.

Statistical details for each figure are reported in Supplementary Tables as indicated in figure legends.

## 5. Conclusions

We are aware of the limitations of our study such as (a) the limited battery of tests used to evaluate anxious-, depressive-, and anhedonic-like behaviors, (b) the small number of animals and clock genes, as well as the selection of a single brain area in molecular evaluations, and (c) the lack of consideration of a possible impact of estrous cycle (and associated hormonal waives) on the results obtained in female animals.

Nevertheless, our results show, for the first time in the literature, sex and circadian rhythm dependent behavioral changes induced by chronic restraint stress in mice and identify some possible hormonal and molecular correlates. Further studies are required to understand the specific circadian rhythm dependent determinants of different stress behavioral susceptibility in the two sexes. However, based on our results, we recommend taking into consideration both biological sex and the circadian phase in the study of behavioral and molecular effects of stress, and hypothesize that such an approach could improve consistency across studies and help identify druggable targets for the personalized therapy of stress-related disorders.

## Data Availability

The data presented in this study are available on request from the corresponding author. The data are not publicly available due to the fact that no big data were produced in this study.

## Supplementary Materials

The following supporting information can be downloaded at: https://www.mdpi.com/article/10.3390/ijms26136410/s1.

## Abbreviations

The following abbreviations are used in this manuscript:

CRS	Chronic Restraint Stress
FST	Forced swim test
HPA	Hypothalamic–pituitary–adrenal axis
OFT	Open Field test
PFC	Prefrontal cortex
SCN	Suprachiasmatic nucleus
SST	Sucrose splash test
TST	Tail suspension test
