# Supplementary material for: Sex and Circadian Rhythm Dependent Behavioral Effects of Chronic Stress in Mice and Modulation of Clock Genes in the Prefrontal Cortex
Source: Int J Mol Sci. 2025 Jul 3;26(13):6410. doi: 10.3390/ijms26136410 (PMC12250008; doi:10.3390/ijms26136410)
Supplement: Supplementary file 1 [file ijms-26-06410-s001.zip › Table S1.pdf]

Supplementary Table S1

Statistics of Figure 1a

Table Analyzed	% Weight gain – Light phase				
Mixed-effects model (REML)	Matching by factor: time				
Assume sphericity?	No				
Alpha	0,05				
Fixed effects (type III)	P value	P value summary	Statistically significant (P < 0,05)?	F (DFn, DFd)	Geisser-Greenhouse's epsilon
time	<0,0001	****	Yes	F (3,625, 502,6) = 63,66	0,6041
sex	0,1863	ns	No	F (1, 141) = 1,764	
stress	<0,0001	****	Yes	F (1, 141) = 60,94	
time x sex	<0,0001	****	Yes	F (6, 832) = 12,45	
time x stress	<0,0001	****	Yes	F (6, 832) = 86,24	
sex x stress	0,1563	ns	No	F (1, 141) = 2,031	
time x sex x stress	<0,0001	****	Yes	F (6, 832) = 9,022	
Random effects	SD	Variance			
Subject	4,375	19,14			
Residual	2,702	7,303			
Was the matching effective?					
Chi-square, df	840,5, 1				
P value	<0,0001				
P value summary	****				
Is there significant matching (P < 0.05)?	Yes				
Data summary					
Number of columns	2 x 2				
Number of rows (time)	7				
Number of subjects (Subject)	145				
Number of missing values	14				

Compare each cell mean with every other cell mean								
Number of families	1							
Number of comparisons per family	378							
Alpha	0,05							
Tukey's multiple comparisons test	Mean Diff,	95,00% CI of diff,	Below threshold?	Summary	Adjusted P Value			
-1:Males CNT Light phase vs. -1:Males CRS Light phase	1,627	-3,610 to 6,864	No	ns	>0,9999			
-1:Males CNT Light phase vs. -1:Females CNT Light phase	0,0003521	-6,658 to 6,658	No	ns	>0,9999			
-1:Males CNT Light phase vs. -1:Females CRS Light phase	-0,3635	-6,055 to 5,328	No	ns	>0,9999			
-1:Males CNT Light phase vs. 2:Males CNT Light phase	-1,917	-3,756 to -0,07790	Yes	*	0,0338			
-1:Males CNT Light phase vs. 2:Males CRS Light phase	-0,8023	-6,097 to 4,492	No	ns	>0,9999			
-1:Males CNT Light phase vs. 2:Females CNT Light phase	-2,838	-9,334 to 3,658	No	ns	0,9934			
-1:Males CNT Light phase vs. 2:Females CRS Light phase	-2,134	-7,857 to 3,588	No	ns	0,9992			
-1:Males CNT Light phase vs. 5:Males CNT Light phase	-4,694	-8,562 to -0,8268	Yes	**	0,0063			

-1:Males CNT Light phase vs. 5:Males CRS Light phase	-1,622	-6,988 to 3,744	No	ns	>0,9999			
-1:Males CNT Light phase vs. 5:Females CNT Light phase	-3,375	-10,15 to 3,401	No	ns	0,9678			
-1:Males CNT Light phase vs. 5:Females CRS Light phase	0,1154	-5,402 to 5,633	No	ns	>0,9999			
-1:Males CNT Light phase vs. 9:Males CNT Light phase	-5,568	-10,13 to -1,009	Yes	**	0,0058			
-1:Males CNT Light phase vs. 9:Males CRS Light phase	-0,1425	-5,341 to 5,056	No	ns	>0,9999			
-1:Males CNT Light phase vs. 9:Females CNT Light phase	-6,003	-13,23 to 1,223	No	ns	0,2420			
-1:Males CNT Light phase vs. 9:Females CRS Light phase	3,657	-1,854 to 9,167	No	ns	0,6463			
-1:Males CNT Light phase vs. 12:Males CNT Light phase	-7,302	-11,53 to -3,077	Yes	****	<0,0001			
-1:Males CNT Light phase vs. 12:Males CRS Light phase	-0,4706	-5,692 to 4,750	No	ns	>0,9999			
-1:Males CNT Light phase vs. 12:Females CNT Light phase	-7,748	-14,68 to -0,8188	Yes	*	0,0132			
-1:Males CNT Light phase vs. 12:Females CRS Light phase	3,279	-2,210 to 8,769	No	ns	0,8110			
-1:Males CNT Light phase vs. 16:Males CNT Light phase	-9,275	-13,52 to -5,025	Yes	****	<0,0001			
-1:Males CNT Light phase vs. 16:Males CRS Light phase	-1,518	-6,801 to 3,766	No	ns	>0,9999			
-1:Males CNT Light phase vs. 16:Females CNT Light phase	-8,247	-15,18 to -1,313	Yes	**	0,0055			
-1:Males CNT Light phase vs. 16:Females CRS Light phase	4,760	-0,7977 to 10,32	No	ns	0,1909			
-1:Males CNT Light phase vs. 19:Males CNT Light phase	-10,90	-14,69 to -7,109	Yes	****	<0,0001			
-1:Males CNT Light phase vs. 19:Males CRS Light phase	-1,596	-7,003 to 3,811	No	ns	>0,9999			
-1:Males CNT Light phase vs. 19:Females CNT Light phase	-11,64	-19,02 to -4,266	Yes	****	<0,0001			
-1:Males CNT Light phase vs. 19:Females CRS Light phase	0,6524	-4,817 to 6,122	No	ns	>0,9999			
-1:Males CRS Light phase vs. -1:Females CNT Light phase	-1,627	-6,819 to 3,566	No	ns	>0,9999			
-1:Males CRS Light phase vs. -1:Females CRS Light phase	-1,990	-5,616 to 1,635	No	ns	0,9228			
-1:Males CRS Light phase vs. 2:Males CNT Light phase	-3,544	-8,640 to 1,552	No	ns	0,5363			
-1:Males CRS Light phase vs. 2:Males CRS Light phase	-2,611	-4,015 to -1,207	Yes	****	<0,0001			
-1:Males CRS Light phase vs. 2:Females CNT Light phase	-4,465	-9,426 to 0,4956	No	ns	0,1271			
-1:Males CRS Light phase vs. 2:Females CRS Light phase	-3,761	-7,443 to -0,07932	Yes	*	0,0395			
-1:Males CRS Light phase vs. 5:Males CNT Light phase	-6,321	-11,24 to -1,406	Yes	**	0,0022			
-1:Males CRS Light phase vs. 5:Males CRS Light phase	-3,217	-5,781 to -0,6538	Yes	**	0,0026			
-1:Males CRS Light phase vs. 5:Females CNT Light phase	-5,002	-10,36 to 0,3556	No	ns	0,0936			
-1:Males CRS Light phase vs. 5:Females CRS Light phase	-1,512	-4,800 to 1,777	No	ns	0,9901			
-1:Males CRS Light phase vs. 9:Males CNT Light phase	-7,195	-12,48 to -1,908	Yes	***	0,0010			
-1:Males CRS Light phase vs. 9:Males CRS Light phase	-1,678	-3,759 to 0,4024	No	ns	0,2866			
-1:Males CRS Light phase vs. 9:Females CNT Light phase	-7,630	-13,59 to -1,669	Yes	**	0,0026			
-1:Males CRS Light phase vs. 9:Females CRS Light phase	2,030	-1,243 to 5,303	No	ns	0,8001			
-1:Males CRS Light phase vs. 12:Males CNT Light phase	-8,929	-14,04 to -3,818	Yes	****	<0,0001			
-1:Males CRS Light phase vs. 12:Males CRS Light phase	-1,958	-4,070 to 0,1540	No	ns	0,1033			
-1:Males CRS Light phase vs. 12:Females CNT Light phase	-9,375	-14,94 to -3,807	Yes	****	<0,0001			
-1:Males CRS Light phase vs. 12:Females CRS Light phase	1,653	-1,577 to 4,882	No	ns	0,9649			
-1:Males CRS Light phase vs. 16:Males CNT Light phase	-10,61	-16,01 to -5,212	Yes	****	<0,0001			
-1:Males CRS Light phase vs. 16:Males CRS Light phase	-2,986	-5,206 to -0,7649	Yes	***	0,0008			
-1:Males CRS Light phase vs. 16:Females CNT Light phase	-9,874	-15,45 to -4,300	Yes	****	<0,0001			
-1:Males CRS Light phase vs. 16:Females CRS Light phase	3,133	-0,2351 to 6,501	No	ns	0,1033			
-1:Males CRS Light phase vs. 19:Males CNT Light phase	-12,24	-17,45 to -7,018	Yes	****	<0,0001			
-1:Males CRS Light phase vs. 19:Males CRS Light phase	-3,072	-5,203 to -0,9418	Yes	***	0,0003			
-1:Males CRS Light phase vs. 19:Females CNT Light phase	-13,27	-19,42 to -7,114	Yes	****	<0,0001			
-1:Males CRS Light phase vs. 19:Females CRS Light phase	-0,9745	-4,162 to 2,213	No	ns	>0,9999			
-1:Females CNT Light phase vs. -1:Females CRS Light phase	-0,3639	-6,016 to 5,288	No	ns	>0,9999			
-1:Females CNT Light phase vs. 2:Males CNT Light phase	-1,917	-8,475 to 4,640	No	ns	>0,9999			
-1:Females CNT Light phase vs. 2:Males CRS Light phase	-0,8027	-6,053 to 4,448	No	ns	>0,9999			
-1:Females CNT Light phase vs. 2:Females CNT Light phase	-2,839	-5,370 to -0,3072	Yes	*	0,0158			

-1:Females CNT Light phase vs. 2:Females CRS Light phase	-2,135	-7,817 to 3,548	No	ns	0,9992			
-1:Females CNT Light phase vs. 5:Males CNT Light phase	-4,695	-11,13 to 1,737	No	ns	0,4828			
-1:Females CNT Light phase vs. 5:Males CRS Light phase	-1,623	-6,945 to 3,700	No	ns	>0,9999			
-1:Females CNT Light phase vs. 5:Females CNT Light phase	-3,376	-5,799 to -0,9516	Yes	**	0,0010			
-1:Females CNT Light phase vs. 5:Females CRS Light phase	0,1151	-5,361 to 5,591	No	ns	>0,9999			
-1:Females CNT Light phase vs. 9:Males CNT Light phase	-5,569	-12,26 to 1,125	No	ns	0,2406			
-1:Females CNT Light phase vs. 9:Males CRS Light phase	-0,1429	-5,296 to 5,010	No	ns	>0,9999			
-1:Females CNT Light phase vs. 9:Females CNT Light phase	-6,003	-9,166 to -2,840	Yes	****	<0,0001			
-1:Females CNT Light phase vs. 9:Females CRS Light phase	3,656	-1,812 to 9,125	No	ns	0,6333			
-1:Females CNT Light phase vs. 12:Males CNT Light phase	-7,302	-13,87 to -0,7346	Yes	*	0,0142			
-1:Females CNT Light phase vs. 12:Males CRS Light phase	-0,4710	-5,647 to 4,705	No	ns	>0,9999			
-1:Females CNT Light phase vs. 12:Females CNT Light phase	-7,748	-10,32 to -5,180	Yes	****	<0,0001			
-1:Females CNT Light phase vs. 12:Females CRS Light phase	3,279	-2,168 to 8,726	No	ns	0,8016			
-1:Females CNT Light phase vs. 16:Males CNT Light phase	-8,986	-15,76 to -2,214	Yes	***	0,0010			
-1:Females CNT Light phase vs. 16:Males CRS Light phase	-1,518	-6,758 to 3,721	No	ns	>0,9999			
-1:Females CNT Light phase vs. 16:Females CNT Light phase	-8,247	-11,76 to -4,738	Yes	****	<0,0001			
-1:Females CNT Light phase vs. 16:Females CRS Light phase	4,760	-0,7567 to 10,28	No	ns	0,1813			
-1:Females CNT Light phase vs. 19:Males CNT Light phase	-10,61	-17,25 to -3,969	Yes	****	<0,0001			
-1:Females CNT Light phase vs. 19:Males CRS Light phase	-1,596	-6,961 to 3,768	No	ns	>0,9999			
-1:Females CNT Light phase vs. 19:Females CNT Light phase	-11,64	-14,60 to -8,687	Yes	****	<0,0001			
-1:Females CNT Light phase vs. 19:Females CRS Light phase	0,6521	-4,775 to 6,079	No	ns	>0,9999			
-1:Females CRS Light phase vs. 2:Males CNT Light phase	-1,553	-7,119 to 4,012	No	ns	>0,9999			
-1:Females CRS Light phase vs. 2:Males CRS Light phase	-0,4388	-4,157 to 3,279	No	ns	>0,9999			
-1:Females CRS Light phase vs. 2:Females CNT Light phase	-2,475	-7,920 to 2,971	No	ns	0,9882			
-1:Females CRS Light phase vs. 2:Females CRS Light phase	-1,771	-4,587 to 1,045	No	ns	0,7134			
-1:Females CRS Light phase vs. 5:Males CNT Light phase	-4,331	-9,736 to 1,075	No	ns	0,3006			
-1:Females CRS Light phase vs. 5:Males CRS Light phase	-1,259	-5,092 to 2,574	No	ns	>0,9999			
-1:Females CRS Light phase vs. 5:Females CNT Light phase	-3,012	-8,812 to 2,788	No	ns	0,9450			
-1:Females CRS Light phase vs. 5:Females CRS Light phase	0,4790	-2,822 to 3,780	No	ns	>0,9999			
-1:Females CRS Light phase vs. 9:Males CNT Light phase	-5,205	-10,94 to 0,5318	No	ns	0,1240			
-1:Females CRS Light phase vs. 9:Males CRS Light phase	0,2210	-3,340 to 3,782	No	ns	>0,9999			
-1:Females CRS Light phase vs. 9:Females CNT Light phase	-5,639	-11,99 to 0,7110	No	ns	0,1452			
-1:Females CRS Light phase vs. 9:Females CRS Light phase	4,020	1,205 to 6,836	Yes	***	0,0004			
-1:Females CRS Light phase vs. 12:Males CNT Light phase	-6,939	-12,52 to -1,360	Yes	**	0,0029			
-1:Females CRS Light phase vs. 12:Males CRS Light phase	-0,1071	-3,706 to 3,491	No	ns	>0,9999			
-1:Females CRS Light phase vs. 12:Females CNT Light phase	-7,384	-13,37 to -1,394	Yes	**	0,0035			
-1:Females CRS Light phase vs. 12:Females CRS Light phase	3,643	0,9774 to 6,309	Yes	***	0,0009			
-1:Females CRS Light phase vs. 16:Males CNT Light phase	-8,622	-14,46 to -2,786	Yes	***	0,0002			
-1:Females CRS Light phase vs. 16:Males CRS Light phase	-1,154	-4,855 to 2,547	No	ns	>0,9999			
-1:Females CRS Light phase vs. 16:Females CNT Light phase	-7,883	-13,88 to -1,888	Yes	**	0,0013			
-1:Females CRS Light phase vs. 16:Females CRS Light phase	5,123	1,258 to 8,989	Yes	**	0,0014			
-1:Females CRS Light phase vs. 19:Males CNT Light phase	-10,24	-15,92 to -4,574	Yes	****	<0,0001			
-1:Females CRS Light phase vs. 19:Males CRS Light phase	-1,233	-5,133 to 2,668	No	ns	>0,9999			
-1:Females CRS Light phase vs. 19:Females CNT Light phase	-11,28	-17,81 to -4,749	Yes	****	<0,0001			
-1:Females CRS Light phase vs. 19:Females CRS Light phase	1,016	-1,791 to 3,823	No	ns	0,9991			
2:Males CNT Light phase vs. 2:Males CRS Light phase	1,114	-4,040 to 6,269	No	ns	>0,9999			
2:Males CNT Light phase vs. 2:Females CNT Light phase	-0,9214	-7,314 to 5,471	No	ns	>0,9999			
2:Males CNT Light phase vs. 2:Females CRS Light phase	-0,2174	-5,814 to 5,380	No	ns	>0,9999			
2:Males CNT Light phase vs. 5:Males CNT Light phase	-2,778	-6,396 to 0,8410	No	ns	0,3218			

2:Males CNT Light phase vs. 5:Males CRS Light phase	0,2945	-4,935 to 5,523	No	ns	>0,9999				
2:Males CNT Light phase vs. 5:Females CNT Light phase	-1,458	-8,136 to 5,220	No	ns	>0,9999				
2:Males CNT Light phase vs. 5:Females CRS Light phase	2,032	-3,354 to 7,418	No	ns	0,9989				
2:Males CNT Light phase vs. 9:Males CNT Light phase	-3,651	-8,099 to 0,7961	No	ns	0,2221				
2:Males CNT Light phase vs. 9:Males CRS Light phase	1,774	-3,281 to 6,830	No	ns	0,9994				
2:Males CNT Light phase vs. 9:Females CNT Light phase	-4,086	-11,22 to 3,051	No	ns	0,8769				
2:Males CNT Light phase vs. 9:Females CRS Light phase	5,574	0,1953 to 10,95	Yes	*	0,0342				
2:Males CNT Light phase vs. 12:Males CNT Light phase	-5,385	-9,527 to -1,244	Yes	**	0,0026				
2:Males CNT Light phase vs. 12:Males CRS Light phase	1,446	-3,633 to 6,525	No	ns	>0,9999				
2:Males CNT Light phase vs. 12:Females CNT Light phase	-5,831	-12,67 to 1,003	No	ns	0,2021				
2:Males CNT Light phase vs. 12:Females CRS Light phase	5,196	-0,1603 to 10,55	No	ns	0,0676				
2:Males CNT Light phase vs. 16:Males CNT Light phase	-7,367	-11,81 to -2,926	Yes	****	<0,0001				
2:Males CNT Light phase vs. 16:Males CRS Light phase	0,3991	-4,745 to 5,543	No	ns	>0,9999				
2:Males CNT Light phase vs. 16:Females CNT Light phase	-6,330	-13,17 to 0,5090	No	ns	0,1058				
2:Males CNT Light phase vs. 16:Females CRS Light phase	6,677	1,250 to 12,10	Yes	**	0,0036				
2:Males CNT Light phase vs. 19:Males CNT Light phase	-8,990	-13,18 to -4,802	Yes	****	<0,0001				
2:Males CNT Light phase vs. 19:Males CRS Light phase	0,3207	-4,951 to 5,592	No	ns	>0,9999				
2:Males CNT Light phase vs. 19:Females CNT Light phase	-9,725	-17,01 to -2,436	Yes	***	0,0009				
2:Males CNT Light phase vs. 19:Females CRS Light phase	2,569	-2,766 to 7,905	No	ns	0,9725				
2:Males CRS Light phase vs. 2:Females CNT Light phase	-2,036	-7,058 to 2,986	No	ns	0,9962				
2:Males CRS Light phase vs. 2:Females CRS Light phase	-1,332	-5,105 to 2,441	No	ns	0,9998				
2:Males CRS Light phase vs. 5:Males CNT Light phase	-3,892	-8,869 to 1,085	No	ns	0,3259				
2:Males CRS Light phase vs. 5:Males CRS Light phase	-0,8200	-3,066 to 1,426	No	ns	0,9995				
2:Males CRS Light phase vs. 5:Females CNT Light phase	-2,573	-7,986 to 2,840	No	ns	0,9714				
2:Males CRS Light phase vs. 5:Females CRS Light phase	0,9178	-2,475 to 4,310	No	ns	>0,9999				
2:Males CRS Light phase vs. 9:Males CNT Light phase	-4,766	-10,11 to 0,5776	No	ns	0,1369				
2:Males CRS Light phase vs. 9:Males CRS Light phase	0,6598	-1,508 to 2,828	No	ns	>0,9999				
2:Males CRS Light phase vs. 9:Females CNT Light phase	-5,200	-11,21 to 0,8093	No	ns	0,1688				
2:Males CRS Light phase vs. 9:Females CRS Light phase	4,459	1,081 to 7,837	Yes	***	0,0008				
2:Males CRS Light phase vs. 12:Males CNT Light phase	-6,500	-11,67 to -1,330	Yes	**	0,0030				
2:Males CRS Light phase vs. 12:Males CRS Light phase	0,3317	-2,017 to 2,681	No	ns	>0,9999				
2:Males CRS Light phase vs. 12:Females CNT Light phase	-6,946	-12,57 to -1,325	Yes	**	0,0040				
2:Males CRS Light phase vs. 12:Females CRS Light phase	4,082	0,7456 to 7,418	Yes	**	0,0031				
2:Males CRS Light phase vs. 16:Males CNT Light phase	-8,183	-13,64 to -2,728	Yes	***	0,0002				
2:Males CRS Light phase vs. 16:Males CRS Light phase	-0,7154	-3,238 to 1,807	No	ns	>0,9999				
2:Males CRS Light phase vs. 16:Females CNT Light phase	-7,444	-13,07 to -1,818	Yes	**	0,0014				
2:Males CRS Light phase vs. 16:Females CRS Light phase	5,562	2,093 to 9,032	Yes	****	<0,0001				
2:Males CRS Light phase vs. 19:Males CNT Light phase	-9,806	-15,08 to -4,532	Yes	****	<0,0001				
2:Males CRS Light phase vs. 19:Males CRS Light phase	-1,356	-3,909 to 1,196	No	ns	0,9224				
2:Males CRS Light phase vs. 19:Females CNT Light phase	-10,84	-17,04 to -4,638	Yes	****	<0,0001				
2:Males CRS Light phase vs. 19:Females CRS Light phase	1,455	-1,841 to 4,751	No	ns	0,9948				
2:Females CNT Light phase vs. 2:Females CRS Light phase	0,7039	-4,774 to 6,182	No	ns	>0,9999				
2:Females CNT Light phase vs. 5:Males CNT Light phase	-1,856	-8,118 to 4,406	No	ns	>0,9999				
2:Females CNT Light phase vs. 5:Males CRS Light phase	1,216	-3,882 to 6,314	No	ns	>0,9999				
2:Females CNT Light phase vs. 5:Females CNT Light phase	-0,5370	-2,227 to 1,152	No	ns	0,9997				
2:Females CNT Light phase vs. 5:Females CRS Light phase	2,954	-2,307 to 8,214	No	ns	0,8861				
2:Females CNT Light phase vs. 9:Males CNT Light phase	-2,730	-9,263 to 3,803	No	ns	0,9964				
2:Females CNT Light phase vs. 9:Males CRS Light phase	2,696	-2,224 to 7,615	No	ns	0,8870				
2:Females CNT Light phase vs. 9:Females CNT Light phase	-3,165	-5,478 to -0,8516	Yes	**	0,0013				

2:Females CNT Light phase vs. 9:Females CRS Light phase	6,495	1,242 to 11,75	Yes	**	0,0034			
2:Females CNT Light phase vs. 12:Males CNT Light phase	-4,464	-10,87 to 1,939	No	ns	0,5748			
2:Females CNT Light phase vs. 12:Males CRS Light phase	2,368	-2,576 to 7,311	No	ns	0,9681			
2:Females CNT Light phase vs. 12:Females CNT Light phase	-4,910	-7,504 to -2,316	Yes	****	<0,0001			
2:Females CNT Light phase vs. 12:Females CRS Light phase	6,118	0,8874 to 11,35	Yes	**	0,0076			
2:Females CNT Light phase vs. 16:Males CNT Light phase	-6,147	-12,76 to 0,4669	No	ns	0,1018			
2:Females CNT Light phase vs. 16:Males CRS Light phase	1,320	-3,690 to 6,331	No	ns	>0,9999			
2:Females CNT Light phase vs. 16:Females CNT Light phase	-5,409	-8,317 to -2,500	Yes	****	<0,0001			
2:Females CNT Light phase vs. 16:Females CRS Light phase	7,598	2,295 to 12,90	Yes	***	0,0003			
2:Females CNT Light phase vs. 19:Males CNT Light phase	-7,770	-14,25 to -1,293	Yes	**	0,0049			
2:Females CNT Light phase vs. 19:Males CRS Light phase	1,242	-3,900 to 6,385	No	ns	>0,9999			
2:Females CNT Light phase vs. 19:Females CNT Light phase	-8,803	-11,97 to -5,636	Yes	****	<0,0001			
2:Females CNT Light phase vs. 19:Females CRS Light phase	3,491	-1,718 to 8,699	No	ns	0,6301			
2:Females CRS Light phase vs. 5:Males CNT Light phase	-2,560	-7,998 to 2,878	No	ns	0,9823			
2:Females CRS Light phase vs. 5:Males CRS Light phase	0,5119	-3,374 to 4,397	No	ns	>0,9999			
2:Females CRS Light phase vs. 5:Females CNT Light phase	-1,241	-7,071 to 4,589	No	ns	>0,9999			
2:Females CRS Light phase vs. 5:Females CRS Light phase	2,250	-0,1459 to 4,645	No	ns	0,0892			
2:Females CRS Light phase vs. 9:Males CNT Light phase	-3,434	-9,201 to 2,333	No	ns	0,8268			
2:Females CRS Light phase vs. 9:Males CRS Light phase	1,992	-1,627 to 5,610	No	ns	0,9177			
2:Females CRS Light phase vs. 9:Females CNT Light phase	-3,868	-10,25 to 2,508	No	ns	0,7931			
2:Females CRS Light phase vs. 9:Females CRS Light phase	5,791	3,582 to 8,000	Yes	****	<0,0001			
2:Females CRS Light phase vs. 12:Males CNT Light phase	-5,168	-10,78 to 0,4422	No	ns	0,1096			
2:Females CRS Light phase vs. 12:Males CRS Light phase	1,664	-1,992 to 5,319	No	ns	0,9900			
2:Females CRS Light phase vs. 12:Females CNT Light phase	-5,614	-11,63 to 0,4048	No	ns	0,0975			
2:Females CRS Light phase vs. 12:Females CRS Light phase	5,414	2,967 to 7,861	Yes	****	<0,0001			
2:Females CRS Light phase vs. 16:Males CNT Light phase	-6,851	-12,72 to -0,9859	Yes	**	0,0076			
2:Females CRS Light phase vs. 16:Males CRS Light phase	0,6165	-3,139 to 4,372	No	ns	>0,9999			
2:Females CRS Light phase vs. 16:Females CNT Light phase	-6,112	-12,14 to -0,08840	Yes	*	0,0429			
2:Females CRS Light phase vs. 16:Females CRS Light phase	6,894	3,168 to 10,62	Yes	****	<0,0001			
2:Females CRS Light phase vs. 19:Males CNT Light phase	-8,474	-14,18 to -2,772	Yes	***	0,0001			
2:Females CRS Light phase vs. 19:Males CRS Light phase	0,5382	-3,414 to 4,490	No	ns	>0,9999			
2:Females CRS Light phase vs. 19:Females CNT Light phase	-9,507	-16,06 to -2,953	Yes	***	0,0002			
2:Females CRS Light phase vs. 19:Females CRS Light phase	2,787	-0,2331 to 5,807	No	ns	0,1034			
5:Males CNT Light phase vs. 5:Males CRS Light phase	3,072	-1,982 to 8,126	No	ns	0,7854			
5:Males CNT Light phase vs. 5:Females CNT Light phase	1,319	-5,236 to 7,875	No	ns	>0,9999			
5:Males CNT Light phase vs. 5:Females CRS Light phase	4,810	-0,4086 to 10,03	No	ns	0,1080			
5:Males CNT Light phase vs. 9:Males CNT Light phase	-0,8739	-2,670 to 0,9223	No	ns	0,9450			
5:Males CNT Light phase vs. 9:Males CRS Light phase	4,552	-0,3212 to 9,425	No	ns	0,0934			
5:Males CNT Light phase vs. 9:Females CNT Light phase	-1,308	-8,334 to 5,717	No	ns	>0,9999			
5:Males CNT Light phase vs. 9:Females CRS Light phase	8,351	3,141 to 13,56	Yes	****	<0,0001			
5:Males CNT Light phase vs. 12:Males CNT Light phase	-2,608	-5,342 to 0,1262	No	ns	0,0756			
5:Males CNT Light phase vs. 12:Males CRS Light phase	4,224	-0,6737 to 9,121	No	ns	0,1745			
5:Males CNT Light phase vs. 12:Females CNT Light phase	-3,054	-9,770 to 3,662	No	ns	0,9887			
5:Males CNT Light phase vs. 12:Females CRS Light phase	7,974	2,786 to 13,16	Yes	****	<0,0001			
5:Males CNT Light phase vs. 16:Males CNT Light phase	-4,518	-7,434 to -1,602	Yes	***	0,0002			
5:Males CNT Light phase vs. 16:Males CRS Light phase	3,177	-1,789 to 8,142	No	ns	0,6966			
5:Males CNT Light phase vs. 16:Females CNT Light phase	-3,552	-10,27 to 3,168	No	ns	0,9399			
5:Males CNT Light phase vs. 16:Females CRS Light phase	9,454	4,193 to 14,72	Yes	****	<0,0001			
5:Males CNT Light phase vs. 19:Males CNT Light phase	-6,141	-9,620 to -2,662	Yes	****	<0,0001			

5:Males CNT Light phase vs. 19:Males CRS Light phase	3,098	-2,001 to 8,197	No	ns	0,7882				
5:Males CNT Light phase vs. 19:Females CNT Light phase	-6,947	-14,13 to 0,2336	No	ns	0,0699				
5:Males CNT Light phase vs. 19:Females CRS Light phase	5,347	0,1809 to 10,51	Yes	*	0,0347				
5:Males CRS Light phase vs. 5:Females CNT Light phase	-1,753	-7,236 to 3,730	No	ns	>0,9999				
5:Males CRS Light phase vs. 5:Females CRS Light phase	1,738	-1,783 to 5,258	No	ns	0,9786				
5:Males CRS Light phase vs. 9:Males CNT Light phase	-3,946	-9,360 to 1,468	No	ns	0,4574				
5:Males CRS Light phase vs. 9:Males CRS Light phase	1,480	-0,3755 to 3,335	No	ns	0,3084				
5:Males CRS Light phase vs. 9:Females CNT Light phase	-4,380	-10,45 to 1,691	No	ns	0,4677				
5:Males CRS Light phase vs. 9:Females CRS Light phase	5,279	1,772 to 8,786	Yes	****	<0,0001				
5:Males CRS Light phase vs. 12:Males CNT Light phase	-5,680	-10,92 to -0,4365	Yes	*	0,0208				
5:Males CRS Light phase vs. 12:Males CRS Light phase	1,152	-0,8222 to 3,126	No	ns	0,8547				
5:Males CRS Light phase vs. 12:Females CNT Light phase	-6,126	-11,81 to -0,4385	Yes	*	0,0225				
5:Males CRS Light phase vs. 12:Females CRS Light phase	4,902	1,435 to 8,369	Yes	***	0,0002				
5:Males CRS Light phase vs. 16:Males CNT Light phase	-7,363	-12,89 to -1,840	Yes	**	0,0012				
5:Males CRS Light phase vs. 16:Males CRS Light phase	0,1046	-1,855 to 2,065	No	ns	>0,9999				
5:Males CRS Light phase vs. 16:Females CNT Light phase	-6,624	-12,32 to -0,9313	Yes	**	0,0087				
5:Males CRS Light phase vs. 16:Females CRS Light phase	6,382	2,788 to 9,976	Yes	****	<0,0001				
5:Males CRS Light phase vs. 19:Males CNT Light phase	-8,986	-14,33 to -3,641	Yes	****	<0,0001				
5:Males CRS Light phase vs. 19:Males CRS Light phase	-0,6550	-2,960 to 1,650	No	ns	>0,9999				
5:Males CRS Light phase vs. 19:Females CNT Light phase	-10,02	-16,28 to -3,759	Yes	****	<0,0001				
5:Males CRS Light phase vs. 19:Females CRS Light phase	2,275	-1,154 to 5,703	No	ns	0,7026				
5:Females CNT Light phase vs. 5:Females CRS Light phase	3,491	-2,140 to 9,121	No	ns	0,7588				
5:Females CNT Light phase vs. 9:Males CNT Light phase	-2,193	-9,004 to 4,618	No	ns	>0,9999				
5:Females CNT Light phase vs. 9:Males CRS Light phase	3,233	-2,087 to 8,553	No	ns	0,7599				
5:Females CNT Light phase vs. 9:Females CNT Light phase	-2,628	-4,615 to -0,6404	Yes	**	0,0021				
5:Females CNT Light phase vs. 9:Females CRS Light phase	7,032	1,409 to 12,66	Yes	**	0,0030				
5:Females CNT Light phase vs. 12:Males CNT Light phase	-3,927	-10,62 to 2,761	No	ns	0,8518				
5:Females CNT Light phase vs. 12:Males CRS Light phase	2,905	-2,437 to 8,246	No	ns	0,8921				
5:Females CNT Light phase vs. 12:Females CNT Light phase	-4,373	-6,761 to -1,984	Yes	****	<0,0001				
5:Females CNT Light phase vs. 12:Females CRS Light phase	6,655	1,052 to 12,26	Yes	**	0,0063				
5:Females CNT Light phase vs. 16:Males CNT Light phase	-5,610	-12,50 to 1,276	No	ns	0,2740				
5:Females CNT Light phase vs. 16:Males CRS Light phase	1,857	-3,545 to 7,260	No	ns	0,9996				
5:Females CNT Light phase vs. 16:Females CNT Light phase	-4,871	-7,503 to -2,240	Yes	****	<0,0001				
5:Females CNT Light phase vs. 16:Females CRS Light phase	8,135	2,466 to 13,80	Yes	***	0,0003				
5:Females CNT Light phase vs. 19:Males CNT Light phase	-7,233	-13,99 to -0,4756	Yes	*	0,0231				
5:Females CNT Light phase vs. 19:Males CRS Light phase	1,779	-3,744 to 7,302	No	ns	0,9999				
5:Females CNT Light phase vs. 19:Females CNT Light phase	-8,266	-10,62 to -5,912	Yes	****	<0,0001				
5:Females CNT Light phase vs. 19:Females CRS Light phase	4,028	-1,555 to 9,611	No	ns	0,4821				
5:Females CRS Light phase vs. 9:Males CNT Light phase	-5,684	-11,25 to -0,1193	Yes	*	0,0401				
5:Females CRS Light phase vs. 9:Males CRS Light phase	-0,2580	-3,473 to 2,957	No	ns	>0,9999				
5:Females CRS Light phase vs. 9:Females CNT Light phase	-6,118	-12,32 to 0,08217	No	ns	0,0571				
5:Females CRS Light phase vs. 9:Females CRS Light phase	3,541	2,255 to 4,828	Yes	****	<0,0001				
5:Females CRS Light phase vs. 12:Males CNT Light phase	-7,418	-12,82 to -2,018	Yes	***	0,0006				
5:Females CRS Light phase vs. 12:Males CRS Light phase	-0,5860	-3,843 to 2,671	No	ns	>0,9999				
5:Females CRS Light phase vs. 12:Females CNT Light phase	-7,863	-13,69 to -2,036	Yes	***	0,0009				
5:Females CRS Light phase vs. 12:Females CRS Light phase	3,164	1,341 to 4,987	Yes	****	<0,0001				
5:Females CRS Light phase vs. 16:Males CNT Light phase	-9,101	-14,77 to -3,432	Yes	****	<0,0001				
5:Females CRS Light phase vs. 16:Males CRS Light phase	-1,633	-5,006 to 1,740	No	ns	0,9820				
5:Females CRS Light phase vs. 16:Females CNT Light phase	-8,362	-14,20 to -2,529	Yes	***	0,0003				

5:Females CRS Light phase vs. 16:Females CRS Light phase	4,644	2,333 to 6,956	Yes	****	<0,0001			
5:Females CRS Light phase vs. 19:Males CNT Light phase	-10,72	-16,22 to -5,227	Yes	****	<0,0001			
5:Females CRS Light phase vs. 19:Males CRS Light phase	-1,711	-5,308 to 1,885	No	ns	0,9858			
5:Females CRS Light phase vs. 19:Females CNT Light phase	-11,76	-18,14 to -5,372	Yes	****	<0,0001			
5:Females CRS Light phase vs. 19:Females CRS Light phase	0,5370	-1,896 to 2,970	No	ns	>0,9999			
9:Males CNT Light phase vs. 9:Males CRS Light phase	5,426	0,1770 to 10,67	Yes	*	0,0358			
9:Males CNT Light phase vs. 9:Females CNT Light phase	-0,4345	-7,692 to 6,823	No	ns	>0,9999			
9:Males CNT Light phase vs. 9:Females CRS Light phase	9,225	3,668 to 14,78	Yes	****	<0,0001			
9:Males CNT Light phase vs. 12:Males CNT Light phase	-1,734	-3,608 to 0,1398	No	ns	0,0967			
9:Males CNT Light phase vs. 12:Males CRS Light phase	5,098	-0,1734 to 10,37	No	ns	0,0686			
9:Males CNT Light phase vs. 12:Females CNT Light phase	-2,180	-9,143 to 4,783	No	ns	>0,9999			
9:Males CNT Light phase vs. 12:Females CRS Light phase	8,848	3,312 to 14,38	Yes	****	<0,0001			
9:Males CNT Light phase vs. 16:Males CNT Light phase	-3,357	-5,798 to -0,9171	Yes	**	0,0013			
9:Males CNT Light phase vs. 16:Males CRS Light phase	4,050	-1,282 to 9,383	No	ns	0,3728			
9:Males CNT Light phase vs. 16:Females CNT Light phase	-2,678	-9,646 to 4,289	No	ns	0,9989			
9:Males CNT Light phase vs. 16:Females CRS Light phase	10,33	4,724 to 15,93	Yes	****	<0,0001			
9:Males CNT Light phase vs. 19:Males CNT Light phase	-4,980	-8,619 to -1,341	Yes	**	0,0014			
9:Males CNT Light phase vs. 19:Males CRS Light phase	3,972	-1,483 to 9,427	No	ns	0,4614			
9:Males CNT Light phase vs. 19:Females CNT Light phase	-6,073	-13,48 to 1,333	No	ns	0,2627			
9:Males CNT Light phase vs. 19:Females CRS Light phase	6,221	0,7045 to 11,74	Yes	*	0,0126			
9:Males CRS Light phase vs. 9:Females CNT Light phase	-5,860	-11,79 to 0,06740	No	ns	0,0557			
9:Males CRS Light phase vs. 9:Females CRS Light phase	3,799	0,5999 to 6,999	Yes	**	0,0052			
9:Males CRS Light phase vs. 12:Males CNT Light phase	-7,160	-12,23 to -2,089	Yes	***	0,0005			
9:Males CRS Light phase vs. 12:Males CRS Light phase	-0,3281	-1,483 to 0,8272	No	ns	>0,9999			
9:Males CRS Light phase vs. 12:Females CNT Light phase	-7,605	-13,14 to -2,074	Yes	***	0,0009			
9:Males CRS Light phase vs. 12:Females CRS Light phase	3,422	0,2673 to 6,577	Yes	*	0,0189			
9:Males CRS Light phase vs. 16:Males CNT Light phase	-8,843	-14,21 to -3,480	Yes	****	<0,0001			
9:Males CRS Light phase vs. 16:Males CRS Light phase	-1,375	-2,473 to -0,2773	Yes	**	0,0026			
9:Males CRS Light phase vs. 16:Females CNT Light phase	-8,104	-13,64 to -2,567	Yes	***	0,0003			
9:Males CRS Light phase vs. 16:Females CRS Light phase	4,902	1,605 to 8,200	Yes	****	<0,0001			
9:Males CRS Light phase vs. 19:Males CNT Light phase	-10,47	-15,64 to -5,288	Yes	****	<0,0001			
9:Males CRS Light phase vs. 19:Males CRS Light phase	-1,555	-3,489 to 0,3782	No	ns	0,2804			
9:Males CRS Light phase vs. 19:Females CNT Light phase	-11,50	-17,62 to -5,377	Yes	****	<0,0001			
9:Males CRS Light phase vs. 19:Females CRS Light phase	0,7950	-2,316 to 3,906	No	ns	>0,9999			
9:Females CNT Light phase vs. 9:Females CRS Light phase	9,660	3,466 to 15,85	Yes	****	<0,0001			
9:Females CNT Light phase vs. 12:Males CNT Light phase	-1,299	-8,445 to 5,846	No	ns	>0,9999			
9:Females CNT Light phase vs. 12:Males CRS Light phase	5,532	-0,4148 to 11,48	No	ns	0,0957			
9:Females CNT Light phase vs. 12:Females CNT Light phase	-1,745	-4,187 to 0,6967	No	ns	0,4440			
9:Females CNT Light phase vs. 12:Females CRS Light phase	9,282	3,106 to 15,46	Yes	***	0,0001			
9:Females CNT Light phase vs. 16:Males CNT Light phase	-2,982	-10,31 to 4,344	No	ns	0,9974			
9:Females CNT Light phase vs. 16:Males CRS Light phase	4,485	-1,516 to 10,49	No	ns	0,3953			
9:Females CNT Light phase vs. 16:Females CNT Light phase	-2,244	-4,716 to 0,2282	No	ns	0,1123			
9:Females CNT Light phase vs. 16:Females CRS Light phase	10,76	4,528 to 17,00	Yes	****	<0,0001			
9:Females CNT Light phase vs. 19:Males CNT Light phase	-4,605	-11,81 to 2,603	No	ns	0,7308			
9:Females CNT Light phase vs. 19:Males CRS Light phase	4,407	-1,699 to 10,51	No	ns	0,4696			
9:Females CNT Light phase vs. 19:Females CNT Light phase	-5,639	-8,225 to -3,053	Yes	****	<0,0001			
9:Females CNT Light phase vs. 19:Females CRS Light phase	6,655	0,4966 to 12,81	Yes	*	0,0217			
9:Females CRS Light phase vs. 12:Males CNT Light phase	-10,96	-16,35 to -5,567	Yes	****	<0,0001			
9:Females CRS Light phase vs. 12:Males CRS Light phase	-4,127	-7,370 to -0,8853	Yes	**	0,0016			

9:Females CRS Light phase vs. 12:Females CNT Light phase	-11,40	-17,23 to -5,584	Yes	****	<0,0001			
9:Females CRS Light phase vs. 12:Females CRS Light phase	-0,3774	-1,759 to 1,005	No	ns	>0,9999			
9:Females CRS Light phase vs. 16:Males CNT Light phase	-12,64	-18,30 to -6,981	Yes	****	<0,0001			
9:Females CRS Light phase vs. 16:Males CRS Light phase	-5,175	-8,533 to -1,816	Yes	****	<0,0001			
9:Females CRS Light phase vs. 16:Females CNT Light phase	-11,90	-17,73 to -6,077	Yes	****	<0,0001			
9:Females CRS Light phase vs. 16:Females CRS Light phase	1,103	-1,093 to 3,299	No	ns	0,9476			
9:Females CRS Light phase vs. 19:Males CNT Light phase	-14,26	-19,75 to -8,776	Yes	****	<0,0001			
9:Females CRS Light phase vs. 19:Males CRS Light phase	-5,253	-8,836 to -1,669	Yes	****	<0,0001			
9:Females CRS Light phase vs. 19:Females CNT Light phase	-15,30	-21,68 to -8,920	Yes	****	<0,0001			
9:Females CRS Light phase vs. 19:Females CRS Light phase	-3,004	-5,283 to -0,7262	Yes	**	0,0016			
12:Males CNT Light phase vs. 12:Males CRS Light phase	6,832	1,738 to 11,93	Yes	**	0,0012			
12:Males CNT Light phase vs. 12:Females CNT Light phase	-0,4459	-7,290 to 6,398	No	ns	>0,9999			
12:Males CNT Light phase vs. 12:Females CRS Light phase	10,58	5,211 to 15,95	Yes	****	<0,0001			
12:Males CNT Light phase vs. 16:Males CNT Light phase	-1,607	-3,214 to 0,0001085	No	ns	0,0500			
12:Males CNT Light phase vs. 16:Males CRS Light phase	5,784	0,6260 to 10,94	Yes	*	0,0140			
12:Males CNT Light phase vs. 16:Females CNT Light phase	-0,9445	-7,793 to 5,904	No	ns	>0,9999			
12:Males CNT Light phase vs. 16:Females CRS Light phase	12,06	6,621 to 17,50	Yes	****	<0,0001			
12:Males CNT Light phase vs. 19:Males CNT Light phase	-3,230	-6,447 to -0,01316	Yes	*	0,0482			
12:Males CNT Light phase vs. 19:Males CRS Light phase	5,706	0,4203 to 10,99	Yes	*	0,0216			
12:Males CNT Light phase vs. 19:Females CNT Light phase	-4,339	-11,64 to 2,958	No	ns	0,8339			
12:Males CNT Light phase vs. 19:Females CRS Light phase	7,955	2,605 to 13,30	Yes	***	0,0002			
12:Males CRS Light phase vs. 12:Females CNT Light phase	-7,277	-12,83 to -1,725	Yes	**	0,0018			
12:Males CRS Light phase vs. 12:Females CRS Light phase	3,750	0,5519 to 6,948	Yes	**	0,0062			
12:Males CRS Light phase vs. 16:Males CNT Light phase	-8,515	-13,90 to -3,130	Yes	****	<0,0001			
12:Males CRS Light phase vs. 16:Males CRS Light phase	-1,047	-2,091 to -0,003354	Yes	*	0,0483			
12:Males CRS Light phase vs. 16:Females CNT Light phase	-7,776	-13,33 to -2,218	Yes	***	0,0006			
12:Males CRS Light phase vs. 16:Females CRS Light phase	5,230	1,892 to 8,569	Yes	****	<0,0001			
12:Males CRS Light phase vs. 19:Males CNT Light phase	-10,14	-15,34 to -4,937	Yes	****	<0,0001			
12:Males CRS Light phase vs. 19:Males CRS Light phase	-1,181	-3,220 to 0,8576	No	ns	0,8431			
12:Males CRS Light phase vs. 19:Females CNT Light phase	-11,17	-17,31 to -5,030	Yes	****	<0,0001			
12:Males CRS Light phase vs. 19:Females CRS Light phase	1,123	-2,033 to 4,279	No	ns	0,9998			
12:Females CNT Light phase vs. 12:Females CRS Light phase	11,03	5,226 to 16,83	Yes	****	<0,0001			
12:Females CNT Light phase vs. 16:Males CNT Light phase	-1,237	-8,273 to 5,799	No	ns	>0,9999			
12:Females CNT Light phase vs. 16:Males CRS Light phase	6,230	0,6197 to 11,84	Yes	*	0,0160			
12:Females CNT Light phase vs. 16:Females CNT Light phase	-0,4987	-3,194 to 2,197	No	ns	>0,9999			
12:Females CNT Light phase vs. 16:Females CRS Light phase	12,51	6,643 to 18,37	Yes	****	<0,0001			
12:Females CNT Light phase vs. 19:Males CNT Light phase	-2,860	-9,771 to 4,051	No	ns	0,9968			
12:Females CNT Light phase vs. 19:Males CRS Light phase	6,152	0,4268 to 11,88	Yes	*	0,0231			
12:Females CNT Light phase vs. 19:Females CNT Light phase	-3,893	-6,564 to -1,223	Yes	***	0,0005			
12:Females CNT Light phase vs. 19:Females CRS Light phase	8,400	2,618 to 14,18	Yes	***	0,0003			
12:Females CRS Light phase vs. 16:Males CNT Light phase	-12,26	-17,91 to -6,624	Yes	****	<0,0001			
12:Females CRS Light phase vs. 16:Males CRS Light phase	-4,797	-8,113 to -1,481	Yes	***	0,0001			
12:Females CRS Light phase vs. 16:Females CNT Light phase	-11,53	-17,33 to -5,719	Yes	****	<0,0001			
12:Females CRS Light phase vs. 16:Females CRS Light phase	1,480	-0,7698 to 3,731	No	ns	0,6364			
12:Females CRS Light phase vs. 19:Males CNT Light phase	-13,89	-19,36 to -8,419	Yes	****	<0,0001			
12:Females CRS Light phase vs. 19:Males CRS Light phase	-4,876	-8,420 to -1,331	Yes	***	0,0003			
12:Females CRS Light phase vs. 19:Females CNT Light phase	-14,92	-21,28 to -8,560	Yes	****	<0,0001			
12:Females CRS Light phase vs. 19:Females CRS Light phase	-2,627	-4,683 to -0,5712	Yes	**	0,0025			
16:Males CNT Light phase vs. 16:Males CRS Light phase	7,467	2,023 to 12,91	Yes	***	0,0008			

16:Males CNT Light phase vs. 16:Females CNT Light phase	0,7385	-6,302 to 7,779	No	ns	>0,9999			
16:Males CNT Light phase vs. 16:Females CRS Light phase	13,75	8,039 to 19,45	Yes	****	<0,0001			
16:Males CNT Light phase vs. 19:Males CNT Light phase	-1,623	-4,383 to 1,137	No	ns	0,7647			
16:Males CNT Light phase vs. 19:Males CRS Light phase	7,389	1,827 to 12,95	Yes	**	0,0013			
16:Males CNT Light phase vs. 19:Females CNT Light phase	-2,656	-10,13 to 4,817	No	ns	0,9997			
16:Males CNT Light phase vs. 19:Females CRS Light phase	9,638	4,016 to 15,26	Yes	****	<0,0001			
16:Males CRS Light phase vs. 16:Females CNT Light phase	-6,729	-12,35 to -1,112	Yes	**	0,0061			
16:Males CRS Light phase vs. 16:Females CRS Light phase	6,278	2,827 to 9,728	Yes	****	<0,0001			
16:Males CRS Light phase vs. 19:Males CNT Light phase	-9,090	-14,35 to -3,828	Yes	****	<0,0001			
16:Males CRS Light phase vs. 19:Males CRS Light phase	-0,3655	-2,403 to 1,672	No	ns	>0,9999			
16:Males CRS Light phase vs. 19:Females CNT Light phase	-10,12	-16,32 to -3,932	Yes	****	<0,0001			
16:Males CRS Light phase vs. 19:Females CRS Light phase	2,170	-1,106 to 5,446	No	ns	0,7002			
16:Females CNT Light phase vs. 16:Females CRS Light phase	13,01	7,136 to 18,88	Yes	****	<0,0001			
16:Females CNT Light phase vs. 19:Males CNT Light phase	-2,361	-9,277 to 4,554	No	ns	0,9998			
16:Females CNT Light phase vs. 19:Males CRS Light phase	6,651	0,9196 to 12,38	Yes	**	0,0090			
16:Females CNT Light phase vs. 19:Females CNT Light phase	-3,395	-5,684 to -1,105	Yes	***	0,0004			
16:Females CNT Light phase vs. 19:Females CRS Light phase	8,899	3,111 to 14,69	Yes	****	<0,0001			
16:Females CRS Light phase vs. 19:Males CNT Light phase	-15,37	-20,90 to -9,832	Yes	****	<0,0001			
16:Females CRS Light phase vs. 19:Males CRS Light phase	-6,356	-10,02 to -2,688	Yes	****	<0,0001			
16:Females CRS Light phase vs. 19:Females CNT Light phase	-16,40	-22,82 to -9,984	Yes	****	<0,0001			
16:Females CRS Light phase vs. 19:Females CRS Light phase	-4,107	-6,709 to -1,505	Yes	****	<0,0001			
19:Males CNT Light phase vs. 19:Males CRS Light phase	9,012	3,626 to 14,40	Yes	****	<0,0001			
19:Males CNT Light phase vs. 19:Females CNT Light phase	-1,033	-8,391 to 6,325	No	ns	>0,9999			
19:Males CNT Light phase vs. 19:Females CRS Light phase	11,26	5,812 to 16,71	Yes	****	<0,0001			
19:Males CRS Light phase vs. 19:Females CNT Light phase	-10,05	-16,34 to -3,752	Yes	****	<0,0001			
19:Males CRS Light phase vs. 19:Females CRS Light phase	2,249	-1,259 to 5,756	No	ns	0,7571			
19:Females CNT Light phase vs. 19:Females CRS Light phase	12,29	5,950 to 18,64	Yes	****	<0,0001			
Test details	Mean 1	Mean 2	Mean Diff,	SE of diff,	N1	N2	q	DF
-1:Males CNT Light phase vs. -1:Males CRS Light phase	100,0	98,37	1,627	1,303	28	50	1,766	36,08
-1:Males CNT Light phase vs. -1:Females CNT Light phase	100,0	100,0	0,0003521	1,699	28	28	0,0002931	54,00
-1:Males CNT Light phase vs. -1:Females CRS Light phase	100,0	100,4	-0,3635	1,444	28	37	0,3560	48,53
-1:Males CNT Light phase vs. 2:Males CNT Light phase	100,0	101,9	-1,917	0,4459	28	28	6,079	27,00
-1:Males CNT Light phase vs. 2:Males CRS Light phase	100,0	100,8	-0,8023	1,322	28	52	0,8581	38,05
-1:Males CNT Light phase vs. 2:Females CNT Light phase	100,0	102,8	-2,838	1,658	28	28	2,421	53,80
-1:Males CNT Light phase vs. 2:Females CRS Light phase	100,0	102,1	-2,134	1,453	28	37	2,077	49,24
-1:Males CNT Light phase vs. 5:Males CNT Light phase	100,0	104,7	-4,694	0,9378	28	28	7,079	27,00
-1:Males CNT Light phase vs. 5:Males CRS Light phase	100,0	101,6	-1,622	1,346	28	52	1,705	40,45
-1:Males CNT Light phase vs. 5:Females CNT Light phase	100,0	103,4	-3,375	1,729	28	28	2,760	53,97
-1:Males CNT Light phase vs. 5:Females CRS Light phase	100,0	99,88	0,1154	1,392	28	37	0,1173	44,13
-1:Males CNT Light phase vs. 9:Males CNT Light phase	100,0	105,6	-5,568	1,106	28	28	7,123	27,00
-1:Males CNT Light phase vs. 9:Males CRS Light phase	100,0	100,1	-0,1425	1,289	28	52	0,1563	34,77
-1:Males CNT Light phase vs. 9:Females CNT Light phase	100,0	106,0	-6,003	1,842	28	28	4,608	52,95
-1:Males CNT Light phase vs. 9:Females CRS Light phase	100,0	96,34	3,657	1,390	28	37	3,721	43,93
-1:Males CNT Light phase vs. 12:Males CNT Light phase	100,0	107,3	-7,302	1,025	28	28	10,08	27,00
-1:Males CNT Light phase vs. 12:Males CRS Light phase	100,0	100,5	-0,4706	1,297	28	52	0,5130	35,55
-1:Males CNT Light phase vs. 12:Females CNT Light phase	100,0	107,7	-7,748	1,768	28	28	6,197	53,75
-1:Males CNT Light phase vs. 12:Females CRS Light phase	100,0	96,72	3,279	1,383	28	37	3,353	43,35
-1:Males CNT Light phase vs. 16:Males CNT Light phase	99,71	109,0	-9,275	1,027	27	27	12,78	26,00
-1:Males CNT Light phase vs. 16:Males CRS Light phase	100,0	101,5	-1,518	1,319	28	52	1,628	37,69

-1:Males CNT Light phase vs. 16:Females CNT Light phase	100,0	108,2	-8,247	1,769	28	28	6,592	53,75
-1:Males CNT Light phase vs. 16:Females CRS Light phase	100,0	95,24	4,760	1,404	28	37	4,794	45,19
-1:Males CNT Light phase vs. 19:Males CNT Light phase	99,71	110,6	-10,90	0,9151	27	27	16,84	26,00
-1:Males CNT Light phase vs. 19:Males CRS Light phase	100,0	101,6	-1,596	1,358	28	42	1,662	41,34
-1:Males CNT Light phase vs. 19:Females CNT Light phase	100,0	111,6	-11,64	1,879	28	28	8,760	52,40
-1:Males CNT Light phase vs. 19:Females CRS Light phase	100,0	99,35	0,6524	1,377	28	37	0,6701	42,79
-1:Males CRS Light phase vs. -1:Females CNT Light phase	98,37	100,0	-1,627	1,292	50	28	1,780	36,25
-1:Males CRS Light phase vs. -1:Females CRS Light phase	98,37	100,4	-1,990	0,9317	50	37	3,021	62,08
-1:Males CRS Light phase vs. 2:Males CNT Light phase	98,37	101,9	-3,544	1,269	50	28	3,949	36,65
-1:Males CRS Light phase vs. 2:Males CRS Light phase	98,37	101,0	-2,611	0,3564	50	50	10,36	49,00
-1:Males CRS Light phase vs. 2:Females CNT Light phase	98,37	102,8	-4,465	1,237	50	28	5,105	37,26
-1:Males CRS Light phase vs. 2:Females CRS Light phase	98,37	102,1	-3,761	0,9455	50	37	5,626	61,19
-1:Males CRS Light phase vs. 5:Males CNT Light phase	98,37	104,7	-6,321	1,226	50	28	7,291	37,48
-1:Males CRS Light phase vs. 5:Males CRS Light phase	98,37	101,6	-3,217	0,6508	50	50	6,991	49,00
-1:Males CRS Light phase vs. 5:Females CNT Light phase	98,37	103,4	-5,002	1,332	50	28	5,313	35,63
-1:Males CRS Light phase vs. 5:Females CRS Light phase	98,37	99,88	-1,512	0,8485	50	37	2,519	68,40
-1:Males CRS Light phase vs. 9:Males CNT Light phase	98,37	105,6	-7,195	1,315	50	28	7,740	35,89
-1:Males CRS Light phase vs. 9:Males CRS Light phase	98,37	100,1	-1,678	0,5283	50	50	4,493	49,00
-1:Males CRS Light phase vs. 9:Females CNT Light phase	98,37	106,0	-7,630	1,475	50	28	7,314	33,82
-1:Males CRS Light phase vs. 9:Females CRS Light phase	98,37	96,34	2,030	0,8448	50	37	3,398	68,72
-1:Males CRS Light phase vs. 12:Males CNT Light phase	98,37	107,3	-8,929	1,273	50	28	9,922	36,59
-1:Males CRS Light phase vs. 12:Males CRS Light phase	98,37	100,3	-1,958	0,5362	50	50	5,164	49,00
-1:Males CRS Light phase vs. 12:Females CNT Light phase	98,37	107,7	-9,375	1,381	50	28	9,597	34,92
-1:Males CRS Light phase vs. 12:Females CRS Light phase	98,37	96,72	1,653	0,8341	50	37	2,802	69,67
-1:Males CRS Light phase vs. 16:Males CNT Light phase	98,37	109,0	-10,61	1,338	50	27	11,22	34,23
-1:Males CRS Light phase vs. 16:Males CRS Light phase	98,37	101,4	-2,986	0,5638	50	50	7,489	49,00
-1:Males CRS Light phase vs. 16:Females CNT Light phase	98,37	108,2	-9,874	1,383	50	28	10,10	34,90
-1:Males CRS Light phase vs. 16:Females CRS Light phase	98,37	95,24	3,133	0,8683	50	37	5,103	66,75
-1:Males CRS Light phase vs. 19:Males CNT Light phase	98,37	110,6	-12,24	1,294	50	27	13,37	34,90
-1:Males CRS Light phase vs. 19:Males CRS Light phase	98,19	101,3	-3,072	0,5330	40	40	8,151	39,00
-1:Males CRS Light phase vs. 19:Females CNT Light phase	98,37	111,6	-13,27	1,521	50	28	12,33	33,36
-1:Males CRS Light phase vs. 19:Females CRS Light phase	98,37	99,35	-0,9745	0,8237	50	37	1,673	70,62
-1:Females CNT Light phase vs. -1:Females CRS Light phase	100,0	100,4	-0,3639	1,435	28	37	0,3587	48,86
-1:Females CNT Light phase vs. 2:Males CNT Light phase	100,0	101,9	-1,917	1,674	28	28	1,620	53,98
-1:Females CNT Light phase vs. 2:Males CRS Light phase	100,0	100,8	-0,8027	1,312	28	52	0,8653	38,26
-1:Females CNT Light phase vs. 2:Females CNT Light phase	100,0	102,8	-2,839	0,6138	28	28	6,540	27,00
-1:Females CNT Light phase vs. 2:Females CRS Light phase	100,0	102,1	-2,135	1,444	28	37	2,091	49,57
-1:Females CNT Light phase vs. 5:Males CNT Light phase	100,0	104,7	-4,695	1,641	28	28	4,045	53,79
-1:Females CNT Light phase vs. 5:Males CRS Light phase	100,0	101,6	-1,623	1,336	28	52	1,718	40,71
-1:Females CNT Light phase vs. 5:Females CNT Light phase	100,0	103,4	-3,376	0,5878	28	28	8,121	27,00
-1:Females CNT Light phase vs. 5:Females CRS Light phase	100,0	99,88	0,1151	1,382	28	37	0,1178	44,42
-1:Females CNT Light phase vs. 9:Males CNT Light phase	100,0	105,6	-5,569	1,708	28	28	4,610	53,98
-1:Females CNT Light phase vs. 9:Males CRS Light phase	100,0	100,1	-0,1429	1,279	28	52	0,1580	34,92
-1:Females CNT Light phase vs. 9:Females CNT Light phase	100,0	106,0	-6,003	0,7670	28	28	11,07	27,00
-1:Females CNT Light phase vs. 9:Females CRS Light phase	100,0	96,34	3,656	1,380	28	37	3,748	44,22
-1:Females CNT Light phase vs. 12:Males CNT Light phase	100,0	107,3	-7,302	1,676	28	28	6,161	53,98
-1:Females CNT Light phase vs. 12:Males CRS Light phase	100,0	100,5	-0,4710	1,287	28	52	0,5177	35,71
-1:Females CNT Light phase vs. 12:Females CNT Light phase	100,0	107,7	-7,748	0,6228	28	28	17,59	27,00
-1:Females CNT Light phase vs. 12:Females CRS Light phase	100,0	96,72	3,279	1,373	28	37	3,377	43,63

-1:Females CNT Light phase vs. 16:Males CNT Light phase	100,0	109,0	-8,986	1,726	28	27	7,361	52,82
-1:Females CNT Light phase vs. 16:Males CRS Light phase	100,0	101,5	-1,518	1,308	28	52	1,641	37,89
-1:Females CNT Light phase vs. 16:Females CNT Light phase	100,0	108,2	-8,247	0,8510	28	28	13,71	27,00
-1:Females CNT Light phase vs. 16:Females CRS Light phase	100,0	95,24	4,760	1,394	28	37	4,828	45,49
-1:Females CNT Light phase vs. 19:Males CNT Light phase	100,0	110,6	-10,61	1,693	28	27	8,863	52,98
-1:Females CNT Light phase vs. 19:Males CRS Light phase	100,0	101,6	-1,596	1,348	28	42	1,675	41,60
-1:Females CNT Light phase vs. 19:Females CNT Light phase	100,0	111,6	-11,64	0,7166	28	28	22,98	27,00
-1:Females CNT Light phase vs. 19:Females CRS Light phase	100,0	99,35	0,6521	1,367	28	37	0,6746	43,07
-1:Females CRS Light phase vs. 2:Males CNT Light phase	100,4	101,9	-1,553	1,414	37	28	1,554	49,60
-1:Females CRS Light phase vs. 2:Males CRS Light phase	100,4	100,8	-0,4388	0,9587	37	52	0,6473	66,93
-1:Females CRS Light phase vs. 2:Females CNT Light phase	100,4	102,8	-2,475	1,385	37	28	2,527	50,68
-1:Females CRS Light phase vs. 2:Females CRS Light phase	100,4	102,1	-1,771	0,7004	37	37	3,575	36,00
-1:Females CRS Light phase vs. 5:Males CNT Light phase	100,4	104,7	-4,331	1,375	37	28	4,453	51,05
-1:Females CRS Light phase vs. 5:Males CRS Light phase	100,4	101,6	-1,259	0,9911	37	52	1,796	71,90
-1:Females CRS Light phase vs. 5:Females CNT Light phase	100,4	103,4	-3,012	1,470	37	28	2,897	47,66
-1:Females CRS Light phase vs. 5:Females CRS Light phase	100,4	99,88	0,4790	0,8210	37	37	0,8250	36,00
-1:Females CRS Light phase vs. 9:Males CNT Light phase	100,4	105,6	-5,205	1,455	37	28	5,059	48,16
-1:Females CRS Light phase vs. 9:Males CRS Light phase	100,4	100,1	0,2210	0,9129	37	52	0,3424	58,91
-1:Females CRS Light phase vs. 9:Females CNT Light phase	100,4	106,0	-5,639	1,601	37	28	4,980	43,89
-1:Females CRS Light phase vs. 9:Females CRS Light phase	100,4	96,34	4,020	0,7003	37	37	8,119	36,00
-1:Females CRS Light phase vs. 12:Males CNT Light phase	100,4	107,3	-6,939	1,417	37	28	6,924	49,49
-1:Females CRS Light phase vs. 12:Males CRS Light phase	100,4	100,5	-0,1071	0,9239	37	52	0,1639	60,93
-1:Females CRS Light phase vs. 12:Females CNT Light phase	100,4	107,7	-7,384	1,516	37	28	6,891	46,24
-1:Females CRS Light phase vs. 12:Females CRS Light phase	100,4	96,72	3,643	0,6630	37	37	7,771	36,00
-1:Females CRS Light phase vs. 16:Males CNT Light phase	100,4	109,0	-8,622	1,476	37	27	8,262	45,90
-1:Females CRS Light phase vs. 16:Males CRS Light phase	100,4	101,5	-1,154	0,9537	37	52	1,712	66,11
-1:Females CRS Light phase vs. 16:Females CNT Light phase	100,4	108,2	-7,883	1,517	37	28	7,350	46,20
-1:Females CRS Light phase vs. 16:Females CRS Light phase	100,4	95,24	5,123	0,9614	37	37	7,536	36,00
-1:Females CRS Light phase vs. 19:Males CNT Light phase	100,4	110,6	-10,24	1,437	37	27	10,09	47,21
-1:Females CRS Light phase vs. 19:Males CRS Light phase	100,4	101,6	-1,233	1,008	37	42	1,730	70,54
-1:Females CRS Light phase vs. 19:Females CNT Light phase	100,4	111,6	-11,28	1,644	37	28	9,701	42,88
-1:Females CRS Light phase vs. 19:Females CRS Light phase	100,4	99,35	1,016	0,6982	37	37	2,058	36,00
2:Males CNT Light phase vs. 2:Males CRS Light phase	101,9	100,8	1,114	1,289	28	52	1,223	38,75
2:Males CNT Light phase vs. 2:Females CNT Light phase	101,9	102,8	-0,9214	1,631	28	28	0,7987	53,95
2:Males CNT Light phase vs. 2:Females CRS Light phase	101,9	102,1	-0,2174	1,423	28	37	0,2161	50,32
2:Males CNT Light phase vs. 5:Males CNT Light phase	101,9	104,7	-2,778	0,8775	28	28	4,476	27,00
2:Males CNT Light phase vs. 5:Males CRS Light phase	101,9	101,6	0,2945	1,313	28	52	0,3170	41,29
2:Males CNT Light phase vs. 5:Females CNT Light phase	101,9	103,4	-1,458	1,704	28	28	1,210	53,83
2:Males CNT Light phase vs. 5:Females CRS Light phase	101,9	99,88	2,032	1,361	28	37	2,112	45,08
2:Males CNT Light phase vs. 9:Males CNT Light phase	101,9	105,6	-3,651	1,078	28	28	4,788	27,00
2:Males CNT Light phase vs. 9:Males CRS Light phase	101,9	100,1	1,774	1,255	28	52	1,999	35,27
2:Males CNT Light phase vs. 9:Females CNT Light phase	101,9	106,0	-4,086	1,819	28	28	3,177	52,47
2:Males CNT Light phase vs. 9:Females CRS Light phase	101,9	96,34	5,574	1,358	28	37	5,803	44,87
2:Males CNT Light phase vs. 12:Males CNT Light phase	101,9	107,3	-5,385	1,004	28	28	7,583	27,00
2:Males CNT Light phase vs. 12:Males CRS Light phase	101,9	100,5	1,446	1,264	28	52	1,619	36,09
2:Males CNT Light phase vs. 12:Females CNT Light phase	101,9	107,7	-5,831	1,743	28	28	4,730	53,49
2:Males CNT Light phase vs. 12:Females CRS Light phase	101,9	96,72	5,196	1,352	28	37	5,437	44,27
2:Males CNT Light phase vs. 16:Males CNT Light phase	101,6	109,0	-7,367	1,073	27	27	9,713	26,00
2:Males CNT Light phase vs. 16:Males CRS Light phase	101,9	101,5	0,3991	1,285	28	52	0,4390	38,36

2:Males CNT Light phase vs. 16:Females CNT Light phase	101,9	108,2	-6,330	1,745	28	28	5,131	53,47
2:Males CNT Light phase vs. 16:Females CRS Light phase	101,9	95,24	6,677	1,373	28	37	6,877	46,18
2:Males CNT Light phase vs. 19:Males CNT Light phase	101,6	110,6	-8,990	1,012	27	27	12,57	26,00
2:Males CNT Light phase vs. 19:Males CRS Light phase	101,9	101,6	0,3207	1,326	28	42	0,3420	42,20
2:Males CNT Light phase vs. 19:Females CNT Light phase	101,9	111,6	-9,725	1,856	28	28	7,409	51,84
2:Males CNT Light phase vs. 19:Females CRS Light phase	101,9	99,35	2,569	1,345	28	37	2,701	43,69
2:Males CRS Light phase vs. 2:Females CNT Light phase	100,8	102,8	-2,036	1,258	52	28	2,289	39,48
2:Males CRS Light phase vs. 2:Females CRS Light phase	100,8	102,1	-1,332	0,9722	52	37	1,938	65,93
2:Males CRS Light phase vs. 5:Males CNT Light phase	100,8	104,7	-3,892	1,247	52	28	4,414	39,74
2:Males CRS Light phase vs. 5:Males CRS Light phase	100,8	101,6	-0,8200	0,5714	52	52	2,030	51,00
2:Males CRS Light phase vs. 5:Females CNT Light phase	100,8	103,4	-2,573	1,351	52	28	2,694	37,50
2:Males CRS Light phase vs. 5:Females CRS Light phase	100,8	99,88	0,9178	0,8781	52	37	1,478	73,73
2:Males CRS Light phase vs. 9:Males CNT Light phase	100,8	105,6	-4,766	1,334	52	28	5,053	37,82
2:Males CRS Light phase vs. 9:Males CRS Light phase	100,8	100,1	0,6598	0,5517	52	52	1,691	51,00
2:Males CRS Light phase vs. 9:Females CNT Light phase	100,8	106,0	-5,200	1,492	52	28	4,928	35,30
2:Males CRS Light phase vs. 9:Females CRS Light phase	100,8	96,34	4,459	0,8745	52	37	7,211	74,06
2:Males CRS Light phase vs. 12:Males CNT Light phase	100,8	107,3	-6,500	1,293	52	28	7,111	38,67
2:Males CRS Light phase vs. 12:Males CRS Light phase	100,8	100,5	0,3317	0,5977	52	52	0,7850	51,00
2:Males CRS Light phase vs. 12:Females CNT Light phase	100,8	107,7	-6,946	1,400	52	28	7,017	36,64
2:Males CRS Light phase vs. 12:Females CRS Light phase	100,8	96,72	4,082	0,8641	52	37	6,680	75,03
2:Males CRS Light phase vs. 16:Males CNT Light phase	100,8	109,0	-8,183	1,357	52	27	8,529	36,03
2:Males CRS Light phase vs. 16:Males CRS Light phase	100,8	101,5	-0,7154	0,6418	52	52	1,576	51,00
2:Males CRS Light phase vs. 16:Females CNT Light phase	100,8	108,2	-7,444	1,401	52	28	7,513	36,62
2:Males CRS Light phase vs. 16:Females CRS Light phase	100,8	95,24	5,562	0,8972	52	37	8,768	71,99
2:Males CRS Light phase vs. 19:Males CNT Light phase	100,8	110,6	-9,806	1,314	52	27	10,55	36,83
2:Males CRS Light phase vs. 19:Males CRS Light phase	100,2	101,6	-1,356	0,6409	42	42	2,993	41,00
2:Males CRS Light phase vs. 19:Females CNT Light phase	100,8	111,6	-10,84	1,538	52	28	9,966	34,74
2:Males CRS Light phase vs. 19:Females CRS Light phase	100,8	99,35	1,455	0,8541	52	37	2,409	75,99
2:Females CNT Light phase vs. 2:Females CRS Light phase	102,8	102,1	0,7039	1,395	28	37	0,7139	51,41
2:Females CNT Light phase vs. 5:Males CNT Light phase	102,8	104,7	-1,856	1,598	28	28	1,642	53,99
2:Females CNT Light phase vs. 5:Males CRS Light phase	102,8	101,6	1,216	1,282	28	52	1,341	42,17
2:Females CNT Light phase vs. 5:Females CNT Light phase	102,8	103,4	-0,5370	0,4097	28	28	1,854	27,00
2:Females CNT Light phase vs. 5:Females CRS Light phase	102,8	99,88	2,954	1,331	28	37	3,139	46,05
2:Females CNT Light phase vs. 9:Males CNT Light phase	102,8	105,6	-2,730	1,667	28	28	2,316	53,73
2:Females CNT Light phase vs. 9:Males CRS Light phase	102,8	100,1	2,696	1,223	28	52	3,117	35,79
2:Females CNT Light phase vs. 9:Females CNT Light phase	102,8	106,0	-3,165	0,5609	28	28	7,979	27,00
2:Females CNT Light phase vs. 9:Females CRS Light phase	102,8	96,34	6,495	1,328	28	37	6,915	45,84
2:Females CNT Light phase vs. 12:Males CNT Light phase	102,8	107,3	-4,464	1,634	28	28	3,863	53,94
2:Females CNT Light phase vs. 12:Males CRS Light phase	102,8	100,5	2,368	1,231	28	52	2,719	36,66
2:Females CNT Light phase vs. 12:Females CNT Light phase	102,8	107,7	-4,910	0,6291	28	28	11,04	27,00
2:Females CNT Light phase vs. 12:Females CRS Light phase	102,8	96,72	6,118	1,321	28	37	6,547	45,21
2:Females CNT Light phase vs. 16:Males CNT Light phase	102,8	109,0	-6,147	1,685	28	27	5,158	52,36
2:Females CNT Light phase vs. 16:Males CRS Light phase	102,8	101,5	1,320	1,254	28	52	1,489	39,07
2:Females CNT Light phase vs. 16:Females CNT Light phase	102,8	108,2	-5,409	0,7052	28	28	10,85	27,00
2:Females CNT Light phase vs. 16:Females CRS Light phase	102,8	95,24	7,598	1,343	28	37	7,999	47,18
2:Females CNT Light phase vs. 19:Males CNT Light phase	102,8	110,6	-7,770	1,651	28	27	6,655	52,73
2:Females CNT Light phase vs. 19:Males CRS Light phase	102,8	101,6	1,242	1,295	28	42	1,356	43,09
2:Females CNT Light phase vs. 19:Females CNT Light phase	102,8	111,6	-8,803	0,7681	28	28	16,21	27,00
2:Females CNT Light phase vs. 19:Females CRS Light phase	102,8	99,35	3,491	1,315	28	37	3,754	44,61

2:Females CRS Light phase vs. 5:Males CNT Light phase	102,1	104,7	-2,560	1,385	37	28	2,614	51,78
2:Females CRS Light phase vs. 5:Males CRS Light phase	102,1	101,6	0,5119	1,004	37	52	0,7209	70,87
2:Females CRS Light phase vs. 5:Females CNT Light phase	102,1	103,4	-1,241	1,479	37	28	1,187	48,36
2:Females CRS Light phase vs. 5:Females CRS Light phase	102,1	99,88	2,250	0,5958	37	37	5,340	36,00
2:Females CRS Light phase vs. 9:Males CNT Light phase	102,1	105,6	-3,434	1,464	37	28	3,318	48,87
2:Females CRS Light phase vs. 9:Males CRS Light phase	102,1	100,1	1,992	0,9270	37	52	3,039	58,08
2:Females CRS Light phase vs. 9:Females CNT Light phase	102,1	106,0	-3,868	1,610	37	28	3,399	44,51
2:Females CRS Light phase vs. 9:Females CRS Light phase	102,1	96,34	5,791	0,5494	37	37	14,91	36,00
2:Females CRS Light phase vs. 12:Males CNT Light phase	102,1	107,3	-5,168	1,426	37	28	5,124	50,21
2:Females CRS Light phase vs. 12:Males CRS Light phase	102,1	100,5	1,664	0,9379	37	52	2,509	60,05
2:Females CRS Light phase vs. 12:Females CNT Light phase	102,1	107,7	-5,614	1,524	37	28	5,209	46,91
2:Females CRS Light phase vs. 12:Females CRS Light phase	102,1	96,72	5,414	0,6086	37	37	12,58	36,00
2:Females CRS Light phase vs. 16:Males CNT Light phase	102,1	109,0	-6,851	1,485	37	27	6,526	46,59
2:Females CRS Light phase vs. 16:Males CRS Light phase	102,1	101,5	0,6165	0,9672	37	52	0,9014	65,12
2:Females CRS Light phase vs. 16:Females CNT Light phase	102,1	108,2	-6,112	1,525	37	28	5,667	46,87
2:Females CRS Light phase vs. 16:Females CRS Light phase	102,1	95,24	6,894	0,9268	37	37	10,52	36,00
2:Females CRS Light phase vs. 19:Males CNT Light phase	102,1	110,6	-8,474	1,446	37	27	8,290	47,91
2:Females CRS Light phase vs. 19:Males CRS Light phase	102,1	101,6	0,5382	1,021	37	42	0,7457	69,78
2:Females CRS Light phase vs. 19:Females CNT Light phase	102,1	111,6	-9,507	1,652	37	28	8,139	43,47
2:Females CRS Light phase vs. 19:Females CRS Light phase	102,1	99,35	2,787	0,7511	37	37	5,247	36,00
5:Males CNT Light phase vs. 5:Males CRS Light phase	104,7	101,6	3,072	1,272	28	52	3,416	42,49
5:Males CNT Light phase vs. 5:Females CNT Light phase	104,7	103,4	1,319	1,672	28	28	1,115	53,50
5:Males CNT Light phase vs. 5:Females CRS Light phase	104,7	99,88	4,810	1,321	28	37	5,151	46,39
5:Males CNT Light phase vs. 9:Males CNT Light phase	104,7	105,6	-0,8739	0,4356	28	28	2,837	27,00
5:Males CNT Light phase vs. 9:Males CRS Light phase	104,7	100,1	4,552	1,212	28	52	5,311	35,97
5:Males CNT Light phase vs. 9:Females CNT Light phase	104,7	106,0	-1,308	1,789	28	28	1,034	51,71
5:Males CNT Light phase vs. 9:Females CRS Light phase	104,7	96,34	8,351	1,318	28	37	8,960	46,18
5:Males CNT Light phase vs. 12:Males CNT Light phase	104,7	107,3	-2,608	0,6630	28	28	5,563	27,00
5:Males CNT Light phase vs. 12:Males CRS Light phase	104,7	100,5	4,224	1,220	28	52	4,895	36,87
5:Males CNT Light phase vs. 12:Females CNT Light phase	104,7	107,7	-3,054	1,712	28	28	2,522	52,99
5:Males CNT Light phase vs. 12:Females CRS Light phase	104,7	96,72	7,974	1,311	28	37	8,599	45,55
5:Males CNT Light phase vs. 16:Males CNT Light phase	104,5	109,0	-4,518	0,7044	27	27	9,071	26,00
5:Males CNT Light phase vs. 16:Males CRS Light phase	104,7	101,5	3,177	1,243	28	52	3,614	39,33
5:Males CNT Light phase vs. 16:Females CNT Light phase	104,7	108,2	-3,552	1,714	28	28	2,932	52,97
5:Males CNT Light phase vs. 16:Females CRS Light phase	104,7	95,24	9,454	1,333	28	37	10,03	47,54
5:Males CNT Light phase vs. 19:Males CNT Light phase	104,5	110,6	-6,141	0,8404	27	27	10,33	26,00
5:Males CNT Light phase vs. 19:Males CRS Light phase	104,7	101,6	3,098	1,285	28	42	3,410	43,41
5:Males CNT Light phase vs. 19:Females CNT Light phase	104,7	111,6	-6,947	1,827	28	28	5,377	50,99
5:Males CNT Light phase vs. 19:Females CRS Light phase	104,7	99,35	5,347	1,305	28	37	5,795	44,94
5:Males CRS Light phase vs. 5:Females CNT Light phase	101,6	103,4	-1,753	1,374	52	28	1,804	39,79
5:Males CRS Light phase vs. 5:Females CRS Light phase	101,6	99,88	1,738	0,9134	52	37	2,690	78,53
5:Males CRS Light phase vs. 9:Males CNT Light phase	101,6	105,6	-3,946	1,357	52	28	4,111	40,17
5:Males CRS Light phase vs. 9:Males CRS Light phase	101,6	100,1	1,480	0,4721	52	52	4,433	51,00
5:Males CRS Light phase vs. 9:Females CNT Light phase	101,6	106,0	-4,380	1,514	52	28	4,093	37,12
5:Males CRS Light phase vs. 9:Females CRS Light phase	101,6	96,34	5,279	0,9100	52	37	8,204	78,83
5:Males CRS Light phase vs. 12:Males CNT Light phase	101,6	107,3	-5,680	1,317	52	28	6,100	41,20
5:Males CRS Light phase vs. 12:Males CRS Light phase	101,6	100,5	1,152	0,5023	52	52	3,243	51,00
5:Males CRS Light phase vs. 12:Females CNT Light phase	101,6	107,7	-6,126	1,422	52	28	6,091	38,75
5:Males CRS Light phase vs. 12:Females CRS Light phase	101,6	96,72	4,902	0,9000	52	37	7,702	79,71

5:Males CRS Light phase vs. 16:Males CNT Light phase	101,6	109,0	-7,363	1,380	52	27	7,546	38,22
5:Males CRS Light phase vs. 16:Males CRS Light phase	101,6	101,5	0,1046	0,4987	52	52	0,2966	51,00
5:Males CRS Light phase vs. 16:Females CNT Light phase	101,6	108,2	-6,624	1,424	52	28	6,580	38,72
5:Males CRS Light phase vs. 16:Females CRS Light phase	101,6	95,24	6,382	0,9318	52	37	9,687	76,92
5:Males CRS Light phase vs. 19:Males CNT Light phase	101,6	110,6	-8,986	1,338	52	27	9,499	39,19
5:Males CRS Light phase vs. 19:Males CRS Light phase	100,9	101,6	-0,6550	0,5788	42	42	1,600	41,00
5:Males CRS Light phase vs. 19:Females CNT Light phase	101,6	111,6	-10,02	1,558	52	28	9,092	36,44
5:Males CRS Light phase vs. 19:Females CRS Light phase	101,6	99,35	2,275	0,8904	52	37	3,613	80,54
5:Females CNT Light phase vs. 5:Females CRS Light phase	103,4	99,88	3,491	1,419	28	37	3,479	43,37
5:Females CNT Light phase vs. 9:Males CNT Light phase	103,4	105,6	-2,193	1,738	28	28	1,784	53,99
5:Females CNT Light phase vs. 9:Males CRS Light phase	103,4	100,1	3,233	1,318	28	52	3,468	34,38
5:Females CNT Light phase vs. 9:Females CNT Light phase	103,4	106,0	-2,628	0,4819	28	28	7,712	27,00
5:Females CNT Light phase vs. 9:Females CRS Light phase	103,4	96,34	7,032	1,417	28	37	7,020	43,18
5:Females CNT Light phase vs. 12:Males CNT Light phase	103,4	107,3	-3,927	1,707	28	28	3,254	53,85
5:Females CNT Light phase vs. 12:Males CRS Light phase	103,4	100,5	2,905	1,326	28	52	3,098	35,12
5:Females CNT Light phase vs. 12:Females CNT Light phase	103,4	107,7	-4,373	0,5792	28	28	10,68	27,00
5:Females CNT Light phase vs. 12:Females CRS Light phase	103,4	96,72	6,655	1,410	28	37	6,673	42,62
5:Females CNT Light phase vs. 16:Males CNT Light phase	103,4	109,0	-5,610	1,756	28	27	4,518	52,97
5:Females CNT Light phase vs. 16:Males CRS Light phase	103,4	101,5	1,857	1,347	28	52	1,950	37,16
5:Females CNT Light phase vs. 16:Females CNT Light phase	103,4	108,2	-4,871	0,6382	28	28	10,79	27,00
5:Females CNT Light phase vs. 16:Females CRS Light phase	103,4	95,24	8,135	1,431	28	37	8,041	44,40
5:Females CNT Light phase vs. 19:Males CNT Light phase	103,4	110,6	-7,233	1,723	28	27	5,937	52,99
5:Females CNT Light phase vs. 19:Males CRS Light phase	103,4	101,6	1,779	1,386	28	42	1,815	40,66
5:Females CNT Light phase vs. 19:Females CNT Light phase	103,4	111,6	-8,266	0,5710	28	28	20,47	27,00
5:Females CNT Light phase vs. 19:Females CRS Light phase	103,4	99,35	4,028	1,404	28	37	4,056	42,08
5:Females CRS Light phase vs. 9:Males CNT Light phase	99,88	105,6	-5,684	1,403	37	28	5,728	43,81
5:Females CRS Light phase vs. 9:Males CRS Light phase	99,88	100,1	-0,2580	0,8278	37	52	0,4407	64,96
5:Females CRS Light phase vs. 9:Females CNT Light phase	99,88	106,0	-6,118	1,555	37	28	5,566	40,20
5:Females CRS Light phase vs. 9:Females CRS Light phase	99,88	96,34	3,541	0,3200	37	37	15,65	36,00
5:Females CRS Light phase vs. 12:Males CNT Light phase	99,88	107,3	-7,418	1,364	37	28	7,691	44,98
5:Females CRS Light phase vs. 12:Males CRS Light phase	99,88	100,5	-0,5860	0,8400	37	52	0,9866	67,27
5:Females CRS Light phase vs. 12:Females CNT Light phase	99,88	107,7	-7,863	1,466	37	28	7,586	42,16
5:Females CRS Light phase vs. 12:Females CRS Light phase	99,88	96,72	3,164	0,4534	37	37	9,869	36,00
5:Females CRS Light phase vs. 16:Males CNT Light phase	99,88	109,0	-9,101	1,425	37	27	9,033	41,72
5:Females CRS Light phase vs. 16:Males CRS Light phase	99,88	101,5	-1,633	0,8727	37	52	2,647	72,88
5:Females CRS Light phase vs. 16:Females CNT Light phase	99,88	108,2	-8,362	1,467	37	28	8,060	42,12
5:Females CRS Light phase vs. 16:Females CRS Light phase	99,88	95,24	4,644	0,5749	37	37	11,43	36,00
5:Females CRS Light phase vs. 19:Males CNT Light phase	99,88	110,6	-10,72	1,384	37	27	10,96	42,84
5:Females CRS Light phase vs. 19:Males CRS Light phase	99,88	101,6	-1,711	0,9315	37	42	2,598	74,78
5:Females CRS Light phase vs. 19:Females CNT Light phase	99,88	111,6	-11,76	1,598	37	28	10,40	39,36
5:Females CRS Light phase vs. 19:Females CRS Light phase	99,88	99,35	0,5370	0,6052	37	37	1,255	36,00
9:Males CNT Light phase vs. 9:Males CRS Light phase	105,6	100,1	5,426	1,301	28	52	5,896	34,61
9:Males CNT Light phase vs. 9:Females CNT Light phase	105,6	106,0	-0,4345	1,851	28	28	0,3320	53,09
9:Males CNT Light phase vs. 9:Females CRS Light phase	105,6	96,34	9,225	1,401	28	37	9,313	43,61
9:Males CNT Light phase vs. 12:Males CNT Light phase	105,6	107,3	-1,734	0,4544	28	28	5,397	27,00
9:Males CNT Light phase vs. 12:Males CRS Light phase	105,6	100,5	5,098	1,309	28	52	5,506	35,37
9:Males CNT Light phase vs. 12:Females CNT Light phase	105,6	107,7	-2,180	1,777	28	28	1,735	53,82
9:Males CNT Light phase vs. 12:Females CRS Light phase	105,6	96,72	8,848	1,394	28	37	8,973	43,04
9:Males CNT Light phase vs. 16:Males CNT Light phase	105,6	109,0	-3,357	0,5895	27	27	8,055	26,00

9:Males CNT Light phase vs. 16:Males CRS Light phase	105,6	101,5	4,050	1,330	28	52	4,306	37,46
9:Males CNT Light phase vs. 16:Females CNT Light phase	105,6	108,2	-2,678	1,778	28	28	2,130	53,82
9:Males CNT Light phase vs. 16:Females CRS Light phase	105,6	95,24	10,33	1,415	28	37	10,32	44,86
9:Males CNT Light phase vs. 19:Males CNT Light phase	105,6	110,6	-4,980	0,8791	27	27	8,012	26,00
9:Males CNT Light phase vs. 19:Males CRS Light phase	105,6	101,6	3,972	1,370	28	42	4,101	41,06
9:Males CNT Light phase vs. 19:Females CNT Light phase	105,6	111,6	-6,073	1,888	28	28	4,550	52,58
9:Males CNT Light phase vs. 19:Females CRS Light phase	105,6	99,35	6,221	1,388	28	37	6,337	42,49
9:Males CRS Light phase vs. 9:Females CNT Light phase	100,1	106,0	-5,860	1,463	52	28	5,663	32,83
9:Males CRS Light phase vs. 9:Females CRS Light phase	100,1	96,34	3,799	0,8240	52	37	6,521	65,27
9:Males CRS Light phase vs. 12:Males CNT Light phase	100,1	107,3	-7,160	1,259	52	28	8,042	35,21
9:Males CRS Light phase vs. 12:Males CRS Light phase	100,1	100,5	-0,3281	0,2940	52	52	1,578	51,00
9:Males CRS Light phase vs. 12:Females CNT Light phase	100,1	107,7	-7,605	1,369	52	28	7,857	33,78
9:Males CRS Light phase vs. 12:Females CRS Light phase	100,1	96,72	3,422	0,8130	52	37	5,953	66,22
9:Males CRS Light phase vs. 16:Males CNT Light phase	100,1	109,0	-8,843	1,325	52	27	9,440	33,04
9:Males CRS Light phase vs. 16:Males CRS Light phase	100,1	101,5	-1,375	0,2793	52	52	6,962	51,00
9:Males CRS Light phase vs. 16:Females CNT Light phase	100,1	108,2	-8,104	1,370	52	28	8,364	33,76
9:Males CRS Light phase vs. 16:Females CRS Light phase	100,1	95,24	4,902	0,8481	52	37	8,175	63,34
9:Males CRS Light phase vs. 19:Males CNT Light phase	100,1	110,6	-10,47	1,281	52	27	11,56	33,61
9:Males CRS Light phase vs. 19:Males CRS Light phase	100,0	101,6	-1,555	0,4854	42	42	4,531	41,00
9:Males CRS Light phase vs. 19:Females CNT Light phase	100,1	111,6	-11,50	1,510	52	28	10,77	32,43
9:Males CRS Light phase vs. 19:Females CRS Light phase	100,1	99,35	0,7950	0,8023	52	37	1,401	67,17
9:Females CNT Light phase vs. 9:Females CRS Light phase	106,0	96,34	9,660	1,553	28	37	8,799	40,03
9:Females CNT Light phase vs. 12:Males CNT Light phase	106,0	107,3	-1,299	1,821	28	28	1,009	52,52
9:Females CNT Light phase vs. 12:Males CRS Light phase	106,0	100,5	5,532	1,470	28	52	5,321	33,41
9:Females CNT Light phase vs. 12:Females CNT Light phase	106,0	107,7	-1,745	0,5922	28	28	4,168	27,00
9:Females CNT Light phase vs. 12:Females CRS Light phase	106,0	96,72	9,282	1,547	28	37	8,487	39,56
9:Females CNT Light phase vs. 16:Males CNT Light phase	106,0	109,0	-2,982	1,867	28	27	2,259	52,55
9:Females CNT Light phase vs. 16:Males CRS Light phase	106,0	101,5	4,485	1,489	28	52	4,259	35,02
9:Females CNT Light phase vs. 16:Females CNT Light phase	106,0	108,2	-2,244	0,5995	28	28	5,294	27,00
9:Females CNT Light phase vs. 16:Females CRS Light phase	106,0	95,24	10,76	1,565	28	37	9,723	41,07
9:Females CNT Light phase vs. 19:Males CNT Light phase	106,0	110,6	-4,605	1,836	28	27	3,547	52,12
9:Females CNT Light phase vs. 19:Males CRS Light phase	106,0	101,6	4,407	1,524	28	42	4,088	37,89
9:Females CNT Light phase vs. 19:Females CNT Light phase	106,0	111,6	-5,639	0,6271	28	28	12,72	27,00
9:Females CNT Light phase vs. 19:Females CRS Light phase	106,0	99,35	6,655	1,541	28	37	6,107	39,11
9:Females CRS Light phase vs. 12:Males CNT Light phase	96,34	107,3	-10,96	1,362	37	28	11,38	44,77
9:Females CRS Light phase vs. 12:Males CRS Light phase	96,34	100,5	-4,127	0,8363	37	52	6,980	67,60
9:Females CRS Light phase vs. 12:Females CNT Light phase	96,34	107,7	-11,40	1,464	37	28	11,02	41,97
9:Females CRS Light phase vs. 12:Females CRS Light phase	96,34	96,72	-0,3774	0,3437	37	37	1,553	36,00
9:Females CRS Light phase vs. 16:Males CNT Light phase	96,34	109,0	-12,64	1,423	37	27	12,57	41,53
9:Females CRS Light phase vs. 16:Males CRS Light phase	96,34	101,5	-5,175	0,8691	37	52	8,421	73,22
9:Females CRS Light phase vs. 16:Females CNT Light phase	96,34	108,2	-11,90	1,465	37	28	11,49	41,94
9:Females CRS Light phase vs. 16:Females CRS Light phase	96,34	95,24	1,103	0,5462	37	37	2,856	36,00
9:Females CRS Light phase vs. 19:Males CNT Light phase	96,34	110,6	-14,26	1,382	37	27	14,60	42,64
9:Females CRS Light phase vs. 19:Males CRS Light phase	96,34	101,6	-5,253	0,9281	37	42	8,004	74,95
9:Females CRS Light phase vs. 19:Females CNT Light phase	96,34	111,6	-15,30	1,596	37	28	13,55	39,21
9:Females CRS Light phase vs. 19:Females CRS Light phase	96,34	99,35	-3,004	0,5666	37	37	7,499	36,00
12:Males CNT Light phase vs. 12:Males CRS Light phase	107,3	100,5	6,832	1,267	28	52	7,625	36,03
12:Males CNT Light phase vs. 12:Females CNT Light phase	107,3	107,7	-0,4459	1,746	28	28	0,3611	53,52
12:Males CNT Light phase vs. 12:Females CRS Light phase	107,3	96,72	10,58	1,355	28	37	11,04	44,17

12:Males CNT Light phase vs. 16:Males CNT Light phase	107,4	109,0	-1,607	0,3882	27	27	5,854	26,00
12:Males CNT Light phase vs. 16:Males CRS Light phase	107,3	101,5	5,784	1,289	28	52	6,347	38,29
12:Males CNT Light phase vs. 16:Females CNT Light phase	107,3	108,2	-0,9445	1,747	28	28	0,7645	53,51
12:Males CNT Light phase vs. 16:Females CRS Light phase	107,3	95,24	12,06	1,376	28	37	12,39	46,07
12:Males CNT Light phase vs. 19:Males CNT Light phase	107,4	110,6	-3,230	0,7771	27	27	5,878	26,00
12:Males CNT Light phase vs. 19:Males CRS Light phase	107,3	101,6	5,706	1,329	28	42	6,070	42,11
12:Males CNT Light phase vs. 19:Females CNT Light phase	107,3	111,6	-4,339	1,859	28	28	3,302	51,90
12:Males CNT Light phase vs. 19:Females CRS Light phase	107,3	99,35	7,955	1,349	28	37	8,342	43,59
12:Males CRS Light phase vs. 12:Females CNT Light phase	100,5	107,7	-7,277	1,376	52	28	7,478	34,46
12:Males CRS Light phase vs. 12:Females CRS Light phase	100,5	96,72	3,750	0,8254	52	37	6,425	68,57
12:Males CRS Light phase vs. 16:Males CNT Light phase	100,5	109,0	-8,515	1,332	52	27	9,037	33,75
12:Males CRS Light phase vs. 16:Males CRS Light phase	100,5	101,5	-1,047	0,2656	52	52	5,576	51,00
12:Males CRS Light phase vs. 16:Females CNT Light phase	100,5	108,2	-7,776	1,378	52	28	7,982	34,44
12:Males CRS Light phase vs. 16:Females CRS Light phase	100,5	95,24	5,230	0,8600	52	37	8,602	65,59
12:Males CRS Light phase vs. 19:Males CNT Light phase	100,5	110,6	-10,14	1,289	52	27	11,12	34,37
12:Males CRS Light phase vs. 19:Males CRS Light phase	100,4	101,6	-1,181	0,5119	42	42	3,263	41,00
12:Males CRS Light phase vs. 19:Females CNT Light phase	100,5	111,6	-11,17	1,517	52	28	10,42	32,98
12:Males CRS Light phase vs. 19:Females CRS Light phase	100,5	99,35	1,123	0,8149	52	37	1,949	69,55
12:Females CNT Light phase vs. 12:Females CRS Light phase	107,7	96,72	11,03	1,458	28	37	10,70	41,45
12:Females CNT Light phase vs. 16:Males CNT Light phase	107,7	109,0	-1,237	1,794	28	27	0,9753	52,98
12:Females CNT Light phase vs. 16:Males CRS Light phase	107,7	101,5	6,230	1,396	28	52	6,310	36,33
12:Females CNT Light phase vs. 16:Females CNT Light phase	107,7	108,2	-0,4987	0,6536	28	28	1,079	27,00
12:Females CNT Light phase vs. 16:Females CRS Light phase	107,7	95,24	12,51	1,477	28	37	11,97	43,13
12:Females CNT Light phase vs. 19:Males CNT Light phase	107,7	110,6	-2,860	1,762	28	27	2,296	52,83
12:Females CNT Light phase vs. 19:Males CRS Light phase	107,7	101,6	6,152	1,434	28	42	6,067	39,59
12:Females CNT Light phase vs. 19:Females CNT Light phase	107,7	111,6	-3,893	0,6476	28	28	8,503	27,00
12:Females CNT Light phase vs. 19:Females CRS Light phase	107,7	99,35	8,400	1,452	28	37	8,184	40,94
12:Females CRS Light phase vs. 16:Males CNT Light phase	96,72	109,0	-12,26	1,416	37	27	12,25	40,98
12:Females CRS Light phase vs. 16:Males CRS Light phase	96,72	101,5	-4,797	0,8586	37	52	7,901	74,20
12:Females CRS Light phase vs. 16:Females CNT Light phase	96,72	108,2	-11,53	1,459	37	28	11,17	41,42
12:Females CRS Light phase vs. 16:Females CRS Light phase	96,72	95,24	1,480	0,5597	37	37	3,741	36,00
12:Females CRS Light phase vs. 19:Males CNT Light phase	96,72	110,6	-13,89	1,375	37	27	14,28	42,07
12:Females CRS Light phase vs. 19:Males CRS Light phase	96,72	101,6	-4,876	0,9184	37	42	7,508	75,39
12:Females CRS Light phase vs. 19:Females CNT Light phase	96,72	111,6	-14,92	1,591	37	28	13,26	38,77
12:Females CRS Light phase vs. 19:Females CRS Light phase	96,72	99,35	-2,627	0,5113	37	37	7,266	36,00
16:Males CNT Light phase vs. 16:Males CRS Light phase	109,0	101,5	7,467	1,353	27	52	7,804	35,70
16:Males CNT Light phase vs. 16:Females CNT Light phase	109,0	108,2	0,7385	1,795	27	28	0,5818	52,98
16:Males CNT Light phase vs. 16:Females CRS Light phase	109,0	95,24	13,75	1,437	27	37	13,53	42,71
16:Males CNT Light phase vs. 19:Males CNT Light phase	109,0	110,6	-1,623	0,6667	27	27	3,443	26,00
16:Males CNT Light phase vs. 19:Males CRS Light phase	109,0	101,6	7,389	1,392	27	42	7,508	39,08
16:Males CNT Light phase vs. 19:Females CNT Light phase	109,0	111,6	-2,656	1,904	27	28	1,973	52,17
16:Males CNT Light phase vs. 19:Females CRS Light phase	109,0	99,35	9,638	1,410	27	37	9,665	40,46
16:Males CRS Light phase vs. 16:Females CNT Light phase	101,5	108,2	-6,729	1,398	52	28	6,808	36,30
16:Males CRS Light phase vs. 16:Females CRS Light phase	101,5	95,24	6,278	0,8919	52	37	9,954	71,14
16:Males CRS Light phase vs. 19:Males CNT Light phase	101,5	110,6	-9,090	1,310	52	27	9,811	36,48
16:Males CRS Light phase vs. 19:Males CRS Light phase	101,2	101,6	-0,3655	0,5115	42	42	1,010	41,00
16:Males CRS Light phase vs. 19:Females CNT Light phase	101,5	111,6	-10,12	1,535	52	28	9,327	34,48
16:Males CRS Light phase vs. 19:Females CRS Light phase	101,5	99,35	2,170	0,8485	52	37	3,617	75,16
16:Females CNT Light phase vs. 16:Females CRS Light phase	108,2	95,24	13,01	1,479	28	37	12,44	43,09

16:Females CNT Light phase vs. 19:Males CNT Light phase	108,2	110,6	-2,361	1,763	28	27	1,894	52,83
16:Females CNT Light phase vs. 19:Males CRS Light phase	108,2	101,6	6,651	1,435	28	42	6,553	39,56
16:Females CNT Light phase vs. 19:Females CNT Light phase	108,2	111,6	-3,395	0,5552	28	28	8,647	27,00
16:Females CNT Light phase vs. 19:Females CRS Light phase	108,2	99,35	8,899	1,453	28	37	8,661	40,91
16:Females CRS Light phase vs. 19:Males CNT Light phase	95,24	110,6	-15,37	1,396	37	27	15,57	43,89
16:Females CRS Light phase vs. 19:Males CRS Light phase	95,24	101,6	-6,356	0,9495	37	42	9,466	73,87
16:Females CRS Light phase vs. 19:Females CNT Light phase	95,24	111,6	-16,40	1,609	37	28	14,42	40,19
16:Females CRS Light phase vs. 19:Females CRS Light phase	95,24	99,35	-4,107	0,6472	37	37	8,976	36,00
19:Males CNT Light phase vs. 19:Males CRS Light phase	110,6	101,6	9,012	1,350	27	42	9,439	40,08
19:Males CNT Light phase vs. 19:Females CNT Light phase	110,6	111,6	-1,033	1,874	27	28	0,7799	51,62
19:Males CNT Light phase vs. 19:Females CRS Light phase	110,6	99,35	11,26	1,369	27	37	11,63	41,52
19:Males CRS Light phase vs. 19:Females CNT Light phase	101,6	111,6	-10,05	1,569	42	28	9,054	37,17
19:Males CRS Light phase vs. 19:Females CRS Light phase	101,6	99,35	2,249	0,9089	42	37	3,498	75,78
19:Females CNT Light phase vs. 19:Females CRS Light phase	111,6	99,35	12,29	1,585	28	37	10,97	38,34

Statistics of Figure 1b

Table Analyzed	% Weight gain – Dark phase				
Mixed-effects model (REML)	Matching by factor: time				
Assume sphericity?	No				
Alpha	0,05				
Fixed effects (type III)	P value	P value summary	Statistically significant (P < 0,05)?	F (DFn, DFd)	Geisser-Greenhouse's epsilon
time	<0,0001	****	Yes	F (3,529, 345,2) = 55,60	0,5881
sex	0,8375	ns	No	F (1, 98) = 0,04230	
stress	<0,0001	****	Yes	F (1, 98) = 47,35	
time x sex	0,0536	ns	No	F (6, 587) = 2,082	
time x stress	<0,0001	****	Yes	F (6, 587) = 27,39	
sex x stress	0,9259	ns	No	F (1, 98) = 0,008685	
time x sex x stress	0,0030	**	Yes	F (6, 587) = 3,348	
Random effects	SD	Variance			
Subject	4,006	16,05			
Residual	2,991	8,949			
Was the matching effective?					
Chi-square, df	449,0, 1				
P value	<0,0001				
P value summary	****				
Is there significant matching (P < 0.05)?	Yes				
Data summary					

Compare each cell mean with every other cell mean								
Number of families	1							
Number of comparisons per family	378							
Alpha	0,05							
Tukey's multiple comparisons test	Mean Diff,	95,00% CI of diff,	Below threshold?	Summary	Adjusted P Value			
-1:Males CNT Dark phase vs. -1:Males CRS Dark phase	-0,1613	-6,322 to 5,999	No	ns	>0,9999			
-1:Males CNT Dark phase vs. -1:Females CNT Dark phase	-1,542	-7,034 to 3,949	No	ns	>0,9999			
-1:Males CNT Dark phase vs. -1:Females CRS Dark phase	-0,2317	-5,820 to 5,356	No	ns	>0,9999			
-1:Males CNT Dark phase vs. 2:Males CNT Dark phase	-0,6888	-3,435 to 2,057	No	ns	>0,9999			
-1:Males CNT Dark phase vs. 2:Males CRS Dark phase	2,736	-3,162 to 8,634	No	ns	0,9850			
-1:Males CNT Dark phase vs. 2:Females CNT Dark phase	-1,046	-6,555 to 4,463	No	ns	>0,9999			
-1:Males CNT Dark phase vs. 2:Females CRS Dark phase	2,741	-2,821 to 8,304	No	ns	0,9677			
-1:Males CNT Dark phase vs. 5:Males CNT Dark phase	-2,050	-4,861 to 0,7612	No	ns	0,3980			
-1:Males CNT Dark phase vs. 5:Males CRS Dark phase	2,562	-3,253 to 8,376	No	ns	0,9920			
-1:Males CNT Dark phase vs. 5:Females CNT Dark phase	-0,9904	-6,685 to 4,705	No	ns	>0,9999			
-1:Males CNT Dark phase vs. 5:Females CRS Dark phase	4,081	-1,635 to 9,797	No	ns	0,5206			
-1:Males CNT Dark phase vs. 9:Males CNT Dark phase	-4,167	-8,064 to -0,2691	Yes	*	0,0266			
-1:Males CNT Dark phase vs. 9:Males CRS Dark phase	2,605	-3,786 to 8,997	No	ns	0,9975			
-1:Males CNT Dark phase vs. 9:Females CNT Dark phase	-2,531	-8,203 to 3,141	No	ns	0,9862			
-1:Males CNT Dark phase vs. 9:Females CRS Dark phase	3,607	-1,605 to 8,819	No	ns	0,5557			
-1:Males CNT Dark phase vs. 12:Males CNT Dark phase	-7,493	-11,71 to -3,271	Yes	****	<0,0001			
-1:Males CNT Dark phase vs. 12:Males CRS Dark phase	4,585	-0,9478 to 10,12	No	ns	0,2398			

-1:Males CNT Dark phase vs. 12:Females CNT Dark phase	-4,924	-10,53 to 0,6850	No	ns	0,1544			
-1:Males CNT Dark phase vs. 12:Females CRS Dark phase	2,660	-2,774 to 8,093	No	ns	0,9683			
-1:Males CNT Dark phase vs. 16:Males CNT Dark phase	-7,846	-12,84 to -2,854	Yes	***	0,0002			
-1:Males CNT Dark phase vs. 16:Males CRS Dark phase	0,9016	-5,032 to 6,835	No	ns	>0,9999			
-1:Males CNT Dark phase vs. 16:Females CNT Dark phase	-8,353	-14,59 to -2,116	Yes	**	0,0012			
-1:Males CNT Dark phase vs. 16:Females CRS Dark phase	1,892	-3,508 to 7,292	No	ns	0,9997			
-1:Males CNT Dark phase vs. 19:Males CNT Dark phase	-7,796	-14,67 to -0,9197	Yes	*	0,0144			
-1:Males CNT Dark phase vs. 19:Males CRS Dark phase	-1,952	-7,901 to 3,997	No	ns	>0,9999			
-1:Males CNT Dark phase vs. 19:Females CNT Dark phase	-10,03	-17,25 to -2,810	Yes	***	0,0009			
-1:Males CNT Dark phase vs. 19:Females CRS Dark phase	-1,653	-7,004 to 3,698	No	ns	>0,9999			
-1:Males CRS Dark phase vs. -1:Females CNT Dark phase	-1,381	-6,633 to 3,870	No	ns	>0,9999			
-1:Males CRS Dark phase vs. -1:Females CRS Dark phase	-0,07042	-5,423 to 5,282	No	ns	>0,9999			
-1:Males CRS Dark phase vs. 2:Males CNT Dark phase	-0,5275	-6,814 to 5,759	No	ns	>0,9999			
-1:Males CRS Dark phase vs. 2:Males CRS Dark phase	2,898	-0,3282 to 6,123	No	ns	0,1233			
-1:Males CRS Dark phase vs. 2:Females CNT Dark phase	-0,8848	-6,155 to 4,385	No	ns	>0,9999			
-1:Males CRS Dark phase vs. 2:Females CRS Dark phase	2,903	-2,423 to 8,228	No	ns	0,9222			
-1:Males CRS Dark phase vs. 5:Males CNT Dark phase	-1,888	-7,715 to 3,939	No	ns	>0,9999			
-1:Males CRS Dark phase vs. 5:Males CRS Dark phase	2,723	0,6631 to 4,783	Yes	**	0,0019			
-1:Males CRS Dark phase vs. 5:Females CNT Dark phase	-0,8291	-6,300 to 4,642	No	ns	>0,9999			
-1:Males CRS Dark phase vs. 5:Females CRS Dark phase	4,242	-1,248 to 9,732	No	ns	0,3749			
-1:Males CRS Dark phase vs. 9:Males CNT Dark phase	-4,005	-9,925 to 1,915	No	ns	0,6326			
-1:Males CRS Dark phase vs. 9:Males CRS Dark phase	2,767	0,5541 to 4,979	Yes	**	0,0041			
-1:Males CRS Dark phase vs. 9:Females CNT Dark phase	-2,370	-7,816 to 3,076	No	ns	0,9909			
-1:Males CRS Dark phase vs. 9:Females CRS Dark phase	3,768	-1,176 to 8,713	No	ns	0,3870			
-1:Males CRS Dark phase vs. 12:Males CNT Dark phase	-7,332	-13,35 to -1,318	Yes	**	0,0038			
-1:Males CRS Dark phase vs. 12:Males CRS Dark phase	4,747	1,728 to 7,765	Yes	***	0,0001			
-1:Males CRS Dark phase vs. 12:Females CNT Dark phase	-4,763	-10,14 to 0,6154	No	ns	0,1470			
-1:Males CRS Dark phase vs. 12:Females CRS Dark phase	2,821	-2,365 to 8,007	No	ns	0,9213			
-1:Males CRS Dark phase vs. 16:Males CNT Dark phase	-7,685	-13,37 to -2,000	Yes	***	0,0007			
-1:Males CRS Dark phase vs. 16:Males CRS Dark phase	1,063	-1,036 to 3,162	No	ns	0,9280			
-1:Males CRS Dark phase vs. 16:Females CNT Dark phase	-8,191	-14,24 to -2,144	Yes	***	0,0010			
-1:Males CRS Dark phase vs. 16:Females CRS Dark phase	2,053	-3,096 to 7,203	No	ns	0,9981			
-1:Males CRS Dark phase vs. 19:Males CNT Dark phase	-7,634	-14,43 to -0,8429	Yes	*	0,0126			
-1:Males CRS Dark phase vs. 19:Males CRS Dark phase	-1,791	-3,948 to 0,3659	No	ns	0,2115			
-1:Males CRS Dark phase vs. 19:Females CNT Dark phase	-9,870	-16,95 to -2,792	Yes	***	0,0009			
-1:Males CRS Dark phase vs. 19:Females CRS Dark phase	-1,492	-6,588 to 3,605	No	ns	>0,9999			
-1:Females CNT Dark phase vs. -1:Females CRS Dark phase	1,311	-3,217 to 5,838	No	ns	>0,9999			
-1:Females CNT Dark phase vs. 2:Males CNT Dark phase	0,8536	-4,782 to 6,490	No	ns	>0,9999			
-1:Females CNT Dark phase vs. 2:Males CRS Dark phase	4,279	-0,6469 to 9,204	No	ns	0,1683			
-1:Females CNT Dark phase vs. 2:Females CNT Dark phase	0,5531	-1,069 to 2,175	No	ns	0,9901			
-1:Females CNT Dark phase vs. 2:Females CRS Dark phase	4,284	-0,2113 to 8,779	No	ns	0,0787			
-1:Females CNT Dark phase vs. 5:Males CNT Dark phase	-0,5072	-5,610 to 4,596	No	ns	>0,9999			
-1:Females CNT Dark phase vs. 5:Males CRS Dark phase	4,104	-0,7161 to 8,924	No	ns	0,1933			
-1:Females CNT Dark phase vs. 5:Females CNT Dark phase	0,6131	-2,463 to 3,689	No	ns	>0,9999			
-1:Females CNT Dark phase vs. 5:Females CRS Dark phase	5,623	0,9316 to 10,31	Yes	**	0,0058			
-1:Females CNT Dark phase vs. 9:Males CNT Dark phase	-2,624	-7,836 to 2,588	No	ns	0,9479			
-1:Females CNT Dark phase vs. 9:Males CRS Dark phase	4,148	-1,385 to 9,680	No	ns	0,4054			
-1:Females CNT Dark phase vs. 9:Females CNT Dark phase	-1,171	-4,229 to 1,887	No	ns	0,9694			
-1:Females CNT Dark phase vs. 9:Females CRS Dark phase	5,149	1,111 to 9,188	Yes	**	0,0031			

-1:Females CNT Dark phase vs. 12:Males CNT Dark phase	-5,951	-11,27 to -0,6288	Yes	*	0,0147			
-1:Females CNT Dark phase vs. 12:Males CRS Dark phase	6,128	1,668 to 10,59	Yes	***	0,0008			
-1:Females CNT Dark phase vs. 12:Females CNT Dark phase	-3,458	-7,872 to 0,9569	No	ns	0,2073			
-1:Females CNT Dark phase vs. 12:Females CRS Dark phase	4,202	-0,1256 to 8,530	No	ns	0,0661			
-1:Females CNT Dark phase vs. 16:Males CNT Dark phase	-6,303	-11,24 to -1,369	Yes	**	0,0025			
-1:Females CNT Dark phase vs. 16:Males CRS Dark phase	2,444	-2,526 to 7,414	No	ns	0,9620			
-1:Females CNT Dark phase vs. 16:Females CNT Dark phase	-6,777	-12,47 to -1,088	Yes	*	0,0138			
-1:Females CNT Dark phase vs. 16:Females CRS Dark phase	3,434	-0,8493 to 7,718	No	ns	0,2731			
-1:Females CNT Dark phase vs. 19:Males CNT Dark phase	-6,253	-12,46 to -0,04449	Yes	*	0,0466			
-1:Females CNT Dark phase vs. 19:Males CRS Dark phase	-0,4098	-5,399 to 4,579	No	ns	>0,9999			
-1:Females CNT Dark phase vs. 19:Females CNT Dark phase	-8,792	-14,61 to -2,971	Yes	**	0,0018			
-1:Females CNT Dark phase vs. 19:Females CRS Dark phase	-0,1104	-4,330 to 4,109	No	ns	>0,9999			
-1:Females CRS Dark phase vs. 2:Males CNT Dark phase	-0,4571	-6,189 to 5,275	No	ns	>0,9999			
-1:Females CRS Dark phase vs. 2:Males CRS Dark phase	2,968	-2,058 to 7,994	No	ns	0,8511			
-1:Females CRS Dark phase vs. 2:Females CNT Dark phase	-0,8144	-5,362 to 3,734	No	ns	>0,9999			
-1:Females CRS Dark phase vs. 2:Females CRS Dark phase	2,973	-0,2249 to 6,171	No	ns	0,0952			
-1:Females CRS Dark phase vs. 5:Males CNT Dark phase	-1,818	-7,018 to 3,382	No	ns	0,9998			
-1:Females CRS Dark phase vs. 5:Males CRS Dark phase	2,793	-2,127 to 7,713	No	ns	0,8925			
-1:Females CRS Dark phase vs. 5:Females CNT Dark phase	-0,7587	-5,558 to 4,040	No	ns	>0,9999			
-1:Females CRS Dark phase vs. 5:Females CRS Dark phase	4,312	-0,1421 to 8,767	No	ns	0,0673			
-1:Females CRS Dark phase vs. 9:Males CNT Dark phase	-3,935	-9,244 to 1,375	No	ns	0,4467			
-1:Females CRS Dark phase vs. 9:Males CRS Dark phase	2,837	-2,796 to 8,469	No	ns	0,9635			
-1:Females CRS Dark phase vs. 9:Females CNT Dark phase	-2,299	-7,068 to 2,469	No	ns	0,9658			
-1:Females CRS Dark phase vs. 9:Females CRS Dark phase	3,839	0,9796 to 6,698	Yes	**	0,0014			
-1:Females CRS Dark phase vs. 12:Males CNT Dark phase	-7,261	-12,68 to -1,842	Yes	***	0,0009			
-1:Females CRS Dark phase vs. 12:Males CRS Dark phase	4,817	0,2629 to 9,371	Yes	*	0,0263			
-1:Females CRS Dark phase vs. 12:Females CNT Dark phase	-4,692	-9,376 to -0,008406	Yes	*	0,0491			
-1:Females CRS Dark phase vs. 12:Females CRS Dark phase	2,891	-0,3681 to 6,151	No	ns	0,1372			
-1:Females CRS Dark phase vs. 16:Males CNT Dark phase	-7,614	-12,65 to -2,583	Yes	****	<0,0001			
-1:Females CRS Dark phase vs. 16:Males CRS Dark phase	1,133	-3,938 to 6,204	No	ns	>0,9999			
-1:Females CRS Dark phase vs. 16:Females CNT Dark phase	-8,121	-13,61 to -2,628	Yes	***	0,0003			
-1:Females CRS Dark phase vs. 16:Females CRS Dark phase	2,124	-0,6403 to 4,887	No	ns	0,3342			
-1:Females CRS Dark phase vs. 19:Males CNT Dark phase	-7,564	-13,86 to -1,263	Yes	**	0,0054			
-1:Females CRS Dark phase vs. 19:Males CRS Dark phase	-1,721	-6,811 to 3,370	No	ns	0,9999			
-1:Females CRS Dark phase vs. 19:Females CNT Dark phase	-9,799	-16,46 to -3,134	Yes	***	0,0006			
-1:Females CRS Dark phase vs. 19:Females CRS Dark phase	-1,421	-4,574 to 1,732	No	ns	0,9790			
2:Males CNT Dark phase vs. 2:Males CRS Dark phase	3,425	-2,606 to 9,457	No	ns	0,8821			
2:Males CNT Dark phase vs. 2:Females CNT Dark phase	-0,3573	-6,010 to 5,296	No	ns	>0,9999			
2:Males CNT Dark phase vs. 2:Females CRS Dark phase	3,430	-2,277 to 9,137	No	ns	0,8102			
2:Males CNT Dark phase vs. 5:Males CNT Dark phase	-1,361	-3,409 to 0,6870	No	ns	0,5613			
2:Males CNT Dark phase vs. 5:Males CRS Dark phase	3,250	-2,700 to 9,201	No	ns	0,9144			
2:Males CNT Dark phase vs. 5:Females CNT Dark phase	-0,3015	-6,134 to 5,530	No	ns	>0,9999			
2:Males CNT Dark phase vs. 5:Females CRS Dark phase	4,770	-1,086 to 10,62	No	ns	0,2701			
2:Males CNT Dark phase vs. 9:Males CNT Dark phase	-3,478	-7,234 to 0,2783	No	ns	0,0951			
2:Males CNT Dark phase vs. 9:Males CRS Dark phase	3,294	-3,217 to 9,805	No	ns	0,9618			
2:Males CNT Dark phase vs. 9:Females CNT Dark phase	-1,842	-7,652 to 3,968	No	ns	>0,9999			
2:Males CNT Dark phase vs. 9:Females CRS Dark phase	4,296	-1,074 to 9,665	No	ns	0,2856			
2:Males CNT Dark phase vs. 12:Males CNT Dark phase	-6,804	-11,21 to -2,397	Yes	***	0,0003			
2:Males CNT Dark phase vs. 12:Males CRS Dark phase	5,274	-0,4046 to 10,95	No	ns	0,1009			

2:Males CNT Dark phase vs. 12:Females CNT Dark phase	-4,235	-9,984 to 1,514	No	ns	0,4308				
2:Males CNT Dark phase vs. 12:Females CRS Dark phase	3,348	-2,234 to 8,931	No	ns	0,8073				
2:Males CNT Dark phase vs. 16:Males CNT Dark phase	-7,157	-12,35 to -1,961	Yes	**	0,0013				
2:Males CNT Dark phase vs. 16:Males CRS Dark phase	1,590	-4,475 to 7,656	No	ns	>0,9999				
2:Males CNT Dark phase vs. 16:Females CNT Dark phase	-7,664	-14,02 to -1,309	Yes	**	0,0056				
2:Males CNT Dark phase vs. 16:Females CRS Dark phase	2,581	-2,970 to 8,131	No	ns	0,9812				
2:Males CNT Dark phase vs. 19:Males CNT Dark phase	-7,107	-14,50 to 0,2873	No	ns	0,0704				
2:Males CNT Dark phase vs. 19:Males CRS Dark phase	-1,263	-7,344 to 4,817	No	ns	>0,9999				
2:Males CNT Dark phase vs. 19:Females CNT Dark phase	-9,342	-16,66 to -2,027	Yes	**	0,0029				
2:Males CNT Dark phase vs. 19:Females CRS Dark phase	-0,9640	-6,467 to 4,539	No	ns	>0,9999				
2:Males CRS Dark phase vs. 2:Females CNT Dark phase	-3,782	-8,727 to 1,163	No	ns	0,3682				
2:Males CRS Dark phase vs. 2:Females CRS Dark phase	0,004988	-4,992 to 5,002	No	ns	>0,9999				
2:Males CRS Dark phase vs. 5:Males CNT Dark phase	-4,786	-10,33 to 0,7557	No	ns	0,1850				
2:Males CRS Dark phase vs. 5:Males CRS Dark phase	-0,1747	-3,106 to 2,756	No	ns	>0,9999				
2:Males CRS Dark phase vs. 5:Females CNT Dark phase	-3,727	-8,892 to 1,439	No	ns	0,4745				
2:Males CRS Dark phase vs. 5:Females CRS Dark phase	1,344	-3,831 to 6,520	No	ns	>0,9999				
2:Males CRS Dark phase vs. 9:Males CNT Dark phase	-6,903	-12,54 to -1,261	Yes	**	0,0036				
2:Males CRS Dark phase vs. 9:Males CRS Dark phase	-0,1310	-3,553 to 3,291	No	ns	>0,9999				
2:Males CRS Dark phase vs. 9:Females CNT Dark phase	-5,267	-10,41 to -0,1286	Yes	*	0,0388				
2:Males CRS Dark phase vs. 9:Females CRS Dark phase	0,8707	-3,707 to 5,449	No	ns	>0,9999				
2:Males CRS Dark phase vs. 12:Males CNT Dark phase	-10,23	-15,97 to -4,487	Yes	****	<0,0001				
2:Males CRS Dark phase vs. 12:Males CRS Dark phase	1,849	-1,787 to 5,486	No	ns	0,9254				
2:Males CRS Dark phase vs. 12:Females CNT Dark phase	-7,660	-12,72 to -2,596	Yes	***	0,0001				
2:Males CRS Dark phase vs. 12:Females CRS Dark phase	-0,07669	-4,921 to 4,768	No	ns	>0,9999				
2:Males CRS Dark phase vs. 16:Males CNT Dark phase	-10,58	-15,97 to -5,194	Yes	****	<0,0001				
2:Males CRS Dark phase vs. 16:Males CRS Dark phase	-1,835	-5,204 to 1,535	No	ns	0,8709				
2:Males CRS Dark phase vs. 16:Females CNT Dark phase	-11,09	-16,88 to -5,298	Yes	****	<0,0001				
2:Males CRS Dark phase vs. 16:Females CRS Dark phase	-0,8444	-5,649 to 3,960	No	ns	>0,9999				
2:Males CRS Dark phase vs. 19:Males CNT Dark phase	-10,53	-17,10 to -3,968	Yes	****	<0,0001				
2:Males CRS Dark phase vs. 19:Males CRS Dark phase	-4,689	-8,232 to -1,145	Yes	**	0,0019				
2:Males CRS Dark phase vs. 19:Females CNT Dark phase	-12,77	-19,65 to -5,884	Yes	****	<0,0001				
2:Males CRS Dark phase vs. 19:Females CRS Dark phase	-4,389	-9,135 to 0,3566	No	ns	0,1067				
2:Females CNT Dark phase vs. 2:Females CRS Dark phase	3,787	-0,7280 to 8,303	No	ns	0,2134				
2:Females CNT Dark phase vs. 5:Males CNT Dark phase	-1,004	-6,125 to 4,118	No	ns	>0,9999				
2:Females CNT Dark phase vs. 5:Males CRS Dark phase	3,608	-1,232 to 8,447	No	ns	0,4150				
2:Females CNT Dark phase vs. 5:Females CNT Dark phase	0,05571	-2,203 to 2,314	No	ns	>0,9999				
2:Females CNT Dark phase vs. 5:Females CRS Dark phase	5,127	0,4152 to 9,838	Yes	*	0,0198				
2:Females CNT Dark phase vs. 9:Males CNT Dark phase	-3,120	-8,351 to 2,110	No	ns	0,7982				
2:Females CNT Dark phase vs. 9:Males CRS Dark phase	3,651	-1,899 to 9,201	No	ns	0,6564				
2:Females CNT Dark phase vs. 9:Females CNT Dark phase	-1,485	-4,596 to 1,626	No	ns	0,8620				
2:Females CNT Dark phase vs. 9:Females CRS Dark phase	4,653	0,5953 to 8,711	Yes	*	0,0116				
2:Females CNT Dark phase vs. 12:Males CNT Dark phase	-6,447	-11,79 to -1,107	Yes	**	0,0054				
2:Females CNT Dark phase vs. 12:Males CRS Dark phase	5,632	1,151 to 10,11	Yes	**	0,0031				
2:Females CNT Dark phase vs. 12:Females CNT Dark phase	-3,878	-7,702 to -0,05349	Yes	*	0,0453				
2:Females CNT Dark phase vs. 12:Females CRS Dark phase	3,706	-0,6423 to 8,054	No	ns	0,1890				
2:Females CNT Dark phase vs. 16:Males CNT Dark phase	-6,800	-11,75 to -1,847	Yes	***	0,0008				
2:Females CNT Dark phase vs. 16:Males CRS Dark phase	1,948	-3,042 to 6,937	No	ns	0,9979				
2:Females CNT Dark phase vs. 16:Females CNT Dark phase	-7,306	-12,60 to -2,016	Yes	**	0,0035				
2:Females CNT Dark phase vs. 16:Females CRS Dark phase	2,938	-1,366 to 7,242	No	ns	0,5679				

2:Females CNT Dark phase vs. 19:Males CNT Dark phase	-6,749	-12,97 to -0,5253	Yes	*	0,0211			
2:Females CNT Dark phase vs. 19:Males CRS Dark phase	-0,9062	-5,915 to 4,102	No	ns	>0,9999			
2:Females CNT Dark phase vs. 19:Females CNT Dark phase	-8,985	-14,70 to -3,272	Yes	**	0,0010			
2:Females CNT Dark phase vs. 19:Females CRS Dark phase	-0,6068	-4,847 to 3,633	No	ns	>0,9999			
2:Females CRS Dark phase vs. 5:Males CNT Dark phase	-4,791	-9,963 to 0,3811	No	ns	0,1046			
2:Females CRS Dark phase vs. 5:Males CRS Dark phase	-0,1797	-5,069 to 4,710	No	ns	>0,9999			
2:Females CRS Dark phase vs. 5:Females CNT Dark phase	-3,732	-8,501 to 1,037	No	ns	0,3166			
2:Females CRS Dark phase vs. 5:Females CRS Dark phase	1,339	-1,049 to 3,728	No	ns	0,8486			
2:Females CRS Dark phase vs. 9:Males CNT Dark phase	-6,908	-12,19 to -1,626	Yes	**	0,0013			
2:Females CRS Dark phase vs. 9:Males CRS Dark phase	-0,1360	-5,743 to 5,471	No	ns	>0,9999			
2:Females CRS Dark phase vs. 9:Females CNT Dark phase	-5,272	-10,01 to -0,5341	Yes	*	0,0156			
2:Females CRS Dark phase vs. 9:Females CRS Dark phase	0,8658	-1,955 to 3,686	No	ns	>0,9999			
2:Females CRS Dark phase vs. 12:Males CNT Dark phase	-10,23	-15,63 to -4,842	Yes	****	<0,0001			
2:Females CRS Dark phase vs. 12:Males CRS Dark phase	1,844	-2,676 to 6,364	No	ns	0,9978			
2:Females CRS Dark phase vs. 12:Females CNT Dark phase	-7,665	-12,32 to -3,013	Yes	****	<0,0001			
2:Females CRS Dark phase vs. 12:Females CRS Dark phase	-0,08167	-3,154 to 2,991	No	ns	>0,9999			
2:Females CRS Dark phase vs. 16:Males CNT Dark phase	-10,59	-15,59 to -5,585	Yes	****	<0,0001			
2:Females CRS Dark phase vs. 16:Males CRS Dark phase	-1,840	-6,881 to 3,202	No	ns	0,9996			
2:Females CRS Dark phase vs. 16:Females CNT Dark phase	-11,09	-16,56 to -5,625	Yes	****	<0,0001			
2:Females CRS Dark phase vs. 16:Females CRS Dark phase	-0,8494	-4,161 to 2,462	No	ns	>0,9999			
2:Females CRS Dark phase vs. 19:Males CNT Dark phase	-10,54	-16,82 to -4,258	Yes	****	<0,0001			
2:Females CRS Dark phase vs. 19:Males CRS Dark phase	-4,693	-9,754 to 0,3674	No	ns	0,1047			
2:Females CRS Dark phase vs. 19:Females CNT Dark phase	-12,77	-19,42 to -6,124	Yes	****	<0,0001			
2:Females CRS Dark phase vs. 19:Females CRS Dark phase	-4,394	-7,093 to -1,696	Yes	****	<0,0001			
5:Males CNT Dark phase vs. 5:Males CRS Dark phase	4,611	-0,8389 to 10,06	No	ns	0,2138			
5:Males CNT Dark phase vs. 5:Females CNT Dark phase	1,059	-4,270 to 6,388	No	ns	>0,9999			
5:Males CNT Dark phase vs. 5:Females CRS Dark phase	6,130	0,7892 to 11,47	Yes	**	0,0093			
5:Males CNT Dark phase vs. 9:Males CNT Dark phase	-2,117	-4,612 to 0,3784	No	ns	0,1770			
5:Males CNT Dark phase vs. 9:Males CRS Dark phase	4,655	-1,421 to 10,73	No	ns	0,3870			
5:Males CNT Dark phase vs. 9:Females CNT Dark phase	-0,4814	-5,785 to 4,822	No	ns	>0,9999			
5:Males CNT Dark phase vs. 9:Females CRS Dark phase	5,657	0,8765 to 10,44	Yes	**	0,0066			
5:Males CNT Dark phase vs. 12:Males CNT Dark phase	-5,443	-8,259 to -2,628	Yes	****	<0,0001			
5:Males CNT Dark phase vs. 12:Males CRS Dark phase	6,635	1,495 to 11,78	Yes	**	0,0016			
5:Males CNT Dark phase vs. 12:Females CNT Dark phase	-2,874	-8,107 to 2,359	No	ns	0,8896			
5:Males CNT Dark phase vs. 12:Females CRS Dark phase	4,709	-0,3194 to 9,738	No	ns	0,0944			
5:Males CNT Dark phase vs. 16:Males CNT Dark phase	-5,796	-9,512 to -2,081	Yes	***	0,0002			
5:Males CNT Dark phase vs. 16:Males CRS Dark phase	2,951	-2,629 to 8,532	No	ns	0,9400			
5:Males CNT Dark phase vs. 16:Females CNT Dark phase	-6,303	-12,23 to -0,3783	Yes	*	0,0265			
5:Males CNT Dark phase vs. 16:Females CRS Dark phase	3,942	-1,050 to 8,933	No	ns	0,3219			
5:Males CNT Dark phase vs. 19:Males CNT Dark phase	-5,746	-12,06 to 0,5725	No	ns	0,1091			
5:Males CNT Dark phase vs. 19:Males CRS Dark phase	0,09745	-5,500 to 5,694	No	ns	>0,9999			
5:Males CNT Dark phase vs. 19:Females CNT Dark phase	-7,981	-14,96 to -0,9999	Yes	*	0,0126			
5:Males CNT Dark phase vs. 19:Females CRS Dark phase	0,3968	-4,540 to 5,333	No	ns	>0,9999			
5:Males CRS Dark phase vs. 5:Females CNT Dark phase	-3,552	-8,620 to 1,516	No	ns	0,5275			
5:Males CRS Dark phase vs. 5:Females CRS Dark phase	1,519	-3,554 to 6,593	No	ns	>0,9999			
5:Males CRS Dark phase vs. 9:Males CNT Dark phase	-6,728	-12,28 to -1,176	Yes	**	0,0043			
5:Males CRS Dark phase vs. 9:Males CRS Dark phase	0,04368	-1,534 to 1,621	No	ns	>0,9999			
5:Males CRS Dark phase vs. 9:Females CNT Dark phase	-5,093	-10,13 to -0,05269	Yes	*	0,0450			
5:Males CRS Dark phase vs. 9:Females CRS Dark phase	1,045	-3,411 to 5,502	No	ns	>0,9999			

5:Males CRS Dark phase vs. 12:Males CNT Dark phase	-10,05	-15,71 to -4,400	Yes	****	<0,0001			
5:Males CRS Dark phase vs. 12:Males CRS Dark phase	2,024	-0,5355 to 4,583	No	ns	0,2816			
5:Males CRS Dark phase vs. 12:Females CNT Dark phase	-7,485	-12,45 to -2,523	Yes	***	0,0001			
5:Males CRS Dark phase vs. 12:Females CRS Dark phase	0,09804	-4,634 to 4,830	No	ns	>0,9999			
5:Males CRS Dark phase vs. 16:Males CNT Dark phase	-10,41	-15,70 to -5,115	Yes	****	<0,0001			
5:Males CRS Dark phase vs. 16:Males CRS Dark phase	-1,660	-2,998 to -0,3218	Yes	**	0,0045			
5:Males CRS Dark phase vs. 16:Females CNT Dark phase	-10,91	-16,62 to -5,204	Yes	****	<0,0001			
5:Males CRS Dark phase vs. 16:Females CRS Dark phase	-0,6697	-5,361 to 4,021	No	ns	>0,9999			
5:Males CRS Dark phase vs. 19:Males CNT Dark phase	-10,36	-16,85 to -3,865	Yes	****	<0,0001			
5:Males CRS Dark phase vs. 19:Males CRS Dark phase	-4,514	-6,117 to -2,911	Yes	****	<0,0001			
5:Males CRS Dark phase vs. 19:Females CNT Dark phase	-12,59	-19,42 to -5,770	Yes	****	<0,0001			
5:Males CRS Dark phase vs. 19:Females CRS Dark phase	-4,214	-8,845 to 0,4161	No	ns	0,1229			
5:Females CNT Dark phase vs. 5:Females CRS Dark phase	5,071	0,1216 to 10,02	Yes	*	0,0390			
5:Females CNT Dark phase vs. 9:Males CNT Dark phase	-3,176	-8,608 to 2,255	No	ns	0,8228			
5:Females CNT Dark phase vs. 9:Males CRS Dark phase	3,596	-2,140 to 9,331	No	ns	0,7372			
5:Females CNT Dark phase vs. 9:Females CNT Dark phase	-1,541	-5,643 to 2,561	No	ns	0,9795			
5:Females CNT Dark phase vs. 9:Females CRS Dark phase	4,597	0,2410 to 8,954	Yes	*	0,0297			
5:Females CNT Dark phase vs. 12:Males CNT Dark phase	-6,503	-12,04 to -0,9679	Yes	**	0,0078			
5:Females CNT Dark phase vs. 12:Males CRS Dark phase	5,576	0,8394 to 10,31	Yes	**	0,0079			
5:Females CNT Dark phase vs. 12:Females CNT Dark phase	-3,934	-8,213 to 0,3454	No	ns	0,0877			
5:Females CNT Dark phase vs. 12:Females CRS Dark phase	3,650	-0,9667 to 8,267	No	ns	0,2935			
5:Females CNT Dark phase vs. 16:Males CNT Dark phase	-6,856	-12,03 to -1,684	Yes	**	0,0014			
5:Females CNT Dark phase vs. 16:Males CRS Dark phase	1,892	-3,315 to 7,099	No	ns	0,9992			
5:Females CNT Dark phase vs. 16:Females CNT Dark phase	-7,362	-13,02 to -1,709	Yes	**	0,0060			
5:Females CNT Dark phase vs. 16:Females CRS Dark phase	2,882	-1,695 to 7,459	No	ns	0,6971			
5:Females CNT Dark phase vs. 19:Males CNT Dark phase	-6,805	-13,18 to -0,4260	Yes	*	0,0253			
5:Females CNT Dark phase vs. 19:Males CRS Dark phase	-0,9619	-6,187 to 4,263	No	ns	>0,9999			
5:Females CNT Dark phase vs. 19:Females CNT Dark phase	-9,041	-15,63 to -2,447	Yes	**	0,0038			
5:Females CNT Dark phase vs. 19:Females CRS Dark phase	-0,6625	-5,182 to 3,857	No	ns	>0,9999			
5:Females CRS Dark phase vs. 9:Males CNT Dark phase	-8,247	-13,69 to -2,801	Yes	****	<0,0001			
5:Females CRS Dark phase vs. 9:Males CRS Dark phase	-1,475	-7,236 to 4,285	No	ns	>0,9999			
5:Females CRS Dark phase vs. 9:Females CNT Dark phase	-6,612	-11,53 to -1,691	Yes	**	0,0011			
5:Females CRS Dark phase vs. 9:Females CRS Dark phase	-0,4737	-4,112 to 3,165	No	ns	>0,9999			
5:Females CRS Dark phase vs. 12:Males CNT Dark phase	-11,57	-17,13 to -6,021	Yes	****	<0,0001			
5:Females CRS Dark phase vs. 12:Males CRS Dark phase	0,5047	-4,219 to 5,229	No	ns	>0,9999			
5:Females CRS Dark phase vs. 12:Females CNT Dark phase	-9,005	-13,84 to -4,164	Yes	****	<0,0001			
5:Females CRS Dark phase vs. 12:Females CRS Dark phase	-1,421	-5,351 to 2,509	No	ns	0,9988			
5:Females CRS Dark phase vs. 16:Males CNT Dark phase	-11,93	-17,11 to -6,748	Yes	****	<0,0001			
5:Females CRS Dark phase vs. 16:Males CRS Dark phase	-3,179	-8,397 to 2,039	No	ns	0,8132			
5:Females CRS Dark phase vs. 16:Females CNT Dark phase	-12,43	-18,05 to -6,819	Yes	****	<0,0001			
5:Females CRS Dark phase vs. 16:Females CRS Dark phase	-2,189	-6,612 to 2,234	No	ns	0,9459			
5:Females CRS Dark phase vs. 19:Males CNT Dark phase	-11,88	-18,29 to -5,467	Yes	****	<0,0001			
5:Females CRS Dark phase vs. 19:Males CRS Dark phase	-6,033	-11,27 to -0,7963	Yes	**	0,0085			
5:Females CRS Dark phase vs. 19:Females CNT Dark phase	-14,11	-20,87 to -7,358	Yes	****	<0,0001			
5:Females CRS Dark phase vs. 19:Females CRS Dark phase	-5,734	-9,214 to -2,253	Yes	****	<0,0001			
9:Males CNT Dark phase vs. 9:Males CRS Dark phase	6,772	0,6081 to 12,94	Yes	*	0,0166			
9:Males CNT Dark phase vs. 9:Females CNT Dark phase	1,635	-3,771 to 7,042	No	ns	>0,9999			
9:Males CNT Dark phase vs. 9:Females CRS Dark phase	7,774	2,871 to 12,68	Yes	****	<0,0001			
9:Males CNT Dark phase vs. 12:Males CNT Dark phase	-3,327	-5,089 to -1,564	Yes	****	<0,0001			

9:Males CNT Dark phase vs. 12:Males CRS Dark phase	8,752	3,501 to 14,00	Yes	****	<0,0001			
9:Males CNT Dark phase vs. 12:Females CNT Dark phase	-0,7574	-6,096 to 4,581	No	ns	>0,9999			
9:Males CNT Dark phase vs. 12:Females CRS Dark phase	6,826	1,683 to 11,97	Yes	**	0,0010			
9:Males CNT Dark phase vs. 16:Males CNT Dark phase	-3,679	-6,486 to -0,8731	Yes	**	0,0026			
9:Males CNT Dark phase vs. 16:Males CRS Dark phase	5,068	-0,6111 to 10,75	No	ns	0,1438			
9:Males CNT Dark phase vs. 16:Females CNT Dark phase	-4,186	-10,20 to 1,825	No	ns	0,5251			
9:Males CNT Dark phase vs. 16:Females CRS Dark phase	6,058	0,9519 to 11,16	Yes	**	0,0061			
9:Males CNT Dark phase vs. 19:Males CNT Dark phase	-3,629	-9,417 to 2,159	No	ns	0,6605			
9:Males CNT Dark phase vs. 19:Males CRS Dark phase	2,214	-3,481 to 7,910	No	ns	0,9987			
9:Males CNT Dark phase vs. 19:Females CNT Dark phase	-5,865	-12,91 to 1,183	No	ns	0,2071			
9:Males CNT Dark phase vs. 19:Females CRS Dark phase	2,514	-2,540 to 7,567	No	ns	0,9628			
9:Males CRS Dark phase vs. 9:Females CNT Dark phase	-5,136	-10,85 to 0,5767	No	ns	0,1308			
9:Males CRS Dark phase vs. 9:Females CRS Dark phase	1,002	-4,252 to 6,256	No	ns	>0,9999			
9:Males CRS Dark phase vs. 12:Males CNT Dark phase	-10,10	-16,35 to -3,846	Yes	****	<0,0001			
9:Males CRS Dark phase vs. 12:Males CRS Dark phase	1,980	-1,460 to 5,420	No	ns	0,8095			
9:Males CRS Dark phase vs. 12:Females CNT Dark phase	-7,529	-13,18 to -1,879	Yes	**	0,0011			
9:Males CRS Dark phase vs. 12:Females CRS Dark phase	0,05435	-5,423 to 5,532	No	ns	>0,9999			
9:Males CRS Dark phase vs. 16:Males CNT Dark phase	-10,45	-16,39 to -4,509	Yes	****	<0,0001			
9:Males CRS Dark phase vs. 16:Males CRS Dark phase	-1,704	-3,862 to 0,4549	No	ns	0,2846			
9:Males CRS Dark phase vs. 16:Females CNT Dark phase	-10,96	-17,23 to -4,683	Yes	****	<0,0001			
9:Males CRS Dark phase vs. 16:Females CRS Dark phase	-0,7134	-6,157 to 4,730	No	ns	>0,9999			
9:Males CRS Dark phase vs. 19:Males CNT Dark phase	-10,40	-17,39 to -3,407	Yes	***	0,0001			
9:Males CRS Dark phase vs. 19:Males CRS Dark phase	-4,557	-6,739 to -2,376	Yes	****	<0,0001			
9:Males CRS Dark phase vs. 19:Females CNT Dark phase	-12,64	-19,89 to -5,382	Yes	****	<0,0001			
9:Males CRS Dark phase vs. 19:Females CRS Dark phase	-4,258	-9,652 to 1,136	No	ns	0,3240			
9:Females CNT Dark phase vs. 9:Females CRS Dark phase	6,138	1,818 to 10,46	Yes	***	0,0007			
9:Females CNT Dark phase vs. 12:Males CNT Dark phase	-4,962	-10,47 to 0,5489	No	ns	0,1271			
9:Females CNT Dark phase vs. 12:Males CRS Dark phase	7,117	2,411 to 11,82	Yes	***	0,0002			
9:Females CNT Dark phase vs. 12:Females CNT Dark phase	-2,393	-4,559 to -0,2265	Yes	*	0,0239			
9:Females CNT Dark phase vs. 12:Females CRS Dark phase	5,191	0,6065 to 9,775	Yes	*	0,0130			
9:Females CNT Dark phase vs. 16:Males CNT Dark phase	-5,315	-10,46 to -0,1695	Yes	*	0,0359			
9:Females CNT Dark phase vs. 16:Males CRS Dark phase	3,433	-1,748 to 8,613	No	ns	0,6369			
9:Females CNT Dark phase vs. 16:Females CNT Dark phase	-5,821	-9,233 to -2,410	Yes	***	0,0004			
9:Females CNT Dark phase vs. 16:Females CRS Dark phase	4,423	-0,1209 to 8,967	No	ns	0,0644			
9:Females CNT Dark phase vs. 19:Males CNT Dark phase	-5,264	-11,62 to 1,096	No	ns	0,2304			
9:Females CNT Dark phase vs. 19:Males CRS Dark phase	0,5788	-4,620 to 5,777	No	ns	>0,9999			
9:Females CNT Dark phase vs. 19:Females CNT Dark phase	-7,500	-10,65 to -4,353	Yes	****	<0,0001			
9:Females CNT Dark phase vs. 19:Females CRS Dark phase	0,8782	-3,607 to 5,364	No	ns	>0,9999			
9:Females CRS Dark phase vs. 12:Males CNT Dark phase	-11,10	-16,12 to -6,075	Yes	****	<0,0001			
9:Females CRS Dark phase vs. 12:Males CRS Dark phase	0,9785	-3,053 to 5,010	No	ns	>0,9999			
9:Females CRS Dark phase vs. 12:Females CNT Dark phase	-8,531	-12,75 to -4,311	Yes	****	<0,0001			
9:Females CRS Dark phase vs. 12:Females CRS Dark phase	-0,9474	-2,375 to 0,4806	No	ns	0,5996			
9:Females CRS Dark phase vs. 16:Males CNT Dark phase	-11,45	-16,04 to -6,864	Yes	****	<0,0001			
9:Females CRS Dark phase vs. 16:Males CRS Dark phase	-2,705	-7,334 to 1,923	No	ns	0,8509			
9:Females CRS Dark phase vs. 16:Females CNT Dark phase	-11,96	-17,11 to -6,810	Yes	****	<0,0001			
9:Females CRS Dark phase vs. 16:Females CRS Dark phase	-1,715	-3,390 to -0,04060	Yes	*	0,0396			
9:Females CRS Dark phase vs. 19:Males CNT Dark phase	-11,40	-17,39 to -5,418	Yes	****	<0,0001			
9:Females CRS Dark phase vs. 19:Males CRS Dark phase	-5,559	-10,21 to -0,9090	Yes	**	0,0053			
9:Females CRS Dark phase vs. 19:Females CNT Dark phase	-13,64	-20,06 to -7,212	Yes	****	<0,0001			

9:Females CRS Dark phase vs. 19:Females CRS Dark phase	-5,260	-6,835 to -3,684	Yes	****	<0,0001				
12:Males CNT Dark phase vs. 12:Males CRS Dark phase	12,08	6,717 to 17,44	Yes	****	<0,0001				
12:Males CNT Dark phase vs. 12:Females CNT Dark phase	2,569	-2,876 to 8,014	No	ns	0,9739				
12:Males CNT Dark phase vs. 12:Females CRS Dark phase	10,15	4,895 to 15,41	Yes	****	<0,0001				
12:Males CNT Dark phase vs. 16:Males CNT Dark phase	-0,3528	-2,497 to 1,791	No	ns	>0,9999				
12:Males CNT Dark phase vs. 16:Males CRS Dark phase	8,395	2,616 to 14,17	Yes	***	0,0002				
12:Males CNT Dark phase vs. 16:Females CNT Dark phase	-0,8594	-6,958 to 5,239	No	ns	>0,9999				
12:Males CNT Dark phase vs. 16:Females CRS Dark phase	9,385	4,163 to 14,61	Yes	****	<0,0001				
12:Males CNT Dark phase vs. 19:Males CNT Dark phase	-0,3024	-5,439 to 4,834	No	ns	>0,9999				
12:Males CNT Dark phase vs. 19:Males CRS Dark phase	5,541	-0,2539 to 11,34	No	ns	0,0784				
12:Males CNT Dark phase vs. 19:Females CNT Dark phase	-2,538	-9,653 to 4,577	No	ns	0,9988				
12:Males CNT Dark phase vs. 19:Females CRS Dark phase	5,840	0,6695 to 11,01	Yes	*	0,0122				
12:Males CRS Dark phase vs. 12:Females CNT Dark phase	-9,509	-14,13 to -4,890	Yes	****	<0,0001				
12:Males CRS Dark phase vs. 12:Females CRS Dark phase	-1,926	-6,270 to 2,418	No	ns	0,9928				
12:Males CRS Dark phase vs. 16:Males CNT Dark phase	-12,43	-17,40 to -7,463	Yes	****	<0,0001				
12:Males CRS Dark phase vs. 16:Males CRS Dark phase	-3,684	-6,144 to -1,224	Yes	***	0,0003				
12:Males CRS Dark phase vs. 16:Females CNT Dark phase	-12,94	-18,38 to -7,496	Yes	****	<0,0001				
12:Males CRS Dark phase vs. 16:Females CRS Dark phase	-2,694	-6,991 to 1,604	No	ns	0,7728				
12:Males CRS Dark phase vs. 19:Males CNT Dark phase	-12,38	-18,63 to -6,127	Yes	****	<0,0001				
12:Males CRS Dark phase vs. 19:Males CRS Dark phase	-6,538	-8,848 to -4,227	Yes	****	<0,0001				
12:Males CRS Dark phase vs. 19:Females CNT Dark phase	-14,62	-21,25 to -7,987	Yes	****	<0,0001				
12:Males CRS Dark phase vs. 19:Females CRS Dark phase	-6,238	-10,47 to -2,009	Yes	***	0,0001				
12:Females CNT Dark phase vs. 12:Females CRS Dark phase	7,584	3,090 to 12,08	Yes	****	<0,0001				
12:Females CNT Dark phase vs. 16:Males CNT Dark phase	-2,922	-7,993 to 2,149	No	ns	0,8393				
12:Females CNT Dark phase vs. 16:Males CRS Dark phase	5,826	0,7187 to 10,93	Yes	*	0,0111				
12:Females CNT Dark phase vs. 16:Females CNT Dark phase	-3,429	-6,010 to -0,8473	Yes	**	0,0050				
12:Females CNT Dark phase vs. 16:Females CRS Dark phase	6,816	2,364 to 11,27	Yes	***	0,0001				
12:Females CNT Dark phase vs. 19:Males CNT Dark phase	-2,872	-9,179 to 3,436	No	ns	0,9819				
12:Females CNT Dark phase vs. 19:Males CRS Dark phase	2,972	-2,153 to 8,097	No	ns	0,8409				
12:Females CNT Dark phase vs. 19:Females CNT Dark phase	-5,107	-8,874 to -1,341	Yes	**	0,0042				
12:Females CNT Dark phase vs. 19:Females CRS Dark phase	3,271	-1,120 to 7,663	No	ns	0,3946				
12:Females CRS Dark phase vs. 16:Males CNT Dark phase	-10,51	-15,36 to -5,654	Yes	****	<0,0001				
12:Females CRS Dark phase vs. 16:Males CRS Dark phase	-1,758	-6,649 to 3,133	No	ns	0,9997				
12:Females CRS Dark phase vs. 16:Females CNT Dark phase	-11,01	-16,36 to -5,663	Yes	****	<0,0001				
12:Females CRS Dark phase vs. 16:Females CRS Dark phase	-0,7677	-2,229 to 0,6936	No	ns	0,9086				
12:Females CRS Dark phase vs. 19:Males CNT Dark phase	-10,46	-16,63 to -4,285	Yes	****	<0,0001				
12:Females CRS Dark phase vs. 19:Males CRS Dark phase	-4,612	-9,523 to 0,2995	No	ns	0,0932				
12:Females CRS Dark phase vs. 19:Females CNT Dark phase	-12,69	-19,25 to -6,127	Yes	****	<0,0001				
12:Females CRS Dark phase vs. 19:Females CRS Dark phase	-4,312	-6,123 to -2,502	Yes	****	<0,0001				
16:Males CNT Dark phase vs. 16:Males CRS Dark phase	8,748	3,319 to 14,18	Yes	****	<0,0001				
16:Males CNT Dark phase vs. 16:Females CNT Dark phase	-0,5066	-6,300 to 5,287	No	ns	>0,9999				
16:Males CNT Dark phase vs. 16:Females CRS Dark phase	9,738	4,926 to 14,55	Yes	****	<0,0001				
16:Males CNT Dark phase vs. 19:Males CNT Dark phase	0,05040	-4,601 to 4,702	No	ns	>0,9999				
16:Males CNT Dark phase vs. 19:Males CRS Dark phase	5,894	0,4479 to 11,34	Yes	*	0,0201				
16:Males CNT Dark phase vs. 19:Females CNT Dark phase	-2,185	-9,068 to 4,698	No	ns	0,9997				
16:Males CNT Dark phase vs. 19:Females CRS Dark phase	6,193	1,439 to 10,95	Yes	**	0,0014				
16:Males CRS Dark phase vs. 16:Females CNT Dark phase	-9,254	-15,08 to -3,429	Yes	****	<0,0001				
16:Males CRS Dark phase vs. 16:Females CRS Dark phase	0,9902	-3,861 to 5,842	No	ns	>0,9999				
16:Males CRS Dark phase vs. 19:Males CNT Dark phase	-8,697	-15,29 to -2,102	Yes	**	0,0012				

16:Males CRS Dark phase vs. 19:Males CRS Dark phase	-2,854	-4,392 to -1,315	Yes	****	<0,0001			
16:Males CRS Dark phase vs. 19:Females CNT Dark phase	-10,93	-17,84 to -4,024	Yes	***	0,0001			
16:Males CRS Dark phase vs. 19:Females CRS Dark phase	-2,554	-7,349 to 2,240	No	ns	0,9357			
16:Females CNT Dark phase vs. 16:Females CRS Dark phase	10,24	4,927 to 15,56	Yes	****	<0,0001			
16:Females CNT Dark phase vs. 19:Males CNT Dark phase	0,5570	-6,282 to 7,396	No	ns	>0,9999			
16:Females CNT Dark phase vs. 19:Males CRS Dark phase	6,400	0,5604 to 12,24	Yes	*	0,0189			
16:Females CNT Dark phase vs. 19:Females CNT Dark phase	-1,679	-5,271 to 1,913	No	ns	0,8796			
16:Females CNT Dark phase vs. 19:Females CRS Dark phase	6,700	1,427 to 11,97	Yes	**	0,0037			
16:Females CRS Dark phase vs. 19:Males CNT Dark phase	-9,687	-15,83 to -3,546	Yes	****	<0,0001			
16:Females CRS Dark phase vs. 19:Males CRS Dark phase	-3,844	-8,716 to 1,028	No	ns	0,3321			
16:Females CRS Dark phase vs. 19:Females CNT Dark phase	-11,92	-18,46 to -5,381	Yes	****	<0,0001			
16:Females CRS Dark phase vs. 19:Females CRS Dark phase	-3,545	-5,409 to -1,681	Yes	****	<0,0001			
19:Males CNT Dark phase vs. 19:Males CRS Dark phase	5,843	-0,7645 to 12,45	No	ns	0,1520			
19:Males CNT Dark phase vs. 19:Females CNT Dark phase	-2,236	-9,941 to 5,470	No	ns	>0,9999			
19:Males CNT Dark phase vs. 19:Females CRS Dark phase	6,143	0,04190 to 12,24	Yes	*	0,0467			
19:Males CRS Dark phase vs. 19:Females CNT Dark phase	-8,079	-15,00 to -1,159	Yes	**	0,0099			
19:Males CRS Dark phase vs. 19:Females CRS Dark phase	0,2994	-4,515 to 5,114	No	ns	>0,9999			
19:Females CNT Dark phase vs. 19:Females CRS Dark phase	8,378	1,867 to 14,89	Yes	**	0,0040			
Test details	Mean 1	Mean 2	Mean Diff,	SE of diff,	N1	N2	q	DF
-1:Males CNT Dark phase vs. -1:Males CRS Dark phase	99,48	99,64	-0,1613	1,571	26	30	0,1452	53,11
-1:Males CNT Dark phase vs. -1:Females CNT Dark phase	99,48	101,0	-1,542	1,369	26	13	1,594	36,94
-1:Males CNT Dark phase vs. -1:Females CRS Dark phase	99,48	99,71	-0,2317	1,417	26	32	0,2312	48,07
-1:Males CNT Dark phase vs. 2:Males CNT Dark phase	99,48	100,2	-0,6888	0,6605	26	26	1,475	25,00
-1:Males CNT Dark phase vs. 2:Males CRS Dark phase	99,48	96,74	2,736	1,501	26	30	2,578	51,28
-1:Males CNT Dark phase vs. 2:Females CNT Dark phase	99,48	100,5	-1,046	1,375	26	14	1,076	37,84
-1:Males CNT Dark phase vs. 2:Females CRS Dark phase	99,48	96,74	2,741	1,410	26	32	2,750	47,62
-1:Males CNT Dark phase vs. 5:Males CNT Dark phase	99,48	101,5	-2,050	0,6762	26	26	4,287	25,00
-1:Males CNT Dark phase vs. 5:Males CRS Dark phase	99,48	96,92	2,562	1,479	26	30	2,450	50,43
-1:Males CNT Dark phase vs. 5:Females CNT Dark phase	99,48	100,5	-0,9904	1,422	26	14	0,9849	37,97
-1:Males CNT Dark phase vs. 5:Females CRS Dark phase	99,48	95,40	4,081	1,453	26	32	3,972	50,12
-1:Males CNT Dark phase vs. 9:Males CNT Dark phase	99,48	103,6	-4,167	0,9376	26	26	6,285	25,00
-1:Males CNT Dark phase vs. 9:Males CRS Dark phase	99,48	96,87	2,605	1,631	26	30	2,259	53,85
-1:Males CNT Dark phase vs. 9:Females CNT Dark phase	99,48	102,0	-2,531	1,416	26	14	2,527	37,99
-1:Males CNT Dark phase vs. 9:Females CRS Dark phase	99,48	95,87	3,607	1,306	26	32	3,906	39,80
-1:Males CNT Dark phase vs. 12:Males CNT Dark phase	99,48	107,0	-7,493	1,016	26	26	10,43	25,00
-1:Males CNT Dark phase vs. 12:Males CRS Dark phase	99,48	94,89	4,585	1,400	26	30	4,631	46,46
-1:Males CNT Dark phase vs. 12:Females CNT Dark phase	99,48	104,4	-4,924	1,401	26	14	4,971	37,99
-1:Males CNT Dark phase vs. 12:Females CRS Dark phase	99,48	96,82	2,660	1,373	26	32	2,740	45,09
-1:Males CNT Dark phase vs. 16:Males CNT Dark phase	99,48	107,3	-7,846	1,201	26	26	9,239	25,00
-1:Males CNT Dark phase vs. 16:Males CRS Dark phase	99,48	98,58	0,9016	1,511	26	30	0,8440	51,60
-1:Males CNT Dark phase vs. 16:Females CNT Dark phase	99,48	107,8	-8,353	1,551	26	14	7,618	35,84
-1:Males CNT Dark phase vs. 16:Females CRS Dark phase	99,48	97,58	1,892	1,363	26	32	1,963	44,36
-1:Males CNT Dark phase vs. 19:Males CNT Dark phase	99,48	107,3	-7,796	1,654	26	26	6,665	25,00
-1:Males CNT Dark phase vs. 19:Males CRS Dark phase	99,48	101,4	-1,952	1,515	26	30	1,823	51,73
-1:Males CNT Dark phase vs. 19:Females CNT Dark phase	99,48	109,5	-10,03	1,770	26	14	8,016	30,15
-1:Males CNT Dark phase vs. 19:Females CRS Dark phase	99,48	101,1	-1,653	1,348	26	32	1,734	43,25
-1:Males CRS Dark phase vs. -1:Females CNT Dark phase	99,64	101,0	-1,381	1,318	30	13	1,482	40,75
-1:Males CRS Dark phase vs. -1:Females CRS Dark phase	99,64	99,71	-0,07042	1,368	30	32	0,07278	55,66
-1:Males CRS Dark phase vs. 2:Males CNT Dark phase	99,64	100,2	-0,5275	1,602	30	26	0,4657	52,55

-1:Males CRS Dark phase vs. 2:Males CRS Dark phase	99,64	96,74	2,898	0,7877	30	30	5,202	29,00
-1:Males CRS Dark phase vs. 2:Females CNT Dark phase	99,64	100,5	-0,8848	1,325	30	14	0,9444	41,86
-1:Males CRS Dark phase vs. 2:Females CRS Dark phase	99,64	96,74	2,903	1,361	30	32	3,016	55,26
-1:Males CRS Dark phase vs. 5:Males CNT Dark phase	99,64	101,5	-1,888	1,487	30	26	1,796	53,98
-1:Males CRS Dark phase vs. 5:Males CRS Dark phase	99,64	96,92	2,723	0,5029	30	30	7,656	29,00
-1:Males CRS Dark phase vs. 5:Females CNT Dark phase	99,64	100,5	-0,8291	1,374	30	14	0,8536	41,00
-1:Males CRS Dark phase vs. 5:Females CRS Dark phase	99,64	95,40	4,242	1,405	30	32	4,268	57,37
-1:Males CRS Dark phase vs. 9:Males CNT Dark phase	99,64	103,6	-4,005	1,511	30	26	3,749	53,85
-1:Males CRS Dark phase vs. 9:Males CRS Dark phase	99,64	96,87	2,767	0,5402	30	30	7,242	29,00
-1:Males CRS Dark phase vs. 9:Females CNT Dark phase	99,64	102,0	-2,370	1,368	30	14	2,450	41,13
-1:Males CRS Dark phase vs. 9:Females CRS Dark phase	99,64	95,87	3,768	1,253	30	32	4,254	47,24
-1:Males CRS Dark phase vs. 12:Males CNT Dark phase	99,64	107,0	-7,332	1,534	30	26	6,757	53,62
-1:Males CRS Dark phase vs. 12:Males CRS Dark phase	99,64	94,89	4,747	0,7371	30	30	9,107	29,00
-1:Males CRS Dark phase vs. 12:Females CNT Dark phase	99,64	104,4	-4,763	1,351	30	14	4,984	41,48
-1:Males CRS Dark phase vs. 12:Females CRS Dark phase	99,64	96,82	2,821	1,322	30	32	3,017	52,86
-1:Males CRS Dark phase vs. 16:Males CNT Dark phase	99,64	107,3	-7,685	1,451	30	26	7,491	53,94
-1:Males CRS Dark phase vs. 16:Males CRS Dark phase	99,64	98,58	1,063	0,5125	30	30	2,933	29,00
-1:Males CRS Dark phase vs. 16:Females CNT Dark phase	99,64	107,8	-8,191	1,506	30	14	7,691	36,66
-1:Males CRS Dark phase vs. 16:Females CRS Dark phase	99,64	97,58	2,053	1,312	30	32	2,213	52,13
-1:Males CRS Dark phase vs. 19:Males CNT Dark phase	99,64	107,3	-7,634	1,726	30	26	6,256	49,76
-1:Males CRS Dark phase vs. 19:Males CRS Dark phase	99,64	101,4	-1,791	0,5267	30	30	4,809	29,00
-1:Males CRS Dark phase vs. 19:Females CNT Dark phase	99,64	109,5	-9,870	1,731	30	14	8,064	29,48
-1:Males CRS Dark phase vs. 19:Females CRS Dark phase	99,64	101,1	-1,492	1,297	30	32	1,627	50,99
-1:Females CNT Dark phase vs. -1:Females CRS Dark phase	101,0	99,71	1,311	1,130	13	32	1,640	37,71
-1:Females CNT Dark phase vs. 2:Males CNT Dark phase	101,0	100,2	0,8536	1,404	13	26	0,8598	36,80
-1:Females CNT Dark phase vs. 2:Males CRS Dark phase	101,0	96,74	4,279	1,234	13	30	4,904	39,70
-1:Females CNT Dark phase vs. 2:Females CNT Dark phase	101,0	100,5	0,5531	0,3484	13	13	2,245	12,00
-1:Females CNT Dark phase vs. 2:Females CRS Dark phase	101,0	96,74	4,284	1,121	13	32	5,404	37,31
-1:Females CNT Dark phase vs. 5:Males CNT Dark phase	101,0	101,5	-0,5072	1,271	13	26	0,5642	36,86
-1:Females CNT Dark phase vs. 5:Males CRS Dark phase	101,0	96,92	4,104	1,206	13	30	4,812	39,12
-1:Females CNT Dark phase vs. 5:Females CNT Dark phase	101,0	100,4	0,6131	0,6606	13	13	1,312	12,00
-1:Females CNT Dark phase vs. 5:Females CRS Dark phase	101,0	95,40	5,623	1,175	13	32	6,769	39,45
-1:Females CNT Dark phase vs. 9:Males CNT Dark phase	101,0	103,6	-2,624	1,299	13	26	2,857	36,97
-1:Females CNT Dark phase vs. 9:Males CRS Dark phase	101,0	96,87	4,148	1,389	13	30	4,223	41,00
-1:Females CNT Dark phase vs. 9:Females CNT Dark phase	101,0	102,2	-1,171	0,6568	13	13	2,521	12,00
-1:Females CNT Dark phase vs. 9:Females CRS Dark phase	101,0	95,87	5,149	0,9869	13	32	7,379	29,28
-1:Females CNT Dark phase vs. 12:Males CNT Dark phase	101,0	107,0	-5,951	1,326	13	26	6,345	37,00
-1:Females CNT Dark phase vs. 12:Males CRS Dark phase	101,0	94,89	6,128	1,109	13	30	7,814	35,90
-1:Females CNT Dark phase vs. 12:Females CNT Dark phase	101,0	104,5	-3,458	0,9482	13	13	5,157	12,00
-1:Females CNT Dark phase vs. 12:Females CRS Dark phase	101,0	96,82	4,202	1,074	13	32	5,534	34,90
-1:Females CNT Dark phase vs. 16:Males CNT Dark phase	101,0	107,3	-6,303	1,229	13	26	7,256	36,52
-1:Females CNT Dark phase vs. 16:Males CRS Dark phase	101,0	98,58	2,444	1,246	13	30	2,775	39,91
-1:Females CNT Dark phase vs. 16:Females CNT Dark phase	101,0	107,8	-6,777	1,222	13	13	7,843	12,00
-1:Females CNT Dark phase vs. 16:Females CRS Dark phase	101,0	97,58	3,434	1,061	13	32	4,577	34,17
-1:Females CNT Dark phase vs. 19:Males CNT Dark phase	101,0	107,3	-6,253	1,544	13	26	5,729	35,83
-1:Females CNT Dark phase vs. 19:Males CRS Dark phase	101,0	101,4	-0,4098	1,250	13	30	0,4634	39,99
-1:Females CNT Dark phase vs. 19:Females CNT Dark phase	101,0	109,8	-8,792	1,250	13	13	9,945	12,00
-1:Females CNT Dark phase vs. 19:Females CRS Dark phase	101,0	101,1	-0,1104	1,042	13	32	0,1498	33,03
-1:Females CRS Dark phase vs. 2:Males CNT Dark phase	99,71	100,2	-0,4571	1,452	32	26	0,4454	46,94

-1:Females CRS Dark phase vs. 2:Males CRS Dark phase	99,71	96,74	2,968	1,288	32	30	3,260	58,09
-1:Females CRS Dark phase vs. 2:Females CNT Dark phase	99,71	100,5	-0,8144	1,138	32	14	1,012	39,21
-1:Females CRS Dark phase vs. 2:Females CRS Dark phase	99,71	96,74	2,973	0,7856	32	32	5,352	31,00
-1:Females CRS Dark phase vs. 5:Males CNT Dark phase	99,71	101,5	-1,818	1,324	32	26	1,942	51,30
-1:Females CRS Dark phase vs. 5:Males CRS Dark phase	99,71	96,92	2,793	1,261	32	30	3,132	58,76
-1:Females CRS Dark phase vs. 5:Females CNT Dark phase	99,71	100,5	-0,7587	1,194	32	14	0,8982	36,34
-1:Females CRS Dark phase vs. 5:Females CRS Dark phase	99,71	95,40	4,312	1,094	32	32	5,573	31,00
-1:Females CRS Dark phase vs. 9:Males CNT Dark phase	99,71	103,6	-3,935	1,350	32	26	4,122	50,38
-1:Females CRS Dark phase vs. 9:Males CRS Dark phase	99,71	96,87	2,837	1,437	32	30	2,792	53,47
-1:Females CRS Dark phase vs. 9:Females CNT Dark phase	99,71	102,0	-2,299	1,188	32	14	2,738	36,67
-1:Females CRS Dark phase vs. 9:Females CRS Dark phase	99,71	95,87	3,839	0,7024	32	32	7,729	31,00
-1:Females CRS Dark phase vs. 12:Males CNT Dark phase	99,71	107,0	-7,261	1,376	32	26	7,460	49,46
-1:Females CRS Dark phase vs. 12:Males CRS Dark phase	99,71	94,89	4,817	1,168	32	30	5,830	60,00
-1:Females CRS Dark phase vs. 12:Females CNT Dark phase	99,71	104,4	-4,692	1,169	32	14	5,677	37,63
-1:Females CRS Dark phase vs. 12:Females CRS Dark phase	99,71	96,82	2,891	0,8008	32	32	5,106	31,00
-1:Females CRS Dark phase vs. 16:Males CNT Dark phase	99,71	107,3	-7,614	1,283	32	26	8,396	52,71
-1:Females CRS Dark phase vs. 16:Males CRS Dark phase	99,71	98,58	1,133	1,299	32	30	1,234	57,78
-1:Females CRS Dark phase vs. 16:Females CNT Dark phase	99,71	107,8	-8,121	1,345	32	14	8,540	29,85
-1:Females CRS Dark phase vs. 16:Females CRS Dark phase	99,71	97,58	2,124	0,6790	32	32	4,423	31,00
-1:Females CRS Dark phase vs. 19:Males CNT Dark phase	99,71	107,3	-7,564	1,587	32	26	6,741	43,02
-1:Females CRS Dark phase vs. 19:Males CRS Dark phase	99,71	101,4	-1,721	1,303	32	30	1,867	57,64
-1:Females CRS Dark phase vs. 19:Females CNT Dark phase	99,71	109,5	-9,799	1,592	32	14	8,702	23,50
-1:Females CRS Dark phase vs. 19:Females CRS Dark phase	99,71	101,1	-1,421	0,7746	32	32	2,595	31,00
2:Males CNT Dark phase vs. 2:Males CRS Dark phase	100,2	96,74	3,425	1,534	26	30	3,158	50,44
2:Males CNT Dark phase vs. 2:Females CNT Dark phase	100,2	100,5	-0,3573	1,411	26	14	0,3581	37,65
2:Males CNT Dark phase vs. 2:Females CRS Dark phase	100,2	96,74	3,430	1,444	26	32	3,358	46,49
2:Males CNT Dark phase vs. 5:Males CNT Dark phase	100,2	101,5	-1,361	0,4926	26	26	3,907	25,00
2:Males CNT Dark phase vs. 5:Males CRS Dark phase	100,2	96,92	3,250	1,512	26	30	3,041	49,52
2:Males CNT Dark phase vs. 5:Females CNT Dark phase	100,2	100,5	-0,3015	1,456	26	14	0,2928	38,00
2:Males CNT Dark phase vs. 5:Females CRS Dark phase	100,2	95,40	4,770	1,487	26	32	4,537	49,03
2:Males CNT Dark phase vs. 9:Males CNT Dark phase	100,2	103,6	-3,478	0,9035	26	26	5,443	25,00
2:Males CNT Dark phase vs. 9:Males CRS Dark phase	100,2	96,87	3,294	1,661	26	30	2,805	53,57
2:Males CNT Dark phase vs. 9:Females CNT Dark phase	100,2	102,0	-1,842	1,451	26	14	1,796	37,99
2:Males CNT Dark phase vs. 9:Females CRS Dark phase	100,2	95,87	4,296	1,343	26	32	4,523	38,87
2:Males CNT Dark phase vs. 12:Males CNT Dark phase	100,2	107,0	-6,804	1,060	26	26	9,077	25,00
2:Males CNT Dark phase vs. 12:Males CRS Dark phase	100,2	94,89	5,274	1,435	26	30	5,197	45,40
2:Males CNT Dark phase vs. 12:Females CNT Dark phase	100,2	104,4	-4,235	1,436	26	14	4,172	37,92
2:Males CNT Dark phase vs. 12:Females CRS Dark phase	100,2	96,82	3,348	1,408	26	32	3,363	43,97
2:Males CNT Dark phase vs. 16:Males CNT Dark phase	100,2	107,3	-7,157	1,250	26	26	8,098	25,00
2:Males CNT Dark phase vs. 16:Males CRS Dark phase	100,2	98,58	1,590	1,543	26	30	1,458	50,79
2:Males CNT Dark phase vs. 16:Females CNT Dark phase	100,2	107,8	-7,664	1,582	26	14	6,851	36,44
2:Males CNT Dark phase vs. 16:Females CRS Dark phase	100,2	97,58	2,581	1,398	26	32	2,610	43,26
2:Males CNT Dark phase vs. 19:Males CNT Dark phase	100,2	107,3	-7,107	1,779	26	26	5,651	25,00
2:Males CNT Dark phase vs. 19:Males CRS Dark phase	100,2	101,4	-1,263	1,547	26	30	1,155	50,94
2:Males CNT Dark phase vs. 19:Females CNT Dark phase	100,2	109,5	-9,342	1,797	26	14	7,351	31,06
2:Males CNT Dark phase vs. 19:Females CRS Dark phase	100,2	101,1	-0,9640	1,384	26	32	0,9849	42,18
2:Males CRS Dark phase vs. 2:Females CNT Dark phase	96,74	100,5	-3,782	1,241	30	14	4,309	40,98
2:Males CRS Dark phase vs. 2:Females CRS Dark phase	96,74	96,74	0,004988	1,280	30	32	0,005512	57,78
2:Males CRS Dark phase vs. 5:Males CNT Dark phase	96,74	101,5	-4,786	1,413	30	26	4,789	53,21

2:Males CRS Dark phase vs. 5:Males CRS Dark phase	96,74	96,92	-0,1747	0,7157	30	30	0,3453	29,00
2:Males CRS Dark phase vs. 5:Females CNT Dark phase	96,74	100,5	-3,727	1,293	30	14	4,076	39,29
2:Males CRS Dark phase vs. 5:Females CRS Dark phase	96,74	95,40	1,344	1,327	30	32	1,433	59,23
2:Males CRS Dark phase vs. 9:Males CNT Dark phase	96,74	103,6	-6,903	1,438	30	26	6,788	52,75
2:Males CRS Dark phase vs. 9:Males CRS Dark phase	96,74	96,87	-0,1310	0,8357	30	30	0,2218	29,00
2:Males CRS Dark phase vs. 9:Females CNT Dark phase	96,74	102,0	-5,267	1,287	30	14	5,788	39,52
2:Males CRS Dark phase vs. 9:Females CRS Dark phase	96,74	95,87	0,8707	1,164	30	32	1,058	50,33
2:Males CRS Dark phase vs. 12:Males CNT Dark phase	96,74	107,0	-10,23	1,463	30	26	9,889	52,21
2:Males CRS Dark phase vs. 12:Males CRS Dark phase	96,74	94,89	1,849	0,8880	30	30	2,945	29,00
2:Males CRS Dark phase vs. 12:Females CNT Dark phase	96,74	104,4	-7,660	1,270	30	14	8,533	40,12
2:Males CRS Dark phase vs. 12:Females CRS Dark phase	96,74	96,82	-0,07669	1,238	30	32	0,08757	55,80
2:Males CRS Dark phase vs. 16:Males CNT Dark phase	96,74	107,3	-10,58	1,375	30	26	10,88	53,76
2:Males CRS Dark phase vs. 16:Males CRS Dark phase	96,74	98,58	-1,835	0,8228	30	30	3,153	29,00
2:Males CRS Dark phase vs. 16:Females CNT Dark phase	96,74	107,8	-11,09	1,433	30	14	10,94	33,83
2:Males CRS Dark phase vs. 16:Females CRS Dark phase	96,74	97,58	-0,8444	1,227	30	32	0,9729	55,14
2:Males CRS Dark phase vs. 19:Males CNT Dark phase	96,74	107,3	-10,53	1,662	30	26	8,959	46,99
2:Males CRS Dark phase vs. 19:Males CRS Dark phase	96,74	101,4	-4,689	0,8653	30	30	7,663	29,00
2:Males CRS Dark phase vs. 19:Females CNT Dark phase	96,74	109,5	-12,77	1,668	30	14	10,83	26,79
2:Males CRS Dark phase vs. 19:Females CRS Dark phase	96,74	101,1	-4,389	1,211	30	32	5,124	54,08
2:Females CNT Dark phase vs. 2:Females CRS Dark phase	100,5	96,74	3,787	1,129	14	32	4,743	38,81
2:Females CNT Dark phase vs. 5:Males CNT Dark phase	100,5	101,5	-1,004	1,279	14	26	1,110	37,94
2:Females CNT Dark phase vs. 5:Males CRS Dark phase	100,5	96,92	3,608	1,214	14	30	4,203	40,45
2:Females CNT Dark phase vs. 5:Females CNT Dark phase	100,5	100,5	0,05571	0,4931	14	14	0,1598	13,00
2:Females CNT Dark phase vs. 5:Females CRS Dark phase	100,5	95,40	5,127	1,183	14	32	6,130	40,88
2:Females CNT Dark phase vs. 9:Males CNT Dark phase	100,5	103,6	-3,120	1,306	14	26	3,379	38,00
2:Females CNT Dark phase vs. 9:Males CRS Dark phase	100,5	96,87	3,651	1,396	14	30	3,700	41,99
2:Females CNT Dark phase vs. 9:Females CNT Dark phase	100,5	102,0	-1,485	0,6791	14	14	3,092	13,00
2:Females CNT Dark phase vs. 9:Females CRS Dark phase	100,5	95,87	4,653	0,9964	14	32	6,604	30,82
2:Females CNT Dark phase vs. 12:Males CNT Dark phase	100,5	107,0	-6,447	1,333	14	26	6,838	37,98
2:Females CNT Dark phase vs. 12:Males CRS Dark phase	100,5	94,89	5,632	1,117	14	30	7,127	37,38
2:Females CNT Dark phase vs. 12:Females CNT Dark phase	100,5	104,4	-3,878	0,8350	14	14	6,568	13,00
2:Females CNT Dark phase vs. 12:Females CRS Dark phase	100,5	96,82	3,706	1,082	14	32	4,841	36,45
2:Females CNT Dark phase vs. 16:Males CNT Dark phase	100,5	107,3	-6,800	1,236	14	26	7,779	37,68
2:Females CNT Dark phase vs. 16:Males CRS Dark phase	100,5	98,58	1,948	1,253	14	30	2,198	41,16
2:Females CNT Dark phase vs. 16:Females CNT Dark phase	100,5	107,8	-7,306	1,155	14	14	8,947	13,00
2:Females CNT Dark phase vs. 16:Females CRS Dark phase	100,5	97,58	2,938	1,070	14	32	3,884	35,72
2:Females CNT Dark phase vs. 19:Males CNT Dark phase	100,5	107,3	-6,749	1,550	14	26	6,159	36,49
2:Females CNT Dark phase vs. 19:Males CRS Dark phase	100,5	101,4	-0,9062	1,258	14	30	1,019	41,23
2:Females CNT Dark phase vs. 19:Females CNT Dark phase	100,5	109,5	-8,985	1,247	14	14	10,19	13,00
2:Females CNT Dark phase vs. 19:Females CRS Dark phase	100,5	101,1	-0,6068	1,051	14	32	0,8162	34,59
2:Females CRS Dark phase vs. 5:Males CNT Dark phase	96,74	101,5	-4,791	1,316	32	26	5,149	50,89
2:Females CRS Dark phase vs. 5:Males CRS Dark phase	96,74	96,92	-0,1797	1,253	32	30	0,2028	58,51
2:Females CRS Dark phase vs. 5:Females CNT Dark phase	96,74	100,5	-3,732	1,186	32	14	4,450	35,89
2:Females CRS Dark phase vs. 5:Females CRS Dark phase	96,74	95,40	1,339	0,5867	32	32	3,229	31,00
2:Females CRS Dark phase vs. 9:Males CNT Dark phase	96,74	103,6	-6,908	1,342	32	26	7,277	49,95
2:Females CRS Dark phase vs. 9:Males CRS Dark phase	96,74	96,87	-0,1360	1,430	32	30	0,1346	53,03
2:Females CRS Dark phase vs. 9:Females CNT Dark phase	96,74	102,0	-5,272	1,179	32	14	6,324	36,23
2:Females CRS Dark phase vs. 9:Females CRS Dark phase	96,74	95,87	0,8658	0,6929	32	32	1,767	31,00
2:Females CRS Dark phase vs. 12:Males CNT Dark phase	96,74	107,0	-10,23	1,369	32	26	10,57	49,01

2:Females CRS Dark phase vs. 12:Males CRS Dark phase	96,74	94,89	1,844	1,160	32	30	2,249	59,99
2:Females CRS Dark phase vs. 12:Females CNT Dark phase	96,74	104,4	-7,665	1,160	32	14	9,344	37,21
2:Females CRS Dark phase vs. 12:Females CRS Dark phase	96,74	96,82	-0,08167	0,7548	32	32	0,1530	31,00
2:Females CRS Dark phase vs. 16:Males CNT Dark phase	96,74	107,3	-10,59	1,275	32	26	11,75	52,34
2:Females CRS Dark phase vs. 16:Males CRS Dark phase	96,74	98,58	-1,840	1,291	32	30	2,015	57,46
2:Females CRS Dark phase vs. 16:Females CNT Dark phase	96,74	107,8	-11,09	1,337	32	14	11,73	29,43
2:Females CRS Dark phase vs. 16:Females CRS Dark phase	96,74	97,58	-0,8494	0,8136	32	32	1,476	31,00
2:Females CRS Dark phase vs. 19:Males CNT Dark phase	96,74	107,3	-10,54	1,580	32	26	9,429	42,59
2:Females CRS Dark phase vs. 19:Males CRS Dark phase	96,74	101,4	-4,693	1,296	32	30	5,123	57,32
2:Females CRS Dark phase vs. 19:Females CNT Dark phase	96,74	109,5	-12,77	1,586	32	14	11,39	23,20
2:Females CRS Dark phase vs. 19:Females CRS Dark phase	96,74	101,1	-4,394	0,6630	32	32	9,373	31,00
5:Males CNT Dark phase vs. 5:Males CRS Dark phase	101,5	96,92	4,611	1,389	26	30	4,694	52,68
5:Males CNT Dark phase vs. 5:Females CNT Dark phase	101,5	100,5	1,059	1,329	26	14	1,127	37,28
5:Males CNT Dark phase vs. 5:Females CRS Dark phase	101,5	95,40	6,130	1,362	26	32	6,366	53,05
5:Males CNT Dark phase vs. 9:Males CNT Dark phase	101,5	103,6	-2,117	0,6003	26	26	4,987	25,00
5:Males CNT Dark phase vs. 9:Males CRS Dark phase	101,5	96,87	4,655	1,551	26	30	4,246	53,83
5:Males CNT Dark phase vs. 9:Females CNT Dark phase	101,5	102,0	-0,4814	1,323	26	14	0,5146	37,39
5:Males CNT Dark phase vs. 9:Females CRS Dark phase	101,5	95,87	5,657	1,204	26	32	6,646	42,86
5:Males CNT Dark phase vs. 12:Males CNT Dark phase	101,5	107,0	-5,443	0,6772	26	26	11,37	25,00
5:Males CNT Dark phase vs. 12:Males CRS Dark phase	101,5	94,89	6,635	1,306	26	30	7,187	49,50
5:Males CNT Dark phase vs. 12:Females CNT Dark phase	101,5	104,4	-2,874	1,306	26	14	3,112	37,66
5:Males CNT Dark phase vs. 12:Females CRS Dark phase	101,5	96,82	4,709	1,276	26	32	5,220	48,47
5:Males CNT Dark phase vs. 16:Males CNT Dark phase	101,5	107,3	-5,796	0,8938	26	26	9,172	25,00
5:Males CNT Dark phase vs. 16:Males CRS Dark phase	101,5	98,58	2,951	1,423	26	30	2,932	53,39
5:Males CNT Dark phase vs. 16:Females CNT Dark phase	101,5	107,8	-6,303	1,466	26	14	6,082	33,63
5:Males CNT Dark phase vs. 16:Females CRS Dark phase	101,5	97,58	3,942	1,265	26	32	4,406	47,73
5:Males CNT Dark phase vs. 19:Males CNT Dark phase	101,5	107,3	-5,746	1,520	26	26	5,346	25,00
5:Males CNT Dark phase vs. 19:Males CRS Dark phase	101,5	101,4	0,09745	1,428	26	30	0,09652	53,46
5:Males CNT Dark phase vs. 19:Females CNT Dark phase	101,5	109,5	-7,981	1,696	26	14	6,656	27,44
5:Males CNT Dark phase vs. 19:Females CRS Dark phase	101,5	101,1	0,3968	1,250	26	32	0,4491	46,60
5:Males CRS Dark phase vs. 5:Females CNT Dark phase	96,92	100,5	-3,552	1,267	30	14	3,965	38,50
5:Males CRS Dark phase vs. 5:Females CRS Dark phase	96,92	95,40	1,519	1,301	30	32	1,651	59,64
5:Males CRS Dark phase vs. 9:Males CNT Dark phase	96,92	103,6	-6,728	1,414	30	26	6,727	52,11
5:Males CRS Dark phase vs. 9:Males CRS Dark phase	96,92	96,87	0,04368	0,3852	30	30	0,1604	29,00
5:Males CRS Dark phase vs. 9:Females CNT Dark phase	96,92	102,0	-5,093	1,260	30	14	5,714	38,75
5:Males CRS Dark phase vs. 9:Females CRS Dark phase	96,92	95,87	1,045	1,135	30	32	1,303	51,47
5:Males CRS Dark phase vs. 12:Males CNT Dark phase	96,92	107,0	-10,05	1,440	30	26	9,877	51,47
5:Males CRS Dark phase vs. 12:Males CRS Dark phase	96,92	94,89	2,024	0,6250	30	30	4,580	29,00
5:Males CRS Dark phase vs. 12:Females CNT Dark phase	96,92	104,4	-7,485	1,243	30	14	8,519	39,44
5:Males CRS Dark phase vs. 12:Females CRS Dark phase	96,92	96,82	0,09804	1,211	30	32	0,1145	56,75
5:Males CRS Dark phase vs. 16:Males CNT Dark phase	96,92	107,3	-10,41	1,350	30	26	10,90	53,42
5:Males CRS Dark phase vs. 16:Males CRS Dark phase	96,92	98,58	-1,660	0,3268	30	30	7,184	29,00
5:Males CRS Dark phase vs. 16:Females CNT Dark phase	96,92	107,8	-10,91	1,409	30	14	10,95	32,77
5:Males CRS Dark phase vs. 16:Females CRS Dark phase	96,92	97,58	-0,6697	1,200	30	32	0,7895	56,14
5:Males CRS Dark phase vs. 19:Males CNT Dark phase	96,92	107,3	-10,36	1,642	30	26	8,920	45,92
5:Males CRS Dark phase vs. 19:Males CRS Dark phase	96,92	101,4	-4,514	0,3914	30	30	16,31	29,00
5:Males CRS Dark phase vs. 19:Females CNT Dark phase	96,92	109,5	-12,59	1,647	30	14	10,81	25,89
5:Males CRS Dark phase vs. 19:Females CRS Dark phase	96,92	101,1	-4,214	1,183	30	32	5,038	55,14
5:Females CNT Dark phase vs. 5:Females CRS Dark phase	100,5	95,40	5,071	1,237	14	32	5,798	38,33

5:Females CNT Dark phase vs. 9:Males CNT Dark phase	100,5	103,6	-3,176	1,355	14	26	3,314	37,58
5:Females CNT Dark phase vs. 9:Males CRS Dark phase	100,5	96,87	3,596	1,442	14	30	3,527	41,73
5:Females CNT Dark phase vs. 9:Females CNT Dark phase	100,5	102,0	-1,541	0,8956	14	14	2,433	13,00
5:Females CNT Dark phase vs. 9:Females CRS Dark phase	100,5	95,87	4,597	1,060	14	32	6,134	27,95
5:Females CNT Dark phase vs. 12:Males CNT Dark phase	100,5	107,0	-6,503	1,382	14	26	6,656	37,79
5:Females CNT Dark phase vs. 12:Males CRS Dark phase	100,5	94,89	5,576	1,175	14	30	6,713	34,66
5:Females CNT Dark phase vs. 12:Females CNT Dark phase	100,5	104,4	-3,934	0,9342	14	14	5,955	13,00
5:Females CNT Dark phase vs. 12:Females CRS Dark phase	100,5	96,82	3,650	1,141	14	32	4,523	33,36
5:Females CNT Dark phase vs. 16:Males CNT Dark phase	100,5	107,3	-6,856	1,288	14	26	7,527	36,62
5:Females CNT Dark phase vs. 16:Males CRS Dark phase	100,5	98,58	1,892	1,304	14	30	2,052	39,59
5:Females CNT Dark phase vs. 16:Females CNT Dark phase	100,5	107,8	-7,362	1,234	14	14	8,436	13,00
5:Females CNT Dark phase vs. 16:Females CRS Dark phase	100,5	97,58	2,882	1,129	14	32	3,609	32,62
5:Females CNT Dark phase vs. 19:Males CNT Dark phase	100,5	107,3	-6,805	1,591	14	26	6,048	37,45
5:Females CNT Dark phase vs. 19:Males CRS Dark phase	100,5	101,4	-0,9619	1,309	14	30	1,039	39,71
5:Females CNT Dark phase vs. 19:Females CNT Dark phase	100,5	109,5	-9,041	1,440	14	14	8,881	13,00
5:Females CNT Dark phase vs. 19:Females CRS Dark phase	100,5	101,1	-0,6625	1,112	14	32	0,8427	31,50
5:Females CRS Dark phase vs. 9:Males CNT Dark phase	95,40	103,6	-8,247	1,388	32	26	8,405	52,24
5:Females CRS Dark phase vs. 9:Males CRS Dark phase	95,40	96,87	-1,475	1,472	32	30	1,417	55,44
5:Females CRS Dark phase vs. 9:Females CNT Dark phase	95,40	102,0	-6,612	1,230	32	14	7,600	38,64
5:Females CRS Dark phase vs. 9:Females CRS Dark phase	95,40	95,87	-0,4737	0,8938	32	32	0,7495	31,00
5:Females CRS Dark phase vs. 12:Males CNT Dark phase	95,40	107,0	-11,57	1,413	32	26	11,58	51,41
5:Females CRS Dark phase vs. 12:Males CRS Dark phase	95,40	94,89	0,5047	1,212	32	30	0,5891	59,72
5:Females CRS Dark phase vs. 12:Females CNT Dark phase	95,40	104,4	-9,005	1,212	32	14	10,51	39,51
5:Females CRS Dark phase vs. 12:Females CRS Dark phase	95,40	96,82	-1,421	0,9655	32	32	2,082	31,00
5:Females CRS Dark phase vs. 16:Males CNT Dark phase	95,40	107,3	-11,93	1,322	32	26	12,76	54,20
5:Females CRS Dark phase vs. 16:Males CRS Dark phase	95,40	98,58	-3,179	1,338	32	30	3,361	59,02
5:Females CRS Dark phase vs. 16:Females CNT Dark phase	95,40	107,8	-12,43	1,383	32	14	12,72	31,89
5:Females CRS Dark phase vs. 16:Females CRS Dark phase	95,40	97,58	-2,189	1,087	32	32	2,849	31,00
5:Females CRS Dark phase vs. 19:Males CNT Dark phase	95,40	107,3	-11,88	1,619	32	26	10,37	45,07
5:Females CRS Dark phase vs. 19:Males CRS Dark phase	95,40	101,4	-6,033	1,342	32	30	6,356	58,92
5:Females CRS Dark phase vs. 19:Females CNT Dark phase	95,40	109,5	-14,11	1,625	32	14	12,29	24,99
5:Females CRS Dark phase vs. 19:Females CRS Dark phase	95,40	101,1	-5,734	0,8551	32	32	9,482	31,00
9:Males CNT Dark phase vs. 9:Males CRS Dark phase	103,6	96,87	6,772	1,573	26	30	6,088	53,97
9:Males CNT Dark phase vs. 9:Females CNT Dark phase	103,6	102,0	1,635	1,349	26	14	1,714	37,66
9:Males CNT Dark phase vs. 9:Females CRS Dark phase	103,6	95,87	7,774	1,233	26	32	8,918	41,91
9:Males CNT Dark phase vs. 12:Males CNT Dark phase	103,6	107,0	-3,327	0,4239	26	26	11,10	25,00
9:Males CNT Dark phase vs. 12:Males CRS Dark phase	103,6	94,89	8,752	1,333	26	30	9,289	48,62
9:Males CNT Dark phase vs. 12:Females CNT Dark phase	103,6	104,4	-0,7574	1,333	26	14	0,8036	37,85
9:Males CNT Dark phase vs. 12:Females CRS Dark phase	103,6	96,82	6,826	1,303	26	32	7,407	47,46
9:Males CNT Dark phase vs. 16:Males CNT Dark phase	103,6	107,3	-3,679	0,6751	26	26	7,708	25,00
9:Males CNT Dark phase vs. 16:Males CRS Dark phase	103,6	98,58	5,068	1,448	26	30	4,950	52,97
9:Males CNT Dark phase vs. 16:Females CNT Dark phase	103,6	107,8	-4,186	1,490	26	14	3,974	34,35
9:Males CNT Dark phase vs. 16:Females CRS Dark phase	103,6	97,58	6,058	1,293	26	32	6,627	46,72
9:Males CNT Dark phase vs. 19:Males CNT Dark phase	103,6	107,3	-3,629	1,392	26	26	3,686	25,00
9:Males CNT Dark phase vs. 19:Males CRS Dark phase	103,6	101,4	2,214	1,452	26	30	2,156	53,06
9:Males CNT Dark phase vs. 19:Females CNT Dark phase	103,6	109,5	-5,865	1,716	26	14	4,832	28,24
9:Males CNT Dark phase vs. 19:Females CRS Dark phase	103,6	101,1	2,514	1,278	26	32	2,783	45,58
9:Males CRS Dark phase vs. 9:Females CNT Dark phase	96,87	102,0	-5,136	1,436	30	14	5,058	41,80
9:Males CRS Dark phase vs. 9:Females CRS Dark phase	96,87	95,87	1,002	1,327	30	32	1,067	45,06

9:Males CRS Dark phase vs. 12:Males CNT Dark phase	96,87	107,0	-10,10	1,596	30	26	8,949	54,00
9:Males CRS Dark phase vs. 12:Males CRS Dark phase	96,87	94,89	1,980	0,8400	30	30	3,334	29,00
9:Males CRS Dark phase vs. 12:Females CNT Dark phase	96,87	104,4	-7,529	1,421	30	14	7,495	41,94
9:Males CRS Dark phase vs. 12:Females CRS Dark phase	96,87	96,82	0,05435	1,393	30	32	0,05518	50,51
9:Males CRS Dark phase vs. 16:Males CNT Dark phase	96,87	107,3	-10,45	1,516	30	26	9,752	53,37
9:Males CRS Dark phase vs. 16:Males CRS Dark phase	96,87	98,58	-1,704	0,5271	30	30	4,571	29,00
9:Males CRS Dark phase vs. 16:Females CNT Dark phase	96,87	107,8	-10,96	1,569	30	14	9,879	38,54
9:Males CRS Dark phase vs. 16:Females CRS Dark phase	96,87	97,58	-0,7134	1,383	30	32	0,7294	49,77
9:Males CRS Dark phase vs. 19:Males CNT Dark phase	96,87	107,3	-10,40	1,781	30	26	8,261	51,56
9:Males CRS Dark phase vs. 19:Males CRS Dark phase	96,87	101,4	-4,557	0,5326	30	30	12,10	29,00
9:Males CRS Dark phase vs. 19:Females CNT Dark phase	96,87	109,5	-12,64	1,786	30	14	10,01	31,65
9:Males CRS Dark phase vs. 19:Females CRS Dark phase	96,87	101,1	-4,258	1,369	30	32	4,399	48,64
9:Females CNT Dark phase vs. 9:Females CRS Dark phase	102,0	95,87	6,138	1,052	14	32	8,248	28,26
9:Females CNT Dark phase vs. 12:Males CNT Dark phase	102,0	107,0	-4,962	1,376	14	26	5,100	37,85
9:Females CNT Dark phase vs. 12:Males CRS Dark phase	102,0	94,89	7,117	1,168	14	30	8,619	34,98
9:Females CNT Dark phase vs. 12:Females CNT Dark phase	102,0	104,4	-2,393	0,4730	14	14	7,155	13,00
9:Females CNT Dark phase vs. 12:Females CRS Dark phase	102,0	96,82	5,191	1,134	14	32	6,472	33,71
9:Females CNT Dark phase vs. 16:Males CNT Dark phase	102,0	107,3	-5,315	1,282	14	26	5,864	36,77
9:Females CNT Dark phase vs. 16:Males CRS Dark phase	102,0	98,58	3,433	1,298	14	30	3,740	39,81
9:Females CNT Dark phase vs. 16:Females CNT Dark phase	102,0	107,8	-5,821	0,7448	14	14	11,05	13,00
9:Females CNT Dark phase vs. 16:Females CRS Dark phase	102,0	97,58	4,423	1,122	14	32	5,574	32,97
9:Females CNT Dark phase vs. 19:Males CNT Dark phase	102,0	107,3	-5,264	1,586	14	26	4,693	37,36
9:Females CNT Dark phase vs. 19:Males CRS Dark phase	102,0	101,4	0,5788	1,303	14	30	0,6284	39,92
9:Females CNT Dark phase vs. 19:Females CNT Dark phase	102,0	109,5	-7,500	0,6870	14	14	15,44	13,00
9:Females CNT Dark phase vs. 19:Females CRS Dark phase	102,0	101,1	0,8782	1,105	14	32	1,124	31,84
9:Females CRS Dark phase vs. 12:Males CNT Dark phase	95,87	107,0	-11,10	1,262	32	26	12,44	41,03
9:Females CRS Dark phase vs. 12:Males CRS Dark phase	95,87	94,89	0,9785	1,031	32	30	1,343	55,80
9:Females CRS Dark phase vs. 12:Females CNT Dark phase	95,87	104,4	-8,531	1,031	32	14	11,70	29,17
9:Females CRS Dark phase vs. 12:Females CRS Dark phase	95,87	96,82	-0,9474	0,3508	32	32	3,819	31,00
9:Females CRS Dark phase vs. 16:Males CNT Dark phase	95,87	107,3	-11,45	1,158	32	26	13,98	44,47
9:Females CRS Dark phase vs. 16:Males CRS Dark phase	95,87	98,58	-2,705	1,176	32	30	3,253	49,86
9:Females CRS Dark phase vs. 16:Females CNT Dark phase	95,87	107,8	-11,96	1,227	32	14	13,78	22,96
9:Females CRS Dark phase vs. 16:Females CRS Dark phase	95,87	97,58	-1,715	0,4114	32	32	5,896	31,00
9:Females CRS Dark phase vs. 19:Males CNT Dark phase	95,87	107,3	-11,40	1,488	32	26	10,83	35,93
9:Females CRS Dark phase vs. 19:Males CRS Dark phase	95,87	101,4	-5,559	1,182	32	30	6,654	49,67
9:Females CRS Dark phase vs. 19:Females CNT Dark phase	95,87	109,5	-13,64	1,494	32	14	12,91	18,97
9:Females CRS Dark phase vs. 19:Females CRS Dark phase	95,87	101,1	-5,260	0,3870	32	32	19,22	31,00
12:Males CNT Dark phase vs. 12:Males CRS Dark phase	107,0	94,89	12,08	1,359	26	30	12,57	47,76
12:Males CNT Dark phase vs. 12:Females CNT Dark phase	107,0	104,4	2,569	1,360	26	14	2,672	37,96
12:Males CNT Dark phase vs. 12:Females CRS Dark phase	107,0	96,82	10,15	1,331	26	32	10,79	46,49
12:Males CNT Dark phase vs. 16:Males CNT Dark phase	107,0	107,3	-0,3528	0,5157	26	26	0,9675	25,00
12:Males CNT Dark phase vs. 16:Males CRS Dark phase	107,0	98,58	8,395	1,473	26	30	8,061	52,48
12:Males CNT Dark phase vs. 16:Females CNT Dark phase	107,0	107,8	-0,8594	1,514	26	14	0,8030	34,99
12:Males CNT Dark phase vs. 16:Females CRS Dark phase	107,0	97,58	9,385	1,320	26	32	10,05	45,75
12:Males CNT Dark phase vs. 19:Males CNT Dark phase	107,0	107,3	-0,3024	1,236	26	26	0,3461	25,00
12:Males CNT Dark phase vs. 19:Males CRS Dark phase	107,0	101,4	5,541	1,477	26	30	5,306	52,58
12:Males CNT Dark phase vs. 19:Females CNT Dark phase	107,0	109,5	-2,538	1,737	26	14	2,066	29,01
12:Males CNT Dark phase vs. 19:Females CRS Dark phase	107,0	101,1	5,840	1,305	26	32	6,327	44,62
12:Males CRS Dark phase vs. 12:Females CNT Dark phase	94,89	104,4	-9,509	1,149	30	14	11,71	35,89

12:Males CRS Dark phase vs. 12:Females CRS Dark phase	94,89	96,82	-1,926	1,114	30	32	2,445	59,46
12:Males CRS Dark phase vs. 16:Males CNT Dark phase	94,89	107,3	-12,43	1,264	30	26	13,91	50,83
12:Males CRS Dark phase vs. 16:Males CRS Dark phase	94,89	98,58	-3,684	0,6007	30	30	8,672	29,00
12:Males CRS Dark phase vs. 16:Females CNT Dark phase	94,89	107,8	-12,94	1,327	30	14	13,79	28,61
12:Males CRS Dark phase vs. 16:Females CRS Dark phase	94,89	97,58	-2,694	1,102	30	32	3,457	59,16
12:Males CRS Dark phase vs. 19:Males CNT Dark phase	94,89	107,3	-12,38	1,572	30	26	11,14	41,74
12:Males CRS Dark phase vs. 19:Males CRS Dark phase	94,89	101,4	-6,538	0,5641	30	30	16,39	29,00
12:Males CRS Dark phase vs. 19:Females CNT Dark phase	94,89	109,5	-14,62	1,578	30	14	13,10	22,73
12:Males CRS Dark phase vs. 19:Females CRS Dark phase	94,89	101,1	-6,238	1,084	30	32	8,140	58,58
12:Females CNT Dark phase vs. 12:Females CRS Dark phase	104,4	96,82	7,584	1,115	14	32	9,623	34,72
12:Females CNT Dark phase vs. 16:Males CNT Dark phase	104,4	107,3	-2,922	1,264	14	26	3,268	37,17
12:Females CNT Dark phase vs. 16:Males CRS Dark phase	104,4	98,58	5,826	1,281	14	30	6,432	40,37
12:Females CNT Dark phase vs. 16:Females CNT Dark phase	104,4	107,8	-3,429	0,5636	14	14	8,604	13,00
12:Females CNT Dark phase vs. 16:Females CRS Dark phase	104,4	97,58	6,816	1,102	14	32	8,745	33,98
12:Females CNT Dark phase vs. 19:Males CNT Dark phase	104,4	107,3	-2,872	1,572	14	26	2,583	37,07
12:Females CNT Dark phase vs. 19:Males CRS Dark phase	104,4	101,4	2,972	1,286	14	30	3,269	40,47
12:Females CNT Dark phase vs. 19:Females CNT Dark phase	104,4	109,5	-5,107	0,8224	14	14	8,783	13,00
12:Females CNT Dark phase vs. 19:Females CRS Dark phase	104,4	101,1	3,271	1,084	14	32	4,267	32,84
12:Females CRS Dark phase vs. 16:Males CNT Dark phase	96,82	107,3	-10,51	1,233	32	26	12,05	50,09
12:Females CRS Dark phase vs. 16:Males CRS Dark phase	96,82	98,58	-1,758	1,250	32	30	1,989	55,39
12:Females CRS Dark phase vs. 16:Females CNT Dark phase	96,82	107,8	-11,01	1,298	32	14	12,00	27,18
12:Females CRS Dark phase vs. 16:Females CRS Dark phase	96,82	97,58	-0,7677	0,3590	32	32	3,024	31,00
12:Females CRS Dark phase vs. 19:Males CNT Dark phase	96,82	107,3	-10,46	1,547	32	26	9,556	40,29
12:Females CRS Dark phase vs. 19:Males CRS Dark phase	96,82	101,4	-4,612	1,255	32	30	5,197	55,22
12:Females CRS Dark phase vs. 19:Females CNT Dark phase	96,82	109,5	-12,69	1,553	32	14	11,56	21,67
12:Females CRS Dark phase vs. 19:Females CRS Dark phase	96,82	101,1	-4,312	0,4447	32	32	13,71	31,00
16:Males CNT Dark phase vs. 16:Males CRS Dark phase	107,3	98,58	8,748	1,385	26	30	8,930	53,85
16:Males CNT Dark phase vs. 16:Females CNT Dark phase	107,3	107,8	-0,5066	1,429	26	14	0,5015	32,36
16:Males CNT Dark phase vs. 16:Females CRS Dark phase	107,3	97,58	9,738	1,222	26	32	11,27	49,38
16:Males CNT Dark phase vs. 19:Males CNT Dark phase	107,3	107,3	0,05040	1,119	26	26	0,06371	25,00
16:Males CNT Dark phase vs. 19:Males CRS Dark phase	107,3	101,4	5,894	1,390	26	30	5,997	53,89
16:Males CNT Dark phase vs. 19:Females CNT Dark phase	107,3	109,5	-2,185	1,664	26	14	1,857	26,18
16:Males CNT Dark phase vs. 19:Females CRS Dark phase	107,3	101,1	6,193	1,206	26	32	7,263	48,26
16:Males CRS Dark phase vs. 16:Females CNT Dark phase	98,58	107,8	-9,254	1,443	30	14	9,068	34,26
16:Males CRS Dark phase vs. 16:Females CRS Dark phase	98,58	97,58	0,9902	1,239	30	32	1,130	54,72
16:Males CRS Dark phase vs. 19:Males CNT Dark phase	98,58	107,3	-8,697	1,671	30	26	7,360	47,41
16:Males CRS Dark phase vs. 19:Males CRS Dark phase	98,58	101,4	-2,854	0,3757	30	30	10,74	29,00
16:Males CRS Dark phase vs. 19:Females CNT Dark phase	98,58	109,5	-10,93	1,676	30	14	9,223	27,17
16:Males CRS Dark phase vs. 19:Females CRS Dark phase	98,58	101,1	-2,554	1,223	30	32	2,953	53,64
16:Females CNT Dark phase vs. 16:Females CRS Dark phase	107,8	97,58	10,24	1,287	14	32	11,25	26,56
16:Females CNT Dark phase vs. 19:Males CNT Dark phase	107,8	107,3	0,5570	1,707	14	26	0,4614	37,78
16:Females CNT Dark phase vs. 19:Males CRS Dark phase	107,8	101,4	6,400	1,447	14	30	6,253	34,44
16:Females CNT Dark phase vs. 19:Females CNT Dark phase	107,8	109,5	-1,679	0,7842	14	14	3,027	13,00
16:Females CNT Dark phase vs. 19:Females CRS Dark phase	107,8	101,1	6,700	1,272	14	32	7,449	25,65
16:Females CRS Dark phase vs. 19:Males CNT Dark phase	97,58	107,3	-9,687	1,538	32	26	8,905	39,66
16:Females CRS Dark phase vs. 19:Males CRS Dark phase	97,58	101,4	-3,844	1,244	32	30	4,370	54,54
16:Females CRS Dark phase vs. 19:Females CNT Dark phase	97,58	109,5	-11,92	1,544	32	14	10,92	21,26
16:Females CRS Dark phase vs. 19:Females CRS Dark phase	97,58	101,1	-3,545	0,4580	32	32	10,95	31,00
19:Males CNT Dark phase vs. 19:Males CRS Dark phase	107,3	101,4	5,843	1,675	26	30	4,934	47,59

19:Males CNT Dark phase vs. 19:Females CNT Dark phase	107,3	109,5	-2,236	1,908	26	14	1,657	34,10
19:Males CNT Dark phase vs. 19:Females CRS Dark phase	107,3	101,1	6,143	1,526	26	32	5,694	38,72
19:Males CRS Dark phase vs. 19:Females CNT Dark phase	101,4	109,5	-8,079	1,680	30	14	6,800	27,33
19:Males CRS Dark phase vs. 19:Females CRS Dark phase	101,4	101,1	0,2994	1,228	30	32	0,3447	53,46
19:Females CNT Dark phase vs. 19:Females CRS Dark phase	109,5	101,1	8,378	1,531	14	32	7,737	20,67

Statistics of Figure 1c

Table Analyzed	% Weight gain - Day 12-19				
Three-way ANOVA	Ordinary				
Alpha	0,05				
Source of Variation	% of total variation	P value	P value summary	Significant?	
sex	0,9923	0,0332	*	Yes	
light	1,548	0,0080	**	Yes	
stress	40,30	<0,0001	****	Yes	
sex x light	0,4332	0,1583	ns	No	
sex x stress	0,3369	0,2132	ns	No	
light x stress	0,02721	0,7231	ns	No	
sex x light x stress	1,315	0,0144	*	Yes	
ANOVA table	SS (Type III)	DF	MS	F (DFn, DFd)	P value
stress	100,6	1	100,6	F (1, 238) = 4,588	P=0,0332
light	157,0	1	157,0	F (1, 238) = 7,158	P=0,0080
sex	4086	1	4086	F (1, 238) = 186,3	P<0,0001
stress x light	43,92	1	43,92	F (1, 238) = 2,003	P=0,1583
stress x sex	34,16	1	34,16	F (1, 238) = 1,558	P=0,2132
light x sex	2,759	1	2,759	F (1, 238) = 0,1258	P=0,7231
stress x light x sex	133,3	1	133,3	F (1, 238) = 6,081	P=0,0144
Residual	5219	238	21,93		

Compare each cell mean with every other cell mean								
Number of families	1							
Number of comparisons per family	28							
Alpha	0,05							
Tukey's multiple comparisons test	Predicted (LS) mean diff,	95,00% CI of diff,	Below threshold?	Summary	Adjusted P Value			
Males:Light phase CNT vs. Males:Light phase CRS	6,523	3,166 to 9,880	Yes	****	<0,0001			

Males:Light phase CNT vs. Males:Dark phase CNT	1,255	-2,645 to 5,155	No	ns	0,9764				
Males:Light phase CNT vs. Males:Dark phase CRS	10,45	6,691 to 14,22	Yes	****	<0,0001				
Males:Light phase CNT vs. Females:Light phase CNT	-0,09943	-3,927 to 3,728	No	ns	>0,9999				
Males:Light phase CNT vs. Females:Light phase CRS	11,13	7,545 to 14,72	Yes	****	<0,0001				
Males:Light phase CNT vs. Females:Dark phase CNT	2,487	-2,200 to 7,175	No	ns	0,7361				
Males:Light phase CNT vs. Females:Dark phase CRS	10,14	6,409 to 13,88	Yes	****	<0,0001				
Males:Light phase CRS vs. Males:Dark phase CNT	-5,268	-8,708 to -1,828	Yes	***	0,0001				
Males:Light phase CRS vs. Males:Dark phase CRS	3,931	0,6480 to 7,215	Yes	**	0,0074				
Males:Light phase CRS vs. Females:Light phase CNT	-6,622	-9,979 to -3,265	Yes	****	<0,0001				
Males:Light phase CRS vs. Females:Light phase CRS	4,609	1,529 to 7,689	Yes	***	0,0002				
Males:Light phase CRS vs. Females:Dark phase CNT	-4,035	-8,347 to 0,2766	No	ns	0,0852				
Males:Light phase CRS vs. Females:Dark phase CRS	3,620	0,3705 to 6,869	Yes	*	0,0173				
Males:Dark phase CNT vs. Males:Dark phase CRS	9,199	5,362 to 13,04	Yes	****	<0,0001				
Males:Dark phase CNT vs. Females:Light phase CNT	-1,354	-5,254 to 2,546	No	ns	0,9639				
Males:Dark phase CNT vs. Females:Light phase CRS	9,877	6,212 to 13,54	Yes	****	<0,0001				
Males:Dark phase CNT vs. Females:Dark phase CNT	1,233	-3,515 to 5,980	No	ns	0,9933				
Males:Dark phase CNT vs. Females:Dark phase CRS	8,888	5,080 to 12,70	Yes	****	<0,0001				
Males:Dark phase CRS vs. Females:Light phase CNT	-10,55	-14,32 to -6,790	Yes	****	<0,0001				
Males:Dark phase CRS vs. Females:Light phase CRS	0,6777	-2,841 to 4,196	No	ns	0,9990				
Males:Dark phase CRS vs. Females:Dark phase CNT	-7,967	-12,60 to -3,331	Yes	****	<0,0001				
Males:Dark phase CRS vs. Females:Dark phase CRS	-0,3113	-3,979 to 3,356	No	ns	>0,9999				
Females:Light phase CNT vs. Females:Light phase CRS	11,23	7,644 to 14,82	Yes	****	<0,0001				
Females:Light phase CNT vs. Females:Dark phase CNT	2,587	-2,101 to 7,274	No	ns	0,6953				
Females:Light phase CNT vs. Females:Dark phase CRS	10,24	6,509 to 13,98	Yes	****	<0,0001				
Females:Light phase CRS vs. Females:Dark phase CNT	-8,644	-13,14 to -4,151	Yes	****	<0,0001				
Females:Light phase CRS vs. Females:Dark phase CRS	-0,9891	-4,476 to 2,498	No	ns	0,9886				
Females:Dark phase CNT vs. Females:Dark phase CRS	7,655	3,044 to 12,27	Yes	****	<0,0001				
Test details	Predicted (LS) mean 1	Predicted (LS) mean 2	Predicted (LS) mean diff,	SE of diff,	N1	N2	q	DF	
Males:Light phase CNT vs. Males:Light phase CRS	107,2	100,7	6,523	1,098	28	52	8,404	238,0	
Males:Light phase CNT vs. Males:Dark phase CNT	107,2	106,0	1,255	1,275	28	26	1,391	238,0	
Males:Light phase CNT vs. Males:Dark phase CRS	107,2	96,78	10,45	1,231	28	30	12,01	238,0	
Males:Light phase CNT vs. Females:Light phase CNT	107,2	107,3	-0,09943	1,252	28	28	0,1123	238,0	
Males:Light phase CNT vs. Females:Light phase CRS	107,2	96,10	11,13	1,173	28	37	13,42	238,0	
Males:Light phase CNT vs. Females:Dark phase CNT	107,2	104,7	2,487	1,533	28	14	2,295	238,0	
Males:Light phase CNT vs. Females:Dark phase CRS	107,2	97,09	10,14	1,221	28	31	11,75	238,0	
Males:Light phase CRS vs. Males:Dark phase CNT	100,7	106,0	-5,268	1,125	52	26	6,624	238,0	
Males:Light phase CRS vs. Males:Dark phase CRS	100,7	96,78	3,931	1,074	52	30	5,178	238,0	
Males:Light phase CRS vs. Females:Light phase CNT	100,7	107,3	-6,622	1,098	52	28	8,532	238,0	
Males:Light phase CRS vs. Females:Light phase CRS	100,7	96,10	4,609	1,007	52	37	6,472	238,0	
Males:Light phase CRS vs. Females:Dark phase CNT	100,7	104,7	-4,035	1,410	52	14	4,047	238,0	
Males:Light phase CRS vs. Females:Dark phase CRS	100,7	97,09	3,620	1,063	52	31	4,818	238,0	
Males:Dark phase CNT vs. Males:Dark phase CRS	106,0	96,78	9,199	1,255	26	30	10,37	238,0	
Males:Dark phase CNT vs. Females:Light phase CNT	106,0	107,3	-1,354	1,275	26	28	1,502	238,0	
Males:Dark phase CNT vs. Females:Light phase CRS	106,0	96,10	9,877	1,198	26	37	11,66	238,0	
Males:Dark phase CNT vs. Females:Dark phase CNT	106,0	104,7	1,233	1,552	26	14	1,123	238,0	
Males:Dark phase CNT vs. Females:Dark phase CRS	106,0	97,09	8,888	1,245	26	31	10,09	238,0	
Males:Dark phase CRS vs. Females:Light phase CNT	96,78	107,3	-10,55	1,231	30	28	12,13	238,0	
Males:Dark phase CRS vs. Females:Light phase CRS	96,78	96,10	0,6777	1,150	30	37	0,8331	238,0	
Males:Dark phase CRS vs. Females:Dark phase CNT	96,78	104,7	-7,967	1,516	30	14	7,433	238,0	

Males:Dark phase CRS vs. Females:Dark phase CRS	96,78	97,09	-0,3113	1,199	30	31	0,3671	238,0
Females:Light phase CNT vs. Females:Light phase CRS	107,3	96,10	11,23	1,173	28	37	13,54	238,0
Females:Light phase CNT vs. Females:Dark phase CNT	107,3	104,7	2,587	1,533	28	14	2,387	238,0
Females:Light phase CNT vs. Females:Dark phase CRS	107,3	97,09	10,24	1,221	28	31	11,86	238,0
Females:Light phase CRS vs. Females:Dark phase CNT	96,10	104,7	-8,644	1,469	37	14	8,320	238,0
Females:Light phase CRS vs. Females:Dark phase CRS	96,10	97,09	-0,9891	1,140	37	31	1,227	238,0
Females:Dark phase CNT vs. Females:Dark phase CRS	104,7	97,09	7,655	1,508	14	31	7,180	238,0
