# Supplementary material for: Sex and Circadian Rhythm Dependent Behavioral Effects of Chronic Stress in Mice and Modulation of Clock Genes in the Prefrontal Cortex
Source: Int J Mol Sci. 2025 Jul 3;26(13):6410. doi: 10.3390/ijms26136410 (PMC12250008; doi:10.3390/ijms26136410)
Supplement: Supplementary file 1 [file ijms-26-06410-s001.zip › Table S2.pdf]

Supplementary Table S2

Statistics of Figure 2a

Table Analyzed	OFT time in the center; Grouped: Three-way ANOVA (2x2x2)				
Three-way ANOVA	Ordinary				
Alpha	0,05				
Source of Variation	% of total variation	P value	P value summary	Significant?	
sex	7,177	<0,0001	****	Yes	
light	1,952	0,0188	*	Yes	
stress	8,341	<0,0001	****	Yes	
sex x light	11,82	<0,0001	****	Yes	
sex x stress	0,05056	0,7035	ns	No	
light x stress	0,009771	0,8671	ns	No	
sex x light x stress	1,614	0,0324	*	Yes	
ANOVA table	SS (Type III)	DF	MS	F (DFn, DFd)	P value
stress	1847	1	1847	F (1, 210) = 20,62	P<0,0001
light	502,3	1	502,3	F (1, 210) = 5,609	P=0,0188
sex	2146	1	2146	F (1, 210) = 23,97	P<0,0001
stress x light	3041	1	3041	F (1, 210) = 33,96	P<0,0001
stress x sex	13,01	1	13,01	F (1, 210) = 0,1453	P=0,7035
light x sex	2,514	1	2,514	F (1, 210) = 0,02808	P=0,8671
stress x light x sex	415,4	1	415,4	F (1, 210) = 4,639	P=0,0324
Residual	18805	210	89,55		

Compare each cell mean with every other cell mean								
Number of families	1							
Number of comparisons per family	28							
Alpha	0,05							
Tukey's multiple comparisons test	Predicted (LS) mean diff,	95,00% CI of diff,	Below threshold?	Summary	Adjusted P Value			
Males:Light phase CNT vs. Males:Light phase CRS	8,600	-0,3807 to 17,58	No	ns	0,0714			
Males:Light phase CNT vs. Males:Dark phase CNT	13,47	3,854 to 23,09	Yes	***	0,0007			
Males:Light phase CNT vs. Males:Dark phase CRS	16,81	7,544 to 26,08	Yes	****	<0,0001			
Males:Light phase CNT vs. Females:Light phase CNT	16,06	6,667 to 25,46	Yes	****	<0,0001			
Males:Light phase CNT vs. Females:Light phase CRS	19,97	11,03 to 28,91	Yes	****	<0,0001			
Males:Light phase CNT vs. Females:Dark phase CNT	8,415	-0,9155 to 17,75	No	ns	0,1107			
Males:Light phase CNT vs. Females:Dark phase CRS	18,47	9,304 to 27,63	Yes	****	<0,0001			
Males:Light phase CRS vs. Males:Dark phase CNT	4,870	-2,953 to 12,69	No	ns	0,5483			
Males:Light phase CRS vs. Males:Dark phase CRS	8,214	0,8203 to 15,61	Yes	*	0,0179			
Males:Light phase CRS vs. Females:Light phase CNT	7,462	-0,08674 to 15,01	No	ns	0,0552			
Males:Light phase CRS vs. Females:Light phase CRS	11,37	4,395 to 18,35	Yes	****	<0,0001			
Males:Light phase CRS vs. Females:Dark phase CNT	-0,1852	-7,654 to 7,283	No	ns	>0,9999			
Males:Light phase CRS vs. Females:Dark phase CRS	9,867	2,609 to 17,12	Yes	**	0,0012			

Males:Dark phase CNT vs. Males:Dark phase CRS	3,345	-4,809 to 11,50	No	ns	0,9137				
Males:Dark phase CNT vs. Females:Light phase CNT	2,592	-5,702 to 10,89	No	ns	0,9798				
Males:Dark phase CNT vs. Females:Light phase CRS	6,502	-1,275 to 14,28	No	ns	0,1769				
Males:Dark phase CNT vs. Females:Dark phase CNT	-5,055	-13,28 to 3,166	No	ns	0,5645				
Males:Dark phase CNT vs. Females:Dark phase CRS	4,997	-3,033 to 13,03	No	ns	0,5488				
Males:Dark phase CRS vs. Females:Light phase CNT	-0,7527	-8,644 to 7,138	No	ns	>0,9999				
Males:Dark phase CRS vs. Females:Light phase CRS	3,157	-4,189 to 10,50	No	ns	0,8920				
Males:Dark phase CRS vs. Females:Dark phase CNT	-8,399	-16,21 to -0,5847	Yes	*	0,0254				
Males:Dark phase CRS vs. Females:Dark phase CRS	1,652	-5,961 to 9,266	No	ns	0,9978				
Females:Light phase CNT vs. Females:Light phase CRS	3,910	-3,592 to 11,41	No	ns	0,7524				
Females:Light phase CNT vs. Females:Dark phase CNT	-7,647	-15,61 to 0,3143	No	ns	0,0697				
Females:Light phase CNT vs. Females:Dark phase CRS	2,405	-5,358 to 10,17	No	ns	0,9808				
Females:Light phase CRS vs. Females:Dark phase CNT	-11,56	-18,98 to -4,135	Yes	****	<0,0001				
Females:Light phase CRS vs. Females:Dark phase CRS	-1,505	-8,714 to 5,704	No	ns	0,9983				
Females:Dark phase CNT vs. Females:Dark phase CRS	10,05	2,366 to 17,74	Yes	**	0,0022				

Test details	Predicted (LS) mean 1	Predicted (LS) mean 2	Predicted (LS) mean diff,	SE of diff,	N1	N2	q	DF
Males:Light phase CNT vs. Males:Light phase CRS	31,60	23,00	8,600	2,933	15	34	4,146	210,0
Males:Light phase CNT vs. Males:Dark phase CNT	31,60	18,13	13,47	3,141	15	23	6,065	210,0
Males:Light phase CNT vs. Males:Dark phase CRS	31,60	14,79	16,81	3,028	15	28	7,853	210,0
Males:Light phase CNT vs. Females:Light phase CNT	31,60	15,54	16,06	3,068	15	26	7,403	210,0
Males:Light phase CNT vs. Females:Light phase CRS	31,60	11,63	19,97	2,920	15	35	9,671	210,0
Males:Light phase CNT vs. Females:Dark phase CNT	31,60	23,19	8,415	3,047	15	27	3,905	210,0
Males:Light phase CNT vs. Females:Dark phase CRS	31,60	13,13	18,47	2,992	15	30	8,727	210,0
Males:Light phase CRS vs. Males:Dark phase CNT	23,00	18,13	4,870	2,555	34	23	2,696	210,0
Males:Light phase CRS vs. Males:Dark phase CRS	23,00	14,79	8,214	2,415	34	28	4,810	210,0
Males:Light phase CRS vs. Females:Light phase CNT	23,00	15,54	7,462	2,465	34	26	4,280	210,0
Males:Light phase CRS vs. Females:Light phase CRS	23,00	11,63	11,37	2,279	34	35	7,057	210,0
Males:Light phase CRS vs. Females:Dark phase CNT	23,00	23,19	-0,1852	2,439	34	27	0,1074	210,0
Males:Light phase CRS vs. Females:Dark phase CRS	23,00	13,13	9,867	2,370	34	30	5,887	210,0
Males:Dark phase CNT vs. Males:Dark phase CRS	18,13	14,79	3,345	2,663	23	28	1,776	210,0
Males:Dark phase CNT vs. Females:Light phase CNT	18,13	15,54	2,592	2,709	23	26	1,353	210,0
Males:Dark phase CNT vs. Females:Light phase CRS	18,13	11,63	6,502	2,540	23	35	3,620	210,0
Males:Dark phase CNT vs. Females:Dark phase CNT	18,13	23,19	-5,055	2,685	23	27	2,662	210,0
Males:Dark phase CNT vs. Females:Dark phase CRS	18,13	13,13	4,997	2,623	23	30	2,695	210,0
Males:Dark phase CRS vs. Females:Light phase CNT	14,79	15,54	-0,7527	2,577	28	26	0,4131	210,0
Males:Dark phase CRS vs. Females:Light phase CRS	14,79	11,63	3,157	2,399	28	35	1,861	210,0
Males:Dark phase CRS vs. Females:Dark phase CNT	14,79	23,19	-8,399	2,552	28	27	4,654	210,0
Males:Dark phase CRS vs. Females:Dark phase CRS	14,79	13,13	1,652	2,487	28	30	0,9398	210,0
Females:Light phase CNT vs. Females:Light phase CRS	15,54	11,63	3,910	2,450	26	35	2,257	210,0
Females:Light phase CNT vs. Females:Dark phase CNT	15,54	23,19	-7,647	2,600	26	27	4,159	210,0
Females:Light phase CNT vs. Females:Dark phase CRS	15,54	13,13	2,405	2,536	26	30	1,341	210,0
Females:Light phase CRS vs. Females:Dark phase CNT	11,63	23,19	-11,56	2,424	35	27	6,743	210,0
Females:Light phase CRS vs. Females:Dark phase CRS	11,63	13,13	-1,505	2,354	35	30	0,9038	210,0
Females:Dark phase CNT vs. Females:Dark phase CRS	23,19	13,13	10,05	2,510	27	30	5,663	210,0

Statistics of Figure 2b

Table Analyzed	OFT N entries; Grouped: Three-way ANOVA (2x2x2)				
Three-way ANOVA	Ordinary				
Alpha	0,05				
Source of Variation	% of total variation	P value	P value summary	Significant?	
sex	0,5239	0,2289	ns	No	
light	1,841	0,0247	*	Yes	
stress	8,423	<0,0001	****	Yes	
sex x light	7,339	<0,0001	****	Yes	
sex x stress	1,484	0,0435	*	Yes	
light x stress	0,0009205	0,9597	ns	No	
sex x light x stress	0,9341	0,1086	ns	No	
ANOVA table	SS (Type III)	DF	MS	F (DFn, DFd)	P value
stress	26,08	1	26,08	F (1, 223) = 1,455	P=0,2289
light	91,65	1	91,65	F (1, 223) = 5,116	P=0,0247
sex	419,2	1	419,2	F (1, 223) = 23,40	P<0,0001
stress x light	365,3	1	365,3	F (1, 223) = 20,39	P<0,0001
stress x sex	73,84	1	73,84	F (1, 223) = 4,122	P=0,0435
light x sex	0,04582	1	0,04582	F (1, 223) = 0,002557	P=0,9597
stress x light x sex	46,49	1	46,49	F (1, 223) = 2,595	P=0,1086
Residual	3995	223	17,92		

Compare each cell mean with every other cell mean								
Number of families	1							
Number of comparisons per family	28							
Alpha	0,05							
Tukey's multiple comparisons test	Predicted (LS) mean diff,	95,00% CI of diff,	Below threshold?	Summary	Adjusted P Value			
Males:Light phase CNT vs. Males:Light phase CRS	2,510	-1,365 to 6,385	No	ns	0,4964			
Males:Light phase CNT vs. Males:Dark phase CNT	4,792	0,6115 to 8,972	Yes	*	0,0126			
Males:Light phase CNT vs. Males:Dark phase CRS	5,508	1,499 to 9,518	Yes	***	0,0010			
Males:Light phase CNT vs. Females:Light phase CNT	1,661	-2,398 to 5,720	No	ns	0,9151			
Males:Light phase CNT vs. Females:Light phase CRS	4,653	0,7613 to 8,544	Yes	**	0,0075			
Males:Light phase CNT vs. Females:Dark phase CNT	-0,5893	-4,648 to 3,470	No	ns	0,9998			
Males:Light phase CNT vs. Females:Dark phase CRS	4,313	0,3469 to 8,278	Yes	*	0,0225			
Males:Light phase CRS vs. Males:Dark phase CNT	2,282	-1,113 to 5,676	No	ns	0,4464			
Males:Light phase CRS vs. Males:Dark phase CRS	2,998	-0,1838 to 6,180	No	ns	0,0809			
Males:Light phase CRS vs. Females:Light phase CNT	-0,8494	-4,094 to 2,395	No	ns	0,9929			
Males:Light phase CRS vs. Females:Light phase CRS	2,143	-0,8894 to 5,175	No	ns	0,3787			
Males:Light phase CRS vs. Females:Dark phase CNT	-3,099	-6,344 to 0,1448	No	ns	0,0728			
Males:Light phase CRS vs. Females:Dark phase CRS	1,802	-1,324 to 4,929	No	ns	0,6451			
Males:Dark phase CNT vs. Males:Dark phase CRS	0,7167	-2,830 to 4,264	No	ns	0,9986			
Males:Dark phase CNT vs. Females:Light phase CNT	-3,131	-6,734 to 0,4719	No	ns	0,1410			
Males:Dark phase CNT vs. Females:Light phase CRS	-0,1389	-3,552 to 3,274	No	ns	>0,9999			
Males:Dark phase CNT vs. Females:Dark phase CNT	-5,381	-8,984 to -1,778	Yes	***	0,0002			

Males:Dark phase CNT vs. Females:Dark phase CRS	-0,4792	-3,977 to 3,018	No	ns	0,9999			
Males:Dark phase CRS vs. Females:Light phase CNT	-3,848	-7,251 to -0,4443	Yes	*	0,0147			
Males:Dark phase CRS vs. Females:Light phase CRS	-0,8556	-4,057 to 2,346	No	ns	0,9920			
Males:Dark phase CRS vs. Females:Dark phase CNT	-6,098	-9,501 to -2,694	Yes	****	<0,0001			
Males:Dark phase CRS vs. Females:Dark phase CRS	-1,196	-4,487 to 2,096	No	ns	0,9538			
Females:Light phase CNT vs. Females:Light phase CRS	2,992	-0,2715 to 6,256	No	ns	0,0988			
Females:Light phase CNT vs. Females:Dark phase CNT	-2,250	-5,711 to 1,211	No	ns	0,4917			
Females:Light phase CNT vs. Females:Dark phase CRS	2,652	-0,6998 to 6,003	No	ns	0,2364			
Females:Light phase CRS vs. Females:Dark phase CNT	-5,242	-8,506 to -1,979	Yes	****	<0,0001			
Females:Light phase CRS vs. Females:Dark phase CRS	-0,3403	-3,487 to 2,806	No	ns	>0,9999			
Females:Dark phase CNT vs. Females:Dark phase CRS	4,902	1,550 to 8,253	Yes	***	0,0003			
Test details	Predicted (LS) mean 1	Predicted (LS) mean 2	Predicted (LS) mean diff,	SE of diff,	N1	N2	q	DF
Males:Light phase CNT vs. Males:Light phase CRS	13,38	10,86	2,510	1,266	16	37	2,803	223,0
Males:Light phase CNT vs. Males:Dark phase CNT	13,38	8,583	4,792	1,366	16	24	4,960	223,0
Males:Light phase CNT vs. Males:Dark phase CRS	13,38	7,867	5,508	1,310	16	30	5,945	223,0
Males:Light phase CNT vs. Females:Light phase CNT	13,38	11,71	1,661	1,326	16	28	1,771	223,0
Males:Light phase CNT vs. Females:Light phase CRS	13,38	8,722	4,653	1,272	16	36	5,174	223,0
Males:Light phase CNT vs. Females:Dark phase CNT	13,38	13,96	-0,5893	1,326	16	28	0,6283	223,0
Males:Light phase CNT vs. Females:Dark phase CRS	13,38	9,063	4,313	1,296	16	32	4,706	223,0
Males:Light phase CRS vs. Males:Dark phase CNT	10,86	8,583	2,282	1,109	37	24	2,908	223,0
Males:Light phase CRS vs. Males:Dark phase CRS	10,86	7,867	2,998	1,040	37	30	4,077	223,0
Males:Light phase CRS vs. Females:Light phase CNT	10,86	11,71	-0,8494	1,060	37	28	1,133	223,0
Males:Light phase CRS vs. Females:Light phase CRS	10,86	8,722	2,143	0,9909	37	36	3,058	223,0
Males:Light phase CRS vs. Females:Dark phase CNT	10,86	13,96	-3,099	1,060	37	28	4,134	223,0
Males:Light phase CRS vs. Females:Dark phase CRS	10,86	9,063	1,802	1,022	37	32	2,495	223,0
Males:Dark phase CNT vs. Males:Dark phase CRS	8,583	7,867	0,7167	1,159	24	30	0,8744	223,0
Males:Dark phase CNT vs. Females:Light phase CNT	8,583	11,71	-3,131	1,177	24	28	3,761	223,0
Males:Dark phase CNT vs. Females:Light phase CRS	8,583	8,722	-0,1389	1,115	24	36	0,1761	223,0
Males:Dark phase CNT vs. Females:Dark phase CNT	8,583	13,96	-5,381	1,177	24	28	6,463	223,0
Males:Dark phase CNT vs. Females:Dark phase CRS	8,583	9,063	-0,4792	1,143	24	32	0,5929	223,0
Males:Dark phase CRS vs. Females:Light phase CNT	7,867	11,71	-3,848	1,112	30	28	4,892	223,0
Males:Dark phase CRS vs. Females:Light phase CRS	7,867	8,722	-0,8556	1,046	30	36	1,156	223,0
Males:Dark phase CRS vs. Females:Dark phase CNT	7,867	13,96	-6,098	1,112	30	28	7,753	223,0
Males:Dark phase CRS vs. Females:Dark phase CRS	7,867	9,063	-1,196	1,076	30	32	1,572	223,0
Females:Light phase CNT vs. Females:Light phase CRS	11,71	8,722	2,992	1,067	28	36	3,967	223,0
Females:Light phase CNT vs. Females:Dark phase CNT	11,71	13,96	-2,250	1,131	28	28	2,813	223,0
Females:Light phase CNT vs. Females:Dark phase CRS	11,71	9,063	2,652	1,095	28	32	3,424	223,0
Females:Light phase CRS vs. Females:Dark phase CNT	8,722	13,96	-5,242	1,067	36	28	6,951	223,0
Females:Light phase CRS vs. Females:Dark phase CRS	8,722	9,063	-0,3403	1,028	36	32	0,4680	223,0
Females:Dark phase CNT vs. Females:Dark phase CRS	13,96	9,063	4,902	1,095	28	32	6,329	223,0

Statistics of Figure 2c

Table Analyzed	TST; Grouped: Three-way ANOVA (2x2x2)				
Three-way ANOVA	Ordinary				
Alpha	0,05				
Source of Variation	% of total variation	P value	P value summary	Significant?	
sex	0,2885	0,4506	ns	No	
light	2,410	0,0302	*	Yes	
stress	0,6616	0,2537	ns	No	
sex x light	3,863	0,0063	**	Yes	
sex x stress	0,01557	0,8608	ns	No	
light x stress	0,1232	0,6219	ns	No	
sex x light x stress	1,569	0,0796	ns	No	
ANOVA table	SS (Type III)	DF	MS	F (DFn, DFd)	P value
sex	632,6	1	632,6	F (1, 178) = 0,5717	P=0,4506
light	5286	1	5286	F (1, 178) = 4,777	P=0,0302
stress	1451	1	1451	F (1, 178) = 1,311	P=0,2537
sex x light	8471	1	8471	F (1, 178) = 7,655	P=0,0063
sex x stress	34,13	1	34,13	F (1, 178) = 0,03085	P=0,8608
light x stress	270,1	1	270,1	F (1, 178) = 0,2441	P=0,6219
sex x light x stress	3440	1	3440	F (1, 178) = 3,109	P=0,0796
Residual	196968	178	1107		

Compare each cell mean with every other cell mean								
Number of families	1							
Number of comparisons per family	28							
Alpha	0,05							
Tukey's multiple comparisons test	Predicted (LS) mean diff,	95,00% CI of diff,	Below threshold?	Summary	Adjusted P Value			
Males:Light phase CNT vs. Males:Light phase CRS	-11,45	-37,43 to 14,53	No	ns	0,8774			
Males:Light phase CNT vs. Males:Dark phase CNT	-31,82	-60,42 to -3,219	Yes	*	0,0178			
Males:Light phase CNT vs. Males:Dark phase CRS	-30,29	-58,56 to -2,010	Yes	*	0,0264			
Males:Light phase CNT vs. Females:Light phase CNT	-26,14	-55,87 to 3,599	No	ns	0,1306			
Males:Light phase CNT vs. Females:Light phase CRS	-21,35	-48,30 to 5,608	No	ns	0,2338			
Males:Light phase CNT vs. Females:Dark phase CNT	-11,62	-46,59 to 23,35	No	ns	0,9710			
Males:Light phase CNT vs. Females:Dark phase CRS	-37,30	-69,76 to -4,845	Yes	*	0,0123			
Males:Light phase CRS vs. Males:Dark phase CNT	-20,37	-47,58 to 6,841	No	ns	0,3015			
Males:Light phase CRS vs. Males:Dark phase CRS	-18,84	-45,71 to 8,034	No	ns	0,3870			
Males:Light phase CRS vs. Females:Light phase CNT	-14,69	-43,09 to 13,72	No	ns	0,7579			
Males:Light phase CRS vs. Females:Light phase CRS	-9,899	-35,38 to 15,58	No	ns	0,9334			
Males:Light phase CRS vs. Females:Dark phase CNT	-0,1716	-34,02 to 33,67	No	ns	>0,9999			
Males:Light phase CRS vs. Females:Dark phase CRS	-25,85	-57,10 to 5,387	No	ns	0,1863			
Males:Dark phase CNT vs. Males:Dark phase CRS	1,533	-27,88 to 30,94	No	ns	>0,9999			
Males:Dark phase CNT vs. Females:Light phase CNT	5,683	-25,13 to 36,50	No	ns	0,9992			
Males:Dark phase CNT vs. Females:Light phase CRS	10,47	-17,67 to 38,62	No	ns	0,9466			
Males:Dark phase CNT vs. Females:Dark phase CNT	20,20	-15,69 to 56,09	No	ns	0,6700			
Males:Dark phase CNT vs. Females:Dark phase CRS	-5,484	-38,94 to 27,97	No	ns	0,9996			

Males:Dark phase CRS vs. Females:Light phase CNT	4,150	-26,37 to 34,67	No	ns	0,9999			
Males:Dark phase CRS vs. Females:Light phase CRS	8,940	-18,87 to 36,75	No	ns	0,9759			
Males:Dark phase CRS vs. Females:Dark phase CNT	18,67	-16,97 to 54,30	No	ns	0,7456			
Males:Dark phase CRS vs. Females:Dark phase CRS	-7,017	-40,19 to 26,16	No	ns	0,9981			
Females:Light phase CNT vs. Females:Light phase CRS	4,790	-24,51 to 34,09	No	ns	0,9996			
Females:Light phase CNT vs. Females:Dark phase CNT	14,52	-22,29 to 51,32	No	ns	0,9281			
Females:Light phase CNT vs. Females:Dark phase CRS	-11,17	-45,59 to 23,26	No	ns	0,9746			
Females:Light phase CRS vs. Females:Dark phase CNT	9,727	-24,87 to 44,32	No	ns	0,9889			
Females:Light phase CRS vs. Females:Dark phase CRS	-15,96	-48,01 to 16,10	No	ns	0,7919			
Females:Dark phase CNT vs. Females:Dark phase CRS	-25,68	-64,72 to 13,35	No	ns	0,4726			

Test details	Predicted (LS) mean 1	Predicted (LS) mean 2	Predicted (LS) mean diff,	SE of diff,	N1	N2	q	DF
Males:Light phase CNT vs. Males:Light phase CRS	142,0	153,4	-11,45	8,469	27	34	1,912	176,0
Males:Light phase CNT vs. Males:Dark phase CNT	142,0	173,8	-31,82	9,323	27	23	4,827	176,0
Males:Light phase CNT vs. Males:Dark phase CRS	142,0	172,3	-30,29	9,217	27	24	4,647	176,0
Males:Light phase CNT vs. Females:Light phase CNT	142,0	168,1	-26,14	9,693	27	20	3,813	176,0
Males:Light phase CNT vs. Females:Light phase CRS	142,0	163,3	-21,35	8,787	27	29	3,436	176,0
Males:Light phase CNT vs. Females:Dark phase CNT	142,0	153,6	-11,62	11,40	27	12	1,442	176,0
Males:Light phase CNT vs. Females:Dark phase CRS	142,0	179,3	-37,30	10,58	27	15	4,986	176,0
Males:Light phase CRS vs. Males:Dark phase CNT	153,4	173,8	-20,37	8,870	34	23	3,248	176,0
Males:Light phase CRS vs. Males:Dark phase CRS	153,4	172,3	-18,84	8,760	34	24	3,041	176,0
Males:Light phase CRS vs. Females:Light phase CNT	153,4	168,1	-14,69	9,259	34	20	2,244	176,0
Males:Light phase CRS vs. Females:Light phase CRS	153,4	163,3	-9,899	8,305	34	29	1,686	176,0
Males:Light phase CRS vs. Females:Dark phase CNT	153,4	153,6	-0,1716	11,03	34	12	0,02199	176,0
Males:Light phase CRS vs. Females:Dark phase CRS	153,4	179,3	-25,85	10,18	34	15	3,590	176,0
Males:Dark phase CNT vs. Males:Dark phase CRS	173,8	172,3	1,533	9,587	23	24	0,2261	176,0
Males:Dark phase CNT vs. Females:Light phase CNT	173,8	168,1	5,683	10,05	23	20	0,8000	176,0
Males:Dark phase CNT vs. Females:Light phase CRS	173,8	163,3	10,47	9,174	23	29	1,614	176,0
Males:Dark phase CNT vs. Females:Dark phase CNT	173,8	153,6	20,20	11,70	23	12	2,442	176,0
Males:Dark phase CNT vs. Females:Dark phase CRS	173,8	179,3	-5,484	10,90	23	15	0,7113	176,0
Males:Dark phase CRS vs. Females:Light phase CNT	172,3	168,1	4,150	9,948	24	20	0,5900	176,0
Males:Dark phase CRS vs. Females:Light phase CRS	172,3	163,3	8,940	9,067	24	29	1,394	176,0
Males:Dark phase CRS vs. Females:Dark phase CNT	172,3	153,6	18,67	11,62	24	12	2,273	176,0
Males:Dark phase CRS vs. Females:Dark phase CRS	172,3	179,3	-7,017	10,81	24	15	0,9176	176,0
Females:Light phase CNT vs. Females:Light phase CRS	168,1	163,3	4,790	9,550	20	29	0,7093	176,0
Females:Light phase CNT vs. Females:Dark phase CNT	168,1	153,6	14,52	12,00	20	12	1,711	176,0
Females:Light phase CNT vs. Females:Dark phase CRS	168,1	179,3	-11,17	11,22	20	15	1,407	176,0
Females:Light phase CRS vs. Females:Dark phase CNT	163,3	153,6	9,727	11,28	29	12	1,220	176,0
Females:Light phase CRS vs. Females:Dark phase CRS	163,3	179,3	-15,96	10,45	29	15	2,160	176,0
Females:Dark phase CNT vs. Females:Dark phase CRS	153,6	179,3	-25,68	12,72	12	15	2,854	176,0

Statistics of Figure 2d

Table Analyzed	FST; Grouped: Three-way ANOVA (2x2x2)				
Three-way ANOVA	Ordinary				
Alpha	0,05				
Source of Variation	% of total variation	P value	P value summary	Significant?	
sex	10,26	<0,0001	****	Yes	
light	22,00	<0,0001	****	Yes	
stress	9,990	<0,0001	****	Yes	
sex x light	0,04257	0,7468	ns	No	
sex x stress	0,02065	0,8221	ns	No	
light x stress	1,104	0,1017	ns	No	
sex x light x stress	0,8955	0,1401	ns	No	
ANOVA table	SS (Type III)	DF	MS	F (DFn, DFd)	P value
stress	18848	1	18848	F (1, 147) = 25,22	P<0,0001
light	40403	1	40403	F (1, 147) = 54,07	P<0,0001
sex	18346	1	18346	F (1, 147) = 24,55	P<0,0001
stress x light	78,18	1	78,18	F (1, 147) = 0,1046	P=0,7468
stress x sex	37,91	1	37,91	F (1, 147) = 0,05073	P=0,8221
light x sex	2027	1	2027	F (1, 147) = 2,712	P=0,1017
stress x light x sex	1644	1	1644	F (1, 147) = 2,201	P=0,1401
Residual	109851	147	747,3		

Compare each cell mean with every other cell mean								
Number of families	1							
Number of comparisons per family	28							
Alpha	0,05							
Tukey's multiple comparisons test	Predicted (LS) mean diff,	95,00% CI of diff,	Below threshold?	Summary	Adjusted P Value			
Males:Light phase CNT vs. Males:Light phase CRS	-21,20	-43,02 to 0,6121	No	ns	0,0633			
Males:Light phase CNT vs. Males:Dark phase CNT	36,40	6,335 to 66,47	Yes	**	0,0066			
Males:Light phase CNT vs. Males:Dark phase CRS	13,68	-14,69 to 42,06	No	ns	0,8154			
Males:Light phase CNT vs. Females:Light phase CNT	-27,66	-53,69 to -1,633	Yes	*	0,0286			
Males:Light phase CNT vs. Females:Light phase CRS	-37,18	-61,98 to -12,38	Yes	***	0,0002			
Males:Light phase CNT vs. Females:Dark phase CNT	19,52	-6,510 to 45,54	No	ns	0,2975			
Males:Light phase CNT vs. Females:Dark phase CRS	-19,07	-45,10 to 6,955	No	ns	0,3267			
Males:Light phase CRS vs. Males:Dark phase CNT	57,61	28,34 to 86,87	Yes	****	<0,0001			
Males:Light phase CRS vs. Males:Dark phase CRS	34,89	7,359 to 62,41	Yes	**	0,0036			
Males:Light phase CRS vs. Females:Light phase CNT	-6,458	-31,55 to 18,64	No	ns	0,9933			
Males:Light phase CRS vs. Females:Light phase CRS	-15,98	-39,80 to 7,846	No	ns	0,4442			
Males:Light phase CRS vs. Females:Dark phase CNT	40,72	15,62 to 65,81	Yes	****	<0,0001			
Males:Light phase CRS vs. Females:Dark phase CRS	2,130	-22,97 to 27,23	No	ns	>0,9999			
Males:Dark phase CNT vs. Males:Dark phase CRS	-22,72	-57,16 to 11,72	No	ns	0,4663			
Males:Dark phase CNT vs. Females:Light phase CNT	-64,06	-96,59 to -31,54	Yes	****	<0,0001			
Males:Dark phase CNT vs. Females:Light phase CRS	-73,58	-105,1 to -42,03	Yes	****	<0,0001			
Males:Dark phase CNT vs. Females:Dark phase CNT	-16,89	-49,42 to 15,64	No	ns	0,7517			
Males:Dark phase CNT vs. Females:Dark phase CRS	-55,48	-88,00 to -22,95	Yes	****	<0,0001			
Males:Dark phase CRS vs. Females:Light phase CNT	-41,34	-72,32 to -10,37	Yes	**	0,0017			

Males:Dark phase CRS vs. Females:Light phase CRS	-50,86	-80,81 to -20,91	Yes	****	<0,0001			
Males:Dark phase CRS vs. Females:Dark phase CNT	5,833	-25,14 to 36,80	No	ns	0,9991			
Males:Dark phase CRS vs. Females:Dark phase CRS	-32,76	-63,73 to -1,784	Yes	*	0,0300			
Females:Light phase CNT vs. Females:Light phase CRS	-9,518	-37,25 to 18,21	No	ns	0,9647			
Females:Light phase CNT vs. Females:Dark phase CNT	47,18	18,34 to 76,01	Yes	****	<0,0001			
Females:Light phase CNT vs. Females:Dark phase CRS	8,588	-20,24 to 37,42	No	ns	0,9841			
Females:Light phase CRS vs. Females:Dark phase CNT	56,69	28,96 to 84,42	Yes	****	<0,0001			
Females:Light phase CRS vs. Females:Dark phase CRS	18,11	-9,625 to 45,84	No	ns	0,4803			
Females:Dark phase CNT vs. Females:Dark phase CRS	-38,59	-67,42 to -9,755	Yes	**	0,0016			

Test details	Predicted (LS) mean 1	Predicted (LS) mean 2	Predicted (LS) mean diff,	SE of diff,	N1	N2	q	DF
Males:Light phase CNT vs. Males:Light phase CRS	122,2	143,4	-21,20	7,094	27	33	4,227	147,0
Males:Light phase CNT vs. Males:Dark phase CNT	122,2	85,82	36,40	9,778	27	11	5,265	147,0
Males:Light phase CNT vs. Males:Dark phase CRS	122,2	108,5	13,68	9,228	27	13	2,097	147,0
Males:Light phase CNT vs. Females:Light phase CNT	122,2	149,9	-27,66	8,464	27	17	4,622	147,0
Males:Light phase CNT vs. Females:Light phase CRS	122,2	159,4	-37,18	8,065	27	20	6,519	147,0
Males:Light phase CNT vs. Females:Dark phase CNT	122,2	102,7	19,52	8,464	27	17	3,261	147,0
Males:Light phase CNT vs. Females:Dark phase CRS	122,2	141,3	-19,07	8,464	27	17	3,187	147,0
Males:Light phase CRS vs. Males:Dark phase CNT	143,4	85,82	57,61	9,517	33	11	8,560	147,0
Males:Light phase CRS vs. Males:Dark phase CRS	143,4	108,5	34,89	8,951	33	13	5,511	147,0
Males:Light phase CRS vs. Females:Light phase CNT	143,4	149,9	-6,458	8,161	33	17	1,119	147,0
Males:Light phase CRS vs. Females:Light phase CRS	143,4	159,4	-15,98	7,747	33	20	2,917	147,0
Males:Light phase CRS vs. Females:Dark phase CNT	143,4	102,7	40,72	8,161	33	17	7,056	147,0
Males:Light phase CRS vs. Females:Dark phase CRS	143,4	141,3	2,130	8,161	33	17	0,3691	147,0
Males:Dark phase CNT vs. Males:Dark phase CRS	85,82	108,5	-22,72	11,20	11	13	2,869	147,0
Males:Dark phase CNT vs. Females:Light phase CNT	85,82	149,9	-64,06	10,58	11	17	8,565	147,0
Males:Dark phase CNT vs. Females:Light phase CRS	85,82	159,4	-73,58	10,26	11	20	10,14	147,0
Males:Dark phase CNT vs. Females:Dark phase CNT	85,82	102,7	-16,89	10,58	11	17	2,258	147,0
Males:Dark phase CNT vs. Females:Dark phase CRS	85,82	141,3	-55,48	10,58	11	17	7,417	147,0
Males:Dark phase CRS vs. Females:Light phase CNT	108,5	149,9	-41,34	10,07	13	17	5,805	147,0
Males:Dark phase CRS vs. Females:Light phase CRS	108,5	159,4	-50,86	9,739	13	20	7,386	147,0
Males:Dark phase CRS vs. Females:Dark phase CNT	108,5	102,7	5,833	10,07	13	17	0,8190	147,0
Males:Dark phase CRS vs. Females:Dark phase CRS	108,5	141,3	-32,76	10,07	13	17	4,599	147,0
Females:Light phase CNT vs. Females:Light phase CRS	149,9	159,4	-9,518	9,018	17	20	1,493	147,0
Females:Light phase CNT vs. Females:Dark phase CNT	149,9	102,7	47,18	9,376	17	17	7,116	147,0
Females:Light phase CNT vs. Females:Dark phase CRS	149,9	141,3	8,588	9,376	17	17	1,295	147,0
Females:Light phase CRS vs. Females:Dark phase CNT	159,4	102,7	56,69	9,018	20	17	8,891	147,0
Females:Light phase CRS vs. Females:Dark phase CRS	159,4	141,3	18,11	9,018	20	17	2,839	147,0
Females:Dark phase CNT vs. Females:Dark phase CRS	102,7	141,3	-38,59	9,376	17	17	5,820	147,0
