# Supplementary material for: Sex and Circadian Rhythm Dependent Behavioral Effects of Chronic Stress in Mice and Modulation of Clock Genes in the Prefrontal Cortex
Source: Int J Mol Sci. 2025 Jul 3;26(13):6410. doi: 10.3390/ijms26136410 (PMC12250008; doi:10.3390/ijms26136410)
Supplement: Supplementary file 1 [file ijms-26-06410-s001.zip › Table S3.pdf]

Supplementary Table S3

Statistics of Figure 3a

Table Analyzed	SST time of grooming; Grouped: Three-way ANOVA (2x2x2)				
Three-way ANOVA	Ordinary				
Alpha	0,05				
Source of Variation	% of total variation	P value	P value summary	Significant?	
sex	0,06932	0,7282	ns	No	
light	0,1911	0,5641	ns	No	
stress	0,1627	0,5946	ns	No	
sex x light	6,315	0,0011	**	Yes	
sex x stress	6,666e-005	0,9914	ns	No	
light x stress	1,506	0,1068	ns	No	
sex x light x stress	0,01516	0,8709	ns	No	
ANOVA table	SS (Type III)	DF	MS	F (DFn, DFd)	P value
stress	47,93	1	47,93	F (1, 158) = 0,1212	P=0,7282
light	132,1	1	132,1	F (1, 158) = 0,3340	P=0,5641
sex	112,5	1	112,5	F (1, 158) = 0,2843	P=0,5946
stress x light	4366	1	4366	F (1, 158) = 11,04	P=0,0011
stress x sex	0,04609	1	0,04609	F (1, 158) = 0,0001165	P=0,9914
light x sex	1041	1	1041	F (1, 158) = 2,632	P=0,1068
stress x light x sex	10,48	1	10,48	F (1, 158) = 0,02650	P=0,8709
Residual	62505	158	395,6		

Compare each cell mean with every other cell mean								
Number of families	1							
Number of comparisons per family	28							
Alpha	0,05							
Tukey's multiple comparisons test	Predicted (LS) mean diff,	95,00% CI of diff,	Below threshold?	Summary	Adjusted P Value			
Males:Light phase CNT vs. Males:Light phase CRS	7,364	-9,233 to 23,96	No	ns	0,8725			
Males:Light phase CNT vs. Males:Dark phase CNT	-6,688	-26,58 to 13,20	No	ns	0,9687			
Males:Light phase CNT vs. Males:Dark phase CRS	-10,61	-29,84 to 8,617	No	ns	0,6903			
Males:Light phase CNT vs. Females:Light phase CNT	-11,06	-30,28 to 8,173	No	ns	0,6437			
Males:Light phase CNT vs. Females:Light phase CRS	-4,789	-23,73 to 14,15	No	ns	0,9941			
Males:Light phase CNT vs. Females:Dark phase CNT	2,235	-17,31 to 21,78	No	ns	>0,9999			
Males:Light phase CNT vs. Females:Dark phase CRS	-0,7273	-18,95 to 17,49	No	ns	>0,9999			
Males:Light phase CRS vs. Males:Dark phase CNT	-14,05	-32,66 to 4,562	No	ns	0,2900			
Males:Light phase CRS vs. Males:Dark phase CRS	-17,97	-35,88 to -0,07119	Yes	*	0,0483			
Males:Light phase CRS vs. Females:Light phase CNT	-18,42	-36,32 to -0,5156	Yes	*	0,0389			
Males:Light phase CRS vs. Females:Light phase CRS	-12,15	-29,75 to 5,443	No	ns	0,4057			
Males:Light phase CRS vs. Females:Dark phase CNT	-5,128	-23,37 to 13,11	No	ns	0,9888			
Males:Light phase CRS vs. Females:Dark phase CRS	-8,091	-24,91 to 8,727	No	ns	0,8181			
Males:Dark phase CNT vs. Males:Dark phase CRS	-3,924	-24,92 to 17,07	No	ns	0,9991			
Males:Dark phase CNT vs. Females:Light phase CNT	-4,368	-25,36 to 16,63	No	ns	0,9983			
Males:Dark phase CNT vs. Females:Light phase CRS	1,898	-18,83 to 22,63	No	ns	>0,9999			

Males:Dark phase CNT vs. Females:Dark phase CNT	8,923	-12,36 to 30,21	No	ns	0,9021			
Males:Dark phase CNT vs. Females:Dark phase CRS	5,960	-14,12 to 26,04	No	ns	0,9845			
Males:Dark phase CRS vs. Females:Light phase CNT	-0,4444	-20,81 to 19,92	No	ns	>0,9999			
Males:Dark phase CRS vs. Females:Light phase CRS	5,822	-14,28 to 25,92	No	ns	0,9866			
Males:Dark phase CRS vs. Females:Dark phase CNT	12,85	-7,818 to 33,51	No	ns	0,5463			
Males:Dark phase CRS vs. Females:Dark phase CRS	9,884	-9,535 to 29,30	No	ns	0,7711			
Females:Light phase CNT vs. Females:Light phase CRS	6,266	-13,83 to 26,36	No	ns	0,9795			
Females:Light phase CNT vs. Females:Dark phase CNT	13,29	-7,373 to 33,96	No	ns	0,5015			
Females:Light phase CNT vs. Females:Dark phase CRS	10,33	-9,091 to 29,75	No	ns	0,7291			
Females:Light phase CRS vs. Females:Dark phase CNT	7,025	-13,37 to 27,42	No	ns	0,9643			
Females:Light phase CRS vs. Females:Dark phase CRS	4,062	-15,07 to 23,20	No	ns	0,9980			
Females:Dark phase CNT vs. Females:Dark phase CRS	-2,963	-22,69 to 16,77	No	ns	0,9998			
Test details	Predicted (LS) mean 1	Predicted (LS) mean 2	Predicted (LS) mean diff,	SE of diff,	N1	N2	q	DF
Males:Light phase CNT vs. Males:Light phase CRS	47,00	39,64	7,364	5,403	23	33	1,928	158,0
Males:Light phase CNT vs. Males:Dark phase CNT	47,00	53,69	-6,688	6,475	23	16	1,461	158,0
Males:Light phase CNT vs. Males:Dark phase CRS	47,00	57,61	-10,61	6,259	23	18	2,397	158,0
Males:Light phase CNT vs. Females:Light phase CNT	47,00	58,06	-11,06	6,259	23	18	2,498	158,0
Males:Light phase CNT vs. Females:Light phase CRS	47,00	51,79	-4,789	6,166	23	19	1,098	158,0
Males:Light phase CNT vs. Females:Dark phase CNT	47,00	44,76	2,235	6,362	23	17	0,4969	158,0
Males:Light phase CNT vs. Females:Dark phase CRS	47,00	47,73	-0,7273	5,931	23	22	0,1734	158,0
Males:Light phase CRS vs. Males:Dark phase CNT	39,64	53,69	-14,05	6,059	33	16	3,280	158,0
Males:Light phase CRS vs. Males:Dark phase CRS	39,64	57,61	-17,97	5,828	33	18	4,362	158,0
Males:Light phase CRS vs. Females:Light phase CNT	39,64	58,06	-18,42	5,828	33	18	4,470	158,0
Males:Light phase CRS vs. Females:Light phase CRS	39,64	51,79	-12,15	5,728	33	19	3,001	158,0
Males:Light phase CRS vs. Females:Dark phase CNT	39,64	44,76	-5,128	5,938	33	17	1,221	158,0
Males:Light phase CRS vs. Females:Dark phase CRS	39,64	47,73	-8,091	5,474	33	22	2,090	158,0
Males:Dark phase CNT vs. Males:Dark phase CRS	53,69	57,61	-3,924	6,834	16	18	0,8120	158,0
Males:Dark phase CNT vs. Females:Light phase CNT	53,69	58,06	-4,368	6,834	16	18	0,9039	158,0
Males:Dark phase CNT vs. Females:Light phase CRS	53,69	51,79	1,898	6,749	16	19	0,3977	158,0
Males:Dark phase CNT vs. Females:Dark phase CNT	53,69	44,76	8,923	6,928	16	17	1,821	158,0
Males:Dark phase CNT vs. Females:Dark phase CRS	53,69	47,73	5,960	6,535	16	22	1,290	158,0
Males:Dark phase CRS vs. Females:Light phase CNT	57,61	58,06	-0,4444	6,630	18	18	0,09480	158,0
Males:Dark phase CRS vs. Females:Light phase CRS	57,61	51,79	5,822	6,542	18	19	1,258	158,0
Males:Dark phase CRS vs. Females:Dark phase CNT	57,61	44,76	12,85	6,727	18	17	2,701	158,0
Males:Dark phase CRS vs. Females:Dark phase CRS	57,61	47,73	9,884	6,321	18	22	2,211	158,0
Females:Light phase CNT vs. Females:Light phase CRS	58,06	51,79	6,266	6,542	18	19	1,355	158,0
Females:Light phase CNT vs. Females:Dark phase CNT	58,06	44,76	13,29	6,727	18	17	2,794	158,0
Females:Light phase CNT vs. Females:Dark phase CRS	58,06	47,73	10,33	6,321	18	22	2,311	158,0
Females:Light phase CRS vs. Females:Dark phase CNT	51,79	44,76	7,025	6,640	19	17	1,496	158,0
Females:Light phase CRS vs. Females:Dark phase CRS	51,79	47,73	4,062	6,229	19	22	0,9222	158,0
Females:Dark phase CNT vs. Females:Dark phase CRS	44,76	47,73	-2,963	6,423	17	22	0,6523	158,0

Statistics of Figure 3b

Table Analyzed	SST N of bouts; Grouped: Three-way ANOVA (2x2x2)				
Three-way ANOVA	Ordinary				
Alpha	0,05				
Source of Variation	% of total variation	P value	P value summary	Significant?	
sex	1,470	0,0962	ns	No	
light	5,593	0,0013	**	Yes	
stress	3,877	0,0073	**	Yes	
sex x light	0,5244	0,3191	ns	No	
sex x stress	0,2408	0,4992	ns	No	
light x stress	0,2063	0,5317	ns	No	
sex x light x stress	0,3534	0,4132	ns	No	
ANOVA table	SS (Type III)	DF	MS	F (DFn, DFd)	P value
stress	75,39	1	75,39	F (1, 165) = 2,799	P=0,0962
light	286,9	1	286,9	F (1, 165) = 10,65	P=0,0013
sex	198,9	1	198,9	F (1, 165) = 7,384	P=0,0073
stress x light	26,90	1	26,90	F (1, 165) = 0,9987	P=0,3191
stress x sex	12,35	1	12,35	F (1, 165) = 0,4587	P=0,4992
light x sex	10,58	1	10,58	F (1, 165) = 0,3929	P=0,5317
stress x light x sex	18,13	1	18,13	F (1, 165) = 0,6731	P=0,4132
Residual	4444	165	26,93		

Compare each cell mean with every other cell mean								
Number of families	1							
Number of comparisons per family	28							
Alpha	0,05							
Tukey's multiple comparisons test	Predicted (LS) mean diff,	95,00% CI of diff,	Below threshold?	Summary	Adjusted P Value			
Males:Light phase CNT vs. Males:Light phase CRS	3,941	-0,2070 to 8,089	No	ns	0,0757			
Males:Light phase CNT vs. Males:Dark phase CNT	3,021	-1,988 to 8,030	No	ns	0,5858			
Males:Light phase CNT vs. Males:Dark phase CRS	4,606	-0,2430 to 9,456	No	ns	0,0759			
Males:Light phase CNT vs. Females:Light phase CNT	1,768	-3,334 to 6,869	No	ns	0,9633			
Males:Light phase CNT vs. Females:Light phase CRS	3,270	-1,446 to 7,987	No	ns	0,4008			
Males:Light phase CNT vs. Females:Dark phase CNT	5,080	0,07119 to 10,09	Yes	*	0,0442			
Males:Light phase CNT vs. Females:Dark phase CRS	6,898	2,240 to 11,56	Yes	***	0,0003			
Males:Light phase CRS vs. Males:Dark phase CNT	-0,9199	-5,609 to 3,769	No	ns	0,9988			
Males:Light phase CRS vs. Males:Dark phase CRS	0,6652	-3,853 to 5,183	No	ns	0,9998			
Males:Light phase CRS vs. Females:Light phase CNT	-2,174	-6,961 to 2,614	No	ns	0,8589			
Males:Light phase CRS vs. Females:Light phase CRS	-0,6706	-5,046 to 3,704	No	ns	0,9998			
Males:Light phase CRS vs. Females:Dark phase CNT	1,139	-3,550 to 5,828	No	ns	0,9954			
Males:Light phase CRS vs. Females:Dark phase CRS	2,957	-1,355 to 7,269	No	ns	0,4157			
Males:Dark phase CNT vs. Males:Dark phase CRS	1,585	-3,734 to 6,904	No	ns	0,9843			
Males:Dark phase CNT vs. Females:Light phase CNT	-1,254	-6,803 to 4,296	No	ns	0,9971			
Males:Dark phase CNT vs. Females:Light phase CRS	0,2493	-4,949 to 5,448	No	ns	>0,9999			

Males:Dark phase CNT vs. Females:Dark phase CNT	2,059	-3,406 to 7,524	No	ns	0,9428				
Males:Dark phase CNT vs. Females:Dark phase CRS	3,877	-1,268 to 9,022	No	ns	0,2926				
Males:Dark phase CRS vs. Females:Light phase CNT	-2,839	-8,245 to 2,568	No	ns	0,7424				
Males:Dark phase CRS vs. Females:Light phase CRS	-1,336	-6,381 to 3,709	No	ns	0,9922				
Males:Dark phase CRS vs. Females:Dark phase CNT	0,4737	-4,846 to 5,793	No	ns	>0,9999				
Males:Dark phase CRS vs. Females:Dark phase CRS	2,292	-2,698 to 7,282	No	ns	0,8515				
Females:Light phase CNT vs. Females:Light phase CRS	1,503	-3,784 to 6,790	No	ns	0,9881				
Females:Light phase CNT vs. Females:Dark phase CNT	3,313	-2,237 to 8,862	No	ns	0,5989				
Females:Light phase CNT vs. Females:Dark phase CRS	5,131	-0,1044 to 10,37	No	ns	0,0592				
Females:Light phase CRS vs. Females:Dark phase CNT	1,810	-3,389 to 7,008	No	ns	0,9623				
Females:Light phase CRS vs. Females:Dark phase CRS	3,628	-1,233 to 8,489	No	ns	0,3047				
Females:Dark phase CNT vs. Females:Dark phase CRS	1,818	-3,327 to 6,963	No	ns	0,9592				
Test details	Predicted (LS) mean 1	Predicted (LS) mean 2	Predicted (LS) mean diff,	SE of diff,	N1	N2	q	DF	
Males:Light phase CNT vs. Males:Light phase CRS	17,08	13,14	3,941	1,351	25	36	4,125	165,0	
Males:Light phase CNT vs. Males:Dark phase CNT	17,08	14,06	3,021	1,631	25	17	2,619	165,0	
Males:Light phase CNT vs. Males:Dark phase CRS	17,08	12,47	4,606	1,579	25	19	4,124	165,0	
Males:Light phase CNT vs. Females:Light phase CNT	17,08	15,31	1,768	1,661	25	16	1,504	165,0	
Males:Light phase CNT vs. Females:Light phase CRS	17,08	13,81	3,270	1,536	25	21	3,011	165,0	
Males:Light phase CNT vs. Females:Dark phase CNT	17,08	12,00	5,080	1,631	25	17	4,404	165,0	
Males:Light phase CNT vs. Females:Dark phase CRS	17,08	10,18	6,898	1,517	25	22	6,431	165,0	
Males:Light phase CRS vs. Males:Dark phase CNT	13,14	14,06	-0,9199	1,527	36	17	0,8519	165,0	
Males:Light phase CRS vs. Males:Dark phase CRS	13,14	12,47	0,6652	1,472	36	19	0,6393	165,0	
Males:Light phase CRS vs. Females:Light phase CNT	13,14	15,31	-2,174	1,559	36	16	1,971	165,0	
Males:Light phase CRS vs. Females:Light phase CRS	13,14	13,81	-0,6706	1,425	36	21	0,6656	165,0	
Males:Light phase CRS vs. Females:Dark phase CNT	13,14	12,00	1,139	1,527	36	17	1,055	165,0	
Males:Light phase CRS vs. Females:Dark phase CRS	13,14	10,18	2,957	1,404	36	22	2,978	165,0	
Males:Dark phase CNT vs. Males:Dark phase CRS	14,06	12,47	1,585	1,733	17	19	1,294	165,0	
Males:Dark phase CNT vs. Females:Light phase CNT	14,06	15,31	-1,254	1,808	17	16	0,9808	165,0	
Males:Dark phase CNT vs. Females:Light phase CRS	14,06	13,81	0,2493	1,693	17	21	0,2082	165,0	
Males:Dark phase CNT vs. Females:Dark phase CNT	14,06	12,00	2,059	1,780	17	17	1,636	165,0	
Males:Dark phase CNT vs. Females:Dark phase CRS	14,06	10,18	3,877	1,676	17	22	3,272	165,0	
Males:Dark phase CRS vs. Females:Light phase CNT	12,47	15,31	-2,839	1,761	19	16	2,280	165,0	
Males:Dark phase CRS vs. Females:Light phase CRS	12,47	13,81	-1,336	1,643	19	21	1,150	165,0	
Males:Dark phase CRS vs. Females:Dark phase CNT	12,47	12,00	0,4737	1,733	19	17	0,3867	165,0	
Males:Dark phase CRS vs. Females:Dark phase CRS	12,47	10,18	2,292	1,625	19	22	1,994	165,0	
Females:Light phase CNT vs. Females:Light phase CRS	15,31	13,81	1,503	1,722	16	21	1,234	165,0	
Females:Light phase CNT vs. Females:Dark phase CNT	15,31	12,00	3,313	1,808	16	17	2,592	165,0	
Females:Light phase CNT vs. Females:Dark phase CRS	15,31	10,18	5,131	1,705	16	22	4,255	165,0	
Females:Light phase CRS vs. Females:Dark phase CNT	13,81	12,00	1,810	1,693	21	17	1,511	165,0	
Females:Light phase CRS vs. Females:Dark phase CRS	13,81	10,18	3,628	1,583	21	22	3,240	165,0	
Females:Dark phase CNT vs. Females:Dark phase CRS	12,00	10,18	1,818	1,676	17	22	1,534	165,0	

Statistics of Figure 3c

Table Analyzed	SST latency to grooming; Grouped: Three-way ANOVA (2x2x2)				
Three-way ANOVA	Ordinary				
Alpha	0,05				
Source of Variation	% of total variation	P value	P value summary	Significant?	
sex	2,042	0,0494	*	Yes	
light	16,36	<0,0001	****	Yes	
stress	2,447	0,0317	*	Yes	
sex x light	5,272	0,0018	**	Yes	
sex x stress	0,04440	0,7705	ns	No	
light x stress	0,4293	0,3649	ns	No	
sex x light x stress	3,953	0,0066	**	Yes	
ANOVA table	SS (Type III)	DF	MS	F (DFn, DFd)	P value
stress	129,2	1	129,2	F (1, 137) = 3,930	P=0,0494
light	1035	1	1035	F (1, 137) = 31,49	P<0,0001
sex	154,8	1	154,8	F (1, 137) = 4,711	P=0,0317
stress x light	333,6	1	333,6	F (1, 137) = 10,15	P=0,0018
stress x sex	2,809	1	2,809	F (1, 137) = 0,08547	P=0,7705
light x sex	27,16	1	27,16	F (1, 137) = 0,8263	P=0,3649
stress x light x sex	250,1	1	250,1	F (1, 137) = 7,609	P=0,0066
Residual	4503	137	32,87		

Compare each cell mean with every other cell mean								
Number of families	1							
Number of comparisons per family	28							
Alpha	0,05							
Tukey's multiple comparisons test	Predicted (LS) mean diff,	95,00% CI of diff,	Below threshold?	Summary	Adjusted P Value			
Males:Light phase CNT vs. Males:Light phase CRS	-4,218	-9,245 to 0,8104	No	ns	0,1715			
Males:Light phase CNT vs. Males:Dark phase CNT	-4,182	-10,22 to 1,852	No	ns	0,3989			
Males:Light phase CNT vs. Males:Dark phase CRS	-4,782	-10,23 to 0,6706	No	ns	0,1316			
Males:Light phase CNT vs. Females:Light phase CNT	-1,807	-7,605 to 3,992	No	ns	0,9792			
Males:Light phase CNT vs. Females:Light phase CRS	-0,05682	-5,855 to 5,742	No	ns	>0,9999			
Males:Light phase CNT vs. Females:Dark phase CNT	-6,825	-12,86 to -0,7912	Yes	*	0,0150			
Males:Light phase CNT vs. Females:Dark phase CRS	-12,25	-18,16 to -6,339	Yes	****	<0,0001			
Males:Light phase CRS vs. Males:Dark phase CNT	0,03571	-5,741 to 5,812	No	ns	>0,9999			
Males:Light phase CRS vs. Males:Dark phase CRS	-0,5643	-5,731 to 4,602	No	ns	>0,9999			
Males:Light phase CRS vs. Females:Light phase CNT	2,411	-3,120 to 7,941	No	ns	0,8811			
Males:Light phase CRS vs. Females:Light phase CRS	4,161	-1,370 to 9,691	No	ns	0,2927			
Males:Light phase CRS vs. Females:Dark phase CNT	-2,607	-8,384 to 3,169	No	ns	0,8607			
Males:Light phase CRS vs. Females:Dark phase CRS	-8,031	-13,68 to -2,384	Yes	***	0,0006			
Males:Dark phase CNT vs. Males:Dark phase CRS	-0,6000	-6,750 to 5,550	No	ns	>0,9999			
Males:Dark phase CNT vs. Females:Light phase CNT	2,375	-4,083 to 8,833	No	ns	0,9486			
Males:Dark phase CNT vs. Females:Light phase CRS	4,125	-2,333 to 10,58	No	ns	0,5085			

Males:Dark phase CNT vs. Females:Dark phase CNT	-2,643	-9,313 to 4,027	No	ns	0,9248				
Males:Dark phase CNT vs. Females:Dark phase CRS	-8,067	-14,62 to -1,509	Yes	**	0,0055				
Males:Dark phase CRS vs. Females:Light phase CNT	2,975	-2,944 to 8,894	No	ns	0,7803				
Males:Dark phase CRS vs. Females:Light phase CRS	4,725	-1,194 to 10,64	No	ns	0,2230				
Males:Dark phase CRS vs. Females:Dark phase CNT	-2,043	-8,193 to 4,107	No	ns	0,9703				
Males:Dark phase CRS vs. Females:Dark phase CRS	-7,467	-13,49 to -1,439	Yes	**	0,0050				
Females:Light phase CNT vs. Females:Light phase CRS	1,750	-4,489 to 7,989	No	ns	0,9887				
Females:Light phase CNT vs. Females:Dark phase CNT	-5,018	-11,48 to 1,441	No	ns	0,2538				
Females:Light phase CNT vs. Females:Dark phase CRS	-10,44	-16,78 to -4,099	Yes	****	<0,0001				
Females:Light phase CRS vs. Females:Dark phase CNT	-6,768	-13,23 to -0,3094	Yes	*	0,0328				
Females:Light phase CRS vs. Females:Dark phase CRS	-12,19	-18,53 to -5,849	Yes	****	<0,0001				
Females:Dark phase CNT vs. Females:Dark phase CRS	-5,424	-11,98 to 1,134	No	ns	0,1855				
Test details	Predicted (LS) mean 1	Predicted (LS) mean 2	Predicted (LS) mean diff,	SE of diff,	N1	N2	q	DF	
Males:Light phase CNT vs. Males:Light phase CRS	5,818	10,04	-4,218	1,633	22	28	3,652	137,0	
Males:Light phase CNT vs. Males:Dark phase CNT	5,818	10,00	-4,182	1,960	22	14	3,017	137,0	
Males:Light phase CNT vs. Males:Dark phase CRS	5,818	10,60	-4,782	1,771	22	20	3,818	137,0	
Males:Light phase CNT vs. Females:Light phase CNT	5,818	7,625	-1,807	1,884	22	16	1,357	137,0	
Males:Light phase CNT vs. Females:Light phase CRS	5,818	5,875	-0,05682	1,884	22	16	0,04266	137,0	
Males:Light phase CNT vs. Females:Dark phase CNT	5,818	12,64	-6,825	1,960	22	14	4,924	137,0	
Males:Light phase CNT vs. Females:Dark phase CRS	5,818	18,07	-12,25	1,920	22	15	9,024	137,0	
Males:Light phase CRS vs. Males:Dark phase CNT	10,04	10,00	0,03571	1,877	28	14	0,02692	137,0	
Males:Light phase CRS vs. Males:Dark phase CRS	10,04	10,60	-0,5643	1,678	28	20	0,4755	137,0	
Males:Light phase CRS vs. Females:Light phase CNT	10,04	7,625	2,411	1,797	28	16	1,898	137,0	
Males:Light phase CRS vs. Females:Light phase CRS	10,04	5,875	4,161	1,797	28	16	3,275	137,0	
Males:Light phase CRS vs. Females:Dark phase CNT	10,04	12,64	-2,607	1,877	28	14	1,965	137,0	
Males:Light phase CRS vs. Females:Dark phase CRS	10,04	18,07	-8,031	1,834	28	15	6,192	137,0	
Males:Dark phase CNT vs. Males:Dark phase CRS	10,00	10,60	-0,6000	1,998	14	20	0,4247	137,0	
Males:Dark phase CNT vs. Females:Light phase CNT	10,00	7,625	2,375	2,098	14	16	1,601	137,0	
Males:Dark phase CNT vs. Females:Light phase CRS	10,00	5,875	4,125	2,098	14	16	2,781	137,0	
Males:Dark phase CNT vs. Females:Dark phase CNT	10,00	12,64	-2,643	2,167	14	14	1,725	137,0	
Males:Dark phase CNT vs. Females:Dark phase CRS	10,00	18,07	-8,067	2,130	14	15	5,355	137,0	
Males:Dark phase CRS vs. Females:Light phase CNT	10,60	7,625	2,975	1,923	20	16	2,188	137,0	
Males:Dark phase CRS vs. Females:Light phase CRS	10,60	5,875	4,725	1,923	20	16	3,475	137,0	
Males:Dark phase CRS vs. Females:Dark phase CNT	10,60	12,64	-2,043	1,998	20	14	1,446	137,0	
Males:Dark phase CRS vs. Females:Dark phase CRS	10,60	18,07	-7,467	1,958	20	15	5,393	137,0	
Females:Light phase CNT vs. Females:Light phase CRS	7,625	5,875	1,750	2,027	16	16	1,221	137,0	
Females:Light phase CNT vs. Females:Dark phase CNT	7,625	12,64	-5,018	2,098	16	14	3,382	137,0	
Females:Light phase CNT vs. Females:Dark phase CRS	7,625	18,07	-10,44	2,060	16	15	7,167	137,0	
Females:Light phase CRS vs. Females:Dark phase CNT	5,875	12,64	-6,768	2,098	16	14	4,562	137,0	
Females:Light phase CRS vs. Females:Dark phase CRS	5,875	18,07	-12,19	2,060	16	15	8,368	137,0	
Females:Dark phase CNT vs. Females:Dark phase CRS	12,64	18,07	-5,424	2,130	14	15	3,600	137,0	
