# Supplementary material for: Sex and Circadian Rhythm Dependent Behavioral Effects of Chronic Stress in Mice and Modulation of Clock Genes in the Prefrontal Cortex
Source: Int J Mol Sci. 2025 Jul 3;26(13):6410. doi: 10.3390/ijms26136410 (PMC12250008; doi:10.3390/ijms26136410)
Supplement: Supplementary file 1 [file ijms-26-06410-s001.zip › Table S4.pdf]

Supplementary Table S4

Statistics of Figure 4a

Table Analyzed	z-score anxious behavior				
Three-way ANOVA	Ordinary				
Alpha	0,05				
Source of Variation	% of total variation	P value	P value summary	Significant?	
sex	4,378	0,0017	**	Yes	
light	1,169	0,1002	ns	No	
stress	13,11	<0,0001	****	Yes	
sex x light	2,588	0,0150	*	Yes	
sex x stress	4,526	0,0014	**	Yes	
light x stress	2,282	0,0222	*	Yes	
sex x light x stress	2,410	0,0188	*	Yes	
ANOVA table	SS (Type III)	DF	MS	F (DFn, DFd)	P value
stress	22,65	1	22,65	F (1, 164) = 10,23	P=0,0017
light	6,050	1	6,050	F (1, 164) = 2,732	P=0,1002
sex	67,86	1	67,86	F (1, 164) = 30,65	P<0,0001
stress x light	13,39	1	13,39	F (1, 164) = 6,047	P=0,0150
stress x sex	23,42	1	23,42	F (1, 164) = 10,58	P=0,0014
light x sex	11,81	1	11,81	F (1, 164) = 5,333	P=0,0222
stress x light x sex	12,47	1	12,47	F (1, 164) = 5,633	P=0,0188
Residual	363,1	164	2,214		

Compare each cell mean with every other cell mean								
Number of families	1							
Number of comparisons per family	28							
Alpha	0,05							
Tukey's multiple comparisons test	Predicted (LS) mean diff,	95,00% CI of diff,	Below threshold?	Summary	Adjusted P Value			
Males:Light phase CNT vs. Males:Light phase CRS	-0,5494	-1,740 to 0,6409	No	ns	0,8482			
Males:Light phase CNT vs. Males:Dark phase CNT	0,1737	-1,185 to 1,533	No	ns	>0,9999			
Males:Light phase CNT vs. Males:Dark phase CRS	-0,3458	-1,705 to 1,013	No	ns	0,9939			
Males:Light phase CNT vs. Females:Light phase CNT	0,03258	-1,482 to 1,547	No	ns	>0,9999			
Males:Light phase CNT vs. Females:Light phase CRS	-0,9281	-2,269 to 0,4124	No	ns	0,4029			
Males:Light phase CNT vs. Females:Dark phase CNT	0,1662	-1,259 to 1,591	No	ns	>0,9999			
Males:Light phase CNT vs. Females:Dark phase CRS	-2,986	-4,345 to -1,627	Yes	****	<0,0001			
Males:Light phase CRS vs. Males:Dark phase CNT	0,7231	-0,5645 to 2,011	No	ns	0,6714			
Males:Light phase CRS vs. Males:Dark phase CRS	0,2036	-1,084 to 1,491	No	ns	0,9997			
Males:Light phase CRS vs. Females:Light phase CNT	0,5820	-0,8689 to 2,033	No	ns	0,9213			
Males:Light phase CRS vs. Females:Light phase CRS	-0,3787	-1,647 to 0,8894	No	ns	0,9841			
Males:Light phase CRS vs. Females:Dark phase CNT	0,7156	-0,6416 to 2,073	No	ns	0,7383			
Males:Light phase CRS vs. Females:Dark phase CRS	-2,437	-3,724 to -1,149	Yes	****	<0,0001			
Males:Dark phase CNT vs. Males:Dark phase CRS	-0,5195	-1,964 to 0,9254	No	ns	0,9552			
Males:Dark phase CNT vs. Females:Light phase CNT	-0,1411	-1,733 to 1,451	No	ns	>0,9999			
Males:Dark phase CNT vs. Females:Light phase CRS	-1,102	-2,529 to 0,3258	No	ns	0,2633			

Males:Dark phase CNT vs. Females:Dark phase CNT	-0,007500	-1,515 to 1,500	No	ns	>0,9999			
Males:Dark phase CNT vs. Females:Dark phase CRS	-3,160	-4,605 to -1,715	Yes	****	<0,0001			
Males:Dark phase CRS vs. Females:Light phase CNT	0,3784	-1,214 to 1,971	No	ns	0,9960			
Males:Dark phase CRS vs. Females:Light phase CRS	-0,5823	-2,010 to 0,8453	No	ns	0,9146			
Males:Dark phase CRS vs. Females:Dark phase CNT	0,5120	-0,9953 to 2,019	No	ns	0,9670			
Males:Dark phase CRS vs. Females:Dark phase CRS	-2,641	-4,085 to -1,196	Yes	****	<0,0001			
Females:Light phase CNT vs. Females:Light phase CRS	-0,9607	-2,537 to 0,6158	No	ns	0,5726			
Females:Light phase CNT vs. Females:Dark phase CNT	0,1336	-1,515 to 1,783	No	ns	>0,9999			
Females:Light phase CNT vs. Females:Dark phase CRS	-3,019	-4,611 to -1,427	Yes	****	<0,0001			
Females:Light phase CRS vs. Females:Dark phase CNT	1,094	-0,3964 to 2,585	No	ns	0,3257			
Females:Light phase CRS vs. Females:Dark phase CRS	-2,058	-3,486 to -0,6307	Yes	***	0,0005			
Females:Dark phase CNT vs. Females:Dark phase CRS	-3,153	-4,660 to -1,645	Yes	****	<0,0001			

Test details	Predicted (LS) mean 1	Predicted (LS) mean 2	Predicted (LS) mean diff,	SE of diff,	N1	N2	q	DF
Males:Light phase CNT vs. Males:Light phase CRS	0,1662	0,7156	-0,5494	0,3877	26	34	2,004	164,0
Males:Light phase CNT vs. Males:Dark phase CNT	0,1662	-0,007500	0,1737	0,4426	26	20	0,5549	164,0
Males:Light phase CNT vs. Males:Dark phase CRS	0,1662	0,5120	-0,3458	0,4426	26	20	1,105	164,0
Males:Light phase CNT vs. Females:Light phase CNT	0,1662	0,1336	0,03258	0,4933	26	14	0,09341	164,0
Males:Light phase CNT vs. Females:Light phase CRS	0,1662	1,094	-0,9281	0,4366	26	21	3,006	164,0
Males:Light phase CNT vs. Females:Dark phase CNT	0,1662	0,000	0,1662	0,4641	26	17	0,5063	164,0
Males:Light phase CNT vs. Females:Dark phase CRS	0,1662	3,153	-2,986	0,4426	26	20	9,542	164,0
Males:Light phase CRS vs. Males:Dark phase CNT	0,7156	-0,007500	0,7231	0,4193	34	20	2,439	164,0
Males:Light phase CRS vs. Males:Dark phase CRS	0,7156	0,5120	0,2036	0,4193	34	20	0,6866	164,0
Males:Light phase CRS vs. Females:Light phase CNT	0,7156	0,1336	0,5820	0,4725	34	14	1,742	164,0
Males:Light phase CRS vs. Females:Light phase CRS	0,7156	1,094	-0,3787	0,4130	34	21	1,297	164,0
Males:Light phase CRS vs. Females:Dark phase CNT	0,7156	0,000	0,7156	0,4420	34	17	2,289	164,0
Males:Light phase CRS vs. Females:Dark phase CRS	0,7156	3,153	-2,437	0,4193	34	20	8,218	164,0
Males:Dark phase CNT vs. Males:Dark phase CRS	-0,007500	0,5120	-0,5195	0,4706	20	20	1,561	164,0
Males:Dark phase CNT vs. Females:Light phase CNT	-0,007500	0,1336	-0,1411	0,5185	20	14	0,3847	164,0
Males:Dark phase CNT vs. Females:Light phase CRS	-0,007500	1,094	-1,102	0,4649	20	21	3,351	164,0
Males:Dark phase CNT vs. Females:Dark phase CNT	-0,007500	0,000	-0,007500	0,4909	20	17	0,02161	164,0
Males:Dark phase CNT vs. Females:Dark phase CRS	-0,007500	3,153	-3,160	0,4706	20	20	9,497	164,0
Males:Dark phase CRS vs. Females:Light phase CNT	0,5120	0,1336	0,3784	0,5185	20	14	1,032	164,0
Males:Dark phase CRS vs. Females:Light phase CRS	0,5120	1,094	-0,5823	0,4649	20	21	1,771	164,0
Males:Dark phase CRS vs. Females:Dark phase CNT	0,5120	0,000	0,5120	0,4909	20	17	1,475	164,0
Males:Dark phase CRS vs. Females:Dark phase CRS	0,5120	3,153	-2,641	0,4706	20	20	7,936	164,0
Females:Light phase CNT vs. Females:Light phase CRS	0,1336	1,094	-0,9607	0,5134	14	21	2,646	164,0
Females:Light phase CNT vs. Females:Dark phase CNT	0,1336	0,000	0,1336	0,5370	14	17	0,3517	164,0
Females:Light phase CNT vs. Females:Dark phase CRS	0,1336	3,153	-3,019	0,5185	14	20	8,234	164,0
Females:Light phase CRS vs. Females:Dark phase CNT	1,094	0,000	1,094	0,4855	21	17	3,188	164,0
Females:Light phase CRS vs. Females:Dark phase CRS	1,094	3,153	-2,058	0,4649	21	20	6,261	164,0
Females:Dark phase CNT vs. Females:Dark phase CRS	0,000	3,153	-3,153	0,4909	17	20	9,082	164,0

Statistics of Figure 4b

Table Analyzed	z-score depressive behavior				
Three-way ANOVA	Ordinary				
Alpha	0,05				
Source of Variation	% of total variation	P value	P value summary	Significant?	
sex	0,3899	0,3887	ns	No	
light	0,5256	0,3172	ns	No	
stress	10,80	<0,0001	****	Yes	
sex x light	0,8511	0,2035	ns	No	
sex x stress	0,3037	0,4467	ns	No	
light x stress	1,544	0,0875	ns	No	
sex x light x stress	0,1044	0,6553	ns	No	
ANOVA table	SS (Type III)	DF	MS	F (DFn, DFd)	P value
stress	1,241	1	1,241	F (1, 160) = 0,7469	P=0,3887
light	1,673	1	1,673	F (1, 160) = 1,007	P=0,3172
sex	34,37	1	34,37	F (1, 160) = 20,68	P<0,0001
stress x light	2,709	1	2,709	F (1, 160) = 1,630	P=0,2035
stress x sex	0,9666	1	0,9666	F (1, 160) = 0,5818	P=0,4467
light x sex	4,913	1	4,913	F (1, 160) = 2,957	P=0,0875
stress x light x sex	0,3323	1	0,3323	F (1, 160) = 0,2000	P=0,6553
Residual	265,8	160	1,662		

Compare each cell mean with every other cell mean								
Number of families	1							
Number of comparisons per family	28							
Alpha	0,05							
Tukey's multiple comparisons test	Predicted (LS) mean diff,	95,00% CI of diff,	Below threshold?	Summary	Adjusted P Value			
Males:Light phase CNT vs. Males:Light phase CRS	-1,220	-2,248 to -0,1928	Yes	**	0,0084			
Males:Light phase CNT vs. Males:Dark phase CNT	0,02326	-1,145 to 1,191	No	ns	>0,9999			
Males:Light phase CNT vs. Males:Dark phase CRS	-0,3085	-1,513 to 0,8962	No	ns	0,9936			
Males:Light phase CNT vs. Females:Light phase CNT	0,1493	-1,126 to 1,424	No	ns	>0,9999			
Males:Light phase CNT vs. Females:Light phase CRS	-1,200	-2,368 to -0,03223	Yes	*	0,0393			
Males:Light phase CNT vs. Females:Dark phase CNT	-0,1676	-1,417 to 1,081	No	ns	>0,9999			
Males:Light phase CNT vs. Females:Dark phase CRS	-0,9955	-2,181 to 0,1901	No	ns	0,1714			
Males:Light phase CRS vs. Males:Dark phase CNT	1,243	0,1215 to 2,365	Yes	*	0,0186			
Males:Light phase CRS vs. Males:Dark phase CRS	0,9116	-0,2485 to 2,072	No	ns	0,2420			
Males:Light phase CRS vs. Females:Light phase CNT	1,369	0,1365 to 2,602	Yes	*	0,0182			
Males:Light phase CRS vs. Females:Light phase CRS	0,01989	-1,102 to 1,142	No	ns	>0,9999			
Males:Light phase CRS vs. Females:Dark phase CNT	1,053	-0,1536 to 2,259	No	ns	0,1363			
Males:Light phase CRS vs. Females:Dark phase CRS	0,2247	-0,9155 to 1,365	No	ns	0,9988			
Males:Dark phase CNT vs. Males:Dark phase CRS	-0,3318	-1,618 to 0,9545	No	ns	0,9933			
Males:Dark phase CNT vs. Females:Light phase CNT	0,1260	-1,226 to 1,478	No	ns	>0,9999			
Males:Dark phase CNT vs. Females:Light phase CRS	-1,224	-2,475 to 0,02848	No	ns	0,0606			

Males:Dark phase CNT vs. Females:Dark phase CNT	-0,1909	-1,519 to 1,137	No	ns	0,9998			
Males:Dark phase CNT vs. Females:Dark phase CRS	-1,019	-2,287 to 0,2496	No	ns	0,2174			
Males:Dark phase CRS vs. Females:Light phase CNT	0,4578	-0,9263 to 1,842	No	ns	0,9715			
Males:Dark phase CRS vs. Females:Light phase CRS	-0,8917	-2,178 to 0,3946	No	ns	0,4008			
Males:Dark phase CRS vs. Females:Dark phase CNT	0,1409	-1,219 to 1,501	No	ns	>0,9999			
Males:Dark phase CRS vs. Females:Dark phase CRS	-0,6870	-1,989 to 0,6153	No	ns	0,7374			
Females:Light phase CNT vs. Females:Light phase CRS	-1,350	-2,702 to 0,002789	No	ns	0,0509			
Females:Light phase CNT vs. Females:Dark phase CNT	-0,3169	-1,740 to 1,106	No	ns	0,9973			
Females:Light phase CNT vs. Females:Dark phase CRS	-1,145	-2,512 to 0,2227	No	ns	0,1744			
Females:Light phase CRS vs. Females:Dark phase CNT	1,033	-0,2953 to 2,361	No	ns	0,2542			
Females:Light phase CRS vs. Females:Dark phase CRS	0,2048	-1,064 to 1,473	No	ns	0,9997			
Females:Dark phase CNT vs. Females:Dark phase CRS	-0,8279	-2,171 to 0,5155	No	ns	0,5579			

Test details	Predicted (LS) mean 1	Predicted (LS) mean 2	Predicted (LS) mean diff,	SE of diff,	N1	N2	q	DF
Males:Light phase CNT vs. Males:Light phase CRS	-0,0007407	1,219	-1,220	0,3345	27	33	5,159	160,0
Males:Light phase CNT vs. Males:Dark phase CNT	-0,0007407	-0,02400	0,02326	0,3803	27	20	0,08650	160,0
Males:Light phase CNT vs. Males:Dark phase CRS	-0,0007407	0,3078	-0,3085	0,3922	27	18	1,112	160,0
Males:Light phase CNT vs. Females:Light phase CNT	-0,0007407	-0,1500	0,1493	0,4151	27	15	0,5085	160,0
Males:Light phase CNT vs. Females:Light phase CRS	-0,0007407	1,200	-1,200	0,3803	27	20	4,464	160,0
Males:Light phase CNT vs. Females:Dark phase CNT	-0,0007407	0,1669	-0,1676	0,4067	27	16	0,5829	160,0
Males:Light phase CNT vs. Females:Dark phase CRS	-0,0007407	0,9947	-0,9955	0,3860	27	19	3,647	160,0
Males:Light phase CRS vs. Males:Dark phase CNT	1,219	-0,02400	1,243	0,3653	33	20	4,814	160,0
Males:Light phase CRS vs. Males:Dark phase CRS	1,219	0,3078	0,9116	0,3777	33	18	3,413	160,0
Males:Light phase CRS vs. Females:Light phase CNT	1,219	-0,1500	1,369	0,4014	33	15	4,825	160,0
Males:Light phase CRS vs. Females:Light phase CRS	1,219	1,200	0,01989	0,3653	33	20	0,07702	160,0
Males:Light phase CRS vs. Females:Dark phase CNT	1,219	0,1669	1,053	0,3927	33	16	3,791	160,0
Males:Light phase CRS vs. Females:Dark phase CRS	1,219	0,9947	0,2247	0,3712	33	19	0,8559	160,0
Males:Dark phase CNT vs. Males:Dark phase CRS	-0,02400	0,3078	-0,3318	0,4188	20	18	1,120	160,0
Males:Dark phase CNT vs. Females:Light phase CNT	-0,02400	-0,1500	0,1260	0,4403	20	15	0,4047	160,0
Males:Dark phase CNT vs. Females:Light phase CRS	-0,02400	1,200	-1,224	0,4076	20	20	4,245	160,0
Males:Dark phase CNT vs. Females:Dark phase CNT	-0,02400	0,1669	-0,1909	0,4323	20	16	0,6244	160,0
Males:Dark phase CNT vs. Females:Dark phase CRS	-0,02400	0,9947	-1,019	0,4129	20	19	3,489	160,0
Males:Dark phase CRS vs. Females:Light phase CNT	0,3078	-0,1500	0,4578	0,4506	18	15	1,437	160,0
Males:Dark phase CRS vs. Females:Light phase CRS	0,3078	1,200	-0,8917	0,4188	18	20	3,011	160,0
Males:Dark phase CRS vs. Females:Dark phase CNT	0,3078	0,1669	0,1409	0,4429	18	16	0,4499	160,0
Males:Dark phase CRS vs. Females:Dark phase CRS	0,3078	0,9947	-0,6870	0,4240	18	19	2,291	160,0
Females:Light phase CNT vs. Females:Light phase CRS	-0,1500	1,200	-1,350	0,4403	15	20	4,335	160,0
Females:Light phase CNT vs. Females:Dark phase CNT	-0,1500	0,1669	-0,3169	0,4633	15	16	0,9673	160,0
Females:Light phase CNT vs. Females:Dark phase CRS	-0,1500	0,9947	-1,145	0,4452	15	19	3,636	160,0
Females:Light phase CRS vs. Females:Dark phase CNT	1,200	0,1669	1,033	0,4323	20	16	3,378	160,0
Females:Light phase CRS vs. Females:Dark phase CRS	1,200	0,9947	0,2048	0,4129	20	19	0,7013	160,0
Females:Dark phase CNT vs. Females:Dark phase CRS	0,1669	0,9947	-0,8279	0,4374	16	19	2,677	160,0

Statistics of Figure 4c

Table Analyzed	z-score anhedonic behavior				
Three-way ANOVA	Ordinary				
Alpha	0,05				
Source of Variation	% of total variation	P value	P value summary	Significant?	
sex	0,1745	0,5738	ns	No	
light	0,5358	0,3248	ns	No	
stress	0,5500	0,3185	ns	No	
sex x light	1,821	0,0705	ns	No	
sex x stress	0,6102	0,2935	ns	No	
light x stress	2,580	0,0317	*	Yes	
sex x light x stress	1,025	0,1738	ns	No	
ANOVA table	SS (Type III)	DF	MS	F (DFn, DFd)	P value
stress	1,313	1	1,313	F (1, 163) = 0,3176	P=0,5738
light	4,030	1	4,030	F (1, 163) = 0,9752	P=0,3248
sex	4,137	1	4,137	F (1, 163) = 1,001	P=0,3185
stress x light	13,70	1	13,70	F (1, 163) = 3,314	P=0,0705
stress x sex	4,590	1	4,590	F (1, 163) = 1,111	P=0,2935
light x sex	19,41	1	19,41	F (1, 163) = 4,696	P=0,0317
stress x light x sex	7,713	1	7,713	F (1, 163) = 1,866	P=0,1738
Residual	673,6	163	4,133		

Compare each cell mean with every other cell mean								
Number of families	1							
Number of comparisons per family	28							
Alpha	0,05							
Tukey's multiple comparisons test	Predicted (LS) mean diff,	95,00% CI of diff,	Below threshold?	Summary	Adjusted P Value			
Males:Light phase CNT vs. Males:Light phase CRS	-1,779	-3,416 to -0,1424	Yes	*	0,0228			
Males:Light phase CNT vs. Males:Dark phase CNT	-0,2309	-2,145 to 1,683	No	ns	>0,9999			
Males:Light phase CNT vs. Males:Dark phase CRS	0,2400	-1,644 to 2,124	No	ns	>0,9999			
Males:Light phase CNT vs. Females:Light phase CNT	-0,01145	-1,926 to 1,903	No	ns	>0,9999			
Males:Light phase CNT vs. Females:Light phase CRS	-0,2495	-2,081 to 1,582	No	ns	0,9999			
Males:Light phase CNT vs. Females:Dark phase CNT	-0,5317	-2,515 to 1,452	No	ns	0,9916			
Males:Light phase CNT vs. Females:Dark phase CRS	-0,2597	-2,116 to 1,597	No	ns	0,9999			
Males:Light phase CRS vs. Males:Dark phase CNT	1,548	-0,2806 to 3,378	No	ns	0,1637			
Males:Light phase CRS vs. Males:Dark phase CRS	2,019	0,2217 to 3,817	Yes	*	0,0161			
Males:Light phase CRS vs. Females:Light phase CNT	1,768	-0,06120 to 3,597	No	ns	0,0662			
Males:Light phase CRS vs. Females:Light phase CRS	1,530	-0,2126 to 3,272	No	ns	0,1313			
Males:Light phase CRS vs. Females:Dark phase CNT	1,248	-0,6540 to 3,149	No	ns	0,4753			
Males:Light phase CRS vs. Females:Dark phase CRS	1,520	-0,2493 to 3,289	No	ns	0,1501			
Males:Dark phase CNT vs. Males:Dark phase CRS	0,4709	-1,582 to 2,524	No	ns	0,9968			
Males:Dark phase CNT vs. Females:Light phase CNT	0,2194	-1,861 to 2,300	No	ns	>0,9999			
Males:Dark phase CNT vs. Females:Light phase CRS	-0,01857	-2,024 to 1,987	No	ns	>0,9999			

Males:Dark phase CNT vs. Females:Dark phase CNT	-0,3008	-2,446 to 1,844	No	ns	0,9999			
Males:Dark phase CNT vs. Females:Dark phase CRS	-0,02883	-2,057 to 1,999	No	ns	>0,9999			
Males:Dark phase CRS vs. Females:Light phase CNT	-0,2514	-2,305 to 1,802	No	ns	>0,9999			
Males:Dark phase CRS vs. Females:Light phase CRS	-0,4894	-2,466 to 1,487	No	ns	0,9948			
Males:Dark phase CRS vs. Females:Dark phase CNT	-0,7717	-2,890 to 1,346	No	ns	0,9519			
Males:Dark phase CRS vs. Females:Dark phase CRS	-0,4997	-2,500 to 1,500	No	ns	0,9945			
Females:Light phase CNT vs. Females:Light phase CRS	-0,2380	-2,243 to 1,767	No	ns	>0,9999			
Females:Light phase CNT vs. Females:Dark phase CNT	-0,5203	-2,665 to 1,625	No	ns	0,9954			
Females:Light phase CNT vs. Females:Dark phase CRS	-0,2483	-2,276 to 1,780	No	ns	>0,9999			
Females:Light phase CRS vs. Females:Dark phase CNT	-0,2823	-2,354 to 1,789	No	ns	0,9999			
Females:Light phase CRS vs. Females:Dark phase CRS	-0,01026	-1,961 to 1,940	No	ns	>0,9999			
Females:Dark phase CNT vs. Females:Dark phase CRS	0,2720	-1,822 to 2,366	No	ns	>0,9999			

Test details	Predicted (LS) mean 1	Predicted (LS) mean 2	Predicted (LS) mean diff,	SE of diff,	N1	N2	q	DF
Males:Light phase CNT vs. Males:Light phase CRS	-0,2142	1,565	-1,779	0,5331	26	33	4,721	163,0
Males:Light phase CNT vs. Males:Dark phase CNT	-0,2142	0,01667	-0,2309	0,6233	26	18	0,5239	163,0
Males:Light phase CNT vs. Males:Dark phase CRS	-0,2142	-0,4542	0,2400	0,6136	26	19	0,5531	163,0
Males:Light phase CNT vs. Females:Light phase CNT	-0,2142	-0,2028	-0,01145	0,6233	26	18	0,02598	163,0
Males:Light phase CNT vs. Females:Light phase CRS	-0,2142	0,03524	-0,2495	0,5964	26	21	0,5915	163,0
Males:Light phase CNT vs. Females:Dark phase CNT	-0,2142	0,3175	-0,5317	0,6459	26	16	1,164	163,0
Males:Light phase CNT vs. Females:Dark phase CRS	-0,2142	0,04550	-0,2597	0,6046	26	20	0,6075	163,0
Males:Light phase CRS vs. Males:Dark phase CNT	1,565	0,01667	1,548	0,5957	33	18	3,676	163,0
Males:Light phase CRS vs. Males:Dark phase CRS	1,565	-0,4542	2,019	0,5854	33	19	4,878	163,0
Males:Light phase CRS vs. Females:Light phase CNT	1,565	-0,2028	1,768	0,5957	33	18	4,197	163,0
Males:Light phase CRS vs. Females:Light phase CRS	1,565	0,03524	1,530	0,5675	33	21	3,813	163,0
Males:Light phase CRS vs. Females:Dark phase CNT	1,565	0,3175	1,248	0,6193	33	16	2,849	163,0
Males:Light phase CRS vs. Females:Dark phase CRS	1,565	0,04550	1,520	0,5761	33	20	3,731	163,0
Males:Dark phase CNT vs. Males:Dark phase CRS	0,01667	-0,4542	0,4709	0,6687	18	19	0,9959	163,0
Males:Dark phase CNT vs. Females:Light phase CNT	0,01667	-0,2028	0,2194	0,6776	18	18	0,4580	163,0
Males:Dark phase CNT vs. Females:Light phase CRS	0,01667	0,03524	-0,01857	0,6530	18	21	0,04022	163,0
Males:Dark phase CNT vs. Females:Dark phase CNT	0,01667	0,3175	-0,3008	0,6985	18	16	0,6091	163,0
Males:Dark phase CNT vs. Females:Dark phase CRS	0,01667	0,04550	-0,02883	0,6605	18	20	0,06174	163,0
Males:Dark phase CRS vs. Females:Light phase CNT	-0,4542	-0,2028	-0,2514	0,6687	19	18	0,5318	163,0
Males:Dark phase CRS vs. Females:Light phase CRS	-0,4542	0,03524	-0,4894	0,6437	19	21	1,075	163,0
Males:Dark phase CRS vs. Females:Dark phase CNT	-0,4542	0,3175	-0,7717	0,6898	19	16	1,582	163,0
Males:Dark phase CRS vs. Females:Dark phase CRS	-0,4542	0,04550	-0,4997	0,6513	19	20	1,085	163,0
Females:Light phase CNT vs. Females:Light phase CRS	-0,2028	0,03524	-0,2380	0,6530	18	21	0,5155	163,0
Females:Light phase CNT vs. Females:Dark phase CNT	-0,2028	0,3175	-0,5203	0,6985	18	16	1,053	163,0
Females:Light phase CNT vs. Females:Dark phase CRS	-0,2028	0,04550	-0,2483	0,6605	18	20	0,5316	163,0
Females:Light phase CRS vs. Females:Dark phase CNT	0,03524	0,3175	-0,2823	0,6746	21	16	0,5917	163,0
Females:Light phase CRS vs. Females:Dark phase CRS	0,03524	0,04550	-0,01026	0,6352	21	20	0,02285	163,0
Females:Dark phase CNT vs. Females:Dark phase CRS	0,3175	0,04550	0,2720	0,6818	16	20	0,5642	163,0

Statistics of Figure 4d

Table Analyzed	z-score all behaviors				
Three-way ANOVA	Ordinary				
Alpha	0,05				
Source of Variation	% of total variation	P value	P value summary	Significant?	
sex	9,326	0,0017	**	Yes	
light	2,008	0,1242	ns	No	
stress	35,60	<0,0001	****	Yes	
sex x light	5,443	0,0136	*	Yes	
sex x stress	9,799	0,0013	**	Yes	
light x stress	2,231	0,1058	ns	No	
sex x light x stress	5,402	0,0139	*	Yes	
ANOVA table	SS (Type III)	DF	MS	F (DFn, DFd)	P value
stress	97,80	1	97,80	F (1, 38) = 11,48	P=0,0017
light	21,06	1	21,06	F (1, 38) = 2,471	P=0,1242
sex	373,3	1	373,3	F (1, 38) = 43,81	P<0,0001
stress x light	57,08	1	57,08	F (1, 38) = 6,698	P=0,0136
stress x sex	102,8	1	102,8	F (1, 38) = 12,06	P=0,0013
light x sex	23,39	1	23,39	F (1, 38) = 2,745	P=0,1058
stress x light x sex	56,65	1	56,65	F (1, 38) = 6,647	P=0,0139
Residual	323,9	38	8,523		

Compare each cell mean with every other cell mean								
Number of families	1							
Number of comparisons per family	28							
Alpha	0,05							
Tukey's multiple comparisons test	Predicted (LS) mean diff,	95,00% CI of diff,	Below threshold?	Summary	Adjusted P Value			
Males:Light phase CNT vs. Males:Light phase CRS	-6,396	-11,88 to -0,9163	Yes	*	0,0127			
Males:Light phase CNT vs. Males:Dark phase CNT	-0,06500	-5,731 to 5,601	No	ns	>0,9999			
Males:Light phase CNT vs. Males:Dark phase CRS	0,8783	-4,788 to 6,545	No	ns	0,9996			
Males:Light phase CNT vs. Females:Light phase CNT	0,08200	-5,836 to 6,000	No	ns	>0,9999			
Males:Light phase CNT vs. Females:Light phase CRS	-7,863	-13,53 to -2,197	Yes	**	0,0017			
Males:Light phase CNT vs. Females:Dark phase CNT	0,000	-5,666 to 5,666	No	ns	>0,9999			
Males:Light phase CNT vs. Females:Dark phase CRS	-9,542	-15,46 to -3,624	Yes	***	0,0002			
Males:Light phase CRS vs. Males:Dark phase CNT	6,331	1,124 to 11,54	Yes	**	0,0083			
Males:Light phase CRS vs. Males:Dark phase CRS	7,274	2,068 to 12,48	Yes	**	0,0016			
Males:Light phase CRS vs. Females:Light phase CNT	6,478	0,9983 to 11,96	Yes	*	0,0111			
Males:Light phase CRS vs. Females:Light phase CRS	-1,468	-6,674 to 3,739	No	ns	0,9839			
Males:Light phase CRS vs. Females:Dark phase CNT	6,396	1,189 to 11,60	Yes	**	0,0074			
Males:Light phase CRS vs. Females:Dark phase CRS	-3,146	-8,626 to 2,333	No	ns	0,5977			
Males:Dark phase CNT vs. Males:Dark phase CRS	0,9433	-4,459 to 6,346	No	ns	0,9992			
Males:Dark phase CNT vs. Females:Light phase CNT	0,1470	-5,519 to 5,813	No	ns	>0,9999			
Males:Dark phase CNT vs. Females:Light phase CRS	-7,798	-13,20 to -2,396	Yes	**	0,0010			

Males:Dark phase CNT vs. Females:Dark phase CNT	0,06500	-5,338 to 5,468	No	ns	>0,9999			
Males:Dark phase CNT vs. Females:Dark phase CRS	-9,477	-15,14 to -3,811	Yes	***	0,0001			
Males:Dark phase CRS vs. Females:Light phase CNT	-0,7963	-6,463 to 4,870	No	ns	0,9998			
Males:Dark phase CRS vs. Females:Light phase CRS	-8,742	-14,14 to -3,339	Yes	***	0,0002			
Males:Dark phase CRS vs. Females:Dark phase CNT	-0,8783	-6,281 to 4,524	No	ns	0,9995			
Males:Dark phase CRS vs. Females:Dark phase CRS	-10,42	-16,09 to -4,754	Yes	****	<0,0001			
Females:Light phase CNT vs. Females:Light phase CRS	-7,945	-13,61 to -2,279	Yes	**	0,0015			
Females:Light phase CNT vs. Females:Dark phase CNT	-0,08200	-5,748 to 5,584	No	ns	>0,9999			
Females:Light phase CNT vs. Females:Dark phase CRS	-9,624	-15,54 to -3,706	Yes	***	0,0002			
Females:Light phase CRS vs. Females:Dark phase CNT	7,863	2,461 to 13,27	Yes	***	0,0009			
Females:Light phase CRS vs. Females:Dark phase CRS	-1,679	-7,345 to 3,988	No	ns	0,9787			
Females:Dark phase CNT vs. Females:Dark phase CRS	-9,542	-15,21 to -3,876	Yes	****	<0,0001			

Test details	Predicted (LS) mean 1	Predicted (LS) mean 2	Predicted (LS) mean diff,	SE of diff,	N1	N2	q	DF
Males:Light phase CNT vs. Males:Light phase CRS	0,000	6,396	-6,396	1,709	5	7	5,291	38,00
Males:Light phase CNT vs. Males:Dark phase CNT	0,000	0,06500	-0,06500	1,768	5	6	0,05200	38,00
Males:Light phase CNT vs. Males:Dark phase CRS	0,000	-0,8783	0,8783	1,768	5	6	0,7027	38,00
Males:Light phase CNT vs. Females:Light phase CNT	0,000	-0,08200	0,08200	1,846	5	5	0,06281	38,00
Males:Light phase CNT vs. Females:Light phase CRS	0,000	7,863	-7,863	1,768	5	6	6,291	38,00
Males:Light phase CNT vs. Females:Dark phase CNT	0,000	0,000	0,000	1,768	5	6	0,000	38,00
Males:Light phase CNT vs. Females:Dark phase CRS	0,000	9,542	-9,542	1,846	5	5	7,309	38,00
Males:Light phase CRS vs. Males:Dark phase CNT	6,396	0,06500	6,331	1,624	7	6	5,512	38,00
Males:Light phase CRS vs. Males:Dark phase CRS	6,396	-0,8783	7,274	1,624	7	6	6,334	38,00
Males:Light phase CRS vs. Females:Light phase CNT	6,396	-0,08200	6,478	1,709	7	5	5,359	38,00
Males:Light phase CRS vs. Females:Light phase CRS	6,396	7,863	-1,468	1,624	7	6	1,278	38,00
Males:Light phase CRS vs. Females:Dark phase CNT	6,396	0,000	6,396	1,624	7	6	5,569	38,00
Males:Light phase CRS vs. Females:Dark phase CRS	6,396	9,542	-3,146	1,709	7	5	2,603	38,00
Males:Dark phase CNT vs. Males:Dark phase CRS	0,06500	-0,8783	0,9433	1,685	6	6	0,7915	38,00
Males:Dark phase CNT vs. Females:Light phase CNT	0,06500	-0,08200	0,1470	1,768	6	5	0,1176	38,00
Males:Dark phase CNT vs. Females:Light phase CRS	0,06500	7,863	-7,798	1,685	6	6	6,543	38,00
Males:Dark phase CNT vs. Females:Dark phase CNT	0,06500	0,000	0,06500	1,685	6	6	0,05454	38,00
Males:Dark phase CNT vs. Females:Dark phase CRS	0,06500	9,542	-9,477	1,768	6	5	7,582	38,00
Males:Dark phase CRS vs. Females:Light phase CNT	-0,8783	-0,08200	-0,7963	1,768	6	5	0,6371	38,00
Males:Dark phase CRS vs. Females:Light phase CRS	-0,8783	7,863	-8,742	1,685	6	6	7,335	38,00
Males:Dark phase CRS vs. Females:Dark phase CNT	-0,8783	0,000	-0,8783	1,685	6	6	0,7370	38,00
Males:Dark phase CRS vs. Females:Dark phase CRS	-0,8783	9,542	-10,42	1,768	6	5	8,336	38,00
Females:Light phase CNT vs. Females:Light phase CRS	-0,08200	7,863	-7,945	1,768	5	6	6,356	38,00
Females:Light phase CNT vs. Females:Dark phase CNT	-0,08200	0,000	-0,08200	1,768	5	6	0,06560	38,00
Females:Light phase CNT vs. Females:Dark phase CRS	-0,08200	9,542	-9,624	1,846	5	5	7,371	38,00
Females:Light phase CRS vs. Females:Dark phase CNT	7,863	0,000	7,863	1,685	6	6	6,598	38,00
Females:Light phase CRS vs. Females:Dark phase CRS	7,863	9,542	-1,679	1,768	6	5	1,343	38,00
Females:Dark phase CNT vs. Females:Dark phase CRS	0,000	9,542	-9,542	1,768	6	5	7,634	38,00
