# Supplementary material for: Sex and Circadian Rhythm Dependent Behavioral Effects of Chronic Stress in Mice and Modulation of Clock Genes in the Prefrontal Cortex
Source: Int J Mol Sci. 2025 Jul 3;26(13):6410. doi: 10.3390/ijms26136410 (PMC12250008; doi:10.3390/ijms26136410)
Supplement: Supplementary file 1 [file ijms-26-06410-s001.zip › Table S5.pdf]

Supplementary Table S5

Statistics of Figure 5a

Table Analyzed	CORT levels: Three-way ANOVA (2x2x2)				
Three-way ANOVA	Ordinary				
Alpha	0,05				
Source of Variation	% of total variation	P value	P value summary	Significant?	
sex	13,52	0,0011	**	Yes	
light	33,69	<0,0001	****	Yes	
stress	0,7870	0,4016	ns	No	
sex x light	6,013	0,0243	*	Yes	
sex x stress	0,9429	0,3590	ns	No	
light x stress	0,07440	0,7956	ns	No	
sex x light x stress	1,565	0,2389	ns	No	
ANOVA table	SS (Type III)	DF	MS	F (DFn, DFd)	P value
sex	19385	1	19385	F (1, 39) = 12,35	P=0,0011
light	48325	1	48325	F (1, 39) = 30,80	P<0,0001
stress	1129	1	1129	F (1, 39) = 0,7193	P=0,4016
sex x light	8623	1	8623	F (1, 39) = 5,496	P=0,0243
sex x stress	1352	1	1352	F (1, 39) = 0,8618	P=0,3590
light x stress	106,7	1	106,7	F (1, 39) = 0,06801	P=0,7956
sex x light x stress	2245	1	2245	F (1, 39) = 1,430	P=0,2389
Residual	61198	39	1569		

Compare each cell mean with every other cell mean								
Number of families	1							
Number of comparisons per family	28							
Alpha	0,05							
Tukey's multiple comparisons test	Predicted (LS) mean diff,	95,00% CI of diff,	Below threshold?	Summary	Adjusted P Value			
Males:Light phase CNT vs. Males:Light phase CRS	-9,933	-84,18 to 64,31	No	ns	0,9999			
Males:Light phase CNT vs. Males:Dark phase CNT	-48,10	-124,9 to 28,68	No	ns	0,4915			
Males:Light phase CNT vs. Males:Dark phase CRS	-36,31	-113,1 to 40,47	No	ns	0,7956			
Males:Light phase CNT vs. Females:Light phase CNT	-16,71	-96,90 to 63,48	No	ns	0,9974			
Males:Light phase CNT vs. Females:Light phase CRS	-20,42	-97,20 to 56,36	No	ns	0,9886			
Males:Light phase CNT vs. Females:Dark phase CNT	-91,49	-168,3 to -14,71	Yes	*	0,0102			
Males:Light phase CNT vs. Females:Dark phase CRS	-129,1	-205,8 to -52,27	Yes	****	<0,0001			
Males:Light phase CRS vs. Males:Dark phase CNT	-38,17	-108,7 to 32,37	No	ns	0,6672			
Males:Light phase CRS vs. Males:Dark phase CRS	-26,37	-96,91 to 44,17	No	ns	0,9281			
Males:Light phase CRS vs. Females:Light phase CNT	-6,775	-81,02 to 67,47	No	ns	>0,9999			
Males:Light phase CRS vs. Females:Light phase CRS	-10,49	-81,03 to 60,05	No	ns	0,9997			
Males:Light phase CRS vs. Females:Dark phase CNT	-81,56	-152,1 to -11,01	Yes	*	0,0139			
Males:Light phase CRS vs. Females:Dark phase CRS	-119,1	-189,7 to -48,58	Yes	****	<0,0001			
Males:Dark phase CNT vs. Males:Dark phase CRS	11,80	-61,41 to 85,00	No	ns	0,9995			
Males:Dark phase CNT vs. Females:Light phase CNT	31,39	-45,38 to 108,2	No	ns	0,8900			
Males:Dark phase CNT vs. Females:Light phase CRS	27,68	-45,53 to 100,9	No	ns	0,9240			

Males:Dark phase CNT vs. Females:Dark phase CNT	-43,39	-116,6 to 29,82	No	ns	0,5610			
Males:Dark phase CNT vs. Females:Dark phase CRS	-80,95	-154,2 to -7,745	Yes	*	0,0213			
Males:Dark phase CRS vs. Females:Light phase CNT	19,60	-57,18 to 96,38	No	ns	0,9911			
Males:Dark phase CRS vs. Females:Light phase CRS	15,88	-57,32 to 89,09	No	ns	0,9967			
Males:Dark phase CRS vs. Females:Dark phase CNT	-55,19	-128,4 to 18,02	No	ns	0,2638			
Males:Dark phase CRS vs. Females:Dark phase CRS	-92,75	-166,0 to -19,54	Yes	**	0,0052			
Females:Light phase CNT vs. Females:Light phase CRS	-3,714	-80,49 to 73,06	No	ns	>0,9999			
Females:Light phase CNT vs. Females:Dark phase CNT	-74,78	-151,6 to 1,996	No	ns	0,0610			
Females:Light phase CNT vs. Females:Dark phase CRS	-112,3	-189,1 to -35,57	Yes	***	0,0008			
Females:Light phase CRS vs. Females:Dark phase CNT	-71,07	-144,3 to 2,137	No	ns	0,0625			
Females:Light phase CRS vs. Females:Dark phase CRS	-108,6	-181,8 to -35,42	Yes	***	0,0007			
Females:Dark phase CNT vs. Females:Dark phase CRS	-37,56	-110,8 to 35,64	No	ns	0,7224			
Test details	Predicted (LS) mean 1	Predicted (LS) mean 2	Predicted (LS) mean diff,	SE of diff,	N1	N2	q	DF
Males:Light phase CNT vs. Males:Light phase CRS	19,25	29,18	-9,933	23,19	5	7	0,6056	39,00
Males:Light phase CNT vs. Males:Dark phase CNT	19,25	67,35	-48,10	23,99	5	6	2,836	39,00
Males:Light phase CNT vs. Males:Dark phase CRS	19,25	55,56	-36,31	23,99	5	6	2,140	39,00
Males:Light phase CNT vs. Females:Light phase CNT	19,25	35,96	-16,71	25,05	5	5	0,9431	39,00
Males:Light phase CNT vs. Females:Light phase CRS	19,25	39,67	-20,42	23,99	5	6	1,204	39,00
Males:Light phase CNT vs. Females:Dark phase CNT	19,25	110,7	-91,49	23,99	5	6	5,394	39,00
Males:Light phase CNT vs. Females:Dark phase CRS	19,25	148,3	-129,1	23,99	5	6	7,609	39,00
Males:Light phase CRS vs. Males:Dark phase CNT	29,18	67,35	-38,17	22,04	7	6	2,449	39,00
Males:Light phase CRS vs. Males:Dark phase CRS	29,18	55,56	-26,37	22,04	7	6	1,692	39,00
Males:Light phase CRS vs. Females:Light phase CNT	29,18	35,96	-6,775	23,19	7	5	0,4131	39,00
Males:Light phase CRS vs. Females:Light phase CRS	29,18	39,67	-10,49	22,04	7	6	0,6731	39,00
Males:Light phase CRS vs. Females:Dark phase CNT	29,18	110,7	-81,56	22,04	7	6	5,234	39,00
Males:Light phase CRS vs. Females:Dark phase CRS	29,18	148,3	-119,1	22,04	7	6	7,644	39,00
Males:Dark phase CNT vs. Males:Dark phase CRS	67,35	55,56	11,80	22,87	6	6	0,7295	39,00
Males:Dark phase CNT vs. Females:Light phase CNT	67,35	35,96	31,39	23,99	6	5	1,851	39,00
Males:Dark phase CNT vs. Females:Light phase CRS	67,35	39,67	27,68	22,87	6	6	1,712	39,00
Males:Dark phase CNT vs. Females:Dark phase CNT	67,35	110,7	-43,39	22,87	6	6	2,683	39,00
Males:Dark phase CNT vs. Females:Dark phase CRS	67,35	148,3	-80,95	22,87	6	6	5,006	39,00
Males:Dark phase CRS vs. Females:Light phase CNT	55,56	35,96	19,60	23,99	6	5	1,155	39,00
Males:Dark phase CRS vs. Females:Light phase CRS	55,56	39,67	15,88	22,87	6	6	0,9822	39,00
Males:Dark phase CRS vs. Females:Dark phase CNT	55,56	110,7	-55,19	22,87	6	6	3,412	39,00
Males:Dark phase CRS vs. Females:Dark phase CRS	55,56	148,3	-92,75	22,87	6	6	5,735	39,00
Females:Light phase CNT vs. Females:Light phase CRS	35,96	39,67	-3,714	23,99	5	6	0,2190	39,00
Females:Light phase CNT vs. Females:Dark phase CNT	35,96	110,7	-74,78	23,99	5	6	4,409	39,00
Females:Light phase CNT vs. Females:Dark phase CRS	35,96	148,3	-112,3	23,99	5	6	6,624	39,00
Females:Light phase CRS vs. Females:Dark phase CNT	39,67	110,7	-71,07	22,87	6	6	4,395	39,00
Females:Light phase CRS vs. Females:Dark phase CRS	39,67	148,3	-108,6	22,87	6	6	6,717	39,00
Females:Dark phase CNT vs. Females:Dark phase CRS	110,7	148,3	-37,56	22,87	6	6	2,323	39,00

Statistics of Figure 5c

Table Analyzed	Bmal1 Grouped: Three-way ANOVA (2x2x2)				
Three-way ANOVA	Ordinary				
Alpha	0,05				
Source of Variation	% of total variation	P value	P value summary	Significant?	
sex	5,386	0,0752	ns	No	
light	17,30	0,0022	**	Yes	
stress	7,009	0,0436	*	Yes	
sex x light	3,829	0,1312	ns	No	
sex x stress	0,4740	0,5905	ns	No	
light x stress	0,08490	0,8195	ns	No	
sex x light x stress	6,704	0,0482	*	Yes	
ANOVA table	SS (Type III)	DF	MS	F (DFn, DFd)	P value
sex	0,1424	1	0,1424	F (1, 38) = 3,347	P=0,0752
light	0,4576	1	0,4576	F (1, 38) = 10,75	P=0,0022
stress	0,1853	1	0,1853	F (1, 38) = 4,356	P=0,0436
sex x light	0,1013	1	0,1013	F (1, 38) = 2,380	P=0,1312
sex x stress	0,01253	1	0,01253	F (1, 38) = 0,2946	P=0,5905
light x stress	0,002245	1	0,002245	F (1, 38) = 0,05277	P=0,8195
sex x light x stress	0,1773	1	0,1773	F (1, 38) = 4,167	P=0,0482
Residual	1,617	38	0,04255		

Compare each cell mean with every other cell mean								
Number of families	1							
Number of comparisons per family	28							
Alpha	0,05							
Tukey's multiple comparisons test	Predicted (LS) mean diff,	95,00% CI of diff,	Below threshold?	Summary	Adjusted P Value			
Males:Light phase CNT vs. Males:Light phase CRS	0,2328	-0,1676 to 0,6332	No	ns	0,5827			
Males:Light phase CNT vs. Males:Dark phase CNT	0,4328	0,03242 to 0,8332	Yes	*	0,0262			
Males:Light phase CNT vs. Males:Dark phase CRS	0,3885	-0,01192 to 0,7888	No	ns	0,0627			
Males:Light phase CNT vs. Females:Light phase CNT	0,2972	-0,1210 to 0,7154	No	ns	0,3315			
Males:Light phase CNT vs. Females:Light phase CRS	0,3471	-0,05325 to 0,7475	No	ns	0,1308			
Males:Light phase CNT vs. Females:Dark phase CNT	0,2926	-0,1077 to 0,6930	No	ns	0,2982			
Males:Light phase CNT vs. Females:Dark phase CRS	0,5636	0,1633 to 0,9640	Yes	**	0,0014			
Males:Light phase CRS vs. Males:Dark phase CNT	0,2000	-0,1817 to 0,5817	No	ns	0,6999			
Males:Light phase CRS vs. Males:Dark phase CRS	0,1557	-0,2261 to 0,5374	No	ns	0,8905			
Males:Light phase CRS vs. Females:Light phase CNT	0,06440	-0,3360 to 0,4648	No	ns	0,9995			
Males:Light phase CRS vs. Females:Light phase CRS	0,1143	-0,2674 to 0,4961	No	ns	0,9774			
Males:Light phase CRS vs. Females:Dark phase CNT	0,05983	-0,3219 to 0,4416	No	ns	0,9996			
Males:Light phase CRS vs. Females:Dark phase CRS	0,3308	-0,05092 to 0,7126	No	ns	0,1311			
Males:Dark phase CNT vs. Males:Dark phase CRS	-0,04433	-0,4261 to 0,3374	No	ns	>0,9999			
Males:Dark phase CNT vs. Females:Light phase CNT	-0,1356	-0,5360 to 0,2648	No	ns	0,9560			
Males:Dark phase CNT vs. Females:Light phase CRS	-0,08567	-0,4674 to 0,2961	No	ns	0,9959			
Males:Dark phase CNT vs. Females:Dark phase CNT	-0,1402	-0,5219 to 0,2416	No	ns	0,9336			
Males:Dark phase CNT vs. Females:Dark phase CRS	0,1308	-0,2509 to 0,5126	No	ns	0,9532			
Males:Dark phase CRS vs. Females:Light phase CNT	-0,09127	-0,4916 to 0,3091	No	ns	0,9954			

Males:Dark phase CRS vs. Females:Light phase CRS	-0,04133	-0,4231 to 0,3404	No	ns	>0,9999			
Males:Dark phase CRS vs. Females:Dark phase CNT	-0,09583	-0,4776 to 0,2859	No	ns	0,9918			
Males:Dark phase CRS vs. Females:Dark phase CRS	0,1752	-0,2066 to 0,5569	No	ns	0,8176			
Females:Light phase CNT vs. Females:Light phase CRS	0,04993	-0,3504 to 0,4503	No	ns	>0,9999			
Females:Light phase CNT vs. Females:Dark phase CNT	-0,004567	-0,4049 to 0,3958	No	ns	>0,9999			
Females:Light phase CNT vs. Females:Dark phase CRS	0,2664	-0,1339 to 0,6668	No	ns	0,4133			
Females:Light phase CRS vs. Females:Dark phase CNT	-0,05450	-0,4362 to 0,3272	No	ns	0,9998			
Females:Light phase CRS vs. Females:Dark phase CRS	0,2165	-0,1652 to 0,5982	No	ns	0,6124			
Females:Dark phase CNT vs. Females:Dark phase CRS	0,2710	-0,1107 to 0,6527	No	ns	0,3329			
Test details	Predicted (LS) mean 1	Predicted (LS) mean 2	Predicted (LS) mean diff,	SE of diff,	N1	N2	q	DF
Males:Light phase CNT vs. Males:Light phase CRS	1,029	0,7960	0,2328	0,1249	5	6	2,636	38,0
Males:Light phase CNT vs. Males:Dark phase CNT	1,029	0,5960	0,4328	0,1249	5	6	4,900	38,0
Males:Light phase CNT vs. Males:Dark phase CRS	1,029	0,6403	0,3885	0,1249	5	6	4,398	38,0
Males:Light phase CNT vs. Females:Light phase CNT	1,029	0,7316	0,2972	0,1305	5	5	3,222	38,0
Males:Light phase CNT vs. Females:Light phase CRS	1,029	0,6817	0,3471	0,1249	5	6	3,930	38,0
Males:Light phase CNT vs. Females:Dark phase CNT	1,029	0,7362	0,2926	0,1249	5	6	3,313	38,0
Males:Light phase CNT vs. Females:Dark phase CRS	1,029	0,4652	0,5636	0,1249	5	6	6,382	38,0
Males:Light phase CRS vs. Males:Dark phase CNT	0,7960	0,5960	0,2000	0,1191	6	6	2,375	38,0
Males:Light phase CRS vs. Males:Dark phase CRS	0,7960	0,6403	0,1557	0,1191	6	6	1,849	38,0
Males:Light phase CRS vs. Females:Light phase CNT	0,7960	0,7316	0,06440	0,1249	6	5	0,7292	38,0
Males:Light phase CRS vs. Females:Light phase CRS	0,7960	0,6817	0,1143	0,1191	6	6	1,358	38,0
Males:Light phase CRS vs. Females:Dark phase CNT	0,7960	0,7362	0,05983	0,1191	6	6	0,7105	38,0
Males:Light phase CRS vs. Females:Dark phase CRS	0,7960	0,4652	0,3308	0,1191	6	6	3,929	38,0
Males:Dark phase CNT vs. Males:Dark phase CRS	0,5960	0,6403	-0,04433	0,1191	6	6	0,5265	38,0
Males:Dark phase CNT vs. Females:Light phase CNT	0,5960	0,7316	-0,1356	0,1249	6	5	1,535	38,0
Males:Dark phase CNT vs. Females:Light phase CRS	0,5960	0,6817	-0,08567	0,1191	6	6	1,017	38,0
Males:Dark phase CNT vs. Females:Dark phase CNT	0,5960	0,7362	-0,1402	0,1191	6	6	1,664	38,0
Males:Dark phase CNT vs. Females:Dark phase CRS	0,5960	0,4652	0,1308	0,1191	6	6	1,554	38,0
Males:Dark phase CRS vs. Females:Light phase CNT	0,6403	0,7316	-0,09127	0,1249	6	5	1,033	38,0
Males:Dark phase CRS vs. Females:Light phase CRS	0,6403	0,6817	-0,04133	0,1191	6	6	0,4908	38,0
Males:Dark phase CRS vs. Females:Dark phase CNT	0,6403	0,7362	-0,09583	0,1191	6	6	1,138	38,0
Males:Dark phase CRS vs. Females:Dark phase CRS	0,6403	0,4652	0,1752	0,1191	6	6	2,080	38,0
Females:Light phase CNT vs. Females:Light phase CRS	0,7316	0,6817	0,04993	0,1249	5	6	0,5654	38,0
Females:Light phase CNT vs. Females:Dark phase CNT	0,7316	0,7362	-0,004567	0,1249	5	6	0,0517	38,0
Females:Light phase CNT vs. Females:Dark phase CRS	0,7316	0,4652	0,2664	0,1249	5	6	3,017	38,0
Females:Light phase CRS vs. Females:Dark phase CNT	0,6817	0,7362	-0,05450	0,1191	6	6	0,6472	38,0
Females:Light phase CRS vs. Females:Dark phase CRS	0,6817	0,4652	0,2165	0,1191	6	6	2,571	38,00
Females:Dark phase CNT vs. Females:Dark phase CRS	0,7362	0,4652	0,2710	0,1191	6	6	3,218	38,00

Statistics of Figure 5d

Table Analyzed	Cry1 Grouped: Three-way ANOVA (2x2x2)				
Three-way ANOVA	Ordinary				
Alpha	0,05				
Source of Variation	% of total variation	P value	P value summary	Significant?	
sex	1,666	0,3210	ns	No	
light	17,41	0,0025	**	Yes	
stress	2,644	0,2131	ns	No	
sex x light	1,667	0,3209	ns	No	
sex x stress	2,340	0,2408	ns	No	
light x stress	13,06	0,0078	**	Yes	
sex x light x stress	3,396	0,1594	ns	No	
ANOVA table	SS (Type III)	DF	MS	F (DFn, DFd)	P value
sex	0,08683	1	0,08683	F (1, 36) = 1,012	P=0,3210
light	0,9074	1	0,9074	F (1, 36) = 10,58	P=0,0025
stress	0,1378	1	0,1378	F (1, 36) = 1,607	P=0,2131
sex x light	0,08690	1	0,08690	F (1, 36) = 1,013	P=0,3209
sex x stress	0,1220	1	0,1220	F (1, 36) = 1,422	P=0,2408
light x stress	0,6811	1	0,6811	F (1, 36) = 7,942	P=0,0078
sex x light x stress	0,1770	1	0,1770	F (1, 36) = 2,064	P=0,1594
Residual	3,087	36	0,08576		

Compare each cell mean with every other cell mean								
Number of families	1							
Number of comparisons per family	28							
Alpha	0,05							
Tukey's multiple comparisons test	Predicted (LS) mean diff,	95,00% CI of diff,	Below threshold?	Summary	Adjusted P Value			
Males:Light phase CNT vs. Males:Light phase CRS	-0,3406	-0,9108 to 0,2296	No	ns	0,5463			
Males:Light phase CNT vs. Males:Dark phase CNT	-0,5001	-1,070 to 0,07007	No	ns	0,1215			
Males:Light phase CNT vs. Males:Dark phase CRS	-0,5958	-1,166 to -0,02560	Yes	*	0,0352			
Males:Light phase CNT vs. Females:Light phase CNT	0,02160	-0,5740 to 0,6172	No	ns	>0,9999			
Males:Light phase CNT vs. Females:Light phase CRS	-0,3623	-0,9325 to 0,2079	No	ns	0,4689			
Males:Light phase CNT vs. Females:Dark phase CNT	-0,5548	-1,150 to 0,04076	No	ns	0,0831			
Males:Light phase CNT vs. Females:Dark phase CRS	-0,1842	-0,7798 to 0,4114	No	ns	0,9723			
Males:Light phase CRS vs. Males:Dark phase CNT	-0,1595	-0,7032 to 0,3842	No	ns	0,9793			
Males:Light phase CRS vs. Males:Dark phase CRS	-0,2552	-0,7988 to 0,2885	No	ns	0,7976			
Males:Light phase CRS vs. Females:Light phase CNT	0,3622	-0,2080 to 0,9324	No	ns	0,4692			
Males:Light phase CRS vs. Females:Light phase CRS	-0,02167	-0,5653 to 0,5220	No	ns	>0,9999			
Males:Light phase CRS vs. Females:Dark phase CNT	-0,2142	-0,7844 to 0,3560	No	ns	0,9244			
Males:Light phase CRS vs. Females:Dark phase CRS	0,1564	-0,4138 to 0,7266	No	ns	0,9859			
Males:Dark phase CNT vs. Males:Dark phase CRS	-0,09567	-0,6393 to 0,4480	No	ns	0,9991			
Males:Dark phase CNT vs. Females:Light phase CNT	0,5217	-0,04847 to 1,092	No	ns	0,0935			
Males:Dark phase CNT vs. Females:Light phase CRS	0,1378	-0,4058 to 0,6815	No	ns	0,9911			
Males:Dark phase CNT vs. Females:Dark phase CNT	-0,05467	-0,6249 to 0,5155	No	ns	>0,9999			
Males:Dark phase CNT vs. Females:Dark phase CRS	0,3159	-0,2543 to 0,8861	No	ns	0,6358			
Males:Dark phase CRS vs. Females:Light phase CNT	0,6174	0,04720 to 1,188	Yes	*	0,0260			

Males:Dark phase CRS vs. Females:Light phase CRS	0,2335	-0,3102 to 0,7772	No	ns	0,8598			
Males:Dark phase CRS vs. Females:Dark phase CNT	0,04100	-0,5292 to 0,6112	No	ns	>0,9999			
Males:Dark phase CRS vs. Females:Dark phase CRS	0,4116	-0,1586 to 0,9818	No	ns	0,3105			
Females:Light phase CNT vs. Females:Light phase CRS	-0,3839	-0,9541 to 0,1863	No	ns	0,3957			
Females:Light phase CNT vs. Females:Dark phase CNT	-0,5764	-1,172 to 0,01916	No	ns	0,0637			
Females:Light phase CNT vs. Females:Dark phase CRS	-0,2058	-0,8014 to 0,3898	No	ns	0,9501			
Females:Light phase CRS vs. Females:Dark phase CNT	-0,1925	-0,7627 to 0,3777	No	ns	0,9558			
Females:Light phase CRS vs. Females:Dark phase CRS	0,1781	-0,3921 to 0,7483	No	ns	0,9708			
Females:Dark phase CNT vs. Females:Dark phase CRS	0,3706	-0,2250 to 0,9662	No	ns	0,4953			
Test details	Predicted (LS) mean 1	Predicted (LS) mean 2	Predicted (LS) mean diff,	SE of diff,	N1	N2	q	DF
Males:Light phase CNT vs. Males:Light phase CRS	1,009	1,350	-0,3406	0,1773	5	6	2,717	36,00
Males:Light phase CNT vs. Males:Dark phase CNT	1,009	1,509	-0,5001	0,1773	5	6	3,989	36,00
Males:Light phase CNT vs. Males:Dark phase CRS	1,009	1,605	-0,5958	0,1773	5	6	4,751	36,00
Males:Light phase CNT vs. Females:Light phase CNT	1,009	0,9876	0,02160	0,1852	5	5	0,1649	36,00
Males:Light phase CNT vs. Females:Light phase CRS	1,009	1,372	-0,3623	0,1773	5	6	2,889	36,00
Males:Light phase CNT vs. Females:Dark phase CNT	1,009	1,564	-0,5548	0,1852	5	5	4,236	36,00
Males:Light phase CNT vs. Females:Dark phase CRS	1,009	1,193	-0,1842	0,1852	5	5	1,406	36,00
Males:Light phase CRS vs. Males:Dark phase CNT	1,350	1,509	-0,1595	0,1691	6	6	1,334	36,00
Males:Light phase CRS vs. Males:Dark phase CRS	1,350	1,605	-0,2552	0,1691	6	6	2,134	36,00
Males:Light phase CRS vs. Females:Light phase CNT	1,350	0,9876	0,3622	0,1773	6	5	2,889	36,00
Males:Light phase CRS vs. Females:Light phase CRS	1,350	1,372	-0,02167	0,1691	6	6	0,1812	36,00
Males:Light phase CRS vs. Females:Dark phase CNT	1,350	1,564	-0,2142	0,1773	6	5	1,708	36,00
Males:Light phase CRS vs. Females:Dark phase CRS	1,350	1,193	0,1564	0,1773	6	5	1,248	36,00
Males:Dark phase CNT vs. Males:Dark phase CRS	1,509	1,605	-0,09567	0,1691	6	6	0,8002	36,00
Males:Dark phase CNT vs. Females:Light phase CNT	1,509	0,9876	0,5217	0,1773	6	5	4,161	36,00
Males:Dark phase CNT vs. Females:Light phase CRS	1,509	1,372	0,1378	0,1691	6	6	1,153	36,00
Males:Dark phase CNT vs. Females:Dark phase CNT	1,509	1,564	-0,05467	0,1773	6	5	0,4360	36,00
Males:Dark phase CNT vs. Females:Dark phase CRS	1,509	1,193	0,3159	0,1773	6	5	2,520	36,00
Males:Dark phase CRS vs. Females:Light phase CNT	1,605	0,9876	0,6174	0,1773	6	5	4,924	36,00
Males:Dark phase CRS vs. Females:Light phase CRS	1,605	1,372	0,2335	0,1691	6	6	1,953	36,00
Males:Dark phase CRS vs. Females:Dark phase CNT	1,605	1,564	0,04100	0,1773	6	5	0,3270	36,00
Males:Dark phase CRS vs. Females:Dark phase CRS	1,605	1,193	0,4116	0,1773	6	5	3,282	36,00
Females:Light phase CNT vs. Females:Light phase CRS	0,9876	1,372	-0,3839	0,1773	5	6	3,062	36,00
Females:Light phase CNT vs. Females:Dark phase CNT	0,9876	1,564	-0,5764	0,1852	5	5	4,401	36,00
Females:Light phase CNT vs. Females:Dark phase CRS	0,9876	1,193	-0,2058	0,1852	5	5	1,571	36,00
Females:Light phase CRS vs. Females:Dark phase CNT	1,372	1,564	-0,1925	0,1773	6	5	1,535	36,00
Females:Light phase CRS vs. Females:Dark phase CRS	1,372	1,193	0,1781	0,1773	6	5	1,420	36,00
Females:Dark phase CNT vs. Females:Dark phase CRS	1,564	1,193	0,3706	0,1852	5	5	2,830	36,00

Statistics of Figure 5e

Table Analyzed	Cry2 Grouped: Three-way ANOVA (2x2x2)				
Three-way ANOVA	Ordinary				
Alpha	0,05				
Source of Variation	% of total variation	P value	P value summary	Significant?	
sex	0,2174	0,7235	ns	No	
light	2,360	0,2476	ns	No	
stress	4,294	0,1217	ns	No	
sex x light	19,43	0,0018	**	Yes	
sex x stress	9,674	0,0228	*	Yes	
light x stress	0,1190	0,7934	ns	No	
sex x light x stress	2,387	0,2450	ns	No	
ANOVA table	SS (Type III)	DF	MS	F (DFn, DFd)	P value
sex	0,004555	1	0,004555	F (1, 36) = 0,1272	P=0,7235
light	0,04946	1	0,04946	F (1, 36) = 1,381	P=0,2476
stress	0,08997	1	0,08997	F (1, 36) = 2,512	P=0,1217
sex x light	0,4071	1	0,4071	F (1, 36) = 11,37	P=0,0018
sex x stress	0,2027	1	0,2027	F (1, 36) = 5,660	P=0,0228
light x stress	0,002493	1	0,002493	F (1, 36) = 0,06961	P=0,7934
sex x light x stress	0,05002	1	0,05002	F (1, 36) = 1,397	P=0,2450
Residual	1,289	36	0,03581		

Compare each cell mean with every other cell mean								
Number of families	1							
Number of comparisons per family	28							
Alpha	0,05							
Tukey's multiple comparisons test	Predicted (LS) Mean diff,	95,00% CI of diff,	Below threshold?	Summary	Adjusted P Value			
Males:Light phase CNT vs. Males:Light phase CRS	0,007100	-0,3614 to 0,3756	No	ns	>0,9999			
Males:Light phase CNT vs. Males:Dark phase CNT	-0,2079	-0,5764 to 0,1606	No	ns	0,6150			
Males:Light phase CNT vs. Males:Dark phase CRS	-0,3060	-0,6909 to 0,07885	No	ns	0,2053			
Males:Light phase CNT vs. Females:Light phase CNT	-0,2822	-0,6671 to 0,1027	No	ns	0,2922			
Males:Light phase CNT vs. Females:Light phase CRS	-0,1379	-0,5064 to 0,2306	No	ns	0,9257			
Males:Light phase CNT vs. Females:Dark phase CNT	-0,2392	-0,6241 to 0,1457	No	ns	0,4968			
Males:Light phase CNT vs. Females:Dark phase CRS	0,07077	-0,2977 to 0,4392	No	ns	0,9984			
Males:Light phase CRS vs. Males:Dark phase CNT	-0,2150	-0,5663 to 0,1363	No	ns	0,5163			
Males:Light phase CRS vs. Males:Dark phase CRS	-0,3131	-0,6816 to 0,05537	No	ns	0,1457			
Males:Light phase CRS vs. Females:Light phase CNT	-0,2893	-0,6578 to 0,07917	No	ns	0,2179			
Males:Light phase CRS vs. Females:Light phase CRS	-0,1450	-0,4963 to 0,2063	No	ns	0,8824			
Males:Light phase CRS vs. Females:Dark phase CNT	-0,2463	-0,6148 to 0,1222	No	ns	0,4047			
Males:Light phase CRS vs. Females:Dark phase CRS	0,06367	-0,2877 to 0,4150	No	ns	0,9989			
Males:Dark phase CNT vs. Males:Dark phase CRS	-0,09810	-0,4666 to 0,2704	No	ns	0,9881			
Males:Dark phase CNT vs. Females:Light phase CNT	-0,07430	-0,4428 to 0,2942	No	ns	0,9978			
Males:Dark phase CNT vs. Females:Light phase CRS	0,07000	-0,2813 to 0,4213	No	ns	0,9980			
Males:Dark phase CNT vs. Females:Dark phase CNT	-0,03130	-0,3998 to 0,3372	No	ns	>0,9999			
Males:Dark phase CNT vs. Females:Dark phase CRS	0,2787	-0,07266 to 0,6300	No	ns	0,2077			
Males:Dark phase CRS vs. Females:Light phase CNT	0,02380	-0,3611 to 0,4087	No	ns	>0,9999			

Males:Dark phase CRS vs. Females:Light phase CRS	0,1681	-0,2004 to 0,5366	No	ns	0,8193			
Males:Dark phase CRS vs. Females:Dark phase CNT	0,06680	-0,3181 to 0,4517	No	ns	0,9992			
Males:Dark phase CRS vs. Females:Dark phase CRS	0,3768	0,008297 to 0,7452	Yes	*	0,0420			
Females:Light phase CNT vs. Females:Light phase CRS	0,1443	-0,2242 to 0,5128	No	ns	0,9076			
Females:Light phase CNT vs. Females:Dark phase CNT	0,04300	-0,3419 to 0,4279	No	ns	>0,9999			
Females:Light phase CNT vs. Females:Dark phase CRS	0,3530	-0,01550 to 0,7214	No	ns	0,0686			
Females:Light phase CRS vs. Females:Dark phase CNT	-0,1013	-0,4698 to 0,2672	No	ns	0,9857			
Females:Light phase CRS vs. Females:Dark phase CRS	0,2087	-0,1427 to 0,5600	No	ns	0,5534			
Females:Dark phase CNT vs. Females:Dark phase CRS	0,3100	-0,05850 to 0,6784	No	ns	0,1540			
Test details	Predicted (LS) Mean 1	Predicted (LS) Mean 2	Predicted (LS) Mean diff,	SE of diff,	N1	N2	q	DF
Males:Light phase CNT vs. Males:Light phase CRS	1,013	1,006	0,007100	0,1146	5	6	0,08762	36,0
Males:Light phase CNT vs. Males:Dark phase CNT	1,013	1,221	-0,2079	0,1146	5	6	2,566	36,0
Males:Light phase CNT vs. Males:Dark phase CRS	1,013	1,319	-0,3060	0,1197	5	5	3,616	36,0
Males:Light phase CNT vs. Females:Light phase CNT	1,013	1,295	-0,2822	0,1197	5	5	3,334	36,0
Males:Light phase CNT vs. Females:Light phase CRS	1,013	1,151	-0,1379	0,1146	5	6	1,702	36,0
Males:Light phase CNT vs. Females:Dark phase CNT	1,013	1,252	-0,2392	0,1197	5	5	2,826	36,0
Males:Light phase CNT vs. Females:Dark phase CRS	1,013	0,9418	0,07077	0,1146	5	6	0,8733	36,0
Males:Light phase CRS vs. Males:Dark phase CNT	1,006	1,221	-0,2150	0,1093	6	6	2,783	36,0
Males:Light phase CRS vs. Males:Dark phase CRS	1,006	1,319	-0,3131	0,1146	6	5	3,864	36,0
Males:Light phase CRS vs. Females:Light phase CNT	1,006	1,295	-0,2893	0,1146	6	5	3,570	36,0
Males:Light phase CRS vs. Females:Light phase CRS	1,006	1,151	-0,1450	0,1093	6	6	1,877	36,0
Males:Light phase CRS vs. Females:Dark phase CNT	1,006	1,252	-0,2463	0,1146	6	5	3,040	36,0
Males:Light phase CRS vs. Females:Dark phase CRS	1,006	0,9418	0,06367	0,1093	6	6	0,8241	36,0
Males:Dark phase CNT vs. Males:Dark phase CRS	1,221	1,319	-0,09810	0,1146	6	5	1,211	36,0
Males:Dark phase CNT vs. Females:Light phase CNT	1,221	1,295	-0,07430	0,1146	6	5	0,9169	36,0
Males:Dark phase CNT vs. Females:Light phase CRS	1,221	1,151	0,07000	0,1093	6	6	0,9060	36,0
Males:Dark phase CNT vs. Females:Dark phase CNT	1,221	1,252	-0,03130	0,1146	6	5	0,3863	36,0
Males:Dark phase CNT vs. Females:Dark phase CRS	1,221	0,9418	0,2787	0,1093	6	6	3,607	36,0
Males:Dark phase CRS vs. Females:Light phase CNT	1,319	1,295	0,02380	0,1197	5	5	0,2812	36,0
Males:Dark phase CRS vs. Females:Light phase CRS	1,319	1,151	0,1681	0,1146	5	6	2,075	36,0
Males:Dark phase CRS vs. Females:Dark phase CNT	1,319	1,252	0,06680	0,1197	5	5	0,7893	36,0
Males:Dark phase CRS vs. Females:Dark phase CRS	1,319	0,9418	0,3768	0,1146	5	6	4,650	36,0
Females:Light phase CNT vs. Females:Light phase CRS	1,295	1,151	0,1443	0,1146	5	6	1,781	36,0
Females:Light phase CNT vs. Females:Dark phase CNT	1,295	1,252	0,04300	0,1197	5	5	0,5081	36,0
Females:Light phase CNT vs. Females:Dark phase CRS	1,295	0,9418	0,3530	0,1146	5	6	4,356	36,0
Females:Light phase CRS vs. Females:Dark phase CNT	1,151	1,252	-0,1013	0,1146	6	5	1,250	36,0
Females:Light phase CRS vs. Females:Dark phase CRS	1,151	0,9418	0,2087	0,1093	6	6	2,701	36,0
Females:Dark phase CNT vs. Females:Dark phase CRS	1,252	0,9418	0,3100	0,1146	5	6	3,825	36,0

Statistics of Figure 5f

Table Analyzed	Per1 Grouped: Three-way ANOVA (2x2x2)				
Three-way ANOVA	Ordinary				
Alpha	0,05				
Source of Variation	% of total variation	P value	P value summary	Significant?	
sex	5,410	0,0977	ns	No	
light	2,607	0,2454	ns	No	
stress	0,1189	0,8023	ns	No	
sex x light	0,001969	0,9743	ns	No	
sex x stress	18,85	0,0031	**	Yes	
light x stress	2,032	0,3042	ns	No	
sex x light x stress	4,311	0,1377	ns	No	
ANOVA table	SS (Type III)	DF	MS	F (DFn, DFd)	P value
sex	0,3745	1	0,3745	F (1, 35) = 2,896	P=0,0977
light	0,1805	1	0,1805	F (1, 35) = 1,396	P=0,2454
stress	0,008229	1	0,008229	F (1, 35) = 0,06363	P=0,8023
sex x light	0,0001363	1	0,0001363	F (1, 35) = 0,001054	P=0,9743
sex x stress	1,305	1	1,305	F (1, 35) = 10,09	P=0,0031
light x stress	0,1406	1	0,1406	F (1, 35) = 1,087	P=0,3042
sex x light x stress	0,2985	1	0,2985	F (1, 35) = 2,308	P=0,1377
Residual	4,526	35	0,1293		

Compare each cell mean with every other cell mean								
Number of families	1							
Number of comparisons per family	28							
Alpha	0,05							
Tukey's multiple comparisons test	Predicted (LS) mean diff,	95,00% CI of diff,	Below threshold?	Summary	Adjusted P Value			
Males:Light phase CNT vs. Males:Light phase CRS	-0,2695	-0,9709 to 0,4319	No	ns	0,9147			
Males:Light phase CNT vs. Males:Dark phase CNT	-0,08120	-0,8138 to 0,6514	No	ns	>0,9999			
Males:Light phase CNT vs. Males:Dark phase CRS	-0,4556	-1,188 to 0,2770	No	ns	0,4943			
Males:Light phase CNT vs. Females:Light phase CNT	-0,3734	-1,106 to 0,3592	No	ns	0,7226			
Males:Light phase CNT vs. Females:Light phase CRS	-0,2780	-0,9794 to 0,4234	No	ns	0,9013			
Males:Light phase CNT vs. Females:Dark phase CNT	-0,7820	-1,483 to -0,08062	Yes	*	0,0200			
Males:Light phase CNT vs. Females:Dark phase CRS	-0,1224	-0,8550 to 0,6102	No	ns	0,9993			
Males:Light phase CRS vs. Males:Dark phase CNT	0,1883	-0,5131 to 0,8897	No	ns	0,9874			
Males:Light phase CRS vs. Males:Dark phase CRS	-0,1861	-0,8875 to 0,5153	No	ns	0,9882			
Males:Light phase CRS vs. Females:Light phase CNT	-0,1039	-0,8053 to 0,5975	No	ns	0,9997			
Males:Light phase CRS vs. Females:Light phase CRS	-0,008500	-0,6772 to 0,6602	No	ns	>0,9999			
Males:Light phase CRS vs. Females:Dark phase CNT	-0,5125	-1,181 to 0,1562	No	ns	0,2421			
Males:Light phase CRS vs. Females:Dark phase CRS	0,1471	-0,5543 to 0,8485	No	ns	0,9972			
Males:Dark phase CNT vs. Males:Dark phase CRS	-0,3744	-1,107 to 0,3582	No	ns	0,7199			
Males:Dark phase CNT vs. Females:Light phase CNT	-0,2922	-1,025 to 0,4404	No	ns	0,8984			
Males:Dark phase CNT vs. Females:Light phase CRS	-0,1968	-0,8982 to 0,5046	No	ns	0,9837			
Males:Dark phase CNT vs. Females:Dark phase CNT	-0,7008	-1,402 to 0,0005756	No	ns	0,0503			
Males:Dark phase CNT vs. Females:Dark phase CRS	-0,04120	-0,7738 to 0,6914	No	ns	>0,9999			
Males:Dark phase CRS vs. Females:Light phase CNT	0,08220	-0,6504 to 0,8148	No	ns	>0,9999			

Males:Dark phase CRS vs. Females:Light phase CRS	0,1776	-0,5238 to 0,8790	No	ns	0,9910			
Males:Dark phase CRS vs. Females:Dark phase CNT	-0,3264	-1,028 to 0,3750	No	ns	0,8028			
Males:Dark phase CRS vs. Females:Dark phase CRS	0,3332	-0,3994 to 1,066	No	ns	0,8201			
Females:Light phase CNT vs. Females:Light phase CRS	0,09540	-0,6060 to 0,7968	No	ns	0,9998			
Females:Light phase CNT vs. Females:Dark phase CNT	-0,4086	-1,110 to 0,2928	No	ns	0,5752			
Females:Light phase CNT vs. Females:Dark phase CRS	0,2510	-0,4816 to 0,9836	No	ns	0,9517			
Females:Light phase CRS vs. Females:Dark phase CNT	-0,5040	-1,173 to 0,1647	No	ns	0,2602			
Females:Light phase CRS vs. Females:Dark phase CRS	0,1556	-0,5458 to 0,8570	No	ns	0,9960			
Females:Dark phase CNT vs. Females:Dark phase CRS	0,6596	-0,04178 to 1,361	No	ns	0,0778			

Test details	Predicted (LS) mean 1	Predicted (LS) mean 2	Predicted (LS) mean diff,	SE of diff,	N1	N2	q	DF
Males:Light phase CNT vs. Males:Light phase CRS	1,022	1,292	-0,2695	0,2178	5	6	1,750	35,0
Males:Light phase CNT vs. Males:Dark phase CNT	1,022	1,103	-0,08120	0,2274	5	5	0,5049	35,0
Males:Light phase CNT vs. Males:Dark phase CRS	1,022	1,478	-0,4556	0,2274	5	5	2,833	35,0
Males:Light phase CNT vs. Females:Light phase CNT	1,022	1,395	-0,3734	0,2274	5	5	2,322	35,0
Males:Light phase CNT vs. Females:Light phase CRS	1,022	1,300	-0,2780	0,2178	5	6	1,805	35,0
Males:Light phase CNT vs. Females:Dark phase CNT	1,022	1,804	-0,7820	0,2178	5	6	5,079	35,0
Males:Light phase CNT vs. Females:Dark phase CRS	1,022	1,144	-0,1224	0,2274	5	5	0,7611	35,0
Males:Light phase CRS vs. Males:Dark phase CNT	1,292	1,103	0,1883	0,2178	6	5	1,223	35,0
Males:Light phase CRS vs. Males:Dark phase CRS	1,292	1,478	-0,1861	0,2178	6	5	1,209	35,0
Males:Light phase CRS vs. Females:Light phase CNT	1,292	1,395	-0,1039	0,2178	6	5	0,6748	35,0
Males:Light phase CRS vs. Females:Light phase CRS	1,292	1,300	-0,008500	0,2076	6	6	0,05790	35,0
Males:Light phase CRS vs. Females:Dark phase CNT	1,292	1,804	-0,5125	0,2076	6	6	3,491	35,0
Males:Light phase CRS vs. Females:Dark phase CRS	1,292	1,144	0,1471	0,2178	6	5	0,9553	35,0
Males:Dark phase CNT vs. Males:Dark phase CRS	1,103	1,478	-0,3744	0,2274	5	5	2,328	35,0
Males:Dark phase CNT vs. Females:Light phase CNT	1,103	1,395	-0,2922	0,2274	5	5	1,817	35,0
Males:Dark phase CNT vs. Females:Light phase CRS	1,103	1,300	-0,1968	0,2178	5	6	1,278	35,0
Males:Dark phase CNT vs. Females:Dark phase CNT	1,103	1,804	-0,7008	0,2178	5	6	4,551	35,0
Males:Dark phase CNT vs. Females:Dark phase CRS	1,103	1,144	-0,04120	0,2274	5	5	0,2562	35,0
Males:Dark phase CRS vs. Females:Light phase CNT	1,478	1,395	0,08220	0,2274	5	5	0,5111	35,0
Males:Dark phase CRS vs. Females:Light phase CRS	1,478	1,300	0,1776	0,2178	5	6	1,153	35,0
Males:Dark phase CRS vs. Females:Dark phase CNT	1,478	1,804	-0,3264	0,2178	5	6	2,120	35,0
Males:Dark phase CRS vs. Females:Dark phase CRS	1,478	1,144	0,3332	0,2274	5	5	2,072	35,0
Females:Light phase CNT vs. Females:Light phase CRS	1,395	1,300	0,09540	0,2178	5	6	0,6196	35,0
Females:Light phase CNT vs. Females:Dark phase CNT	1,395	1,804	-0,4086	0,2178	5	6	2,654	35,0
Females:Light phase CNT vs. Females:Dark phase CRS	1,395	1,144	0,2510	0,2274	5	5	1,561	35,0
Females:Light phase CRS vs. Females:Dark phase CNT	1,300	1,804	-0,5040	0,2076	6	6	3,433	35,0
Females:Light phase CRS vs. Females:Dark phase CRS	1,300	1,144	0,1556	0,2178	6	5	1,011	35,0
Females:Dark phase CNT vs. Females:Dark phase CRS	1,804	1,144	0,6596	0,2178	6	5	4,284	35,0

Statistics of Figure 5g

Table Analyzed	Per2 Grouped: Three-way ANOVA (2x2x2)				
Three-way ANOVA	Ordinary				
Alpha	0,05				
Source of Variation	% of total variation	P value	P value summary	Significant?	
sex	2,066	0,2900	ns	No	
light	4,688	0,1143	ns	No	
stress	15,27	0,0059	**	Yes	
sex x light	7,611	0,0464	*	Yes	
sex x stress	0,6449	0,5523	ns	No	
light x stress	0,9400	0,4735	ns	No	
sex x light x stress	0,9615	0,4685	ns	No	
ANOVA table	SS (Type III)	DF	MS	F (DFn, DFd)	P value
sex	0,1164	1	0,1164	F (1, 37) = 1,152	P=0,2900
light	0,2643	1	0,2643	F (1, 37) = 2,616	P=0,1143
stress	0,8607	1	0,8607	F (1, 37) = 8,519	P=0,0059
sex x light	0,4290	1	0,4290	F (1, 37) = 4,246	P=0,0464
sex x stress	0,03635	1	0,03635	F (1, 37) = 0,3598	P=0,5523
light x stress	0,05299	1	0,05299	F (1, 37) = 0,5245	P=0,4735
sex x light x stress	0,05420	1	0,05420	F (1, 37) = 0,5365	P=0,4685
Residual	3,738	37	0,1010		

Compare each cell mean with every other cell mean								
Number of families	1							
Number of comparisons per family	28							
Alpha	0,05							
Tukey's multiple comparisons test	Predicted (LS) Mean diff,	95,00% CI of diff,	Below threshold?	Summary	Adjusted P Value			
Males:Light phase CNT vs. Males:Light phase CRS	0,2214	-0,4240 to 0,8668	No	ns	0,9525			
Males:Light phase CNT vs. Males:Dark phase CNT	0,04297	-0,5750 to 0,6609	No	ns	>0,9999			
Males:Light phase CNT vs. Males:Dark phase CRS	0,2628	-0,3551 to 0,8807	No	ns	0,8667			
Males:Light phase CNT vs. Females:Light phase CNT	0,3108	-0,3346 to 0,9562	No	ns	0,7778			
Males:Light phase CNT vs. Females:Light phase CRS	0,5070	-0,1110 to 1,125	No	ns	0,1763			
Males:Light phase CNT vs. Females:Dark phase CNT	-0,1777	-0,7956 to 0,4402	No	ns	0,9818			
Males:Light phase CNT vs. Females:Dark phase CRS	0,2956	-0,3223 to 0,9136	No	ns	0,7833			
Males:Light phase CRS vs. Males:Dark phase CNT	-0,1784	-0,7964 to 0,4395	No	ns	0,9813			
Males:Light phase CRS vs. Males:Dark phase CRS	0,04140	-0,5765 to 0,6593	No	ns	>0,9999			
Males:Light phase CRS vs. Females:Light phase CNT	0,08940	-0,5560 to 0,7348	No	ns	0,9998			
Males:Light phase CRS vs. Females:Light phase CRS	0,2856	-0,3324 to 0,9035	No	ns	0,8110			
Males:Light phase CRS vs. Females:Dark phase CNT	-0,3991	-1,017 to 0,2188	No	ns	0,4497			
Males:Light phase CRS vs. Females:Dark phase CRS	0,07423	-0,5437 to 0,6922	No	ns	>0,9999			
Males:Dark phase CNT vs. Males:Dark phase CRS	0,2198	-0,3693 to 0,8090	No	ns	0,9275			
Males:Dark phase CNT vs. Females:Light phase CNT	0,2678	-0,3501 to 0,8858	No	ns	0,8553			
Males:Dark phase CNT vs. Females:Light phase CRS	0,4640	-0,1252 to 1,053	No	ns	0,2156			

Males:Dark phase CNT vs. Females:Dark phase CNT	-0,2207	-0,8098 to 0,3685	No	ns	0,9261				
Males:Dark phase CNT vs. Females:Dark phase CRS	0,2527	-0,3365 to 0,8418	No	ns	0,8618				
Males:Dark phase CRS vs. Females:Light phase CNT	0,04800	-0,5699 to 0,6659	No	ns	>0,9999				
Males:Dark phase CRS vs. Females:Light phase CRS	0,2442	-0,3450 to 0,8333	No	ns	0,8812				
Males:Dark phase CRS vs. Females:Dark phase CNT	-0,4405	-1,030 to 0,1487	No	ns	0,2711				
Males:Dark phase CRS vs. Females:Dark phase CRS	0,03283	-0,5563 to 0,6220	No	ns	>0,9999				
Females:Light phase CNT vs. Females:Light phase CRS	0,1962	-0,4218 to 0,8141	No	ns	0,9685				
Females:Light phase CNT vs. Females:Dark phase CNT	-0,4885	-1,106 to 0,1294	No	ns	0,2117				
Females:Light phase CNT vs. Females:Dark phase CRS	-0,01517	-0,6331 to 0,6028	No	ns	>0,9999				
Females:Light phase CRS vs. Females:Dark phase CNT	-0,6847	-1,274 to -0,09550	Yes	*	0,0133				
Females:Light phase CRS vs. Females:Dark phase CRS	-0,2113	-0,8005 to 0,3778	No	ns	0,9403				
Females:Dark phase CNT vs. Females:Dark phase CRS	0,4733	-0,1158 to 1,062	No	ns	0,1959				
Test details	Predicted (LS) Mean 1	Predicted (LS) Mean 2	Predicted (LS) Mean diff,	SE of diff,	N1	N2	q	DF	
Males:Light phase CNT vs. Males:Light phase CRS	1,009	0,7874	0,2214	0,2010	5	5	1,557	37,00	
Males:Light phase CNT vs. Males:Dark phase CNT	1,009	0,9658	0,04297	0,1925	5	6	0,3157	37,00	
Males:Light phase CNT vs. Males:Dark phase CRS	1,009	0,7460	0,2628	0,1925	5	6	1,931	37,00	
Males:Light phase CNT vs. Females:Light phase CNT	1,009	0,6980	0,3108	0,2010	5	5	2,186	37,00	
Males:Light phase CNT vs. Females:Light phase CRS	1,009	0,5018	0,5070	0,1925	5	6	3,725	37,00	
Males:Light phase CNT vs. Females:Dark phase CNT	1,009	1,187	-0,1777	0,1925	5	6	1,306	37,00	
Males:Light phase CNT vs. Females:Dark phase CRS	1,009	0,7132	0,2956	0,1925	5	6	2,172	37,00	
Males:Light phase CRS vs. Males:Dark phase CNT	0,7874	0,9658	-0,1784	0,1925	5	6	1,311	37,00	
Males:Light phase CRS vs. Males:Dark phase CRS	0,7874	0,7460	0,04140	0,1925	5	6	0,3042	37,00	
Males:Light phase CRS vs. Females:Light phase CNT	0,7874	0,6980	0,08940	0,2010	5	5	0,6289	37,00	
Males:Light phase CRS vs. Females:Light phase CRS	0,7874	0,5018	0,2856	0,1925	5	6	2,098	37,00	
Males:Light phase CRS vs. Females:Dark phase CNT	0,7874	1,187	-0,3991	0,1925	5	6	2,932	37,00	
Males:Light phase CRS vs. Females:Dark phase CRS	0,7874	0,7132	0,07423	0,1925	5	6	0,5454	37,00	
Males:Dark phase CNT vs. Males:Dark phase CRS	0,9658	0,7460	0,2198	0,1835	6	6	1,694	37,00	
Males:Dark phase CNT vs. Females:Light phase CNT	0,9658	0,6980	0,2678	0,1925	6	5	1,968	37,00	
Males:Dark phase CNT vs. Females:Light phase CRS	0,9658	0,5018	0,4640	0,1835	6	6	3,576	37,00	
Males:Dark phase CNT vs. Females:Dark phase CNT	0,9658	1,187	-0,2207	0,1835	6	6	1,700	37,00	
Males:Dark phase CNT vs. Females:Dark phase CRS	0,9658	0,7132	0,2527	0,1835	6	6	1,947	37,00	
Males:Dark phase CRS vs. Females:Light phase CNT	0,7460	0,6980	0,04800	0,1925	6	5	0,3527	37,00	
Males:Dark phase CRS vs. Females:Light phase CRS	0,7460	0,5018	0,2442	0,1835	6	6	1,882	37,00	
Males:Dark phase CRS vs. Females:Dark phase CNT	0,7460	1,187	-0,4405	0,1835	6	6	3,394	37,00	
Males:Dark phase CRS vs. Females:Dark phase CRS	0,7460	0,7132	0,03283	0,1835	6	6	0,2530	37,00	
Females:Light phase CNT vs. Females:Light phase CRS	0,6980	0,5018	0,1962	0,1925	5	6	1,441	37,00	
Females:Light phase CNT vs. Females:Dark phase CNT	0,6980	1,187	-0,4885	0,1925	5	6	3,589	37,00	
Females:Light phase CNT vs. Females:Dark phase CRS	0,6980	0,7132	-0,01517	0,1925	5	6	0,1114	37,00	
Females:Light phase CRS vs. Females:Dark phase CNT	0,5018	1,187	-0,6847	0,1835	6	6	5,276	37,00	
Females:Light phase CRS vs. Females:Dark phase CRS	0,5018	0,7132	-0,2113	0,1835	6	6	1,629	37,00	
Females:Dark phase CNT vs. Females:Dark phase CRS	1,187	0,7132	0,4733	0,1835	6	6	3,648	37,00	
