# Supplementary material for: Sex and Circadian Rhythm Dependent Behavioral Effects of Chronic Stress in Mice and Modulation of Clock Genes in the Prefrontal Cortex
Source: Int J Mol Sci. 2025 Jul 3;26(13):6410. doi: 10.3390/ijms26136410 (PMC12250008; doi:10.3390/ijms26136410)
Supplement: Supplementary file 1 [file ijms-26-06410-s001.zip › Table S6.pdf]

Supplementary Table S6

Statistics of Figure 6

Table Analyzed	z-score molecolare				
Three-way ANOVA	Ordinary				
Alpha	0,05				
Source of Variation	% of total variation	P value	P value summary	Significant?	
sex	1,016	0,3322	ns	No	
light	22,38	<0,0001	****	Yes	
stress	1,206	0,2912	ns	No	
sex x light	3,140	0,0927	ns	No	
sex x stress	13,54	0,0010	**	Yes	
light x stress	19,25	0,0001	***	Yes	
sex x light x stress	4,571	0,0445	*	Yes	
ANOVA table	SS (Type III)	DF	MS	F (DFn, DFd)	P value
sex	4,138	1	4,138	F (1, 34) = 0,9679	P=0,3322
light	91,18	1	91,18	F (1, 34) = 21,33	P<0,0001
stress	4,914	1	4,914	F (1, 34) = 1,149	P=0,2912
sex x light	12,79	1	12,79	F (1, 34) = 2,992	P=0,0927
sex x stress	55,15	1	55,15	F (1, 34) = 12,90	P=0,0010
light x stress	78,41	1	78,41	F (1, 34) = 18,34	P=0,0001
sex x light x stress	18,62	1	18,62	F (1, 34) = 4,356	P=0,0445
Residual	145,4	34	4,276		

Compare each cell mean with every other cell mean								
Number of families	1							
Number of comparisons per family	28							
Alpha	0,05							
Tukey's multiple comparisons test	Predicted (LS) Mean diff,	95,00% CI of diff,	Below threshold?	Summary	Adjusted P Value			
Males:Light phase CNT vs. Males:Light phase CRS	-3,018	-7,238 to 1,202	No	ns	0,3188			
Males:Light phase CNT vs. Males:Dark phase CNT	-5,469	-9,509 to -1,429	Yes	**	0,0025			
Males:Light phase CNT vs. Males:Dark phase CRS	-5,676	-9,896 to -1,456	Yes	**	0,0027			
Males:Light phase CNT vs. Females:Light phase CNT	-2,700	-6,920 to 1,520	No	ns	0,4566			
Males:Light phase CNT vs. Females:Light phase CRS	-3,792	-8,012 to 0,4277	No	ns	0,1043			
Males:Light phase CNT vs. Females:Dark phase CNT	-8,626	-12,85 to -4,406	Yes	****	<0,0001			

Males:Light phase CNT vs. Females:Dark phase CRS	-1,564	-5,604 to 2,476	No	ns	0,9108			
Males:Light phase CRS vs. Males:Dark phase CNT	-2,451	-6,491 to 1,589	No	ns	0,5237			
Males:Light phase CRS vs. Males:Dark phase CRS	-2,658	-6,878 to 1,562	No	ns	0,4764			
Males:Light phase CRS vs. Females:Light phase CNT	0,3180	-3,902 to 4,538	No	ns	>0,9999			
Males:Light phase CRS vs. Females:Light phase CRS	-0,7740	-4,994 to 3,446	No	ns	0,9988			
Males:Light phase CRS vs. Females:Dark phase CNT	-5,608	-9,828 to -1,388	Yes	**	0,0032			
Males:Light phase CRS vs. Females:Dark phase CRS	1,454	-2,586 to 5,494	No	ns	0,9373			
Males:Dark phase CNT vs. Males:Dark phase CRS	-0,2073	-4,247 to 3,833	No	ns	>0,9999			
Males:Dark phase CNT vs. Females:Light phase CNT	2,769	-1,271 to 6,809	No	ns	0,3705			
Males:Dark phase CNT vs. Females:Light phase CRS	1,677	-2,363 to 5,717	No	ns	0,8772			
Males:Dark phase CNT vs. Females:Dark phase CNT	-3,157	-7,197 to 0,8827	No	ns	0,2208			
Males:Dark phase CNT vs. Females:Dark phase CRS	3,905	0,05296 to 7,757	Yes	*	0,0450			
Males:Dark phase CRS vs. Females:Light phase CNT	2,976	-1,244 to 7,196	No	ns	0,3355			
Males:Dark phase CRS vs. Females:Light phase CRS	1,884	-2,336 to 6,104	No	ns	0,8319			
Males:Dark phase CRS vs. Females:Dark phase CNT	-2,950	-7,170 to 1,270	No	ns	0,3461			
Males:Dark phase CRS vs. Females:Dark phase CRS	4,112	0,07228 to 8,152	Yes	*	0,0436			
Females:Light phase CNT vs. Females:Light phase CRS	-1,092	-5,312 to 3,128	No	ns	0,9897			
Females:Light phase CNT vs. Females:Dark phase CNT	-5,926	-10,15 to -1,706	Yes	**	0,0016			
Females:Light phase CNT vs. Females:Dark phase CRS	1,136	-2,904 to 5,176	No	ns	0,9833			
Females:Light phase CRS vs. Females:Dark phase CNT	-4,834	-9,054 to -0,6143	Yes	*	0,0156			
Females:Light phase CRS vs. Females:Dark phase CRS	2,228	-1,812 to 6,268	No	ns	0,6372			
Females:Dark phase CNT vs. Females:Dark phase CRS	7,062	3,022 to 11,10	Yes	****	<0,0001			

Test details	Predicted (LS) Mean 1	Predicted (LS) Mean 2	Predicted (LS) Mean diff,	SE of diff,	N1	N2	q	DF
Males:Light phase CNT vs. Males:Light phase CRS	-0,4520	2,566	-3,018	1,308	5	5	3,264	34,00

Males:Light phase CNT vs. Males:Dark phase CNT	-0,4520	5,017	-5,469	1,252	5	6	6,177	34,00
Males:Light phase CNT vs. Males:Dark phase CRS	-0,4520	5,224	-5,676	1,308	5	5	6,138	34,00
Males:Light phase CNT vs. Females:Light phase CNT	-0,4520	2,248	-2,700	1,308	5	5	2,920	34,00
Males:Light phase CNT vs. Females:Light phase CRS	-0,4520	3,340	-3,792	1,308	5	5	4,101	34,00
Males:Light phase CNT vs. Females:Dark phase CNT	-0,4520	8,174	-8,626	1,308	5	5	9,328	34,00
Males:Light phase CNT vs. Females:Dark phase CRS	-0,4520	1,112	-1,564	1,252	5	6	1,766	34,00
Males:Light phase CRS vs. Males:Dark phase CNT	2,566	5,017	-2,451	1,252	5	6	2,768	34,00
Males:Light phase CRS vs. Males:Dark phase CRS	2,566	5,224	-2,658	1,308	5	5	2,874	34,00
Males:Light phase CRS vs. Females:Light phase CNT	2,566	2,248	0,3180	1,308	5	5	0,3439	34,00
Males:Light phase CRS vs. Females:Light phase CRS	2,566	3,340	-0,7740	1,308	5	5	0,8370	34,00
Males:Light phase CRS vs. Females:Dark phase CNT	2,566	8,174	-5,608	1,308	5	5	6,064	34,00
Males:Light phase CRS vs. Females:Dark phase CRS	2,566	1,112	1,454	1,252	5	6	1,643	34,00
Males:Dark phase CNT vs. Males:Dark phase CRS	5,017	5,224	-0,2073	1,252	6	5	0,2342	34,00
Males:Dark phase CNT vs. Females:Light phase CNT	5,017	2,248	2,769	1,252	6	5	3,127	34,00
Males:Dark phase CNT vs. Females:Light phase CRS	5,017	3,340	1,677	1,252	6	5	1,894	34,00
Males:Dark phase CNT vs. Females:Dark phase CNT	5,017	8,174	-3,157	1,252	6	5	3,566	34,00
Males:Dark phase CNT vs. Females:Dark phase CRS	5,017	1,112	3,905	1,194	6	6	4,626	34,00
Males:Dark phase CRS vs. Females:Light phase CNT	5,224	2,248	2,976	1,308	5	5	3,218	34,00
Males:Dark phase CRS vs. Females:Light phase CRS	5,224	3,340	1,884	1,308	5	5	2,037	34,00
Males:Dark phase CRS vs. Females:Dark phase CNT	5,224	8,174	-2,950	1,308	5	5	3,190	34,00
Males:Dark phase CRS vs. Females:Dark phase CRS	5,224	1,112	4,112	1,252	5	6	4,645	34,00
Females:Light phase CNT vs. Females:Light phase CRS	2,248	3,340	-1,092	1,308	5	5	1,181	34,00

Females:Light phase CNT vs. Females:Dark phase CNT	2,248	8,174	-5,926	1,308	5	5	6,408	34,00
Females:Light phase CNT vs. Females:Dark phase CRS	2,248	1,112	1,136	1,252	5	6	1,283	34,00
Females:Light phase CRS vs. Females:Dark phase CNT	3,340	8,174	-4,834	1,308	5	5	5,227	34,00
Females:Light phase CRS vs. Females:Dark phase CRS	3,340	1,112	2,228	1,252	5	6	2,517	34,00
Females:Dark phase CNT vs. Females:Dark phase CRS	8,174	1,112	7,062	1,252	5	6	7,977	34,00
